# Stabilized Carbon‐Centered Radical‐Mediated Carbosulfenylation of Styrenes: Modular Synthesis of Sulfur‐Containing Glycine and Peptide Derivatives

**DOI:** 10.1002/advs.202402428

**Published:** 2024-06-09

**Authors:** Zihui Yang, Jia Liu, Lan‐Gui Xie

**Affiliations:** ^1^ National and Local Joint Engineering Research Center of Biomedical Functional Materials Jiangsu Key Laboratory of New Power Batteries School of Chemistry and Materials Science Nanjing Normal University Nanjing 210023 China

**Keywords:** capto‐dative effect, carbosulfenylation, photocatalysis, radicals, sulfur‐containing amino acids

## Abstract

Sulfur‐containing amino acids and peptides play critical roles in organisms. Thiol‐ene reactions between the thiol residues of *L*‐cysteine and the alkenyl fragments in the designed coupling partners serve as primary tools for constructing C─S bonds in the synthesis of unnatural sulfur‐containing amino acid derivatives. These reactions are favored due to the preference for hydrogen transfer from thiol to β‐sulfanyl carbon radical intermediates. In this paper, the study proposes utilizing carbon‐centered radicals stabilized by the capto‐dative effect, generated under photocatalytic conditions from *N*–aryl glycine derivatives. The aim is to compete with the thiol hydrogen, enabling radical C─C bond formation with β‐sulfanyl carbon radicals. This protocol is robust in the presence of air and water, offers significant potential as a modular and efficient platform for synthesizing sulfur‐containing amino acids and modifying peptides, particularly with abundant disulfides and styrenes.

## Graphical abstract

Visible light‐induced carbosulfenylation of styrenes with *N*‐aryl glycinates and disulfides is achieved. Using *N*‐aryl glycinate substrates as precursors of the capto‐dative effect‐stabilized carbon‐centered radicals, the protocol shows insensitivity to air and water and demonstrate great potential as a modular and straightforward platform for the synthesis of sulfur‐containing amino acids and peptide modification.

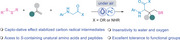

## 1 Introduction

**Scheme 1:**
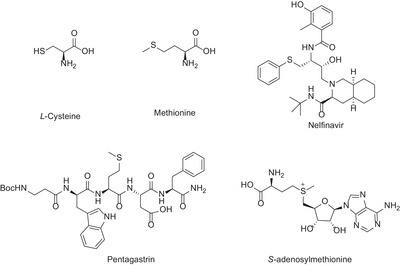
Selected sulfur‐containing amino acids and bioactive molecules.

**Scheme 2:**
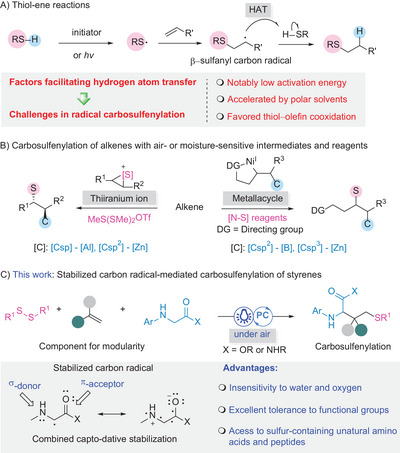
Overview of state‐of‐the‐art. A) Hydrogen atom transfer (HAT) in thiol‐ene reactions and challenges in radical‐type C─C bond formation; B) Carbosulfenylation of alkenes; C) Stabilized carbon radical‐mediated carbosulfenylation of styrenes for modular synthesis of sulfur‐containing amino acid and peptide derivatives.

Sulfur‐containing amino acids and their derivatives play crucial roles in biological processes. For example, *S*‐adenosylmethionine serves as the methyl source for nearly all mammalian transmethylases, and most eukaryotic proteins originate from methionine (**Scheme** **1**).^[^ ^1^ ^]^ However, the bioprocesses involved in the formation of C─S bond during the synthesis of sulfur‐containing molecules in living organisms remain unexplored. The laboratory synthesis of sulfur‐containing unnatural amino acids (UAAs) and peptides heavily relies on the utilization of thiol residues from naturally occurring *L*‐cysteine and its derivatives.^[^ ^2^ ^]^ Among the reported methods, thiol‐ene reactions, a well‐known type of “click reactions”, are commonly used for constructing C─S bonds. Thiol‐ene reactions have found extensive applications in synthetic methodologies, biofunctionalization, and polymer science, as reported.^[^ ^3^ ^]^ The mechanism behind these reactions has been extensively studied and is well established. It involves the addition of thiyl radicals to C─C double bonds, generated from thiols in the presence of light or radical initiators, followed by hydrogen atom transfer (HAT) from another thiol molecule to the carbon radical. (**Scheme** **2**A).^[^ ^4^ ^]^ Despite the potential of difunctionalization of alkenes to construct molecular complexity in a single operation, which has attracted wide interest,^[^ ^5^ ^]^ challenges persist in synthesizing sulfur‐containing UAAs through the incorporation of carbosulfenylation of alkenes with thiols under mild and atmospheric conditions: 1) Thiols serve as effective hydrogen donors with bond dissociation energy of ≈87 and 80 kcal mol^−1^ for RS–H and ArS–H, respectively. The transfer of hydrogen to the β‐sulfanyl carbon‐centered radical intermediates from the thiols is favored due to the particularly low activation energy of the thermoneutral reaction (E_e0_).^[^ ^6^ ^]^ In fact, the facilitated hydrogen transfer from thiols to carbon radicals is the predominant pathway for repairing damage to nucleic acids and proteins caused by other free radicals or toxic species in biological systems.^[^ ^7^ ^]^ 2) The synthesis and modification of amino acids and peptide derivatives typically require polar solvents as the reaction medium due to their limited solubility in non‐polar solvent. Hydrogen transfer from thiols to carbon radicals can be significantly accelerated by utilizing polar solvents because they stabilize the polar transition state.^[^ ^8^ ^]^ 3) When the reaction system is exposed to atmospheric oxygen, thiol‐olefin cooxidation (TOCO) becomes the primary reaction pathway.^[^ ^9^ ^]^ As a result, achieving mild and general carbosulfenylation of alkenes through the addition of thiyl radicals followed by radical‐type C─C cross‐coupling remains challenging (for a highly substrate depended photo‐induced carbosulfenylation of activated styrenes (arylidenemalononitrile) via radical‐polar cross‐over process, see ref. [10])

From a broader perspective, investigations into the intermolecular carbosulfenylation of alkenes pose significant challenges, whether through the thiyl radical intermediates or two‐electron chemistry (Scheme 2B). Trost et al. pioneered carbosulfenylation by the electrophilic activation of alkenes with a sulfenylating agent, followed by the addition of alkynylalaneates, prepared through reactions involving alkynyl lithium reagents and triethylaluminum.^[^ ^11^ ^]^ We recently achieved the arylation of thiiranium ion intermediates, leveraging the covalent nature of organozinc reagents.^[^ ^12^ ^]^ Zhao et al. reported the enantioselective sulfenoarylation of *N*‐allyl sulfonamides by reacting thiiranium ions with electron‐rich phenols. This reaction was conducted in the presence of an indene‐based chiral selenide catalyst and a Tf_2_NH additive (see ref. [13], for intramolecular carbosulfenylation of alkenes intermediated by thirranium ions).^[^ ^14^ ^]^ Engle et al.^[^ ^15^ ^]^ and Wang et al.^[^ ^16^ ^]^ independently established creative sulfenoalkylation/sulfenoarylations of alkenes via metallacycle intermediates by pre‐installing directing groups in alkene substrates and using organometallic agents (dialkylzincs and aryl boronic acids) as carbon sources. These reactions involve air‐ or moisture‐sensitive thiiranium ions or metal complexes and primarily utilize organometallic reagents as carbon nucleophilic partners, thus limiting the reaction conditions. Inspired by the innovative studies on photocatalysis‐enabled thiol‐ene reactions,^[^ ^17^ ^]^ Hao et al. recently developed an elegant carbosulfenylation of 1,3‐butadiene through synergistic catalysis with iridium and titanium.^[^ ^18^ ^]^ In this process, the formation of stabilized allyl radical intermediates was employed to inhibit the HAT process from thiol,^[^ ^19^ ^]^ thereby enabling subsequent C─C bond coupling, which, turned out to be the limitation of the reaction with only 1,3‐butadiene substrate. A more general carbosulfenylation protocol, allowing the modular incorporation of abundant sulfur compounds into amino acid and peptide derivatives under mild atmospheric conditions, is highly desirable.

Photocatalyzed C(sp^3^)–H alkylations of glycinate derivatives have emerged as effective methods for preparing UAAs through various radical transformations, facilitating the modification of naturally occurring amino acid derivatives.^[^ ^20^ ^]^ We realized that integrating thiyl radicals and carbon radical intermediates, derived from disulfides and glycine derivatives, with styrenes under photocatalyzed conditions (Scheme 2C) could overcome the above‐mentioned challenges and provide the following advantages for carbosulfenylation: 1) A significantly large rate constant (≈1 × 10^4^ times) for the inverse reaction of HAT from thiols to benzylic carbon radicals would effectively inhibit the thiol‐ene side reaction.^[^ ^6^, ^21^ ^]^ 2) The low reactivity of disulfides with the β‐sulfanyl carbon‐centered radical intermediates would prevent the formation of disulfenylation byproducts.^[^ ^22^ ^]^ 3) The capto‐dative stabilization (or mero‐stabilization) effect from two mutual substituents (σ‐donor and π‐acceptor) in the radical intermediates of glycine derivatives, combined with the influence of high dielectric constant solvents– known to significantly enhance the capto‐dative effect^[^ ^23^ ^]^ and favored for peptide reaction– provides the opportune stability and reactivity of the glycinate‐derived radical intermediates^[^ ^24a^ ^]^ for radical‐type C─C bond formation. 4) The suitable reactivity of β‐sulfanyl carbon radicals in the presence of stabilized glycinate carbon radicals would circumvent the influences of atmospheric oxygen (TOCO reaction) and water additives, enabling the reaction to be conducted with protonic functionalities in a range of polar solvents without requiring an inert atmosphere. This would enable the modular synthesis of a variety of sulfur‐containing UAAs and peptide derivatives. Herein, we present our studies on the photocatalyzed carbosulfenylation of styrenes with disulfides and glycine derivatives under ambient conditions, facilitated by radical‐type C─C bond formation enabled by the capto‐dative stabilization effect.

## 2 Results and Discussion

We initially investigated the catalytic system with *N*‐phenylglycine ethyl ester **1**, 1,1‐diphenylethylene **2** and diphenyldisulfide **3** in acetonitrile (MeCN) as a model reaction. The system was irradiated with a 15 W blue light (462 nm) using light emitting diode (LED) under an air atmosphere (**Table** **1**). Upon employing Ir(ppy)_3_ as photocatalyst, sulfanyl glycine derivative **4** was isolated in a 61% yield (entry 1). Encouraged by these results, alternative photocatalysts were screened. Both [Ir(ppy)_2_(dtbbpy)]PF_6_ (entry 2) and (Ir[dF(CF_3_)ppy]_2_(dtbbpy))PF_6_ (entry 3) effectively promoted the reaction, yielding **4** in 69% and 68% isolated yields, respectively. Other photocatalysts such as ruthenium‐based complexes and organic dyes, proved much less effective (entry 4 and Table S1, Supporting Information). Throughout the screening process, hydrosulfenylation of **2** was detected as a minor reaction. We hypothesized that the in situ generated thiophenol was responsible for this due to its hydrogen content. Therefore, we attempted to use a base as an additive for the deprotonation process;^[^ ^19a^ ^]^ however, this did not lead to an increase in the yield (entries 5 and 6). Substituting the disulfide reagent with *N*‐(phenylthio)phthalimide, a typical [N‐S] agent, resulted in a low yield (entry 7). Additionally, control experiments illustrated that the reaction proceeded smoothly under nitrogen (entry 8), confirming the essential role of both light and the photocatalyst in the production of the anticipated product (Table S8, Supporting Information). The regioselectivity of the carbosulfenylation of styrene was confirmed through single‐crystal X‐ray crystallographic analysis of **4** (Figure S20, Supporting Information). As the reaction proceeded efficiently in air, we investigated its compatibility with water. Using MeCN and water (4/1, v/v) as cosolvents resulted in a satisfactory yield of the sulfanyl derivative of glycinate (entry 9), suggesting the potential of this protocol for applications under biologically compatible conditions and transformations.

**Table 1:** Optimization of reaction conditions.

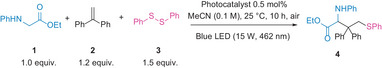			
Entry^a^ ^)^	Photocatalyst	Variation to standard conditions	Yield [%]^b^ ^)^
1	Ir(ppy)_3_	/	61
2	[Ir(ppy)_2_(dtbbpy)]PF_6_	/	69
3	(Ir[dF(CF_3_)ppy]_2_(dtbbpy))PF_6_	/	68
4	4CzIPN	/	32
5	[Ir(ppy)_2_(dtbbpy)]PF_6_	Et_3_N (2.0 equiv.)	69
6	[Ir(ppy)_2_(dtbbpy)]PF_6_	K_3_PO_4_ (2.0 equiv.)	68
7	[Ir(ppy)_2_(dtbbpy)]PF_6_	Replacing **3** with *N*‐(phenylthio)phthalimide	45
8	[Ir(ppy)_2_(dtbbpy)]PF_6_	Nitrogen instead of air	68
9	[Ir(ppy)_2_(dtbbpy)]PF_6_	MeCN/H_2_O = 4/1 as solvents	67

^a)^ Standard conditions: **1** (0.3 mmol, 1.0 equiv.), **2** (0.36 mmol, 1.2 equiv.), **3** (0.45 mmol, 1.5 equiv.), photocatalyst (0.0015 mmol, 0.5 mol%), MeCN (3 mL), Irradiation with a 15 W blue LED (462 nm) under air, 25 °C, 10 h;

^b)^ Isolated yields are given. Ir(ppy)_3_ = tris[2‐phenylpyridinato‐C^2^,*N*]iridium(III); [Ir(dtbbpy)(ppy)_2_]PF_6_ = [4,4′‐*bis*(1,1‐dimethylethyl)‐2,2′‐bipyridine‐*N1,N1′*]*bis*[2‐(2‐pyridinyl‐*N*)phenyl‐C]iridium(III) hexafluorophosphate; (Ir[dF(CF_3_)ppy]_2_(dtbpy))PF_6_ = [4,4′‐*bis*(1,1‐dimethylethyl) ‐2,2′‐bipyridine‐*N1,N1′*]*bis*[3,5‐difluoro‐2‐[5‐(trifluoromethyl)‐2‐pyridinyl‐*N*]phenyl‐C]Iridium(III) hexafluorophosphate; 4CzIPN = 2,4,5,6‐tetra(9*H*‐carbazol‐9‐yl)isophthalonitrile; Et_3_N = triethylamine; MeCN = acetonitrile.

**Scheme 3:**
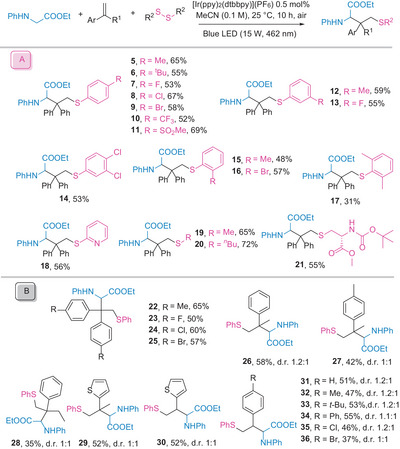
Scope of disulfides and styrenes. Reaction conditions: **1** (0.3 mmol, 1.0 equiv.), styrene (0.36 mmol, 1.2 equiv.), disulfide (0.45 mmol, 1.5 equiv.), [Ir(ppy)_2_(dtbbpy)](PF_6_) (0.0015 mmol, 0.5 mol%), MeCN (3 mL), irradiation with a 15 W blue LED (462 nm) under air, 25 °C, for 10 h. Isolated yields provided, with diastereomeric ratios (d.r.) determined by ^1^H NMR.

With the optimized conditions established, we first explored the substrate scope concerning the disulfides. As shown in **Scheme** **3**A, both alkyl and halide‐derived aryl sulfenyl functionalities could be introduced into the glycinate using 1,1‐diphenylethylene as the linker precursor (**5**–**9**). Despite the electrophilic nature of the thiyl radicals, electron‐withdrawing groups such as trifluoromethyl and sulfonyl were successfully delivered with diaryl disulfides (**10** and **11**). The presence of substituents at the *ortho* and *meta* positions of the aryl rings of the disulfides had little impact on the reaction efficiencies (**12**–**17**). Dipyridyl disulfide proved to be a suitable substrate for the carbosulfenylation (**18**). Furthermore, dialkyl disulfides served as successful sulfenylating agents (**19** and **20**). These results inspired us to test the cystine derivative as a substrate, which transformed smoothly (**21**). Subsequently, the scope of styrenes was investigated (Scheme 3B). The substituents on 1,1‐diarylethylene were varied, resulting in carbosulfenylation products in moderate‐to‐good yields (**22**–**25**). This sequence proved applicable to 1‐Alkylstyrenes and heteroarene‐derived styrenes (**26**–**30**). A range of *para*‐substituted styrenes with diverse functionalities were subjected to standard conditions, yielding products incorporating glycinates and disulfides smoothly (**31**–**36**). Alkyl alkenes typically undergo thiol‐ene reactions primarily due to the fast and nearly irreversible hydrogen transfer from thiols to unstabilized alkyl radicals (rate constants between 6 × 10^6^ and 2 × 10^7^ m ^−1^ s^−1^ with alkyl thiols).^[^ ^8^ ^]^

**Scheme 4:**
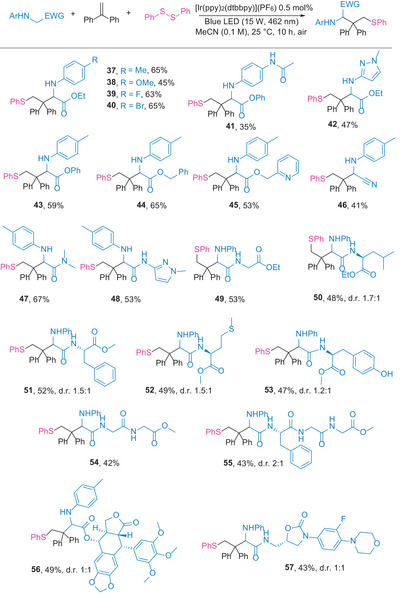
Scope of glycine derivatives. Reaction conditions: glycine derivative (0.3 mmol, 1.0 equiv.), **2** (0.36 mmol, 1.2 equiv.), **3** (0.45 mmol, 1.5 equiv.), [Ir(ppy)_2_(dtbbpy)](PF_6_) (0.0015 mmol, 0.5 mol.%), MeCN (3 mL), irradiation with a 15 W blue LED (462 nm) under air, 25 °C, for 10 h. Isolated yields provided, with diastereomeric ratios determined by ^1^H NMR or ^19^F NMR (**57**).

The broad scope of glycine derivatives and the tolerance of functional group in the modular synthesis of sulfur‐containing amino acid and peptide derivatives were explored, as shown in **Scheme** **4**. The influence of the electron density of the *N*‐aryl moiety on the protocol's outcome was tested (**37**–**41**), revealing that electron‐rich phenyl rings led to slightly low yields (**38** and **41**). The heteroaryl functionality of glycinate was successfully introduced using this procedure (**42**). Both phenol‐and alkyl alcohol‐derived glycinates were compatible (**43**–**45**). Additionally, *N*‐arylaminoacetonitrile could be utilized in this reaction (**46**). The satisfactory performance of amide derivatives of glycine (**47** and **48**), along with their excellent functional group tolerance, prompted us to explore the selective sulfenylative alkylation of various peptides. We prepared a series of dipeptides from *N*‐aryl glycine with leucine, phenylalanine, methionine, and tyrosine, subjecting them to standard conditions. Sulfenylative dipeptides were successfully produced in moderate yields (**49**–**53**). Additionally, tripeptides and tetrapeptides underwent carbosulfenylation smoothly (**54** and **55**). Drug molecules, including podophyllotoxin–an antineoplastic glucoside and antitumor agent–and linezolid–an antibacterial, were coupled with *N*‐phenyl glycine and assessed under standard conditions, leading to the isolation of the corresponding products in moderate yields (**56** and **57**).

**Scheme 5:**
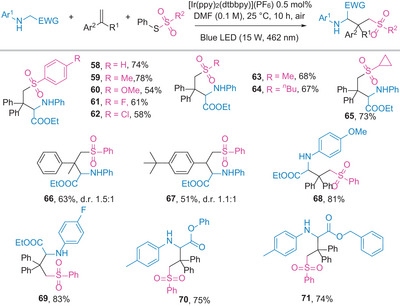
Scope of carbosulfonylation of styrenes. Reaction conditions: glycinate (0.3 mmol, 1.0 equiv.), styrene (0.36 mmol, 1.2 equiv.), *S*‐phenyl sulfonothioate (0.45 mmol, 1.5 equiv.), [Ir(ppy)_2_(dtbbpy)](PF_6_) (0.0015 mmol, 0.5 mol.%), DMF (3 mL), irradiation with a 15 W blue LED (462 nm) under air, 25 °C, for 10 h. Isolated yields provided, with diastereomeric ratios determined by ^1^H NMR.

Encouraged by the method's generality and its resistance to oxygen and water, we extended the photochemical protocol to the carbosulfonylation^[^ ^25^ ^]^ of styrenes, exploring the scope of glycinates and thiosulfonates (R^1^SO_2_SR^2^).^[^ ^26^ ^]^ By slightly modifying the reaction conditions and replacing MeCN with DMF (*N,N*‐dimethylformamide), we achieved the modular synthesis of a range of sulfonyl glycinate derivatives, as shown in **Scheme** **5**. Aryl sulfonyl functionalities, whether bearing electron‐pushing or electron‐withdrawing, substituents were well tolerated (**58**–**62**). *S*‐phenyl alkanethiosulfonates with both acyclic (methyl and butyl) and cyclic (cyclopropyl) carbon chains were successfully transferred (**63**–**65**). Building on the success of the carbosulfenylation, this analogous reaction proved compatible with various styrenes (**66**–**68**) and glycinate derivatives (**69**–**71**), yielding sulfonylated products in good to excellent yields.

**Scheme 6:**
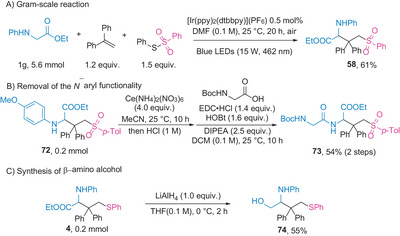
Synthetic applications of carbosulfenylation of styrenes. EDC = 1‐ethyl‐3‐(3‐dimethylaminopropyl)carbodiimide; HOBt = 1‐hydroxybenzotriazole; DIPEA = *N,N*‐diisopropylethylamine; DCM = dichloromethane; THF = tetrahydrofuran.

To demonstrate the practicality of this protocol in synthesizing sulfur‐containing UAAs, a gram‐scale reaction was conducted, yielding product **58** in 61% yield (**Scheme** **6**A). The *N*‐PMP (4‐methoxyphenyl) group was easily removed to obtain the amino ester intermediate, which was then amidated with Boc–Gly (*N*‐(*tert*‐butoxycarbonyl)glycine), resulting in an overall yield of 54% (Scheme 6B). Reduction of the ester group with lithium aluminum hydride (LiAlH_4_) afforded the β‐amino alcohol **74** (Scheme 6C). In addition, the broad scope of glycine derivatives, peptides, disulfides, and styrenes prompted us to test the compatibility of the carbosulfenylation reaction with biomolecules under ambient conditions. The reactions conducted in MeCN:H_2_O (4:1, v/v) using various biomolecules as additives, including nucleobase, protein, amino acid, saccharide, and biotin showed little impact on the outcomes of the reactions (**Table** **2**), further indicating the potential utility of this protocol for biological applications.

**Table 2:** Compatibility with biomolecules.

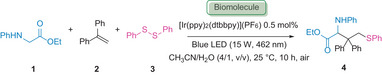		
Entry^a^ ^)^	Biomolecule additive	Yield of 4 [%]^b^ ^)^
1	Thymine (1 equiv.)	61
2	Bovine serum albumin (10 mg mL^−1^)	53
3	L‐Proline (1 equiv.)	57
4	Trypsin (10 mg mL^−1^)	55
5	Adenine (1 equiv.)	57
6	Casein (10 mg mL^−1^)	57
7	D‐ (+)‐Glucose (1 equiv.)	60
8	ATP (1 equiv.)	58

^a)^ Reaction conditions: **1** (0.3 mmol, 1.0 equiv.), **2** (0.36 mmol, 1.2 equiv.), **3** (0.45 mmol, 1.5 equiv.), [Ir(ppy)_2_(dtbbpy)](PF_6_) (0.0015 mmol, 0.5 mol%), biomolecules (0.3 mmol, 1.0 equiv.), MeCN/H_2_O (3 mL, 4/1, v/v), Irradiation with a 15 W blue LED (462 nm) under air, 25 °C, for 10 h;

^b)^ Isolated yields are given.

**Scheme 7:**
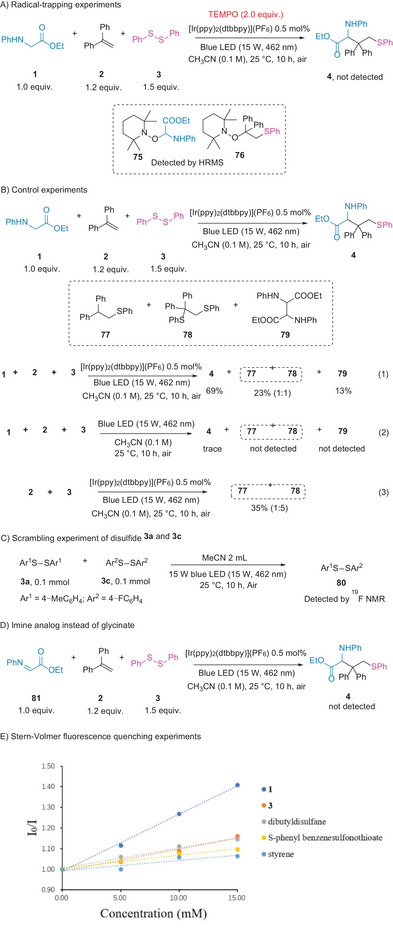
Mechanistic investigations. ^19^F NMR = Fluorine nuclear magnetic resonance.

Several control experiments were conducted to elucidate the carbosulfenylation mechanism (**Scheme** **7**). The reaction was completely inhibited by the addition of an excess of the radical inhibitor 2,2,6,6‐tetramethyl‐1‐peperidyloxy (TEMPO) and afforded the high‐resolution mass spectrometry (HRMS)‐detectable species **75** and **76** (Scheme 7A), which were formed through the coupling of carbon radical intermediates with TEMPO. Under the optimized conditions of the model reaction, the homocoupling product of glycinate (**79**) was isolated as a byproduct in 13% yield (Scheme 7B, Equation (1)). These results indicate the involvement of both the carbon‐centered radical of glycinate and the β‐sulfanyl carbon radical in this process. Additionally, a mixture of sulfide products **77** and **78** (1:1 ratio, as determined by proton nuclear magnetic resonance (^1^H NMR)) was obtained. Control experiments were conducted with and without glycinate (Scheme 7B, Equations (2) and (3)), indicating that a photocatalyst was necessary for the generation of **77** and **78**. These findings, along with the requirement for a photocatalyst under visible light for the formation of the carbosulfenylation product, suggest that the generation of substantial amounts of thiyl radicals is essential for the conversion of styrene and glycinate using a photocatalyst.^[^ ^19^, ^27^ ^]^ Disulfides are effective thiyl radical acceptors^[^ ^28^ ^]^; therefore, the exchange of sulfides (**3a** and **3c**, Scheme 7C) was observed under irradiation with blue light without a photocatalyst. This occurs because the homolytic cleavage of disulfides to produce thiyl radicals under light irradiation is possible, albeit inefficient^[^ ^2a^ ^]^ in the addition of styrene. The absence of carbonsulfenylation product **4** when the imine analog **81** was used under standard conditions instead of glycinate **1** ruled out the possibility of sequential single‐electron oxidation of glycinate (Scheme 7D). A series of Stern‐Volmer fluorescence quenching experiments with substrates showed that only glycinate effectively quenched the excited state of the photocatalyst (Scheme 7E), whereas disulfide or thiosulfonate were relatively less likely to interact with it. These findings were consistent with the literature reports indicating that the standard reduction potentials of disulfides (e.g., 1,2‐di‐*p*‐tolyldisulfide with E_1/2_^red^ = −2.15 V vs SCE^[^ ^26^ ^]^ and 1,2‐dibutyldisulfide with E_1/2_^red^ = −2.14 V vs SCE^[^ ^29^ ^]^) do not match the value of the photocatalyst ([Ir(ppy)_2_(dtbbpy)](PF_6_) with E_1/2_(PC^•─^/PC) = −1.51 V vs SCE). Light on/off experiments revealed that a continuous light irradiation was essential to achieve a satisfactory yield of the product (Figure S17, Supporting Information).

**Scheme 8:**
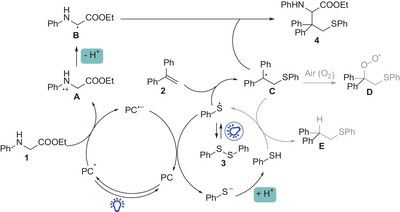
Proposed reaction mechanism.

Based on the aforementioned results and a literature review, a plausible mechanism for the modular synthesis of sulfur‐containing UAAs and peptide modification was proposed (**Scheme** **8**). First, upon irradiation with blue light, the excited state of the photocatalyst [Ir(ppy)_2_(dtbbpy)](PF_6_)* is generated and undergoes a single‐electron transfer event with the glycine derivative. This generates the radical cation intermediate **A** (precursor of the carbon radical **B**) and the radical anion PC^•─^ (glycine ester E_1/2_^ox^ = +0.31 V vs SCE;^[^ ^20k^ ^]^ [Ir(ppy)_2_(dtbbpy)](PF_6_) E_1/2_(PC*/PC^•─^) = +0.66 V vs SCE). The thiyl radical, derived from the irradiation of disulfide, a process that can be accelerated by the presence of photocatalyst, plays dual roles: it oxidizes PC^•─^ to regenerate the photocatalyst (E_1/2_(PhS^•^/PhS^─^) = +0.16 V vs SCE)^[^ ^30^ ^]^ and adds to the C─C double bond of styrene, forming the β‐sulfanyl radical intermediate **C**. The benzyl carbon radical **C** is then trapped by intermediate **B** to yield the desired product. Inhibiting thiol‐ene (**E**) and TOCO (**D**) side reactions hinges on the preformed carbon radical **B**, which is stabilized by two mutual substituents and is an excellent carbon radical acceptor.^[^ ^31^ ^]^ However, a pathway involving single‐electron transfer from PC^•─^ to thiosulfonates in carbosulfonylation cannot be completely ruled out. In some cases, PC^•─^ may directly reduce thiosulfonates (e.g., *S*‐(4‐methylphenyl)‐4‐methylbenzenethiosulfonate: E_p1_ = −1.432 V vs SCE^[^ ^26^ ^]^).

## 3 Conclusion

In summary, a visible‐light‐induced carbosulfenylation of styrenes with *N*‐aryl glycinates and disulfides was developed. This protocol allows the straightforward synthesis of sulfur‐containing amino acid and peptide derivatives by varying the reagent components. The strategic use of *N*‐aryl glycinate substrates, which exhibit capto‐dative effects, stabilizes carbon‐centered radicals and act as coupling partners for C─C bond formation. This feature renders the reaction system insensitive to air and moisture, while maintaining productivity in protic solvents. Notably, this system demonstrates compatibility with biomolecules and biotransformations.^[^ ^32^, ^33^, ^34^, ^35^, ^36^, ^37^, ^38^, ^39^, ^40^, ^41^, ^42^, ^43^, ^44^ ^]^

## Conflict of Interest

The authors declare no conflict of interest.

## Supporting information

Supporting Information

Supporting Information

## Data Availability

The data that support the findings of this study are available in the supplementary material of this article.

## References

[1] J. T. Brosnan , M. E. Brosnan , J. Nutr. 2006, 136, 1636S. 16702333 10.1093/jn/136.6.1636S

[2] a) F. Dénès , M. Pichowicz , G. Povie , P. Renaud , Chem. Rev. 2014, 114, 2587; 24383397 10.1021/cr400441m

[3] C. E. Hoyle , C. N. Bowman , Angew. Chem., Int. Ed. 2010, 49, 1540. 10.1002/anie.200903924 20166107

[4] A. Dondoni , Angew. Chem., Int. Ed. 2008, 47, 8995. 10.1002/anie.200802516 18846513

[5] B. Dong , J. Shen , L.‐G. Xie , Org. Chem. Front. 2023, 10, 1322.

[6] E. T. Denisov , C. Chatgilialoglu , A. Shestakov , T. Denisova , Int. J. Chem. Kinet. 2009, 41, 284.

[7] C. Schoneich , M. Bonifacic , U. Dillinger , K.‐D. Asmus , In Sulfur‐Centered Reaction Intermediates in Chemistry and Biology, Nato ASI Series A, (Eds.: C. Chatgilialoglu , K.‐D. Asmus ) Plenum, New York and London 1990, Vol. 197, pp. 367–376.

[8] C. Tronche , F. N. Martinez , J. H. Horner , M. Newcomb , M. Senn , B. Giese , Tetrahedron Lett. 1996, 37, 5845.

[9] a) O. Ito , H. Watanabe , J. Chem. Soc. Faraday Trans. 1994, 90, 571;

[10] a) A. Wimmer , B. König , Beilstein J. Org. Chem. 2018, 14, 54; 29379578 10.3762/bjoc.14.4 PMC5769090

[11] B. M. Trost , S. J. Martin , J. Am. Chem. Soc. 1984, 106, 4263.

[12] M. Tang , S. Han , S. Huang , S. Huang , L.‐G. Xie , Org. Lett. 2020, 22, 9729. 33253584 10.1021/acs.orglett.0c03810

[13] a) S. E. Denmark , A. Jaunet , J. Am. Chem. Soc. 2013, 135, 6419; 23597174 10.1021/ja401867b PMC3675264

[14] Y. Zhang , Y. Liang , X. Zhao , ACS Catal. 2021, 11, 3755.

[15] a) Z.‐Q. Li , Y. Cao , T. Kang , K. M. Engle , J. Am. Chem. Soc. 2022, 144, 7189; 35436110 10.1021/jacs.1c13252

[16] L. Zhu , X. Meng , L. Xie , Q. Shen , W. Li , L. Zhang , C. Wang , Org. Chem. Front. 2022, 9, 3068.

[17] a) M. Teders , C. Henkel , L. Anhäuser , F. Strieth‐Kalthoff , A. Gómez‐Suárez , R. Kleinmans , A. Kahnt , A. Rentmeister , D. Guldi , F. Glorius , Nat. Chem. 2018, 10, 981; 30082884 10.1038/s41557-018-0102-z

[18] E. Hao , B. Lu , Y. Liu , T. Yang , H. Yan , X. Ding , Y. Jin , L. Shi , Org. Lett. 2023, 25, 5094. 37387472 10.1021/acs.orglett.3c01822

[19] a) J. Kim , B. Kang , S. H. Hong , ACS Catal. 2020, 10, 6013;

[20] a) C. Wang , R. Qi , R. Wang , Z. Xu , Acc. Chem. Res. 2023, 56, 2110; 37467427 10.1021/acs.accounts.3c00260

[21] W. A. Pryor , G. Gojon , D. F. Church , J. Org. Chem. 1978, 43, 793.

[22] A. Ogawa , H. Tanaka , H. Yokoyama , R. Obayashi , K. Yokoyama , N. Sonoda , J. Org. Chem. 1992, 57, 111.

[23] A. R. Katritzky , M. C. Zerner , M. M. Karelson , J. Am. Chem. Soc. 1986, 108, 7213.

[24] a) R. Sustmann , H.‐G. Korth , Adv. Phys. Org. Chem. 1990, 26, 131;

[25] a) A. Garcia‐Dominguez , R. Mondal , C. Nevado , Angew. Chem., Int. Ed. 2019, 58, 12286; 10.1002/anie.201906692 31242342

[26] K. Gadde , P. Mampuys , A. Guidetti , H. Y. V. Ching , W. A. Herrebout , S. Van Doorslaer , K. A. Tehrani , B. U. W. Maes , ACS Catal. 2020, 10, 8765.

[27] O. Ito , M. Matsuda , J. Am. Chem. Soc. 1979, 101, 5732.

[28] M. Bonifačić , K. D. Asmus , J. Phys. Chem. 1984, 88, 6286. 10.1080/09553008414551041 6430835

[29] Q. Zhu , C. Costentin , J. Stubbead , D. G. Nocera , Chem. Sci. 2023, 14, 6876. 37389245 10.1039/d3sc01867a PMC10306091

[30] A. G. Larsen , A. H. Holm , M. Roberson , K. Daasbjerg , J. Am. Chem. Soc. 2001, 123, 1723. 11456773 10.1021/ja003811b

[31] a) H. Tian , W. Xu , Y. Liu , Q. Wang , Org. Lett. 2020, 22, 5005; 32610920 10.1021/acs.orglett.0c01574

[32] J. Luo , J. Zhang , ACS Catal. 2016, 6, 873.

[33] D.‐M. Yan , J.‐R. Chen , W.‐J. Xiao , Angew. Chem., Int. Ed. 2019, 58, 378. 10.1002/anie.201811102 30480861

[34] I. Okamura , S. Park , J. H. Han , S. Notsu , H. Sugiyama , Chem. Lett. 2017, 46, 1597.

[35] R. Wang , J. Wang , Y. Zhang , B. Wang , Y. Xia , F. Xue , W. Jin , C. Liu , Adv. Synth. Catal. 2023, 365, 900.

[36] Y. Zhou , L. Zhao , M. Hu , X.‐H. Duan , L. Liu , Org. Lett. 2023, 25, 5268. 37413688 10.1021/acs.orglett.3c01787

[37] C. Wan , R.‐J. Song , J.‐H. Li , Org. Lett. 2019, 21, 2800. 30969128 10.1021/acs.orglett.9b00771

[38] X. Qiu , X. Yang , Y. Zhang , S. Song , N. Jiao , Org. Chem. Front. 2019, 6, 2220.

[39] G. Hynd , N. C. Ray , H. Finch , D. Middlemiss , M. C. Cramp , P. M. Blaney , K. Williams , Y. Griffon , T. K. Harrison , P. Crackett , CRTH2 Antagonists, (Argenta Discovery AG), WO 2008/074966 A1, 2008.

[40] P. Mampuys , Y. Zhu , S. Sergeyev , E. Ruijter , R. V. A. Orru , S. Van Doorslaer , B. U. W. Maes , Org. Lett. 2016, 18, 2808. 27276236 10.1021/acs.orglett.6b01023

[41] Z. Tan , F. Chen , G. Huang , Y. Li , H. Jiang , W. Zeng , Org. Lett. 2023, 25, 2846. 37058279 10.1021/acs.orglett.3c00813

[42] J. Tong , H. Li , Y. Zhu , P. Liu , P. Sun , Green Chem. 2022, 24, 1995.

[43] L. Cao , S.‐H. Luo , K. Jiang , Z.‐F. Hao , B.‐W. Wang , C.‐M. Pang , Z.‐Y. Wang , Org. Lett. 2018, 20, 4754. 30067375 10.1021/acs.orglett.8b01808

[44] M. Miele , A. Citarella , T. Langer , E. Urban , M. Zehl , W. Holzer , L. Ielo , V. Pace , Org. Lett. 2020, 22, 7629. 32910659 10.1021/acs.orglett.0c02831 PMC8011987

