# Supplementary material for: Stabilized Carbon‐Centered Radical‐Mediated Carbosulfenylation of Styrenes: Modular Synthesis of Sulfur‐Containing Glycine and Peptide Derivatives
Source: Adv Sci (Weinh). 2024 Jun 9;11(29):2402428. doi: 10.1002/advs.202402428 (PMC11304285; doi:10.1002/advs.202402428)

ADVANCED SCIENCE
Open Access

Supporting Information

for *Adv. Sci.*, DOI 10.1002/advs.202402428

Stabilized Carbon-Centered Radical-Mediated Carbosulfenylation of Styrenes: Modular Synthesis of Sulfur-Containing Glycine and Peptide Derivatives

*Zihui Yang, Jia Liu and Lan-Gui Xie**

Stabilized Carbon-Centered Radical-Mediated Carbosulfenylation of Styrenes: Modular Synthesis of Sulfur-Containing Glycine and Peptide Derivatives

Zihui Yang,[+] Jia Liu,[+] and Lan-Gui Xie*

National and Local Joint Engineering Research Center of Biomedical Functional Materials, Jiangsu Key Laboratory of New Power Batteries, School of Chemistry and Materials Science, Nanjing Normal University, Nanjing 210023, China

E-mail: xielg@njnu.edu.cn

[+] These authors contributed equally to this work.

1 Contents

1 Contents 1

2 General information 3

3 Reaction optimization 4

3.1 Photochemical reaction set-up 4

3.2 General procedure for screening reactions 4

3.3 Reaction optimization 5

3.3.1 Screening of photocatalysts 5

3.3.2 Screening of solvents 5

3.3.3 Screening of inner gas 6

3.3.4 Screening of the equivalents of the reactants 6

3.3.5 Screening of concentrations 7

3.3.6 Screening of reaction times 7

3.3.7 Screening of other conditions 8

3.3.8 Optimization of carbosulfonylation 8

4. Experimental procedures and characterization of compound 8

4.1 Synthesis of starting materials 9

4.1.1 Preparation of *N*-aryl glycine derivatives 9

4.1.2 Styrene substrates 17

4.1.3 Disulfide substrates 18

4.1.4 Thiosulfonate substrates 19

4.2 Synthesis of sulfanyl glycine derivative products 19

4.2.1 General procedure for the preparation of sulfanyl glycine derivative products 19

4.2.2 General procedure for the preparation of sulfonyl glycine derivative products 19

4.2.3 Characterization data of sulfanyl glycine derivative products 20

5. Studies on Synthetic Applications 61

5.1 Gram-scale synthesis 61

5.2 Removal of PMP group 62

5.2.1 The preparation of sulfonyl glycine derivative 72 62

5.2.2 Removal of PMP group experiments 62

5.3 Synthesis of β-amino alcohol 64
5.4 Compatibility with biomolecules 64
5.4.1 General procedure for the test of compatibility with biomolecules 64
5.4.2 Results of the test of compatibility with biomolecules 65
6. Mechanistic studies 66
6.1 Radical-trapping experiments 66
6.1.1 Radical-trapping experiments of carbosulfanylation 66
6.1.2 Radical-trapping experiments of carbosulfonylation 67
6.2 By-products and absence of photocatalyst 68
6.3 Stern-Volmer fluorescence quenching 71
6.4 Light-dark cycle experiment 74
6.5 Radical crossover experiments 75
6.5.1 Scrambling experiment between disulfide 3a and 3c 75
6.5.2 Scrambling experiment between thiosulfonates f9 and f10 76
6.6 Imine analogue instead of glycinate experiments 77
6.7 UV light experiment 77
7. X-ray crystallographic data for 4 78
8. References 80
9. ^{1}H NMR, ^{13}C NMR and ^{19}F NMR Spectra 82

2 General information

All reactions involving air or moisture sensitive reagents were carried out in flame dried glass ware under nitrogen atmosphere using standard Schlenk techniques. Solvents were either freshly distilled or obtained in extra-dry grade from commercial sources, and stored over molecular sieves (3 Å). Diethyl ether (Et_2O) was distilled over sodium/benzophenone and stored over activated molecular sieves (3 Å). Dichloromethane (CH_2Cl_2) was refluxed over CaH_2 and used as freshly distilled. The material of vessels for the reactions is borosilicate glass. Blue LED lamp (462 nm) was purchased from Zhongshan Langniu Lighting Technology Co., Ltd. (E27, 15 W). Column chromatography was performed with silica gel (300-400 mesh). Merck silica gel 60 F254 plates were used for thin—layer chromatography (TLC). The NMR spectra were recorded on a Bruker Avance 400 spectrometer at 400 MHz (^1H), 101 MHz (^{13}C) and 376 MHz (^{19}F) in $CDCl_3$ with tetramethylsilane as the internal standard. Chemical shifts (δ) were reported in parts per million (ppm). Splitting patterns were designated as s, singlet; d, doublet; t; dd, doublet of doublets; m, multiplet. High-resolution mass spectra were obtained with an AB Triple 5600 mass spectrometer by ESI on a TOF mass analyzer.

3 Reaction optimization

3.1 Photochemical reaction set-up

All photochemical reactions were performed by irradiation with a blue LED (15 W, 462 nm). The reaction vials were placed at approximately 1.0 – 2.5 cm distance from the LEDs with a cooling fan to keep the reaction temperature at 25 °C (Figure S1).

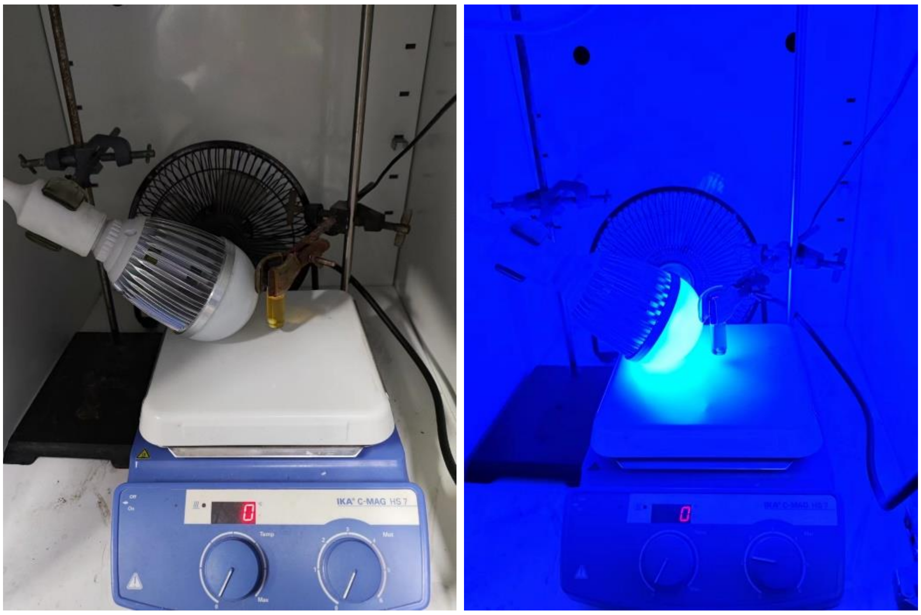

Figure S1 Pictures of the home-made photochemical (LED) reactor

3.2 General procedure for screening reactions

The reactions were carried out in 8 mL transparent vials. Each vial was equipped with a magnetic stir bar and charged with the photocatalyst (0.0015 mmol, 0.5 mol%), *N*-phenylglycine ethyl ester **1** (0.3 mmol, 53.7 mg), 1,1-diphenylethylene **2** (0.36 mmol, 64.8 mg), diphenyl disulfide **3** (0.45 mmol, 98.1 mg) (or *S*-phenyl benzenesulfonothioate **f1** (0.45 mmol, 112.5 mg)) and solvent (3 mL). The reaction vial was closed under the indicated atmosphere, placed in a home-made photochemical (LED) reactor (Figure S1), magnetically stirred and irradiated with a 15 W blue LED (462 nm) at 25 °C (with a cooling fan to keep the reaction temperature) for 10 h. After quenching the reaction with water (5 mL), the resulting mixture was separated and extracted with EtOAc (10 mL x 3). The combined organic layers were dried over Na_2SO_4, concentrated under reduced pressure, and purified through a silica gel column.

3.3 Reaction optimization

3.3.1 Screening of photocatalysts

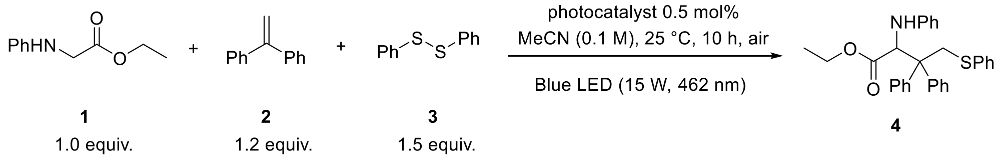

Table S1 Screening of photocatalysts.

Entry	Photocatalyst	Yield [a] (%)
1	$Ir(ppy)_3$	61
2	$[Ir(ppy)_2(dtbbpy)](PF_6)$	69
3	$Ir[dF(CF_3)ppy]_2(dtbbpy)PF_6$	68
4	$[Ru(bpy)_3](PF_6)_2$	N.D.
6	$[Ru(bpz)_3](PF_6)_2$	N.D.
7	4CzIPN	32
8	Esion Y	N.D.
9	Rose bengal	N.D.

Reaction conditions: **1** (0.3 mmol, 1.0 equiv.), **2** (0.36 mmol, 1.2 equiv.), **3** (0.45 mmol, 1.5 equiv.), photocatalyst (0.0015 mmol, 0.5 mol%), MeCN (3 mL), Irradiation with a 15 W blue LED (462 nm) under air, 25 °C, 10 h. [a] Isolated yields are given. N.D. not detected.

Table S2 Electrochemical data for the employed photocatalysts.

Photocatalyst	$E_{1/2}(PC^*/PC^{\bullet-})$ V vs. SCE	$E_{1/2}(PC^*/PC^{\bullet+})$ V vs. SCE	E_T kcal/mol	Reference
*fa*c-$Ir(ppy)_3$	+0.31	-1.73	57.8	[1]
$[Ir(ppy)_2(dtbbpy)](PF_6)$	+0.66	-0.96	49.2	[1]
$Ir[dF(CF_3)ppy]_2(dtbbpy)PF_6$	+1.21	-0.89	60.8	[1]
$[Ru(bpy)_3](PF_6)_2$	+0.77	-0.81	46.5	[1]
$[Ru(bpz)_3](PF_6)_2$	+1.45	-0.26	48.4	[1]
4CzIPN	+1.35	-1.04	-	[2]
Esion Y	+0.83	-1.11	43.5	[2], [3]
Rose bengal	+0.66	-0.99	-	[2]

3.3.2 Screening of solvents

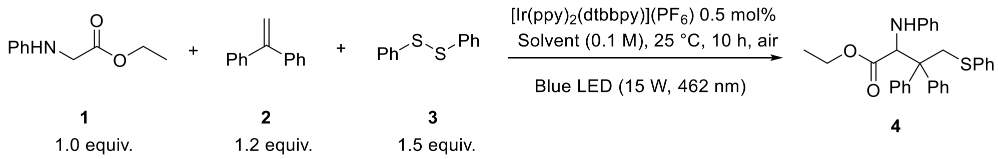

Table S3 Screening of solvents.

Entry	Solvents	Yield [a] (%)
1	MeCN	69
2	DMSO	61
3	THF	30
4	DCM	43
5	DMF	20
6	MeCN /H_2O = 4/1	67
7	MeCN /H_2O = 2/1	61

Reaction conditions: **1** (0.3 mmol, 1.0 equiv.), **2** (0.36 mmol, 1.2 equiv.), **3** (0.45 mmol, 1.5 equiv.), [Ir(ppy)$_2$(dtbbpy)](PF$_6$) (0.0015 mmol, 0.5 mol%), Solvent (3 mL), Irradiation with a 15 W blue LED (462 nm) under air, 25 °C, 10 h. [a] Isolated yields are given.

3.3.3 Screening of inner gas

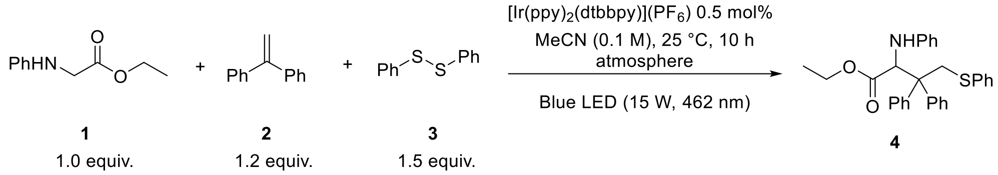

Table S4 Screening of inner gas.

Entry	Atmosphere	Yield [a] (%)
1	N_2	68
2	air	69

Reaction conditions: **1** (0.3 mmol, 1.0 equiv.), **2** (0.36 mmol, 1.2 equiv.), **3** (0.45 mmol, 1.5 equiv.), [Ir(ppy)$_2$(dtbbpy)](PF$_6$) (0.0015 mmol, 0.5 mol%), MeCN (3 mL), Irradiation with a 15 W blue LED (462 nm) under air or N_2, 25 °C, 10 h. [a] Isolated yields are given.

3.3.4 Screening of the equivalents of the reactants

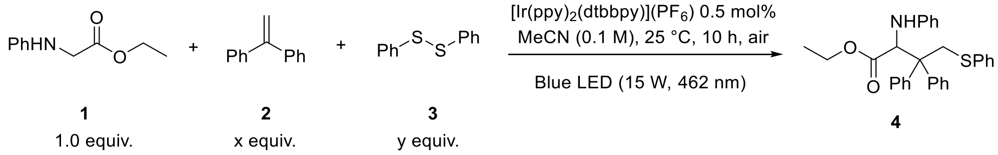

Table S5 Screening of the equivalent of the reactants.

Entry	1,1-diphenylethylene (**2**)	diphenyl disulfide (**3**)	Yield [a] (%)
1	1.2 equiv	1.5 equiv	69

2	1.2 equiv	2.0 equiv	67
3	1.2 equiv	1.0 equiv	31
4	1.5 equiv	1.5 equiv	56
5	1.0 equiv	1.5 equiv	33

Reaction conditions: **1** (0.3 mmol, 1.0 equiv.), **2** (x equiv.), **3** (y equiv.), [Ir(ppy)$_2$(dtbbpy)](PF$_6$) (0.0015 mmol, 0.5 mol%), MeCN (3 mL), Irradiation with a 15 W blue LED (462 nm) under air, 25 °C, 10 h. [a] Isolated yields are given.

3.3.5 Screening of concentrations

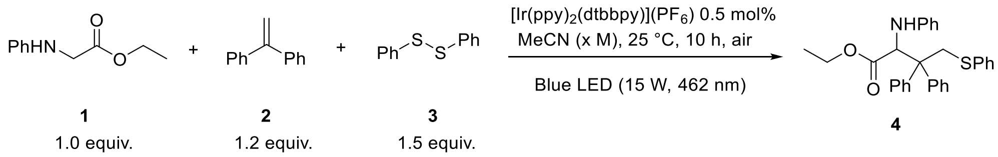

Table S6 Screening of concentrations.

Entry	MeCN (mL)	Yield [a] (%)
1	3	69
2	4	61
3	2	50

Reaction conditions: **1** (0.3 mmol, 1.0 equiv.), **2** (0.36 mmol, 1.2 equiv.), **3** (0.45 mmol, 1.5 equiv.), [Ir(ppy)$_2$(dtbbpy)](PF$_6$) (0.0015 mmol, 0.5 mol%), MeCN (x mL), Irradiation with a 15 W blue LED (462 nm) under air, 25 °C, 10 h. [a] Isolated yields are given.

3.3.6 Screening of reaction times

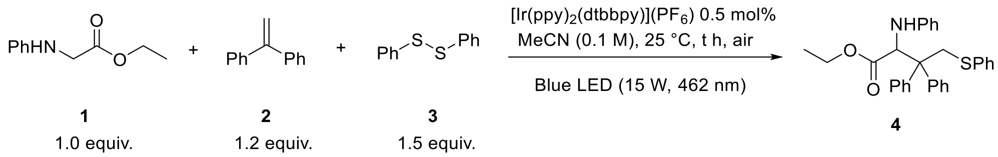

Table S7 Screening of reaction times.

Entry	Times	Yield [a] (%)
1	10	69
2	2	23
3	7	52
4	12	68
5	24	69

Reaction conditions: **1** (0.3 mmol, 1.0 equiv.), **2** (0.36 mmol, 1.2 equiv.), **3** (0.45 mmol, 1.5 equiv.), [Ir(ppy)$_2$(dtbbpy)](PF$_6$) (0.0015 mmol, 0.5 mol%), MeCN (3 mL), Irradiation with a 15 W blue LED (462 nm) under air, 25 °C, t h. [a] Isolated yields are given.

3.3.7 Screening of other conditions

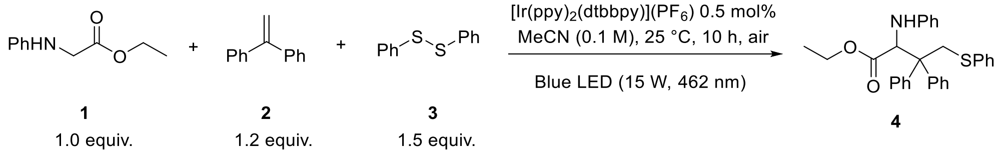

Table S8 Screening of other conditions.

Entry	Variation to standard conditions	Yield [a] (%)
1	Et_3N (2.0 equiv.)	69
2	K_3PO_4 (2.0 equiv.)	68
3	replacing 3 with *N*-(phenylthio)phthalimide	45
4	no light	N.D.
5	no PC	<10
6	no light & no PC	N.D.

Reaction conditions: **1** (0.3 mmol, 1.0 equiv.), **2** (0.36 mmol, 1.2 equiv.), **3** (0.45 mmol, 1.5 equiv.), $[Ir(ppy)_2(dtbbpy)](PF_6)$ (0.0015 mmol, 0.5 mol%), MeCN (3 mL), Irradiation with a 15 W blue LED (462 nm) under air, 25 °C, 10 h. [a] Isolated yields are given. N.D. not detected.

3.3.8 Optimization of carbosulfonylation

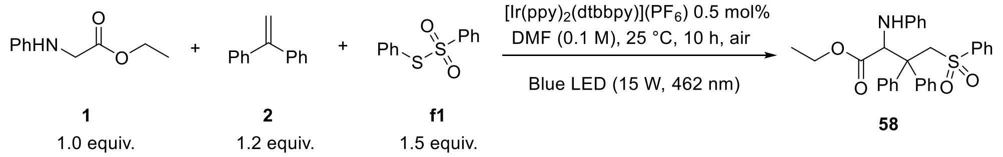

Table S9 Optimization of carbosulfonylation.

Entry	Variation to standard conditions	Yield [a] (%)
1	/	74
2	MeCN as solvent	53
3	$Ir[dF(CF_3)ppy]_2(dtbbpy)PF_6$ as photocatalyst	71
4	*fac*-$[Ir(ppy)_3]$	55
5	$[Ru(bpy)_3](PF_6)_2$	48
6	no light	N.D.
7	no PC	<10
8	no light & no PC	N.D.

Reaction conditions: **1** (0.3 mmol, 1.0 equiv.), **2** (0.36 mmol, 1.2 equiv.), **f1** (0.45 mmol, 1.5 equiv.), $[Ir(ppy)_2(dtbbpy)](PF_6)$ (0.0015 mmol, 0.5 mol%), DMF (3 mL), Irradiation with a 15 W blue LED (462 nm) under air, 25 °C, 10 h. [a] Isolated yields are given. N.D. not detected.

4. Experimental procedures and characterization of

compound

4.1 Synthesis of starting materials

4.1.1 Preparation of *N*-aryl glycine derivatives

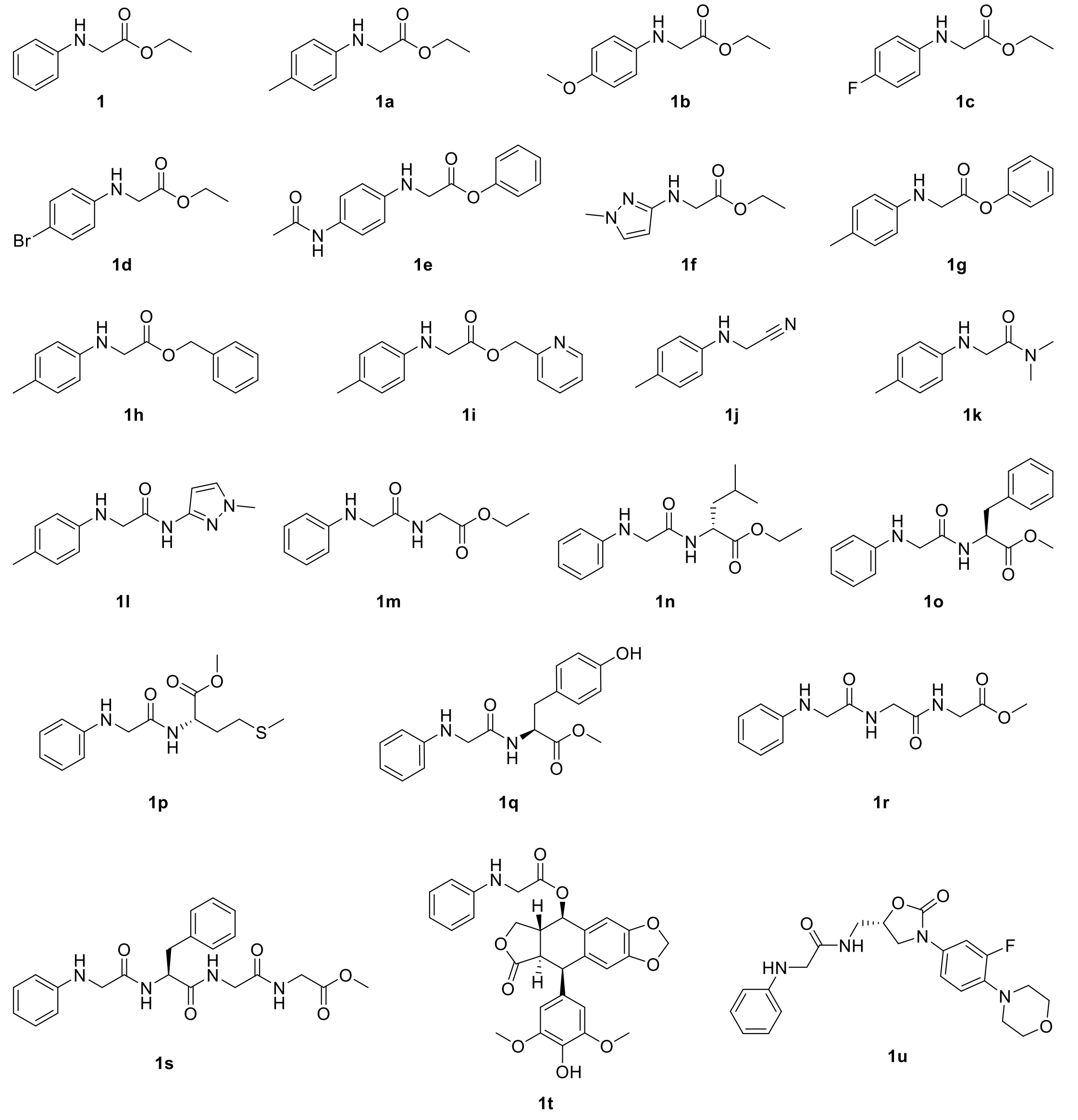

Figure S2 Starting materials of glycine derivatives.

Substrate **1** were purchased from commercial sources.

Substrates **1a**[4], **1b**[4], **1c**[4], **1d**[4], **1g**[4], **1h**[5], **1j**[4], **1k**[6], **1m**[4] and **1o**[4] were prepared according to the literature procedure.

Procedure (A) for the synthesis of substrates 1e, 1f, 1i, and 1l

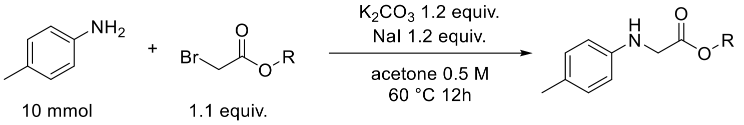

The mixture of amine (10.0 mmol), potassium carbonate (1.7 g, 12.0 mmol) and sodium iodide (1.8 g, 12.0 mmol) were treated with the corresponding bromoacetate-ester (11.0 mmol), in dry acetone at 60 °C for 12 h. After quenching the reaction with water (30 mL), the resulting mixture was separated and extracted with ethyl acetate (EA) (30 mL x 3). The combined organic layers were dried over Na_2SO_4, concentrated under reduced pressure, and purified through a silica gel column (EA and petro ether as the elution).

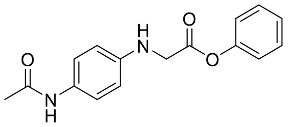

phenyl (4-acetamidophenyl)glycinate (1e) According to the procedure A, *N*-(4-aminophenyl)acetamide (1.5 g, 10.0 mmol) and phenyl 2-bromoacetate (2.4 g, 11.0 mmol) were used. The product was isolated by a silica gel column chromatography using petroleum ether / EtOAc = 1:1 as eluent to give **1e** as a white solid (1.7 g, 62% yield). m.p. 184 – 185 ℃. ^{1}H NMR (400 MHz, DMSO-d_6) δ 9.59 (s, 1H), 7.44 – 7.40 (m, 2H), 7.32 – 7.24 (m, 3H), 7.12 – 7.10 (m, 2H), 6.60 – 6.58 (m, 2H), 5.99 (t, J = 6.4 Hz, 1H), 4.16 (d, J = 6.4 Hz, 2H), 1.97 (s, 3H) ppm. ^{13}C NMR (101 MHz, DMSO-d_6) δ 170.6, 167.4, 150.4, 144.1, 129.6, 129.4, 126.0, 121.7, 120.9, 112.2, 45.2, 23.8 ppm. HRMS (ESI) m/z: [M + H]$^+$ Calcd for $C_{16}H_{17}N_2O_3$ 285.1234; Found 285.1233.

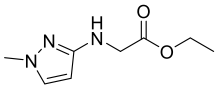

ethyl (1-methyl-1*H*-pyrazol-3-yl)glycinate (1f) According to the procedure A, 1-methyl-1*H*-pyrazol-3-amine (970.6 mg, 10.0 mmol) and ethyl 2-bromoacetate (1.8 g, 11.00 mmol) were used. The product was isolated by a silica gel column chromatography using petroleum ether / EtOAc = 3:1 as eluent to give **1f** as a yellowish

oil (1.2 g, 67% yield). ^{1}H NMR (400 MHz, $CDCl_3$) δ 7.07 (d, *J* = 2.4 Hz, 1H), 5.50 (d, *J* = 2.4 Hz, 1H), 4.18 (q, *J* = 7.2 Hz, 2H), 3.92 (s, 2H), 3.68 (s, 3H), 1.25 (t, *J* = 7.2 Hz, 3H) ppm. ^{13}C NMR (101 MHz, $CDCl_3$) δ 171.6, 155.9, 131.1, 91.0, 60.9, 46.4, 38.4, 14.1 ppm. HRMS (ESI) m/z: $[M + H]^+$ Calcd for $C_8H_{14}N_3O_2$ 184.1081; Found 184.1080.

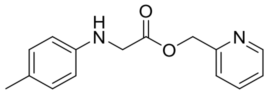

pyridin-2-ylmethyl *p*-tolylglycinate (1i) According to the procedure A, *p*-toluidine (1.1 g, 10.0 mmol) and pyridin-2-ylmethyl 2-bromoacetate (2.5 g, 11.00 mmol) were used. The product was isolated by a silica gel column chromatography using petroleum ether / EtOAc = 5:1 as eluent to give **1i** as a white solid (1.9 g, 75% yield). m.p. 57 – 58 ℃. ^{1}H NMR (400 MHz, $CDCl_3$) δ 8.58 – 8.57 (m, 1H), 7.66 – 7.61 (m, 1H), 7.26 – 7.24 (m, 1H), 7.21 – 7.18 (m, 1H), 6.99 – 6.97 (m, 2H), 6.54 – 6.52 (m, 2H), 5.30 (s, 2H), 3.98 (s, 2H), 2.23 (s, 3H) ppm. ^{13}C NMR (101 MHz, $CDCl_3$) δ 170.9, 154.9, 149.2, 144.5, 136.6, 129.5, 127.1, 122.8, 121.7, 112.9, 67.0, 45.9, 20.2 ppm. HRMS (ESI) m/z: $[M + H]^+$ Calcd for $C_{15}H_{17}N_2O_2$ 257.1285; Found 257.1282.

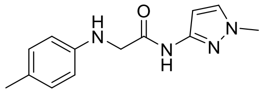

***N*-(1-methyl-1*H*-pyrazol-3-yl)-2-(*p*-tolylamino)acetamide (1l)** According to the procedure A, *p*-toluidine (1.1 g, 10.0 mmol) and 2-bromo-*N*-(1-methyl-1*H*-pyrazol-3-yl)acetamide (2.4 g, 11.00 mmol) were used. The product was isolated by a silica gel column chromatography using petroleum ether / EtOAc = 1:1 as eluent to give **1l** as a yellow solid (1.6 g, 67% yield). m.p. 152 – 153 ℃. ^{1}H NMR (400 MHz, $CDCl_3$) δ 9.05 (s, 1H), 7.24 – 7.23 (m, 1H), 7.01 – 6.99 (m, 2H), 6.72-6.71 (m, 1H), 6.56 – 6.54 (m, 2H), 4.28 – 4.25 (m, 1H), 3.87 – 3.86 (m, 2H), 3.75 (s, 3H), 2.23 (s, 3H) ppm. ^{13}C NMR (101 MHz, $CDCl_3$) δ 168.6, 146.3, 144.6, 130.8, 129.9, 128.7, 113.4, 97.2, 49.5, 38.7, 20.3 ppm. HRMS (ESI) m/z: $[M + H]^+$ Calcd for $C_{13}H_{17}N_4O$ 245.1397; Found 245.1388.

Procedure (B) for the synthesis of dipeptides 1n, 1p, and 1q

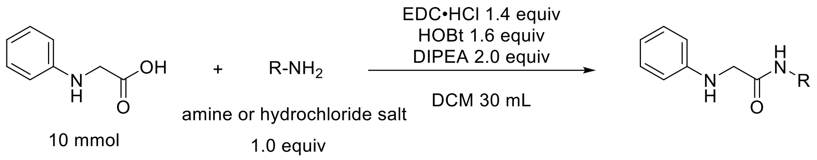

To a 100 mL round-bottom flask, phenylglycine (1.5 g, 10.0 mmol), EDC•HCl (1-ethyl-3-(3-dimethylaminopropyl)carbodiimide hydrochloride, 2.7 g, 14.0 mmol) and HOBt (1-hyd.r.oxybenzotriazole, 2.2 g, 16.00 mmol) were dissolved in DCM (30 mL) under N_2 atmosphere, and the mixture was stirred at 25 °C for 30 min. Then the reaction was cooled in an ice-water bath. The amino ester hydrochloride (10.0 mmol) was added into the flask, followed by the addition of DIPEA (*N,N*-diisopropylethylamine, 2.6 g, 20.00 mmol) under N_2. After 30 min, the reaction mixture was warmed up to 25 °C and stirred overnight. After quenching the reaction with water (30 mL), the resulting mixture was separated and extracted with EtOAc (30 mL x 3). The combined organic layers were dried over Na_2SO_4, concentrated under reduced pressure, and purified through a silica gel column (EtOAc and petro ether as the elution).

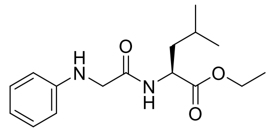

ethyl phenylglycyl-*L*-leucinate (1n) According to the procedure B, phenylglycine (1.5 g, 10.0 mmol) and ethyl *L*-leucinate hydrochloride (2.0 g, 10.0 mmol) were used. The product was isolated by a silica gel column chromatography using petroleum ether / EtOAc = 3:1 as eluent to give **1n** as a white solid (1.5 g, 53% yield). m.p. 46 – 47 ℃. ^{1}H NMR (400 MHz, $CDCl_3$) δ 7.21 – 7.17 (m, 2H), 7.06 (d, *J* = 8.8 Hz, 1H), 6.80 – 6.77 (m, 1H), 6.63 – 6.61 (m, 2H), 4.67 – 4.61 (m, 1H), 4.42 (s, 1H), 4.13 (q, *J* = 7.2 Hz, 2H), 3.84 – 3.75 (m, 2H), 1.64 – 1.45 (m, 3H), 1.22 (t, *J* = 7.2 Hz, 3H), 0.90 – 0.85 (m, 6H) ppm. ^{13}C NMR (101 MHz, $CDCl_3$) δ 172.6, 170.5, 147.1, 129.2, 118.9, 113.2, 61.2, 50.4, 48.7, 41.2, 24.7, 22.7, 21.6, 14.0 ppm. HRMS (ESI) m/z: $[M + H]^+$ Calcd for $C_{16}H_{25}N_2O_3$ 293.1860; Found 293.1855.

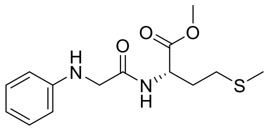

methyl phenylglycyl-*L*-methioninate (1p) According to the procedure B, phenylglycine (1.5 g, 10.0 mmol) and methyl *L*-methioninate hydrochloride (2.0 g, 10.0 mmol) were used. The product was isolated by a silica gel column chromatography using petroleum ether / EtOAc = 3:1 as eluent to give **1p** as a yellow viscous oil (1.6 g, 55% yield). ^{1}H NMR (400 MHz, $CDCl_3$) δ 7.32 (d, *J* = 8.8 Hz, 1H), 7.22 – 7.18 (m, 2H), 6.80 (t, *J* = 7.2 Hz, 1H), 6.62 – 6.61 (m, 2H), 4.78 – 4.73 (m, 1H), 3.86 – 3.77 (m, 2H), 3.70 (s, 3H), 2.38 (t, *J* = 7.6 Hz, 2H), 2.17 – 2.08 (m, 1H), 1.97 (s, 3H), 1.95 – 1.88 (m, 1H) ppm. ^{13}C NMR (101 MHz, $CDCl_3$) δ 172.0, 170.6, 146.9, 129.3, 119.1, 113.2, 52.4, 51.1, 48.6, 31.3, 29.8, 15.3 ppm. HRMS (ESI) m/z: $[M + H]^+$ Calcd for $C_{14}H_{21}N_2O_3S$ 297.1267; Found 297.1264.

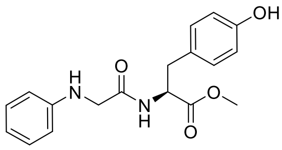

methyl phenylglycyl-*L*-tyrosinate (1q) According to the procedure B, phenylglycine (1.5 g, 10.0 mmol) and methyl *L*-tyrosinate hydrochloride (2.3 g, 10.0 mmol) were used. The product was isolated by a silica gel column chromatography using petroleum ether / EtOAc = 1:1 as eluent to give **1q** as a yellowish viscous oil (1.7 g, 51% yield). ^{1}H NMR (400 MHz, $CDCl_3$) δ 7.94 (br, 1H), 7.28 (d, *J* = 8.4 Hz, 1H), 7.18 (t, *J* = 7.6 Hz, 2H), 6.81 – 6.73 (m, 3H), 6.64 – 6.62 (m, 2H), 6.51 – 6.49 (m, 2H), 4.93 – 4.87 (m, 1H), 3.74 – 3.64 (m, 5H), 3.01 – 2.91 (m, 2H) ppm. ^{13}C NMR (101 MHz, $CDCl_3$) δ 171.9, 171.3, 155.5, 146.6, 130.1, 129.2, 126.3, 119.0, 115.5, 113.1, 52.7, 52.4, 48.2, 36.8 ppm. HRMS (ESI) m/z: $[M + H]^+$ Calcd for $C_{18}H_{21}N_2O_4$ 329.1496; Found 329.1491.

Synthesis of 1r

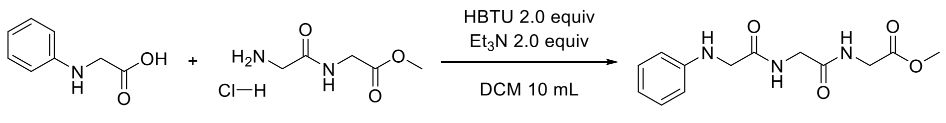

1r

To a 50 mL round-bottom flask, phenylglycine (5.00 mmol, 0.7 g), HBTU (O-benzotriazole-*N,N,N',N'*-tetramethyl-uronium-hexafluorophosphate, 10.0 mmol, 3.8 g), Et_3N (10.0 mmol, 1.0 g), and methyl glycylglycinate hydrochloride (5.00 mmol, 0.9 g) were dissolved in DCM (20 mL) under N_2 atmosphere, and the mixture was stirred at 25 °C for 15 h. After quenching the reaction with water (30 mL), the resulting mixture was separated and extracted with EtOAc (30 mL x 3). The combined organic layers were dried over Na_2SO_4, concentrated under reduced pressure, and purified with a silica gel column (using petroleum ether / EtOAc = 1:1 as eluent) to give **1r** as a white solid (1.0 g, 72% yield). m.p. 137 – 138 ℃.

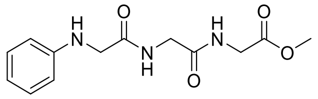

methyl phenylglycylglycylglycinate (1r) According to the procedure, purified with a silica gel column (using petroleum ether / EtOAc = 1:1 as eluent) to give **1r** as a white solid (1.0 g, 72% yield). m.p. 137 – 138 ℃. ^{1}H NMR (400 MHz, $CDCl_3$) δ 7.42 – 7.40 (m, 1H), 7.23 – 7.19 (m, 2H), 6.82 – 6.78 (m, 1H), 6.72 (br 1H), 6.63 – 6.61 (m, 2H), 4.01 – 3.98 (m, 4H), 3.85 (s, 2H), 3.73 (s, 3H) ppm. ^{13}C NMR (101 MHz, $CDCl_3$) δ 171.7, 170.1, 169.0, 146.9, 129.5, 119.2, 113.1, 52.4, 48.5, 42.8, 41.1 ppm. HRMS (ESI) m/z: $[M + H]^+$ Calcd for $C_{13}H_{18}N_3O_4$ 280.1292; Found 280.1285.

Synthesis of 1s

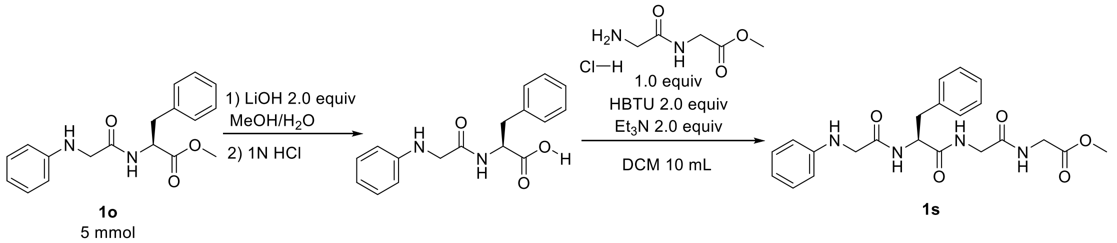

1o
5 mmol
1s

To a solution of compound **1o** (5.00 mmol, 1.6 g, dissolved in 5 mL MeOH) cooled in an ice-water bath was slowly added a solution of LiOH (10.0 mmol, 0.2 g, dissolved

in 5 mL water). The reaction was stirred for 5 h at 25 °C before it was acidified with 1N HCl aqueous solution (to pH = 2). The mixture was extracted with EtOAc (10 mL x 3), washed with brine (20 mL), dried over Na_2SO_4, and concentrated under reduced pressure.

To a 50 mL round-bottom flask, acid, HBTU (10.0 mmol, 3.8 g), Et_3N (10.0 mmol, 1.0 g), and methyl glycylglycinate hydrochloride (5.00 mmol, 0.9 g) were dissolved in DCM (20 mL) under N_2 atmosphere, and the mixture was stirred at 25 °C for 15 h. After quenching the reaction with water (30 mL), the resulting mixture was separated and extracted with EtOAc (30 mL x 3). The combined organic layers were dried over Na_2SO_4, concentrated under reduced pressure, and purified with a silica gel column (using petroleum ether / MeOH = 20:1 as eluent) to give **1s** as a white solid (1.1 g, 52% yield). m.p. 125 – 126 ℃.

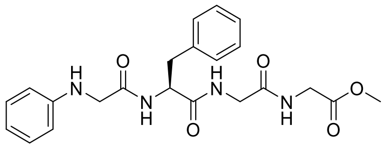

methyl phenylglycyl-*L*-phenylalanylglycylglycinate (1s) According to the procedure, purified with a silica gel column (using petroleum ether / MeOH = 20:1 as eluent) to give **1s** as a white solid (1.1 g, 52% yield). m.p. 125 – 126 ℃. ^{1}H NMR (400 MHz, $CDCl_3$) δ 7.59 (t, J = 6.0 Hz, 1H), 7.47 (d, J = 7.6 Hz, 1H), 7.27 – 7.25 (m, 1H), 7.16 – 7.12 (m, 5H), 7.03 – 7.01 (m, 2H), 6.78 – 6.74 (m, 1H), 6.49 – 6.47 (m, 2H), 4.80 – 4.75 (m, 1H), 4.56 (br, 1H), 3.97 – 3.92 (m, 3H), 3.87 – 3.73 (m, 2H), 3.67 – 3.63 (m, 4H), 3.10 – 2.94 (m, 2H) ppm. ^{13}C NMR (101 MHz, $CDCl_3$) δ 171.8, 171.8, 170.4, 169.5, 147.1, 136.1, 129.3, 129.1, 128.5, 126.9, 118.7, 113.0, 54.3, 52.3, 48.2, 42.8, 41.0, 37.7 ppm. HRMS (ESI) m/z: $[M + H]^+$ Calcd for $C_{22}H_{27}N_4O_5$ 427.1976; Found 427.1975.

Synthesis of glycine derivatives derived from d.r.ug molecules 1t and 1u

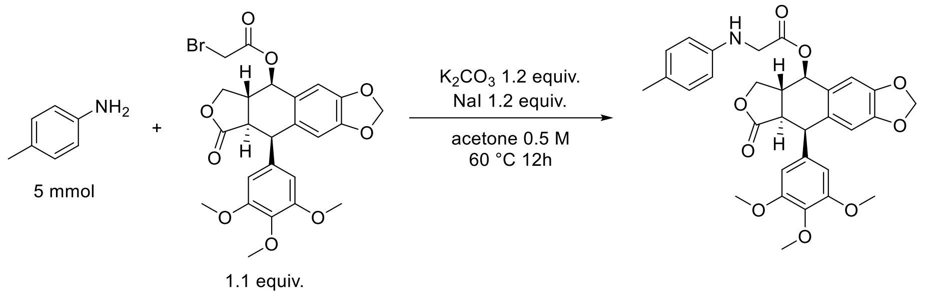

The mixture of *p*-toluidine (5.0 mmol, 0.5 g), potassium carbonate (6.0 mmol, 0.8 g) and sodium iodide (6.0 mmol, 0.9 g) were treated with the corresponding bromoacetate-ester (5.5 mmol, 2.9 g), in d.r.y acetone at 60 °C for 12 h. After quenching the reaction with water (30 mL), the resulting mixture was separated and extracted with EtOAc (30 mL x 3). The combined organic layers were dried over Na_2SO_4, concentrated under reduced pressure, and purified with a silica gel column (using petroleum ether / MeOH = 20:1 as eluent) to give **1t** as a yellow solid (0.9 g, 61% yield). m.p. 98 – 99 ℃.

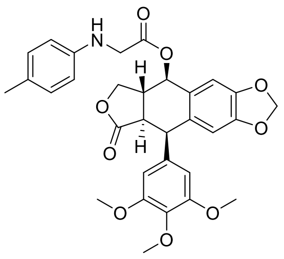

(5*R*,5a*R*,8a*R*,9*R*)-8-oxo-9-(3,4,5-trimethoxyphenyl)-5,5a,6,8,8a,9-hexahyd.r.ofuro[3',4':6,7]naphtho[2,3-d][1,3]dioxol-5-yl *p*-tolylglycinat (1t) According to the procedure, purified with a silica gel column (using petroleum ether / MeOH = 20:1 as eluent) to give **1t** as a yellow solid (0.9 g, 61% yield). m.p. 98 – 99 ℃. ^{1}H NMR (400 MHz, $CDCl_3$) δ 7.00 (d, *J* = 8.0 Hz, 2H), 6.66 (s, 1H), 6.55 – 6.53 (m, 3H), 6.37 (s, 2H), 5.98 – 5.96 (m, 2H), 5.92 (d, *J* = 8.8 Hz, 1H), 4.57 (d, *J* = 4.4 Hz, 1H), 4.28 – 4.24 (m, 1H), 4.17 – 4.08 (m, 2H), 4.04 – 3.96 (m, 2H), 3.81 (s, 3H), 3.75 (s, 6H), 2.91 – 2.75 (m, 2H), 2.23 (s, 3H) ppm. ^{13}C NMR (101 MHz, $CDCl_3$) δ 173.4, 172.0, 152.6, 148.1, 147.5, 144.3, 137.1, 134.7, 132.3, 129.9, 128.0, 127.7, 113.1, 109.7, 108.1, 106.8, 101.6, 74.4, 71.0, 60.6, 56.1, 46.3, 45.3, 43.6, 38.4, 20.3 ppm. HRMS (ESI) m/z: $[M + H]^+$ Calcd for $C_{31}H_{32}NO_9$ 562.2072; Found 562.2070.

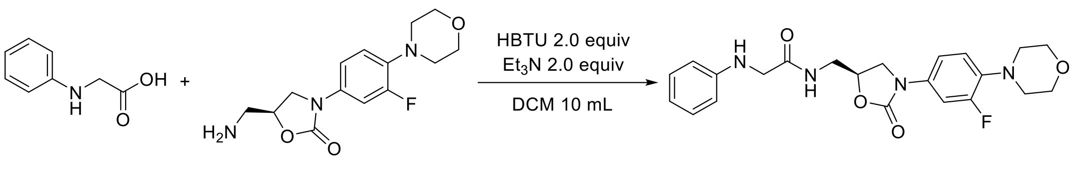

5 mmol 1.0 equiv 1u

To a 50 mL round-bottom flask, phenylglycine (5.0 mmol, 0.7 g), HBTU (10.0 mmol, 3.8 g), Et_3N (10.0 mmol, 1.0 g), and (*S*)-*N*-[[3-[3-fluoro-4-(4-morpholinyl)phenyl]-2-oxo-5-oxazolidinyl]methyl]amine (5.0 mmol, 1.5 g) were dissolved in DCM (20 mL) under N_2 atmosphere, and the mixture was stirred at 25 °C for 15 h. After quenching the reaction with water (30 mL), the resulting mixture was separated and extracted with EtOAc (30 mL x 3). The combined organic layers were dried over Na_2SO_4, concentrated under reduced pressure, and purified with a silica gel column (using petroleum ether / MeOH = 20:1 as eluent) to give **1u** as a white solid (1.1 g, 55% yield). m.p. 143 – 144 ℃.

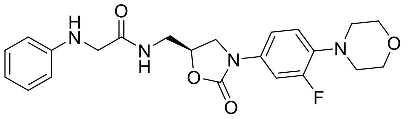

(*S*)-*N*-((3-(3-fluoro-4-morpholinophenyl)-2-oxooxazolidin-5-yl)methyl)-2-(phenylamino)acetamide (1u) According to the procedure, purified with a silica gel column (using petroleum ether / MeOH = 20:1 as eluent) to give **1u** as a white solid (1.1 g, 55% yield). m.p. 143 – 144 ℃. ^{1}H NMR (400 MHz, $CDCl_3$) δ 7.40 (dd, *J* = 14.4, 2.4 Hz, 1H), 7.26 – 7.22 (m, 1H), 7.16 – 7.10 (m, 2H), 7.06 – 7.03 (m, 1H), 6.94 – 6.89 (m, 1H), 6.79 – 6.75 (m, 1H), 6.57 – 6.54 (m, 2H), 4.77 – 4.70 (m, 1H), 4.25 (br, 1H), 3.98 (t, *J* = 9.2 Hz, 1H), 3.88 – 3.86 (m, 4H), 3.81 (d, *J* = 4.8 Hz, 2H), 3.76 – 3.66 (m, 3H), 3.06 – 3.04 (m, 4H) ppm. ^{13}C NMR (101 MHz, $CDCl_3$) δ 171.9, 155.2 (d, $J_{C\text{-}F}$ = 247.1 Hz), 154.1, 146.9, 136.3 (d, $J_{C\text{-}F}$ = 9.0 Hz), 132.7(d, $J_{C\text{-}F}$ = 10.5 Hz), 129.2, 118.7, 118.6 (d, $J_{C\text{-}F}$ = 4.1 Hz), 113.7 (d, $J_{C\text{-}F}$ = 3.3 Hz), 112.8, 107.2 (d, $J_{C\text{-}F}$ = 26.4 Hz), 71.5, 66.7, 50.8, 50.8, 48.2, 47.4, 41.4 ppm. ^{19}F NMR (376 MHz, $CDCl_3$) δ -120.1 ppm. HRMS (ESI) m/z: $[M + H]^+$ Calcd for $C_{22}H_{26}FN_4O_4$ 429.1933; Found 429.1925.

4.1.2 Styrene substrates

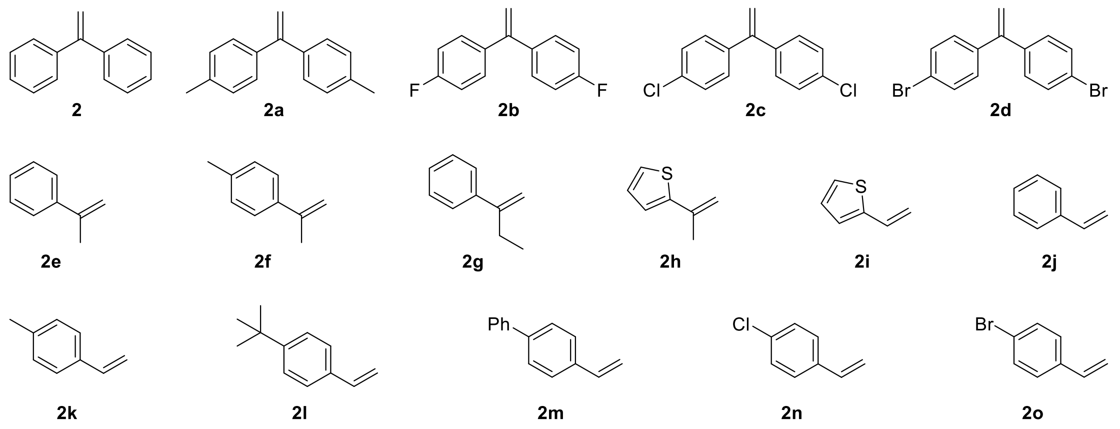

Figure S3 Styrene substrates.

Substrates **2**, **2e**, **2f**, **2g**, **2i**, **1j**, **2k**, **2l**, **2m**, **2n**, and **2o** were purchased from commercial sources.

Substrates **2a**[7], **2b**[7], **2c**[7], **2d**[7] and **2h**[8] were prepared according to the literature procedures.

4.1.3 Disulfide substrates

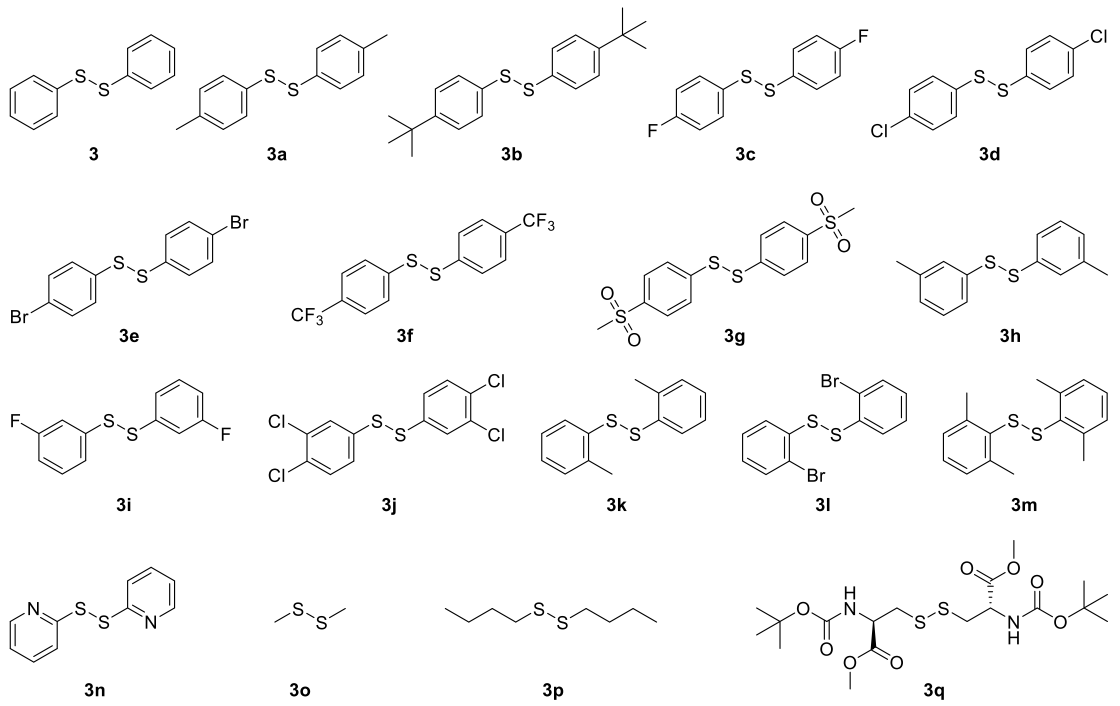

Figure S4 Disulfide substrates.

Substrates **3**, **3a**, **3n**, **3o** and **3p** were purchased from commercial sources.

Substrates **3b**[9], **3c**[9], **3d**[9], **3e**[9], **3f**[9], **3g**[10], **3h**[9], **3i**[9], **3j**[9], **3k**[9], **3l**[9], **3m**[9], and **3q**[11] were prepared according to the literature procedures.

4.1.4 Thiosulfonate substrates

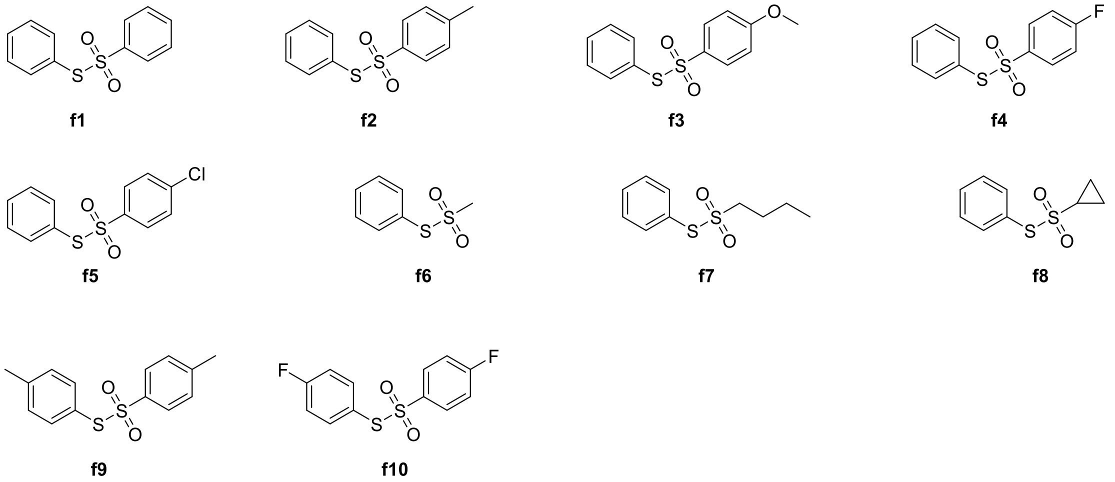

Figure S5 Thiosulfonate substrates.

Substrates **f1**[12], **f2**[13], **f3**[14], **f4**[13], **f5**[13], **f6**[13], **f7**[14], **f8**[15], **f9**[12]and **f10**[12] were prepared according to the literature procedures.

4.2 Synthesis of sulfanyl glycine derivative products

4.2.1 General procedure for the preparation of sulfanyl glycine derivative products

The reactions were carried out in 8 mL transparent vials. Each vial was equipped with a magnetic stir bar and charged with the [Ir(ppy)$_2$(dtbbpy)](PF$_6$) (0.0015 mmol, 0.5 mol%), glycine derivative (0.3 mmol), styrene (0.36 mmol), disulfide (0.45 mmol) and MeCN (3 mL). The reaction vial was closed under air, placed in a home-made photochemical (LED) reactor (Figure S1), magnetically stirred and irradiated with a 15 W blue LED (462 nm) at 25 °C for 10 h. After quenching the reaction with water (5 mL), the resulting mixture was separated and extracted with EtOAc (10 mL x 3). The combined organic layers were dried over Na_2SO_4, concentrated under reduced pressure, and purified through a silica gel column (EtOAc and petro ether as the elution).

Products **4 - 57** were prepared according to this procedure.

4.2.2 General procedure for the preparation of sulfonyl glycine derivative

products

The reactions were carried out in 8 mL transparent vials. Each vial was equipped with a magnetic stir bar and charged with the [Ir(ppy)$_2$(dtbbpy)](PF$_6$) (0.0015 mmol, 0.5 mol%), glycine derivative (0.3 mmol), ethylene (0.36 mmol), thiosulfonate (0.45 mmol)) and DMF (3 mL). The reaction vial was closed under air, placed in a home-made photochemical (LED) reactor (Figure S1), magnetically stirred and irradiated with a 15 W blue LED (462 nm) at 25 °C for 10 h. After quenching the reaction with water (5 mL), the resulting mixture was separated and extracted with EtOAc (10 mL x 3). The combined organic layers were dried over Na_2SO_4, concentrated under reduced pressure, and purified through a silica gel column (EtOAc and petro ether as the elution).

Products **58 - 71** were prepared according to this synthesis procedure.

4.2.3 Characterization data of sulfanyl glycine derivative products

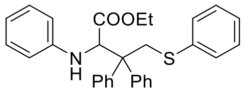

ethyl 3,3-diphenyl-2-(phenylamino)-4-(phenylthio)butanoate (4) According to the general procedure for the preparation of sulfanyl glycine derivative, *N*-phenylglycine ethyl ester **1** (0.3 mmol, 53.7 mg), 1,1-diphenylethylene **2** (0.36 mmol, 64.8 mg), diphenyl disulfide **3** (0.45 mmol, 98.1 mg) were used. The product was isolated by a silica gel column chromatography using petroleum ether / EtOAc = 20:1 as eluent to give **4** as a white solid (96.7 mg, 69% yield). m.p. 117 – 118 ℃. ^{1}H NMR (400 MHz, $CDCl_3$) δ 7.39 – 7.32 (m, 5H), 7.30 – 7.13 (m, 12H), 6.81 – 6.77 (m, 1H), 6.75 – 6.73 (m, 2H), 5.3 (s, 1H), 4.18 – 4.09 (m, 2H), 3.94– 3.80 (m, 2H), 0.93 (t, J = 7.2 Hz, 3H) ppm. ^{13}C NMR (101 MHz, $CDCl_3$) δ 172.1, 146.4, 142.9, 142.2, 136.8, 130.4, 129.3, 129.2, 129.1, 128.7, 127.8, 127.6, 127.3, 127.0, 126.3, 118.7, 114.0, 61.2, 60.9, 53.9, 45.1, 13.7 ppm. HRMS (ESI) m/z: $[M + H]^+$ Calcd for $C_{30}H_{30}NO_2S$ 468.1992; Found 468.1983.

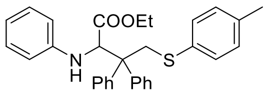

ethyl 3,3-diphenyl-2-(phenylamino)-4-(*p*-tolylthio)butanoate (5) According to the general procedure for the preparation of sulfanyl glycine derivative, *N*-phenylglycine ethyl ester **1** (0.3 mmol, 53.7 mg), 1,1-diphenylethylene **2** (0.36 mmol, 64.8 mg), 1,2-di-*p*-tolyldisulfane **3a** (0.45 mmol, 110.7 mg) were used. The product was isolated by a silica gel column chromatography using petroleum ether / EtOAc = 20:1 as eluent to give **5** as a yellowish oil (93.8 mg, 65% yield). ^{1}H NMR (400 MHz, $CDCl_3$) δ 7.45 – 7.36 (m, 5H), 7.32 – 7.24 (m, 9H), 7.08 – 7.06 (m, 2H), 6.86 – 6.79 (m, 3H), 5.37 (s, 1H), 4.75 (br, 1H), 4.22 – 4.12 (m, 2H), 3.99 – 3.85 (m, 2H), 2.35 (s, 3H), 0.98 (t, *J* = 7.2 Hz, 3H) ppm. ^{13}C NMR (101 MHz, $CDCl_3$) δ 172.0, 146.4, 143.0, 142.3, 136.4, 133.0, 131.0, 129.4, 129.2, 129.1, 127.8, 127.6, 127.2, 126.9, 118.6, 113.9, 61.3, 60.7, 53.8, 45.7, 20.9, 13.6 ppm. HRMS (ESI) m/z: $[M + H]^+$ Calcd for $C_{31}H_{32}NO_2S$ 482.2148; Found 482.2140.

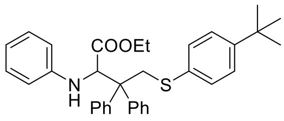

ethyl 4-((4-(*tert*-butyl)phenyl)thio)-3,3-diphenyl-2-(phenylamino)butanoate (6) According to the general procedure for the preparation of sulfanyl glycine derivative, *N*-phenylglycine ethyl ester **1** (0.3 mmol, 53.7 mg), 1,1-diphenylethylene **2** (0.36 mmol, 64.8 mg), 1,2-bis(4-(*tert*-butyl)phenyl)disulfane **3b** (0.45 mmol, 148.6 mg) were used. The product was isolated by a silica gel column chromatography using petroleum ether / EtOAc = 20:1 as eluent to give **6** as a yellowish oil (86.3 mg, 55% yield). ^{1}H NMR (400 MHz, $CDCl_3$) δ 7.39 – 7.32 (m, 6H), 7.26– 7.20 (m, 10H), 6.82 – 6.74 (m, 3H), 5.32 (s, 1H), 4.73 (br, 1H), 4.19 – 4.09 (m, 2H), 3.95 – 3.80 (m, 2H), 1.32 (s, 9H), 0.94 (t, *J* = 7.2 Hz, 3H) ppm. ^{13}C NMR (101 MHz, $CDCl_3$) δ 172.1, 149.6, 146.5, 143.1, 142.3, 133.2, 130.7, 129.3, 129.1, 127.8, 127.6, 127.2, 126.9, 125.7, 118.6, 114.0, 61.4, 60.8, 53.8, 45.5, 34.4, 31.2, 13.7 ppm. HRMS (ESI) m/z: $[M + H]^+$ Calcd for $C_{34}H_{38}NO_2S$ 524.2618; Found 524.2617.

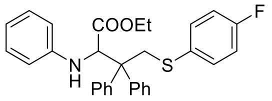

ethyl 4-((4-fluorophenyl)thio)-3,3-diphenyl-2-(phenylamino)butanoate (7) According to the general procedure for the preparation of sulfanyl glycine derivative, *N*-phenylglycine ethyl ester **1** (0.3 mmol, 53.7 mg), 1,1-diphenylethylene **2** (0.36 mmol, 64.8 mg), 1,2-bis(4-fluorophenyl)disulfane **3c** (0.45 mmol, 114.3 mg) were used. The product was isolated by a silica gel column chromatography using petroleum ether / EtOAc = 20:1 as eluent to give **7** as a yellowish viscous oil (77.1 mg, 53% yield). ^{1}H NMR (400 MHz, $CDCl_3$) δ 7.35 – 7.31 (m, 5H), 7.25 – 7.17 (m, 9H), 6.89 – 6.83 (m, 2H), 6.81 – 6.73 (m, 1H), 6.74 – 6.72 (m, 2H), 5.32 – 5.30 (m, 1H), 4.52 – 4.51 (m, 1H), 4.09 – 3.94 (m, 2H), 3.94 – 3.82 (m, 2H), 0.94 (t, *J* = 6.8 Hz, 3H) ppm. ^{13}C NMR (101 MHz, $CDCl_3$) δ 172.1, 161.8 (d, $J_{C\text{-}F}$ = 247.6 Hz), 146.4, 142.4 (d, $J_{C\text{-}F}$ = 63.1 Hz), 133.3 (d, $J_{C\text{-}F}$ = 8.2 Hz), 131.7 (d, $J_{C\text{-}F}$ = 3.3 Hz), 129.3, 129.2 (d, $J_{C\text{-}F}$ = 6.3 Hz), 127.9, 127.6, 127.3, 127.0, 118.8, 115.8, 115.6, 114.0, 60.9, 60.9, 54.1, 46.6, 13.7 ppm. ^{19}F NMR (376 MHz, $CDCl_3$) δ -115.2 ppm. HRMS (ESI) m/z: $[M + H]^+$ Calcd for $C_{30}H_{29}FNO_2S$ 486.1898; Found 486.1896.

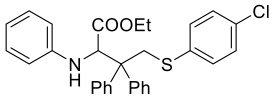

ethyl 4-((4-chlorophenyl)thio)-3,3-diphenyl-2-(phenylamino)butanoate (8) According to the general procedure for the preparation of sulfanyl glycine derivative, *N*-phenylglycine ethyl ester **1** (0.3 mmol, 53.7 mg), 1,1-diphenylethylene **2** (0.36 mmol, 64.8 mg), 1,2-bis(4-chlorophenyl)disulfane **3d** (0.45 mmol, 128.7 mg) were used. The product was isolated by a silica gel column chromatography using petroleum ether / EtOAc = 20:1 as eluent to give **8** as a yellowish oil (100.7 mg, 67% yield). ^{1}H NMR (400 MHz, $CDCl_3$) δ 7.36 – 7.33 (m, 5H), 7.26 – 7.19 (m, 7H), 7.17 – 7.12 (m, 4H), 6.80 (t, *J* = 7.6 Hz, 1H), 6.74 – 6.72 (m, 2H), 5.31 (s, 1H), 4.46 (br, 1H), 4.08 (s, 2H), 3.92 – 3.85 (m, 2H), 0.93 (t, *J* = 7.2 Hz, 3H) ppm. ^{13}C NMR (101 MHz, $CDCl_3$) δ 172.0, 146.4, 142.6, 142.0, 135.3, 132.3, 131.8, 129.3, 129.2, 129.1, 128.7, 127.9, 127.7, 127.4, 127.1, 118.9, 114.1, 61.0, 60.9, 54.1, 45.6, 13.7 ppm. HRMS (ESI) m/z: $[M + H]^+$ Calcd

for $C_{30}H_{29}ClNO_2S$ 502.1602; Found 502.1594.

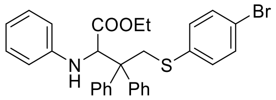

ethyl 4-((4-bromophenyl)thio)-3,3-diphenyl-2-(phenylamino)butanoate (9) According to the general procedure for the preparation of sulfanyl glycine derivative, *N*-phenylglycine ethyl ester **1** (0.3 mmol, 53.7 mg), 1,1-diphenylethylene **2** (0.36 mmol, 64.8 mg), 1,2-bis(4-bromophenyl)disulfane **3e** (0.45 mmol, 168.2 mg) were used. The product was isolated by a silica gel column chromatography using petroleum ether / EtOAc = 20:1 as eluent to give **9** as a yellowish oil (94.8 mg, 58% yield). ^{1}H NMR (400 MHz, $CDCl_3$) δ 7.35 – 7.31 (m, 5H), 7.29 – 7.23 (m, 5H), 7.22 – 7.18 (m, 4H), 7.10 – 7.06 (m, 2H), 6.79 (t, *J* = 7.6 Hz, 1H), 6.73 –6.71 (m, 2H), 5.29 (d, *J* = 10.8 Hz, 1H), 4.44 (d, *J*= 10.8 Hz, 1H), 4.07 (s, 2H), 3.91 – 3.83 (m, 2H), 0.93 (t, *J* = 7.2 Hz, 3H) ppm. ^{13}C NMR (101 MHz, $CDCl_3$) δ 172.0, 146.4, 142.5, 141.9, 136.0, 132.0, 131.6, 129.3, 129.2, 129.1, 127.9, 127.7, 127.4, 127.1, 120.2, 118.9, 114.1, 60.9, 60.9, 54.1, 45.5, 13.7 ppm. HRMS (ESI) m/z: $[M + H]^+$ Calcd for $C_{30}H_{29}BrNO_2S$ 546.1097; Found 546.1090.

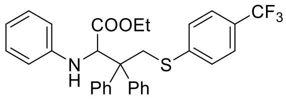

ethyl 3,3-diphenyl-2-(phenylamino)-4-((4-(trifluoromethyl)phenyl)thio)butanoate (10) According to the general procedure for the preparation of sulfanyl glycine derivative, *N*-phenylglycine ethyl ester **1** (0.3 mmol, 53.7 mg), 1,1-diphenylethylene **2** (0.36 mmol, 64.8 mg), 1,2-bis(4-(trifluoromethyl)phenyl)disulfane **3f** (0.45 mmol, 159.3 mg) were used. The product was isolated by a silica gel column chromatography using petroleum ether / EtOAc = 20:1 as eluent to give **10** as a yellowish oil (83.5 mg, 52% yield). ^{1}H NMR (400 MHz, $CDCl_3$) δ 7.40 – 7.31 (m, 7H), 7.28 – 7.17 (m, 9H), 6.81 – 6.77 (m, 1H), 6.73 – 6.70 (m, 2H), 5.31 (s, 1H), 4.34 (br, 1H), 4.17 – 4.09 (m, 2H), 3.93 – 3.82 (m, 2H), 0.92 (t, *J* = 7.2 Hz, 3H) ppm. ^{13}C NMR (101 MHz, $CDCl_3$) δ 171.9, 146.4, 142.3, 142.1, 141.8, 129.3, 129.2, 129.1, 129.1, 127.9, 127.7, 127.5, 127.2,

125.3 (q, $J_{C\text{-}F}$ = 3.8 Hz), 124.1 (q, $J_{C\text{-}F}$ = 272.7 Hz), 119.0, 114.2, 61.0, 60.9, 54.2, 44.3, 13.7 ppm. ^{19}F NMR (376 MHz, $CDCl_3$) δ -62.5 ppm. HRMS (ESI) m/z: $[M + H]^+$ Calcd for $C_{31}H_{29}F_3NO_2S$ 536.1866; Found 536.1865.

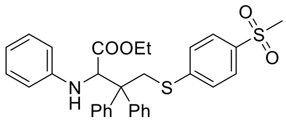

ethyl 4-((4-(methylsulfonyl)phenyl)thio)-3,3-diphenyl-2-(phenylamino)butanoate (11) According to the general procedure for the preparation of sulfanyl glycine derivative, *N*-phenylglycine ethyl ester **1** (0.3 mmol, 53.7 mg), 1,1-diphenylethylene **2** (0.36 mmol, 64.8 mg), 1,2-bis(4-(methylsulfonyl)phenyl)disulfane **3g** (0.45 mmol, 168.3 mg) were used. The product was isolated by a silica gel column chromatography using petroleum ether / EtOAc = 5:1 as eluent to give **11** as a yellowish viscous oil (112.9 mg, 69% yield). ^{1}H NMR (400 MHz, $CDCl_3$) δ 7.68 – 7.66 (m, 2H), 7.39 – 7.29 (m, 7H), 7.26 – 7.17 (m, 7H), 6.78 (t, J = 7.2 Hz, 1H), 6.72 – 6.70 (m, 2H), 5.37 (s, 1H), 4.27 (br, 1H), 4.19 – 4.11 (m, 2H), 3.87 (q, J = 7.2 Hz, 2H), 2.99 (s, 3H), 0.91 (t, J = 7.2 Hz, 3H) ppm. ^{13}C NMR (101 MHz, $CDCl_3$) δ 171.7, 146.2, 145.3, 141.9, 141.4, 136.9, 129.2, 129.2, 129.0, 128.4, 127.9, 127.7, 127.5, 127.3, 127.2, 119.0, 114.1, 61.0, 60.8, 54.2, 44.4, 43.5, 13.6 ppm. HRMS (ESI) m/z: $[M + H]^+$ Calcd for $C_{31}H_{32}NO_4S_2$ 546.1767; Found 546.1761.

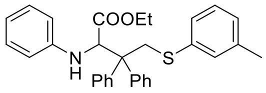

ethyl 3,3-diphenyl-2-(phenylamino)-4-(m-tolylthio)butanoate (12) According to the general procedure for the preparation of sulfanyl glycine derivative, *N*-phenylglycine ethyl ester **1** (0.3 mmol, 53.7 mg), 1,1-diphenylethylene **2** (0.36 mmol, 64.8 mg), 1,2-di-*m*-tolyldisulfane **3h** (0.45 mmol, 110.7 mg) were used. The product was isolated by a silica gel column chromatography using petroleum ether / EtOAc = 20:1 as eluent to give **12** as a yellowish oil (85.2 mg, 59% yield). ^{1}H NMR (400 MHz, $CDCl_3$) δ 7.39 – 7.31 (m, 5H), 7.27 – 7.19 (m, 7H), 7.11 – 7.06 (m, 2H), 7.01 (s, 1H), 6.96 – 6.94 (m, 1H), 6.79 (t, J = 7.2 Hz, 1H), 6.76 – 6.73 (m, 2H), 5.32 (s, 1H), 4.56 (br, 1H), 4.15 –

4.06 (m, 2H), 3.94 – 3.81 (m, 2H), 2.25 (s, 3H), 0.94 (t, J = 7.2 Hz, 3H) ppm. ^{13}C NMR (101 MHz, $CDCl_3$) δ 172.1, 146.4, 142.9, 142.2, 138.3, 136.5, 131.1, 129.3, 129.2, 129.2, 128.5, 127.8, 127.6, 127.5, 127.2, 127.2, 127.0, 118.7, 114.0, 61.1, 60.8, 54.0, 45.2, 21.2, 13.7 ppm. HRMS (ESI) m/z: $[M + H]^+$ Calcd for $C_{31}H_{32}NO_2S$ 482.2148; Found 482.2147.

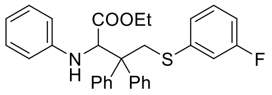

ethyl 4-((3-fluorophenyl)thio)-3,3-diphenyl-2-(phenylamino)butanoate (13)

According to the general procedure for the preparation of sulfanyl glycine derivative, *N*-phenylglycine ethyl ester **1** (0.3 mmol, 53.7 mg), 1,1-diphenylethylene **2** (0.36 mmol, 64.8 mg), 1,2-bis(3-fluorophenyl)disulfane **3i** (0.45 mmol, 114.3 mg) were used. The product was isolated by a silica gel column chromatography using petroleum ether / EtOAc = 20:1 as eluent to give **13** as a yellowish oil (80.1 mg, 55% yield). ^{1}H NMR (400 MHz, $CDCl_3$) δ 7.39 – 7.32 (m, 5H), 7.28 – 7.20 (m, 7H), 7.16 – 7.11 (m, 1H), 7.04 – 7.01 (m, 1H), 6.93 – 6.89 (m, 1H), 6.84 – 6.79 (m, 2H), 6.76 – 6.74 (m, 2H), 5.33 (s, 1H), 4.42 (br, 1H), 4.15 – 4.08 (m, 2H), 3.96 – 3.85 (m, 2H), 0.95 (t, J = 7.2 Hz, 3H) ppm. ^{13}C NMR (101 MHz, $CDCl_3$) δ 171.9, 162.4 (d, J_{C-F} = 248.8 Hz), 146.4, 142.2 (d, J_{C-F} = 57.3 Hz), 139.2 (d, J_{C-F} = 7.9 Hz), 129.8 (d, J_{C-F} = 8.6 Hz), 129.3, 129.2, 129.1, 127.9, 127.7, 127.4, 127.1, 125.4 (d, J_{C-F} = 3.0 Hz), 118.9, 116.6 (d, J_{C-F} = 22.9 Hz), 114.1, 113.0 (d, J_{C-F} = 21.3 Hz), 60.9, 60.9, 54.0, 44.9, 13.7 ppm. ^{19}F NMR (376 MHz, $CDCl_3$) δ -112.5 ppm. HRMS (ESI) m/z: $[M + H]^+$ Calcd for $C_{30}H_{29}FNO_2S$ 486.1898; Found 486.1893.

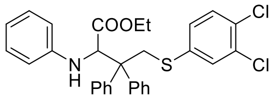

ethyl 4-((3,4-dichlorophenyl)thio)-3,3-diphenyl-2-(phenylamino)butanoate (14)

According to the general procedure for the preparation of sulfanyl glycine derivative, *N*-phenylglycine ethyl ester **1** (0.3 mmol, 53.7 mg), 1,1-diphenylethylene **2** (0.36 mmol, 64.8 mg), 1,2-bis(3,4-dichlorophenyl)disulfane **3j** (0.45 mmol, 159.2 mg) were used.

The product was isolated by a silica gel column chromatography using petroleum ether / EtOAc = 20:1 as eluent to give **14** as a yellowish oil (85.1 mg, 53% yield). ^{1}H NMR (400 MHz, $CDCl_3$) δ 7.36 – 7.32 (m, 5H), 7.26 – 7.15 (m, 9H), 7.04 – 7.01 (m, 1H), 6.81 (t, J = 7.6 Hz, 1H), 6.74 – 6.72 (m, 2H), 5.32 (s, 1H), 4.28 (br, 1H), 4.09 – 4.01 (m, 2H), 3.91 (q, J = 6.8 Hz, 2H), 0.96 (t, J = 7.2 Hz, 3H) ppm. ^{13}C NMR (101 MHz, $CDCl_3$) δ 171.9, 146.3, 142.1, 141.7, 137.0, 132.3, 131.7, 130.3, 130.1, 129.6, 129.3, 129.3, 129.2, 127.9, 127.7, 127.5, 127.2, 119.0, 114.1, 61.0, 60.5, 54.4, 45.7, 13.7 ppm. HRMS (ESI) m/z: $[M + H]^+$ Calcd for $C_{30}H_{28}Cl_2NO_2S$ 536.1212; Found 536.1214.

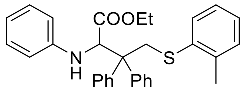

ethyl 3,3-diphenyl-2-(phenylamino)-4-(o-tolylthio)butanoate (15) According to the general procedure for the preparation of sulfanyl glycine derivative, *N*-phenylglycine ethyl ester **1** (0.3 mmol, 53.7 mg), 1,1-diphenylethylene **2** (0.36 mmol, 64.8 mg), 1,2-di-*o*-tolyldisulfane **3k** (0.45 mmol, 110.7 mg) were used. The product was isolated by a silica gel column chromatography using petroleum ether / EtOAc = 20:1 as eluent to give **15** as a yellowish oil (69.3 mg, 48% yield). ^{1}H NMR (400 MHz, $CDCl_3$) δ 7.39 – 7.31 (m, 5H), 7.29 – 7.19 (m, 8H), 7.13 – 7.06 (m, 3H), 6.81 – 6.72 (m, 3H), 5.29 (s, 1H), 4.64 (br, 1H), 4.12 – 3.98 (m, 2H), 3.91 – 3.77 (m, 2H), 2.34 (s, 3H), 0.90 (t, J = 7.2 Hz, 3H) ppm. ^{13}C NMR (101 MHz, $CDCl_3$) δ 172.1, 146.4, 143.0, 142.2, 138.8, 136.0, 130.6, 129.9, 129.3, 129.1, 129.1, 127.8, 127.6, 127.2, 127.0, 126.4, 126.3, 118.7, 114.0, 61.4, 60.8, 53.8, 44.5, 20.7, 13.7 ppm. HRMS (ESI) m/z: $[M + H]^+$ Calcd for $C_{31}H_{32}NO_2S$ 482.2148; Found 482.2145.

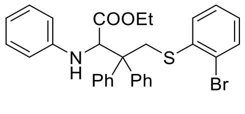

ethyl 4-((2-bromophenyl)thio)-3,3-diphenyl-2-(phenylamino)butanoate (16) According to the general procedure for the preparation of sulfanyl glycine derivative, *N*-phenylglycine ethyl ester **1** (0.3 mmol, 53.7 mg), 1,1-diphenylethylene **2** (0.36 mmol, 64.8 mg), 1,2-bis(2-bromophenyl)disulfane **3l** (0.45 mmol, 168.2 mg) were used. The

product was isolated by a silica gel column chromatography using petroleum ether / EtOAc = 20:1 as eluent to give **16** as a yellowish oil (93.2 mg, 57% yield). ^{1}H NMR (400 MHz, $CDCl_3$) δ 7.52 – 7.50 (m, 1H), 7.42 – 7.33 (m, 5H), 7.27 – 7.22 (m, 8H), 7.16 – 7.12 (m, 1H), 7.03 – 6.98 (m, 1H), 6.84 – 6.78 (m, 3H), 5.36 (s, 1H), 4.55 (br, 1H), 4.20 – 4.07 (m, 2H), 3.93 – 3.84 (m, 2H), 0.95 (t, J = 7.2 Hz, 3H) ppm. ^{13}C NMR (101 MHz, $CDCl_3$) δ 171.9, 146.4, 142.6, 141.9, 137.8, 132.8, 131.1, 129.2, 129.1, 127.8, 127.6, 127.5, 127.3, 127.3, 127.0, 125.6, 118.8, 114.1, 61.2, 60.8, 53.9, 44.4, 13.7 ppm. HRMS (ESI) m/z: $[M + H]^+$ Calcd for $C_{30}H_{29}BrNO_2S$ 546.1097; Found 546.1096.

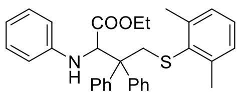

ethyl 4-((2,6-dimethylphenyl)thio)-3,3-diphenyl-2-(phenylamino)butanoate (17) According to the general procedure for the preparation of sulfanyl glycine derivative, *N*-phenylglycine ethyl ester **1** (0.3 mmol, 53.7 mg), 1,1-diphenylethylene **2** (0.36 mmol, 64.8 mg), 1,2-bis(2,6-dimethylphenyl)disulfane **3m** (0.45 mmol, 123.3 mg) were used. The product was isolated by a silica gel column chromatography using petroleum ether / EtOAc = 20:1 as eluent to give **17** as a yellowish oil (46.1 mg, 31% yield). ^{1}H NMR (400 MHz, $CDCl_3$) δ 7.39 – 7.32 (m, 5H), 7.28 – 7.25 (m, 3H), 7.24 – 719 (m, 4H), 7.15 – 7.08 (m, 3H), 6.82 – 6.75 (m, 3H), 5.19 – 5.11 (m, 2H), 3.96 – 3.93 (m, 1H), 3.88 – 3.80 (m, 1H), 3.77 – 3.69 (m, 2H), 2.48 (s, 6H), 0.87 (t, J = 7.2 Hz, 3H) ppm. ^{13}C NMR (101 MHz, $CDCl_3$) δ 172.1, 146.4, 143.9, 143.0, 142.7, 134.0, 129.3, 128.9, 128.9, 128.2, 128.1, 127.8, 127.7, 127.1, 126.9, 118.5, 113.7, 62.1, 60.7, 53.3, 44.0, 21.8, 13.6 ppm. HRMS (ESI) m/z: $[M + H]^+$ Calcd for $C_{32}H_{34}NO_2S$ 496.2305; Found 496.2296.

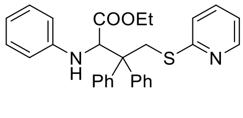

ethyl 3,3-diphenyl-2-(phenylamino)-4-(pyridin-2-ylthio)butanoate (18) According to the general procedure for the preparation of sulfanyl glycine derivative, *N*-

phenylglycine ethyl ester **1** (0.3 mmol, 53.7 mg), 1,1-diphenylethylene **2** (0.36 mmol, 64.8 mg), 1,2-di(pyridin-2-yl)disulfane **3n** (0.45 mmol, 99.0 mg) were used. The product was isolated by a silica gel column chromatography using petroleum ether / EtOAc = 10:1 as eluent to give **18** as a yellowish oil (78.7 mg, 56% yield). ^{1}H NMR (400 MHz, $CDCl_3$) δ 8.32 – 8.31 (m, 1H), 7.39 – 7.29 (m, 6H), 7.28 – 7.24 (m, 2H), 7.22 – 7.15 (m, 5H), 7.00 – 6.98 (m, 1H), 6.90 – 6.87 (m, 1H), 6.76 (t, J = 7.2 Hz, 1H), 6.71 – 6.69 (m, 2H), 5.27 (d, J = 10.8 Hz, 1H), 4.50 – 4.34 (m, 2H), 4.26 (d, J = 11.2 Hz, 1H), 3.96 – 3.83 (m, 2H), 0.94 (t, J = 7.2 Hz, 3H) ppm. ^{13}C NMR (101 MHz, $CDCl_3$) δ 171.9, 158.5, 148.9, 146.4, 142.8, 142.1, 135.6, 129.4, 129.3, 129.2, 127.7, 127.4, 127.2, 126.8, 122.8, 119.4, 118.7, 114.1, 60.9, 60.7, 54.0, 39.8, 13.7 ppm. HRMS (ESI) m/z: $[M + H]^+$ Calcd for $C_{29}H_{29}N_2O_2S$ 469.1944; Found 469.1939.

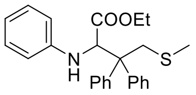

ethyl 4-(methylthio)-3,3-diphenyl-2-(phenylamino)butanoate (19) According to the general procedure for the preparation of sulfanyl glycine derivative, *N*-phenylglycine ethyl ester **1** (0.3 mmol, 53.7 mg), 1,1-diphenylethylene **2** (0.36 mmol, 64.8 mg), 1,2-dimethyldisulfane **3o** (0.45 mmol, 42.3 mg) were used. The product was isolated by a silica gel column chromatography using petroleum ether / EtOAc = 20:1 as eluent to give **19** as a yellowish oil (79.0 mg, 65% yield). ^{1}H NMR (400 MHz, $CDCl_3$) δ 7.37 – 7.32 (m, 5H), 7.32 – 7.27 (m, 3H), 7.24 – 7.19 (m, 4H), 6.80 – 6.73 (m, 3H), 5.31 (s, 1H), 4.50 (br, 1H), 3.91 (q, J = 7.2 Hz, 2H), 3.70 – 3.57 (m, 2H), 1.71 (s, 3H), 0.99 (t, J = 7.2 Hz, 3H) ppm. ^{13}C NMR (101 MHz, $CDCl_3$) δ 172.1, 146.6, 143.2, 142.5, 129.3, 129.3, 129.1, 127.8, 127.6, 127.2, 126.8, 118.6, 113.9, 60.8, 60.7, 54.4, 44.7, 17.1, 13.7 ppm. HRMS (ESI) m/z: $[M + H]^+$ Calcd for $C_{25}H_{28}NO_2S$ 406.1835; Found 406.1836.

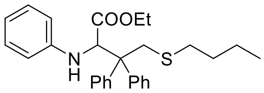

ethyl 4-(butylthio)-3,3-diphenyl-2-(phenylamino)butanoate (20) According to the general procedure for the preparation of sulfanyl glycine derivative, *N*-phenylglycine

ethyl ester **1** (0.3 mmol, 53.7 mg), 1,1-diphenylethylene **2** (0.36 mmol, 64.8 mg), 1,2-dibutyldisulfane **3p** (0.45 mmol, 80.1 mg) were used. The product was isolated by a silica gel column chromatography using petroleum ether / EtOAc = 20:1 as eluent to give **20** as a yellowish oil (96.6 mg, 72% yield). ^{1}H NMR (400 MHz, $CDCl_3$) δ 7.36 – 7.30 (m, 5H), 7.29 – 7.25 (m, 3H), 7.21 – 7.17 (m, 4H), 6.78 – 6.71 (m, 3H), 5.26 (s, 1H), 3.94 – 3.82 (m, 2H), 3.69 – 3.62 (m, 2H), 2.14 – 2.03 (m, 2H), 1.47 – 1.39 (m, 2H), 1.32 – 1.22 (m, 3H), 0.96 (t, *J* = 7.2 Hz, 3H), 0.83 (t, *J* = 7.2 Hz, 3H) ppm. ^{13}C NMR (101 MHz, $CDCl_3$) δ 172.2, 146.6, 143.5, 142.6, 129.3, 129.1, 127.8, 127.6, 127.1, 126.8, 118.5, 113.8, 61.1, 60.7, 54.1, 41.8, 33.2, 31.6, 21.9, 13.8, 13.6 ppm. HRMS (ESI) m/z: $[M + H]^+$ Calcd for $C_{28}H_{34}NO_2S$ 448.2305; Found 448.2299.

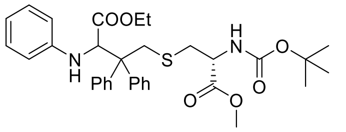

ethyl 4-(((*R*)-2-((*tert*-butoxycarbonyl)amino)-3-methoxy-3-oxopropyl)thio)-3,3-diphenyl-2-(phenylamino)butanoate (21) According to the general procedure for the preparation of sulfanyl glycine derivative, *N*-phenylglycine ethyl ester **1** (0.3 mmol, 53.7 mg), 1,1-diphenylethylene **2** (0.36 mmol, 64.8 mg), methyl *N*-(tert-butoxycarbonyl)-*S*-(((*R*)-2-((*tert*-butoxycarbonyl)amino)-3-methoxy-3-oxopropyl)thio)-*D*-cysteinate **3q** (0.45 mmol, 210.7 mg) were used. The product was isolated by a silica gel column chromatography using petroleum ether / EtOAc = 5:1 as eluent to give **21** as a yellowish oil (97.7 mg, 55% yield). The d.r. is not determined since there is no distinguishable integrations presenting in the ^{1}H NMR spectrum. The data is written as observed. ^{1}H NMR (400 MHz, $CDCl_3$) 7.37 – 7.28 (m, 8H), 7.23 – 7.20 (m, 4H), 6.82 – 6.72 (m, 3H), 5.35 – 5.28 (m, 2H), 4.43 – 4.32 (m, 2H), 3.94 – 3.88 (m, 2H), 3.75 – 3.73 (m, 3H), 3.71 – 3.57 (m, 2H), 2.54 – 2.41 (m, 2H), 1.47 – 1.46 (m, 9H), 0.97 (t, *J* = 7.2 Hz, 3H) ppm. ^{13}C NMR (101 MHz, $CDCl_3$) δ 171.9, 171.9, 171.4, 155.0, 146.5, 142.7, 142.6, 142.1, 142.0, 129.3, 129.2, 129.2, 129.1, 129.1, 127.8, 127.7, 127.3, 127.3, 127.0, 126.9, 118.8, 118.7, 114.3, 114.3, 114.0, 114.0, 79.9, 60.8, 60.5, 60.2, 54.5, 54.4, 53.3, 53.2, 52.3, 42.8, 42.7, 35.6, 35.5, 28.2, 14.0, 13.7 ppm.

HRMS (ESI) m/z: $[M + H]^+$ Calcd for $C_{33}H_{41}N_2O_6S$ 593.2680; Found 593.2675.

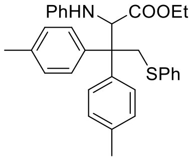

ethyl 2-(phenylamino)-4-(phenylthio)-3,3-di-*p*-tolylbutanoate (22) According to the general procedure for the preparation of sulfanyl glycine derivative, *N*-phenylglycine ethyl ester **1** (0.3 mmol, 53.7 mg), 4,4'-(ethene-1,1-diyl)bis(methylbenzene) **2a** (0.36 mmol, 74.9 mg), diphenyl disulfide **3** (0.45 mmol, 98.1 mg) were used. The product was isolated by a silica gel column chromatography using petroleum ether / EtOAc = 20:1 as eluent to give **22** as a yellowish oil (96.6 mg, 65% yield). ^{1}H NMR (400 MHz, $CDCl_3$) δ 7.28 – 7.13 (m, 11H), 7.10 – 7.03 (m, 4H), 6.79 – 6.70 (m, 3H), 5.27 (s, 1H), 4.12 – 4.02 (m, 2H), 3.93 – 3.84 (m, 2H), 2.36 (s, 3H), 2.32 (s, 3H), 0.94 (t, J = 7.2 Hz, 3H) ppm. ^{13}C NMR (101 MHz, $CDCl_3$) δ 172.2, 146.5, 139.8, 139.1, 137.0, 136.9, 136.5, 130.3, 129.2, 129.0, 129.0, 128.6, 128.5, 128.3, 126.1, 118.6, 114.0, 61.0, 60.8, 53.3, 45.4, 21.0, 20.9, 13.7 ppm. HRMS (ESI) m/z: $[M + H]^+$ Calcd for $C_{32}H_{34}NO_2S$ 496.2305; Found 296.2300.

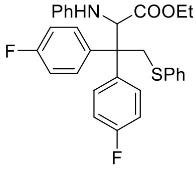

ethyl 3,3-bis(4-fluorophenyl)-2-(phenylamino)-4-(phenylthio)butanoate (23) According to the general procedure for the preparation of sulfanyl glycine derivative, *N*-phenylglycine ethyl ester **1** (0.3 mmol, 53.7 mg), 4,4'-(ethene-1,1-diyl)bis(fluorobenzene) **2b** (0.36 mmol, 77.8 mg), diphenyl disulfide **3** (0.45 mmol, 98.1 mg) were used. The product was isolated by a silica gel column chromatography using petroleum ether / EtOAc = 20:1 as eluent to give **23** as a yellowish solid (75.5 mg, 50% yield). m.p. 113 – 114 ℃. ^{1}H NMR (400 MHz, $CDCl_3$) δ 7.32 – 7.28 (m, 2H), 7.23 – 7.12 (m, 9H), 7.03 – 6.99 (m, 2H), 6.94 – 6.89 (m, 2H), 6.83 – 6.78 (m, 1H), 6.74 – 6.72 (m, 2H), 5.24 (s, 1H), 4.34 (br, 1H), 4.06 – 3.98 (m, 2H), 3.94 – 3.86 (m,

2H), 0.97 (t, J = 7.2 Hz, 3H) ppm. ^{13}C NMR (101 MHz, $CDCl_3$) δ 171.8, 161.9 (d, J_{C-F} = 248.5 Hz), 161.7 (d, J_{C-F} = 248.3 Hz), 146.2, 138.2 (d, J_{C-F} = 3.5 Hz), 137.7 (d, J_{C-F} = 3.5 Hz), 136.4, 130.9, 130.9, 130.5, 129.4, 128.7, 126.5, 119.2, 114.6 (d, J_{C-F} = 42.9 Hz), 114.4 (d, J_{C-F} = 36.3 Hz), 61.2, 61.1, 53.4, 45.5, 13.8 ppm. ^{19}F NMR (376 MHz, $CDCl_3$) δ -114.6, -115.1 ppm. HRMS (ESI) m/z: $[M + H]^+$ Calcd for $C_{30}H_{28}F_2NO_2S$ 504.1803; Found 504.1806.

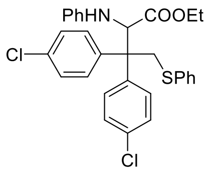

ethyl 3,3-bis(4-chlorophenyl)-2-(phenylamino)-4-(phenylthio)butanoate (24)
According to the general procedure for the preparation of sulfanyl glycine derivative, *N*-phenylglycine ethyl ester **1** (0.3 mmol, 53.7 mg), 4,4'-(ethene-1,1-diyl)bis(chlorobenzene) **2c** (0.36 mmol, 89.3 mg), diphenyl disulfide **3** (0.45 mmol, 98.1 mg) were used. The product was isolated by a silica gel column chromatography using petroleum ether / EtOAc = 20:1 as eluent to give **24** as a yellowish viscous oil (96.3 mg, 60% yield). ^{1}H NMR (400 MHz, $CDCl_3$) δ 7.30 – 7.27 (m, 2H), 7.25 – 7.14 (m, 11H), 7.10 – 7.06 (m, 2H), 6.83 – 6.79 (m, 1H), 6.74 – 6.72 (m, 2H), 5.24 (s, 1H), 4.03 – 3.95 (m, 2H), 3.91 (qd, J = 7.2, 1.6 Hz, 2H), 0.97 (t, J = 7.2 Hz, 3H) ppm. ^{13}C NMR (101 MHz, $CDCl_3$) δ 171.7, 146.1, 140.7, 140.2, 136.2, 133.5, 133.2, 130.7, 130.6, 129.4, 128.7, 128.0, 127.8, 126.5, 119.3, 114.3, 61.2, 60.8, 53.7, 45.3, 13.8 ppm. HRMS (ESI) m/z: $[M + H]^+$ Calcd for $C_{30}H_{28}Cl_2NO_2S$ 536.1212; Found 536.1210.

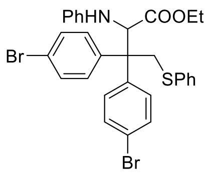

ethyl 3,3-bis(4-bromophenyl)-2-(phenylamino)-4-(phenylthio)butanoate (25)
According to the general procedure for the preparation of sulfanyl glycine derivative, *N*-phenylglycine ethyl ester **1** (0.3 mmol, 53.7 mg), 4,4'-(ethene-1,1-

diyl)bis(bromobenzene) **2d** (0.36 mmol, 120.9 mg), diphenyl disulfide **3** (0.45 mmol, 98.1 mg) were used. The product was isolated by a silica gel column chromatography using petroleum ether / EtOAc = 20:1 as eluent to give **25** as a yellowish viscous oil (106.5 mg, 57% yield). ^{1}H NMR (400 MHz, $CDCl_3$) δ 7.45 – 7.41 (m, 2H), 7.33 – 7.31 (m, 2H), 7.22 – 7.14 (m, 9H), 7.04 – 7.00 (m, 2H), 6.83 – 6.79 (m, 1H), 6.74 – 6.71 (m, 2H), 5.23 (s, 1H), 4.01 – 3.95 (m, 2H), 3.91 (qd, *J* = 7.2, 1.6 Hz, 2H), 0.98 (t, *J* = 7.2 Hz, 3H) ppm. ^{13}C NMR (101 MHz, $CDCl_3$) δ 171.7, 146.1, 141.2, 140.7, 136.1, 131.0, 130.9, 130.8, 130.7, 129.4, 128.7, 126.5, 121.8, 121.4, 119.3, 114.3, 61.2, 60.7, 53.9, 45.2, 13.8 ppm. HRMS (ESI) m/z: $[M + H]^+$ Calcd for $C_{30}H_{28}Br_2NO_2S$ 624.0202; Found 624.0196.

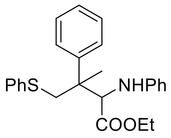

ethyl 3-methyl-3-phenyl-2-(phenylamino)-4-(phenylthio)butanoate (26) According to the general procedure for the preparation of sulfanyl glycine derivative, *N*-phenylglycine ethyl ester **1** (0.3 mmol, 53.7 mg), prop-1-en-2-ylbenzene **2e** (0.36 mmol, 42.5 mg), diphenyl disulfide **3** (0.45 mmol, 98.1 mg) were used. The product was isolated by a silica gel column chromatography using petroleum ether / EtOAc = 20:1 as eluent to give **26** as a yellowish oil (70.5 mg, 58% yield, 1.2:1 d.r.). d.r. is determined by ^{1}H NMR. The data is written as observed. ^{1}H NMR (400 MHz, $CDCl_3$) δ 7.46 – 7.43 (m, 4H), 7.41 – 7.36 (m, 6H), 7.34 – 7.31 (m, 6H), 7.28 – 7.27 (m, 1H), 7.26 – 7.23 (m, 4H), 7.21 – 7.15 (m, 5H), 6.84 – 6.73 (m, 5H), 6.66 – 6.64 (m, 2H), 4.44 – 4.42 (m, 2H) 4.10 – 4.04 (m, 2.2H), 3.88 – 3.82 (m, 3.3H), 3.80 – 3.77 (m, 1.3H), 3.67 – 3.64 (m, 1.2H), 3.55 – 3.52 (m, 1H), 1.75 (s, 3H, minor isomer), 1.73 (s, 3.6H, major isomer), 1.14 (t, *J* = 7.2 Hz, 3H, minor isomer), 0.90 (t, *J* = 7.2 Hz, 3.6H, major isomer) ppm. ^{13}C NMR (101 MHz, $CDCl_3$) δ172.0, 171.9, 147.2, 147.0, 141.6, 141.1, 137.5, 137.4, 129.6, 129.5, 129.3, 129.1, 128.8, 128.7, 128.2, 128.1, 127.1, 127.1, 127.1, 127.0, 126.0, 125.9, 118.9, 118.9, 114.3, 114.3, 65.8, 65.2, 60.9, 60.7, 46.3, 45.9, 44.9, 44.3, 21.7, 19.6, 14.0, 13.6 ppm. HRMS (ESI) m/z: $[M + H]^+$ Calcd for $C_{25}H_{28}NO_2S$ 406.1835;

Found 406.1831.

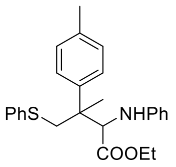

ethyl 3-methyl-2-(phenylamino)-4-(phenylthio)-3-(*p*-tolyl)butanoate (27) According to the general procedure for the preparation of sulfanyl glycine derivative, *N*-phenylglycine ethyl ester **1** (0.3 mmol, 53.7 mg), 1-methyl-4-(prop-1-en-2-yl)benzene **2f** (0.36 mmol, 47.6 mg), diphenyl disulfide **3** (0.45 mmol, 98.1 mg) were used. The product was isolated by a silica gel column chromatography using petroleum ether / EtOAc = 20:1 as eluent to give **27** as a yellowish oil (52.8 mg, 42% yield, 1:1 d.r.). d.r. is determined by ^{1}H NMR. The data is written as observed. ^{1}H NMR (400 MHz, $CDCl_3$) δ 7.33 – 7.27 (m, 7H), 7.25 – 7.21 (m, 5H), 7.19 – 7.12 (m, 10H), 6.80 – 6.61 (m, 6H), 4.38 – 4.36 (m, 2H), 4.22 (br, 1H), 4.10 – 4.04 (m, 2H), 4.00 – 3.94 (m, 1H), 3.88 – 3.72 (m, 4H), 3.61 – 3.47 (m, 2H), 2.36 (s, 6H), 1.69 – 1.68 (m, 6H), 1.13 (t, *J* = 6.8 Hz, 3H, isomer-1), 0.90 (t, *J* = 7.2 Hz, 3H, isomer-2) ppm. ^{13}C NMR (101 MHz, $CDCl_3$) δ 172.1, 172.0, 147.2, 147.0, 138.4, 137.9, 137.7, 137.5, 136.8, 136.7, 129.6, 129.5, 129.3, 129.2, 128.9, 128.9, 128.8, 128.7, 127.0, 126.9, 125.9, 125.8, 118.9, 118.8, 114.3, 114.3, 65.8, 65.1, 60.9, 60.7, 45.9, 45.5, 44.9, 44.5, 21.8, 20.9, 20.0, 14.0, 13.6 ppm. HRMS (ESI) m/z: $[M + H]^+$ Calcd for $C_{26}H_{30}NO_2S$ 420.1992; Found 420.1989.

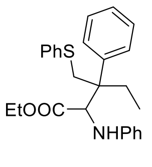

ethyl 3-phenyl-2-(phenylamino)-3-((phenylthio)methyl)pentanoate (28) According to the general procedure for the preparation of sulfanyl glycine derivative, *N*-phenylglycine ethyl ester **1** (0.3 mmol, 53.7 mg), but-1-en-2-ylbenzene **2g** (0.36 mmol, 47.6 mg), diphenyl disulfide **3** (0.45 mmol, 98.1 mg) were used. The product was isolated by a silica gel column chromatography using petroleum ether / EtOAc = 20:1 as eluent to give **28** as a yellowish oil (44.0 mg, 35% yield, 1:1 d.r.). d.r. is determined

by ^{1}H NMR. The data is written as observed. ^{1}H NMR (400 MHz, $CDCl_3$) δ 7.53 – 7.50 (m, 2H), 7.44 – 7.40 (m, 2H), 7.39 – 7.31 (m, 12H), 7.25 – 7.16 (m, 6H), 7.13 – 7.09 (m, 2H), 6.77 – 6.62 (m, 6H), 4.70 (s, 1H), 4.60 (s,1H), 4.18 (br, 1H), 4.08 – 3.99 (m, 2H), 3.97 – 3.87 (m, 4H), 3.82 (br, 1H), 3.70 – 3.54 (m, 2H), 2.40 – 2.10 (m, 4H), 1.12 (t, *J* = 6.8 Hz, 3H, isomer-1), 1.01 (t, *J* = 7.2 Hz, 3H, isomer-2), 0.88 (t, *J* = 7.2 Hz, 3H, isomer-1), 0.81 (t, *J* = 7.2 Hz, 3H, isomer-2) ppm. ^{13}C NMR (101 MHz, $CDCl_3$) δ 172.2, 171.8, 147.5, 147.1, 140.2, 139.5, 137.1, 136.7, 130.4, 130.3, 129.3, 129.1, 128.9, 128.9, 128.2, 128.1, 127.7, 127.2, 127.1, 127.1, 126.4, 126.3, 118.8, 118.4, 114.7, 113.9, 62.8, 62.4, 60.9, 60.7, 49.0, 48.9, 40.1, 39.8, 26.6, 26.2, 14.0, 13.9, 8.3, 8.1 ppm. HRMS (ESI) m/z: $[M + H]^+$ Calcd for $C_{26}H_{30}NO_2S$ 420.1992; Found 420.1985.

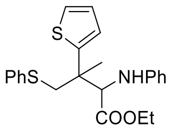

ethyl 3-methyl-2-(phenylamino)-4-(phenylthio)-3-(thiophen-2-yl)butanoate (29)

According to the general procedure for the preparation of sulfanyl glycine derivative, *N*-phenylglycine ethyl ester **1** (0.3 mmol, 53.7 mg), 2-(prop-1-en-2-yl)thiophene **2h** (0.36 mmol, 44.7 mg), diphenyl disulfide **3** (0.45 mmol, 98.1 mg) were used. The product was isolated by a silica gel column chromatography using petroleum ether / EtOAc = 20:1 as eluent to give **29** as a yellowish viscous oil (64.1 mg, 52% yield, 1:1 d.r.). d.r. is determined by ^{1}H NMR. The data is written as observed. ^{1}H NMR (400 MHz, $CDCl_3$) δ 7.40 – 7.39 (m, 4H), 7.31 – 7.28 (m, 4H), 7.25 – 7.17 (m, 8H), 7.06 – 7.04 (m, 1H), 7.02 – 7.01 (m, 3H), 6.84 – 6.79 (m, 2H), 6.76 – 6.73 (m, 4H), 4.51 – 4.48 (m, 2H), 4.33 – 4.21 (m, 2H), 4.14 – 4.05 (m, 2H), 4.03 – 3.96 (m, 2H), 3.75 – 3.61 (m, 4H), 1.76 (s, 3H, major isomer-1), 1.74 (s, 3H, minor isomer-2), 1.17 (t, *J* = 7.2 Hz, 3H, major isomer-1), 1.06 (t, *J* = 7.2 Hz, 3H, minor isomer-2) ppm. ^{13}C NMR (101 MHz, $CDCl_3$) δ 171.7, 171.6, 147.1, 147.1, 146.9, 146.5, 137.2, 137.0, 129.9, 129.8, 129.3, 129.2, 128.8, 128.8, 126.7, 126.5, 126.2, 126.1, 125.1, 125.1, 124.6, 124.5, 119.0, 118.9, 114.7, 114.3, 65.4, 64.2, 61.0, 45.8, 45.4, 23.7, 22.0, 14.0, 13.8 ppm. HRMS (ESI) m/z: $[M + H]^+$ Calcd for $C_{23}H_{26}NO_2S_2$ 412.1399; Found 412.1393.

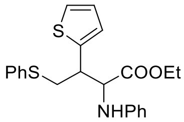

ethyl 2-(phenylamino)-4-(phenylthio)-3-(thiophen-2-yl)butanoate (30) According to the general procedure for the preparation of sulfanyl glycine derivative, *N*-phenylglycine ethyl ester **1** (0.3 mmol, 53.7 mg), 2-vinylthiophene **2i** (0.36 mmol, 39.6 mg), diphenyl disulfide **3** (0.45 mmol, 98.1 mg) were used. The product was isolated by a silica gel column chromatography using petroleum ether / EtOAc = 20:1 as eluent to give **30** as a yellowish viscous oil (62.0 mg, 52% yield, 1:1 d.r.). d.r. is determined by ^{1}H NMR of the crude product. ^{1}H NMR (400 MHz, $CDCl_3$) δ isomer-1 7.44 – 7.41 (m, 2H), 7.33 – 7.28 (m, 2H), 7.26 – 7.18 (m, 4H), 7.00 – 6.98 (m, 1H), 6.87 – 6.85 (m, 1H), 6.81 – 6.77 (m, 3H), 4.83 (dd, *J*= 10.4, 3.2 Hz, 1H), 4.14 – 4.05 (m, 3H), 3.84 – 3.80 (m, 1H), 3.49 – 3.32 (m, 2H), 1.20 (t, *J* = 7.2 Hz, 3H) ppm. ^{13}C NMR (101 MHz, $CDCl_3$) δisomer-1 172.4, 147.5, 140.8, 135.1, 130.5, 129.3, 129.0, 126.8, 126.7, 125.8, 124.8, 119.0, 114.7, 61.3, 59.0, 43.4, 37.5, 14.1 ppm. HRMS (ESI) m/z: $[M + H]^+$ Calcd for $C_{22}H_{24}NO_2S_2$ 398.1243; Found 398.1234. ^{1}H NMR (400 MHz, $CDCl_3$) δ isomer-2 7.39 – 7.36 (m, 2H), 7.32 – 7.28 (m, 2H), 7.24 – 7.19 (m, 2H), 7.15 – 7.11 (m, 2H), 6.98 – 6.95 (m, 1H), 6.87 – 6.86 (m, 1H), 6.76 – 6.72 (m, 1H), 6.61 – 6.59 (m, 2H), 4.59 – 4.56 (m, 1H), 4.38 – 4.36 (m, 1H), 4.07 (qd, *J* = 7.6, 1.2 Hz, 2H), 3.69 – 3.63 (m, 2H), 3.45 – 3.39 (m, 1H), 1.15 (t, *J* = 7.2 Hz, 3H) ppm. ^{13}C NMR (101 MHz, $CDCl_3$) δ isomer-2 171.4, 146.1, 141.5, 135.6, 129.8, 129.3, 129.1, 126.6, 126.5, 126.0, 124.8, 118.6, 113.9, 61.4, 60.4, 43.4, 37.4, 14.0 ppm. HRMS (ESI) m/z: $[M + H]^+$ Calcd for $C_{22}H_{24}NO_2S_2$ 398.1243; Found 398.1234.

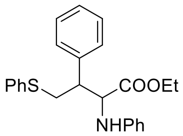

ethyl 3-phenyl-2-(phenylamino)-4-(phenylthio)butanoate (31) According to the general procedure for the preparation of sulfanyl glycine derivative, *N*-phenylglycine ethyl ester **1** (0.3 mmol, 53.7 mg), styrene **2j** (0.36 mmol, 37.5 mg), diphenyl disulfide **3** (0.45 mmol, 98.1 mg) were used. The product was isolated by a silica gel column

chromatography using petroleum ether / EtOAc = 20:1 as eluent to give **31** as a yellowish oil (59.8 mg, 51% yield, 1.2:1 d.r.). d.r. is determined by ^{1}H NMR of the crude product. ^{1}H NMR (400 MHz, $CDCl_3$) δ major isomer 7.43 – 7.40 (m, 2H), 7.37 – 7.29 (m, 5H), 7.25 – 7.16 (m, 5H), 6.81 – 6.74 (m, 3H), 4.80 (s, 1H), 4.14 – 4.08 (m, 2H), 3.88 (br, 1H), 3.56 – 3.46 (m, 2H), 3.38 – 3.34 (m, 1H), 1.20 (t, *J* = 7.2 Hz, 3H) ppm. ^{13}C NMR (101 MHz, $CDCl_3$) δ major isomer 172.7, 147.5, 138.4, 135.4, 130.2, 129.3, 129.0, 128.6, 128.3, 127.7, 126.5, 118.9, 114.5, 61.2, 59.3, 47.3, 36.2, 14.1 ppm. HRMS (ESI) m/z: $[M + H]^+$ Calcd for $C_{24}H_{26}NO_2S$ 392.1679; Found 392.1675. ^{1}H NMR (400 MHz, $CDCl_3$) δ minor isomer 7.36 – 7.26 (m, 7H), 7.22 – 7.13 (m, 5H), 6.77 – 6.73(m, 1H), 6.63 – 6.61 (m, 2H), 4.49 (d, *J* = 6.4 Hz, 1H), 4.23 (br, 1H), 4.00 – 3.92 (m, 2H), 3.74 – 3.69 (m, 1H), 3.42 – 3.27 (m, 2H), 1.02 (t, *J* = 7.2 Hz, 3H) ppm. ^{13}C NMR (101 MHz, $CDCl_3$) δ minor isomer 171.9, 146.4, 138.7, 135.9, 129.6, 129.3, 129.0, 128.4, 128.4 127.6, 126.3, 118.6, 113.9, 61.1, 60.9, 48.2, 36.3, 13.8 ppm. HRMS (ESI) m/z: $[M + H]^+$ Calcd for $C_{24}H_{26}NO_2S$ 392.1679; Found 392.1676.

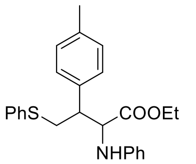

ethyl 2-(phenylamino)-4-(phenylthio)-3-(p-tolyl)butanoate (32) According to the general procedure for the preparation of sulfanyl glycine derivative, *N*-phenylglycine ethyl ester **1** (0.3 mmol, 53.7 mg), 1-methyl-4-vinylbenzene **2k** (0.36 mmol, 42.5 mg), diphenyl disulfide **3** (0.45 mmol, 98.1 mg) were used. The product was isolated by a silica gel column chromatography using petroleum ether / EtOAc = 20:1 as eluent to give **32** as a yellowish oil (57.1 mg, 47% yield, 1.2:1 d.r.). d.r. is determined by ^{1}H NMR of the crude product. ^{1}H NMR (400 MHz, $CDCl_3$) δ major isomer 7.44 – 7.42 (m, 2H), 7.34 – 7.30 (m, 2H), 7.25 – 7.17 (m, 5H), 7.09 – 7.07 (m, 2H), 6.82 – 6.76 (m, 3H), 4.80 (d, *J* = 3.6 Hz, 1H), 4.16 – 4.11 (m, 2H), 3.56 – 3.46 (m, 2H), 3.39 – 3.35 (m, 1H), 2.38 (s, 3H), 1.22 (t, *J* = 7.2 Hz, 3H) ppm. ^{13}C NMR (101 MHz, $CDCl_3$) δ major isomer 172.8, 147.5, 137.3, 135.5, 135.2, 130.1, 129.3, 129.2, 128.9, 128.1, 126.4, 118.8, 114.5, 61.1, 59.3, 46.9, 36.2, 21.0, 14.1 ppm. HRMS (ESI) m/z: $[M + H]^+$ Calcd

for $C_{25}H_{28}NO_2S$ 406.1835; Found 406.1831. ^{1}H NMR (400 MHz, $CDCl_3$) δ minor isomer 7.36 – 7.34 (m, 2H), 7.30 – 7.27 (m, 2H), 7.22 – 7.13 (m, 5H), 7.08 – 7.06 (m, 2H), 6.77 – 6.73 (m, 1H), 6.63 – 6.61 (m, 2H), 4.49 (d, *J* = 6.4 Hz, 1H), 4.01 – 3.96 (m, 2H), 3.73 – 3.68 (m, 1H), 3.41 – 3.35 (m, 1H), 3.30 – 3.25 (m, 1H), 2.35 (s, 3H), 1.05 (t, *J* = 6.8 Hz, 3H) ppm. ^{13}C NMR (101 MHz, $CDCl_3$) δ minor isomer 171.9, 146.4, 137.3, 136.0, 135.5, 129.5, 129.3, 129.1, 128.9, 128.3, 126.2, 118.5, 113.9, 61.0, 60.8, 47.6, 36.4, 21.1, 13.9 ppm. HRMS (ESI) m/z: $[M + H]^+$ Calcd for $C_{25}H_{28}NO_2S$ 406.1835; Found 406.1830.

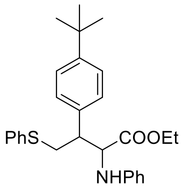

ethyl 3-(4-(*tert*-butyl)phenyl)-2-(phenylamino)-4-(phenylthio)butanoate (33) According to the general procedure for the preparation of sulfanyl glycine derivative, *N*-phenylglycine ethyl ester **1** (0.3 mmol, 53.7 mg), 1-(*tert*-butyl)-4-vinylbenzene **2l** (0.36 mmol, 57.6 mg), diphenyl disulfide **3** (0.45 mmol, 98.1 mg) were used. The product was isolated by a silica gel column chromatography using petroleum ether / EtOAc = 20:1 as eluent to give **33** as a yellowish oil (71.1 mg, 53% yield, 1.2:1 d.r.). d.r. is determined by ^{1}H NMR of the crude product. ^{1}H NMR (400 MHz, $CDCl_3$) δ major isomer 7.45 – 7.42 (m, 2H), 7.38 – 7.36 (m, 2H), 7.33 – 7.29 (m, 2H), 7.26 – 7.19 (m, 3H), 7.12 – 7.10 (m, 2H), 6.82 – 6.77 (m, 3H), 4.85 (d, *J* = 3.6 Hz, 1H), 4.13 (q, *J* = 7.2 Hz, 2H), 3.57 – 3.47 (m, 2H), 3.39 – 3.35 (m, 1H), 1.36 (s, 9H), 1.21 (t, *J* = 7.2 Hz, 3H) ppm. ^{13}C NMR (101 MHz, $CDCl_3$) δ major isomer 172.8, 150.4, 147.5, 135.4, 135.2, 130.3, 129.2, 128.9, 127.9, 126.5, 125.4, 118.8, 114.5, 61.0, 59.0, 46.8, 36.2, 34.4, 31.3, 14.1 ppm. HRMS (ESI) m/z: $[M + H]^+$ Calcd for $C_{28}H_{34}NO_2S$ 448.2305; Found 448.2303. ^{1}H NMR (400 MHz, $CDCl_3$) δ minor isomer 7.35 – 7.26 (m, 5H), 7.21 – 7.10 (m, 6H), 6.77 – 6.73 (m, 1H), 6.62 – 6.60 (m, 2H), 4.48 (d, *J* = 6.8 Hz, 1H), 3.95 (q, *J* = 7.2 Hz, 2H), 3.73 – 3.68 (m, 1H), 3.42 – 3.37 (m, 1H), 3.29 – 3.25 (m, 1H), 1.32 (s, 9H), 0.99 (t, *J* = 7.2 Hz, 3H) ppm. ^{13}C NMR (101 MHz, $CDCl_3$) δ

minor isomer 172.1, 150.4, 146.4, 136.1, 135.6, 129.5, 129.3, 128.9, 128.0, 126.2, 125.3, 118.5, 113.9, 61.0, 60.8, 47.9, 36.3, 34.4, 31.3, 13.8 ppm. HRMS (ESI) m/z: [M + H]$^+$ Calcd for $C_{28}H_{34}NO_2S$ 448.2305; Found 448.2300.

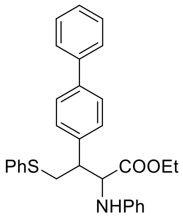

ethyl 3-([1,1'-biphenyl]-4-yl)-2-(phenylamino)-4-(phenylthio)butanoate (34) According to the general procedure for the preparation of sulfanyl glycine derivative, *N*-phenylglycine ethyl ester **1** (0.3 mmol, 53.7 mg), 4-vinyl-1,1'-biphenyl **2m** (0.36 mmol, 64.8 mg), diphenyl disulfide **3** (0.45 mmol, 98.1 mg) were used. The product was isolated by a silica gel column chromatography using petroleum ether / EtOAc = 20:1 as eluent to give **34** as a yellowish viscous oil (77.1 mg, 55% yield, 1.1:1 d.r.). d.r. is determined by ^{1}H NMR of the crude product. ^{1}H NMR (400 MHz, $CDCl_3$) δ major isomer 7.62 – 7.55 (m, 4H), 7.48 – 7.41 (m, 4H), 7.39 – 7.35 (m, 1H), 7.33 – 7.29 (m, 2H), 7.26 – 7.18 (m, 5H), 6.81 – 6.76 (m, 3H), 4.83 (s, 1H), 4.13 (q, *J* = 7.2 Hz, 2H), 3.91 (br, 1H), 3.58 – 3.50 (m, 2H), 3.43 – 3.38 (m, 1H), 1.21 (t, *J* = 7.2 Hz, 3H) ppm. ^{13}C NMR (101 MHz, $CDCl_3$) δ major isomer 172.8, 147.5, 140.6, 140.5, 137.4, 135.4, 130.3, 129.3, 129.0, 128.7, 128.7, 127.3, 127.2, 127.0, 126.6, 119.0, 114.6, 61.2, 59.4, 47.1, 36.3, 14.2 ppm. HRMS (ESI) m/z: [M + H]$^+$ Calcd for $C_{30}H_{30}NO_2S$ 468.1992; Found 468.1988. ^{1}H NMR (400 MHz, $CDCl_3$) δ minor isomer 7.63 – 7.56 (m, 4H), 7.49 – 7.45 (m, 2H), 7.40 – 7.36 (m, 3H), 7.33 – 7.27 (m, 4H), 7.25 – 7.16 (m, 3H), 6.81 – 6.77 (m, 1H), 6.68 – 6.65 (m, 2H), 4.55 (s, 1H), 4.30 (br, 1H), 4.01 (q, *J* = 7.2 Hz, 2H), 3.79 – 3.75 (m, 1H), 3.48 – 3.36 (m, 2H), 1.05 (t, *J* = 7.2 Hz, 3H) ppm. ^{13}C NMR (101 MHz, $CDCl_3$) δ minor isomer 171.9, 146.3, 140.6, 140.4, 137.7, 135.9, 129.6, 129.3, 129.0, 128.9, 128.7, 127.3, 127.1, 127.0, 126.3, 118.6, 113.9, 61.1, 60.9, 47.9, 36.3, 13.9 ppm. HRMS (ESI) m/z: [M + H]$^+$ Calcd for $C_{30}H_{30}NO_2S$ 468.1992; Found 468.1984.

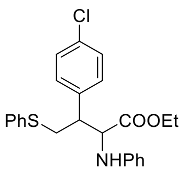

ethyl 3-(4-chlorophenyl)-2-(phenylamino)-4-(phenylthio)butanoate (35) According to the general procedure for the preparation of sulfanyl glycine derivative, *N*-phenylglycine ethyl ester **1** (0.3 mmol, 53.7 mg), 1-chloro-4-vinylbenzene **2n** (0.36 mmol, 49.7 mg), diphenyl disulfide **3** (0.45 mmol, 98.1 mg) were used. The product was isolated by a silica gel column chromatography using petroleum ether / EtOAc = 20:1 as eluent to give **35** as a yellowish oil (58.7 mg, 46% yield, 1.2:1 d.r.). d.r. is determined by ^{1}H NMR of the crude product. ^{1}H NMR (400 MHz, $CDCl_3$) δ major isomer 7.40 – 7.37 (m, 2H), 7.32 – 7.28 (m, 4H), 7.26 – 7.17 (m, 3H), 7.11 – 7.08 (m, 2H), 6.81 – 6.73 (m, 3H), 4.74 (d, *J* = 3.6 Hz, 1H), 4.11 (q, *J* = 7.2 Hz, 2H), 3.51 – 3.41 (m, 2H), 3.32 – 3.28 (m, 1H), 1.20 (t, *J* = 7.2 Hz, 3H) ppm. ^{13}C NMR (101 MHz, $CDCl_3$) δ major isomer 172.5, 147.3, 136.9, 135.1, 133.6, 130.3, 129.7, 129.3, 129.0, 128.7, 126.7, 119.1, 114.6, 61.3, 59.4, 46.9, 36.3, 14.1 ppm. HRMS (ESI) m/z: $[M + H]^+$ Calcd for $C_{24}H_{25}ClNO_2S$ 426.1289; Found 426.1285. ^{1}H NMR (400 MHz, $CDCl_3$) δ minor isomer 7.34 – 7.26 (m, 6H), 7.23 – 7.19 (m, 1H), 7.18 – 7.10 (m, 4H), 6.78 – 6.74 (m, 1H), 6.61 – 6.59 (m, 2H), 4.44 (d, *J* = 6.0 Hz, 1H), 4.21 (br, 1H), 4.02 – 3.94 (m, 2H), 3.70 – 3.66 (m, 1H), 3.35 – 3.25 (m, 2H), 1.06 (t, *J* = 7.2 Hz, 3H) ppm. ^{13}C NMR (101 MHz, $CDCl_3$) δ minor isomer 171.7, 146.2, 137.2, 135.6, 133.4, 129.8, 129.8, 129.3, 129.0, 128.6, 126.5, 118.8, 113.9, 61.2, 60.8, 47.6, 36.2, 13.9 ppm. HRMS (ESI) m/z: $[M + H]^+$ Calcd for $C_{24}H_{25}ClNO_2S$ 426.1289; Found 426.1281.

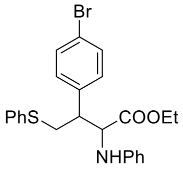

ethyl 3-(4-bromophenyl)-2-(phenylamino)-4-(phenylthio)butanoate (36) According to the general procedure for the preparation of sulfanyl glycine derivative, *N*-phenylglycine ethyl ester **1** (0.3 mmol, 53.7 mg), 1-bromo-4-vinylbenzene **2o** (0.36

mmol, 65.5 mg), diphenyl disulfide **3** (0.45 mmol, 98.1 mg) were used. The product was isolated by a silica gel column chromatography using petroleum ether / EtOAc = 20:1 as eluent to give **36** as a yellowish oil (52.1 mg, 37% yield, 1:1 d.r.). d.r. is determined by ^{1}H NMR of the crude product. ^{1}H NMR (400 MHz, $CDCl_3$) δ isomer-1 7.47 – 7.45 (m, 2H), 7.40 – 7.37 (m, 2H), 7.32 – 7.28 (m, 2H), 7.25 – 7.17 (m, 3H), 7.05 – 7.03 (m, 2H), 6.81 – 6.74 (m, 3H), 4.75 (s, 1H), 4.11 (q, *J* = 7.2 Hz, 2H), 3.83 (br, 1H), 3.51 – 3.40 (m, 2H), 3.32 – 3.28 (m, 1H), 1.21 (t, *J* = 7.2 Hz, 3H) ppm. ^{13}C NMR (101 MHz, $CDCl_3$) δ isomer-1 172.5, 147.3, 137.4, 135.1, 131.6, 130.4, 130.0, 129.3, 129.0, 126.7, 121.7, 119.1, 114.6, 61.3, 59.4, 47.0, 36.2, 14.1 ppm. HRMS (ESI) m/z: $[M + H]^+$ Calcd for $C_{24}H_{25}BrNO_2S$ 470.0784; Found 470.0779. ^{1}H NMR (400 MHz, $CDCl_3$) δ isomer-2 7.45 – 7.42 (m, 2H), 7.33 – 7.28 (m, 4H), 7.23 – 7.19 (m, 1H), 7.17 – 7.12 (m, 2H), 7.07 – 7.04 (m, 2H), 6.78 – 6.74 (m, 1H), 6.60 – 6.58 (m, 2H), 4,43 (d, *J* = 6.0 Hz, 1H), 4.02 – 3.94 (m, 2H), 3.69 – 3.65 (m, 1H), 3.35 – 3.23 (m, 2H), 1.05 (t, *J* = 7.2 Hz, 3H) ppm. ^{13}C NMR (101 MHz, $CDCl_3$) δ isomer-2 171.7, 146.2, 137.8, 135.6, 131.5, 130.2, 129.8, 129.4, 129.0, 126.5, 121.6, 118.8, 113.9, 61.3, 60.8, 47.7, 36.2, 13.9 ppm. HRMS (ESI) m/z: $[M + H]^+$ Calcd for $C_{24}H_{25}BrNO_2S$ 470.0784; Found 470.0778.

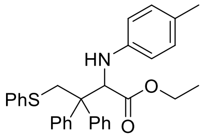

ethyl 3,3-diphenyl-4-(phenylthio)-2-(*p*-tolylamino)butanoate (37) According to the general procedure for the preparation of sulfanyl glycine derivative, ethyl *p*-tolylglycinate **1a** (0.3 mmol, 57.9 mg), 1,1-diphenylethylene **2** (0.36 mmol, 64.8 mg), diphenyl disulfide **3** (0.45 mmol, 98.1 mg) were used. The product was isolated by a silica gel column chromatography using petroleum ether / EtOAc = 20:1 as eluent to give **37** as a yellowish viscous oil (93.8 mg, 65% yield). ^{1}H NMR (400 MHz, $CDCl_3$) δ 7.40 – 7.32 (m, 5H), 7.30 – 7.13 (m, 10H), 7.05 – 7.03 (m, 2H), 6.69 – 6.67 (m, 2H), 5.30 (s, 1H), 4.44 (br, 1H), 4.19 – 4.09 (m, 2H), 3.95 – 3.82 (m, 2H), 2.28 (s, 3H), 0.95 (t, *J* = 7.2 Hz, 3H) ppm. ^{13}C NMR (101 MHz, $CDCl_3$) δ 172.2, 144.1, 142.9, 142.3, 136.9, 130.3, 129.8, 129.2, 129.2, 128.6, 127.9, 127.8, 127.6, 127.2, 126.9, 126.2, 114.2, 61.5, 60.8, 53.9, 45.1, 20.4, 13.7 ppm. HRMS (ESI) m/z: $[M + H]^+$ Calcd for

$C_{31}H_{32}NO_2S$ 482.2148; Found 482.2145.

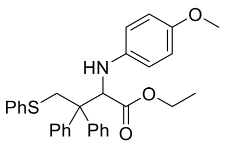

ethyl 2-((4-methoxyphenyl)amino)-3,3-diphenyl-4-(phenylthio)butanoate (38)
According to the general procedure for the preparation of sulfanyl glycine derivative, ethyl (4-methoxyphenyl)glycinate **1b** (0.3 mmol, 62.7 mg), 1,1-diphenylethylene **2** (0.36 mmol, 64.8 mg), diphenyl disulfide **3** (0.45 mmol, 98.1 mg) were used. The product was isolated by a silica gel column chromatography using petroleum ether / EtOAc = 20:1 as eluent to give **38** as a yellowish viscous oil (67.1 mg, 45% yield). ^{1}H NMR (400 MHz, $CDCl_3$) δ 7.38 – 7.30 (m, 5H), 7.26 – 7.24 (m, 7H), 7.20 – 7.11 (m, 3H), 6.79 – 6.69 (m, 4H), 5.22 (s, 1H), 4.17 – 4.02 (m, 2H), 3.89 – 3.81 (m, 2H), 3.75 (s, 3H), 0.92 (t, *J* = 7.2 Hz, 3H) ppm. ^{13}C NMR (101 MHz, $CDCl_3$) δ 172.3, 153.0, 142.9, 142.3, 140.5, 137.0, 130.3, 129.3, 129.3, 128.6, 127.7, 127.6, 127.2, 127.0, 126.2, 115.8, 114.8, 62.3, 60.8, 55.6, 54.1, 45.3, 13.7 ppm. HRMS (ESI) m/z: $[M + H]^+$ Calcd for $C_{31}H_{32}NO_3S$ 498.2097; Found 498.2098.

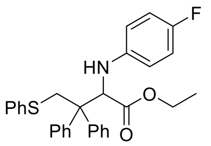

ethyl 2-((4-fluorophenyl)amino)-3,3-diphenyl-4-(phenylthio)butanoate (39)
According to the general procedure for the preparation of sulfanyl glycine derivative, ethyl (4-fluorophenyl)glycinate **1c** (0.3 mmol, 59.1 mg), 1,1-diphenylethylene **2** (0.36 mmol, 64.8 mg), diphenyl disulfide **3** (0.45 mmol, 98.1 mg) were used. The product was isolated by a silica gel column chromatography using petroleum ether / EtOAc = 20:1 as eluent to give **39** as a yellowish viscous oil (91.7 mg, 63% yield). ^{1}H NMR (400 MHz, $CDCl_3$) δ 7.38 – 7.32 (m, 5H), 7.29 – 7.26 (m, 5H), 7.23 – 7.13 (m, 5H), 6.94 – 6.89 (m, 2H), 6.70 – 6.67 (m, 2H), 5.25 (s, 1H), 4.42 (br, 1H), 4.20 – 4.05 (m, 2H), 3.92 – 3.83 (m, 2H), 0.94 (t, *J* = 7.2 Hz, 3H) ppm. ^{13}C NMR (101 MHz, $CDCl_3$) δ 172.0,

156.5 (d, $J_{C\text{-}F}$ = 237.5 Hz), 142.8, 142.8, 142.1, 136.8, 130.3, 129.2, 129.1, 128.7, 127.8, 127.6, 127.3, 127.1, 126.3, 115.7 (d, $J_{C\text{-}F}$ = 22.4 Hz), 115.2 (d, $J_{C\text{-}F}$ = 7.5 Hz), 61.9, 60.9, 54.0, 45.2, 13.7 ppm. ^{19}F NMR (376 MHz, $CDCl_3$) δ -126.2 ppm. HRMS (ESI) m/z: $[M + H]^+$ Calcd for $C_{30}H_{29}FNO_2S$ 486.1898; Found 486.1897.

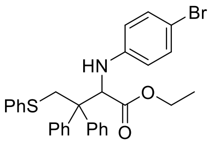

ethyl 2-((4-bromophenyl)amino)-3,3-diphenyl-4-(phenylthio)butanoate (40)

According to the general procedure for the preparation of sulfanyl glycine derivative, ethyl (4-bromophenyl)glycinate **1d** (0.3 mmol, 77.1 mg), 1,1-diphenylethylene **2** (0.36 mmol, 64.8 mg), diphenyl disulfide **3** (0.45 mmol, 98.1 mg) were used. The product was isolated by a silica gel column chromatography using petroleum ether / EtOAc = 20:1 as eluent to give **40** as a yellowish soild (106.3 mg, 65% yield). m.p. 148 – 149 ℃. ^{1}H NMR (400 MHz, $CDCl_3$) δ 7.38 – 7.32 (m, 5H), 7.30 – 7.25 (m, 7H), 7.22 – 7.13 (m, 5H), 6.63 – 6.59 (m, 2H), 5.24 (s, 1H), 4.18 – 4.07 (m, 2H), 3.95 – 3.80 (m, 2H), 0.94 (t, J = 7.2 Hz, 3H) ppm. ^{13}C NMR (101 MHz, $CDCl_3$) δ 171.8, 145.5, 142.8, 142.0, 136.6, 132.0, 130.4, 129.0, 129.0, 128.7, 127.9, 127.7, 127.4, 127.1, 126.4, 115.5, 110.5, 61.2, 61.0, 53.8, 45.0, 13.7 ppm. HRMS (ESI) m/z: $[M + H]^+$ Calcd for $C_{30}H_{29}BrNO_2S$ 546.1097; Found 546.1102.

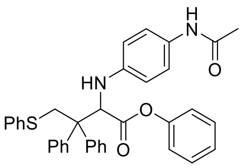

phenyl 2-((4-acetamidophenyl)amino)-3,3-diphenyl-4-(phenylthio)butanoate (41)

According to the general procedure for the preparation of sulfanyl glycine derivative, phenyl (4-acetamidophenyl)glycinate **1e** (0.3 mmol, 85.2 mg), 1,1-diphenylethylene **2** (0.36 mmol, 64.8 mg), diphenyl disulfide **3** (0.45 mmol, 98.1 mg) were used. The product was isolated by a silica gel column chromatography using petroleum ether / EtOAc = 1:1 as eluent to give **41** as a yellowish solid (60.1 mg, 35% yield). m.p. 93 –

94 ℃. ^{1}H NMR (400 MHz, $CDCl_3$) δ 7.45 – 7.43 (m, 3H), 7.40 – 7.33 (m, 5H), 7.31 – 7.29 (m, 6H), 7.23 – 7.10 (m, 6H), 6.80 – 6.77 (m, 2H), 6.57 – 6.54 (m, 2H), 5.53 – 5.51 (m, 1H), 4.80 – 4.77 (m, 1H), 4.27 – 4.17 (m, 2H), 2.08 (s, 3H) ppm. ^{13}C NMR (101 MHz, $CDCl_3$) δ 171.1, 168.2, 150.0, 143.0, 142.6, 142.0, 136.4, 130.5, 129.9, 129.2, 129.1, 128.7, 128.1, 127.9, 127.5, 127.2, 126.4, 125.9, 122.0, 121.1, 114.3, 61.9, 53.7, 45.0, 24.2 ppm. HRMS (ESI) m/z: $[M + H]^+$ Calcd for $C_{36}H_{33}N_2O_3S$ 573.2206; Found 573.2202.

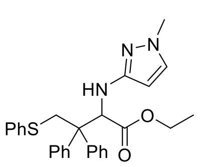

ethyl 2-((1-methyl-1H-pyrazol-3-yl)amino)-3,3-diphenyl-4-(phenylthio)butanoate (42) According to the general procedure for the preparation of sulfanyl glycine derivative, ethyl (1-methyl-1*H*-pyrazol-3-yl)glycinate **1f** (0.3 mmol, 54.9 mg), 1,1-diphenylethylene **2** (0.36 mmol, 64.8 mg), diphenyl disulfide **3** (0.45 mmol, 98.1 mg) were used. The product was isolated by a silica gel column chromatography using petroleum ether / EtOAc = 3:1 as eluent to give **42** as a yellowish viscous oil (66.4 mg, 47% yield). ^{1}H NMR (400 MHz, $CDCl_3$) δ 7.40 – 7.38 (m, 2H), 7.34 – 7.28 (m, 5H), 7.25 – 7.18 (m, 5H), 7.18 – 7.13 (m, 2H), 7.12 – 7.08 (m, 2H), 5.61 (d, J = 2.4 Hz, 1H), 5.40 – 5.37 (m, 1H), 4.26 – 4.23 (m, 1H), 4.09 – 4.02 (m, 2H), 3.90 (q, J = 7.2 Hz, 2H), 3.70 (s, 3H), 0.95 (t, J = 7.2 Hz, 3H) ppm. ^{13}C NMR (101 MHz, $CDCl_3$) δ 172.2, 155.4, 142.4, 141.9, 137.2, 130.9, 130.0, 129.3, 129.2, 128.4, 127.6, 127.4, 127.0, 126.7, 125.8, 91.4, 61.8, 60.7, 54.4, 45.3, 38.4, 13.6 ppm. HRMS (ESI) m/z: $[M + H]^+$ Calcd for $C_{28}H_{30}N_3O_2S$ 472.2053; Found 472.2049.

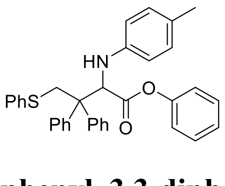

phenyl 3,3-diphenyl-4-(phenylthio)-2-(*p*-tolylamino)butanoate (43) According to the general procedure for the preparation of sulfanyl glycine derivative, phenyl *p*-

tolylglycinate **1g** (0.3 mmol, 72.3 mg), 1,1-diphenylethylene **2** (0.36 mmol, 64.8 mg), diphenyl disulfide **3** (0.45 mmol, 98.1 mg) were used. The product was isolated by a silica gel column chromatography using petroleum ether / EtOAc = 20:1 as eluent to give **43** as a yellowish solid (93.7 mg, 59% yield). m.p. 85 – 86 ℃. ^{1}H NMR (400 MHz, $CDCl_3$) δ 7.49 – 7.47 (m, 2H), 7.42 – 7.36 (m, 3H), 7.34 – 7.32 (m, 7H), 7.24 – 7.13 (m, 6H), 7.10 – 7.08 (m, 2H), 6.81 – 6.78 (m, 2H), 6.60 – 6.57 (m, 2H), 5.56 (s, 1H), 4.71 (br, 1H), 4.29 – 4.20 (m, 2H), 2.31 (s, 3H) ppm. ^{13}C NMR (101 MHz, $CDCl_3$) δ 171.2, 150.1, 143.9, 142.8, 142.2, 136.6, 130.5, 129.9, 129.3, 129.2, 129.1, 128.7, 128.3, 128.0, 127.8, 127.4, 127.2, 126.4, 125.8, 121.1, 114.2, 61.9, 53.7, 45.0, 20.4 ppm. HRMS (ESI) m/z: $[M + H]^+$ Calcd for $C_{35}H_{32}NO_2S$ 530.2148; Found 530.2155.

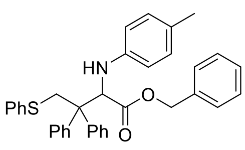

benzyl 3,3-diphenyl-4-(phenylthio)-2-(*p*-tolylamino)butanoate (44) According to the general procedure for the preparation of sulfanyl glycine derivative, benzyl *p*-tolylglycinate **1h** (0.3 mmol, 76.5 mg), 1,1-diphenylethylene **2** (0.36 mmol, 64.8 mg), diphenyl disulfide **3** (0.45 mmol, 98.1 mg) were used. The product was isolated by a silica gel column chromatography using petroleum ether / EtOAc = 20:1 as eluent to give **44** as a yellowish viscous oil (105.9 mg, 65% yield). ^{1}H NMR (400 MHz, $CDCl_3$) δ 7.33 – 7.27 (m, 7H), 7.26 – 7.21 (m, 6H), 7.21 – 7.15 (m, 5H), 7.02 – 6.98 (m, 4H), 6.67 – 6.65 (m, 2H), 5.37 (s, 1H), 4.87 (s, 2H), 4.11 – 4.03 (m, 2H), 2.28 (s, 3H) ppm. ^{13}C NMR (101 MHz, $CDCl_3$) δ 172.0, 144.1, 142.7, 142.0, 136.8, 135.1, 130.4, 129.8, 129.2, 129.2, 128.6, 128.5, 128.2, 128.1, 128.1, 127.8, 127.6, 127.2, 127.0, 126.2, 114.4, 66.7, 61.5, 54.1, 45.3, 20.4 ppm. HRMS (ESI) m/z: $[M + H]^+$ Calcd for $C_{36}H_{34}NO_2S$ 544.2305; Found 544.2297.

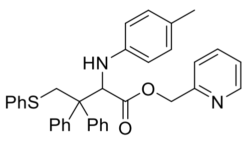

pyridin-2-ylmethyl 3,3-diphenyl-4-(phenylthio)-2-(*p*-tolylamino)butanoate (45) According to the general procedure for the preparation of sulfanyl glycine derivative, pyridin-2-ylmethyl *p*-tolylglycinate **1i** (0.3 mmol, 76.8 mg), 1,1-diphenylethylene **2** (0.36 mmol, 64.8 mg), diphenyl disulfide **3** (0.45 mmol, 98.1 mg) were used. The product was isolated by a silica gel column chromatography using petroleum ether / EtOAc = 5:1 as eluent to give **45** as a yellowish viscous oil (86.5 mg, 53% yield). ^{1}H NMR (400 MHz, $CDCl_3$) δ 8.48 (d, *J* = 6.8 Hz, 1H), 7.47 – 7.43 (m, 1H), 7.39 – 7.36 (m, 2H), 7.34 – 7.29 (m, 4H), 7.25 – 7.23 (m, 6H), 7.18 – 7.10 (m, 4H), 7.02 – 7.00 (m, 2H), 6.69 – 6.67 (m, 3H), 5.46 – 5.43 (m, 1H), 5.07 – 4.93 (m, 2H), 4.40 – 4.37 (m, 1H), 4.18 – 4.10 (m, 2H), 2.27 (s, 3H) ppm. ^{13}C NMR (101 MHz, $CDCl_3$) δ 171.8, 155.1, 148.6, 144.0, 142.6, 142.0, 136.7, 136.6, 130.3, 129.8, 129.2, 129.1, 128.6, 128.2, 127.9, 127.7, 127.3, 127.0, 126.2, 122.6, 121.5, 114.4, 66.8, 61.6, 54.0, 45.2, 20.4 ppm. HRMS (ESI) m/z: $[M + H]^+$ Calcd for $C_{35}H_{33}N_2O_2S$ 545.2257; Found 545.2251.

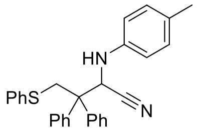

3,3-diphenyl-4-(phenylthio)-2-(*p*-tolylamino)butanenitrile (46) According to the general procedure for the preparation of sulfanyl glycine derivative, 2-(*p*-tolylamino)acetonitrile **1j** (0.3 mmol, 43.8 mg), 1,1-diphenylethylene **2** (0.36 mmol, 64.8 mg), diphenyl disulfide **3** (0.45 mmol, 98.1 mg) were used. The product was isolated by a silica gel column chromatography using petroleum ether / EtOAc = 10:1 as eluent to give **46** as a yellowish oil (53.4 mg, 41% yield). ^{1}H NMR (400 MHz, $CDCl_3$) δ 7.46 – 7.37 (m, 10H), 7.32 – 7.30 (m, 2H), 7.23 – 7.18 (m, 3H), 7.11 (d, *J* = 8.0 Hz, 2H), 6.73 (d, *J* = 8.4 Hz, 2H), 5.73 – 5.70 (m, 1H), 4.23 – 4.20 (m, 1H), 4.03 – 4.00 (m, 1H), 3.41 – 3.38 (m, 1H), 2.33 (s, 3H) ppm. ^{13}C NMR (101 MHz, $CDCl_3$) δ 141.9, 141.0, 140.5, 135.5, 130.8, 130.1, 129.9, 129.0, 128.8, 128.6, 128.3, 128.3, 127.9, 127.8, 126.7, 118.6, 115.5, 54.0, 51.6, 45.3, 20.4 ppm. HRMS (ESI) m/z: $[M + H]^+$ Calcd for $C_{29}H_{27}N_2S$ 435.1889; Found 435.1886.

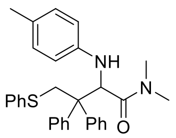

***N,N*-dimethyl-3,3-diphenyl-4-(phenylthio)-2-(*p*-tolylamino)butanamide (47)** According to the general procedure for the preparation of sulfanyl glycine derivative, *N,N*-dimethyl-2-(*p*-tolylamino)acetamide **1k** (0.3 mmol, 57.6 mg), 1,1-diphenylethylene **2** (0.36 mmol, 64.8 mg), diphenyl disulfide **3** (0.45 mmol, 98.1 mg) were used. The product was isolated by a silica gel column chromatography using petroleum ether / EtOAc = 10:1 as eluent to give **47** as a yellowish oil (96.5 mg, 67% yield). ^{1}H NMR (400 MHz, $CDCl_3$) δ 7.55 – 7.54 (m, 2H), 7.43 – 7.41 (m, 2H), 7.38 – 7.32 (m, 3H), 7.30 – 7.26 (m, 7H), 7.20 – 7.16 (m, 1H), 7.05 – 7.03 (m, 2H), 6.68 – 6.66 (m, 2H), 5.35 (s, 1H), 4.70 – 4.67 (m, 1H), 4.27 – 4.24 (m, 1H), 2.62 (s, 3H), 2.47 (s, 3H), 2.29 (s, 3H) ppm. ^{13}C NMR (101 MHz, $CDCl_3$) δ 171.7, 144.6, 144.0, 143.7, 137.3, 129.8, 129.4, 129.2, 129.0, 128.7, 127.8, 127.7, 127.5, 126.9, 126.8, 125.9, 114.0, 58.7, 53.3, 41.7, 36.9, 35.4, 20.3 ppm. HRMS (ESI) m/z: $[M + H]^+$ Calcd for $C_{31}H_{33}N_2OS$ 481.2308; Found 481.2306.

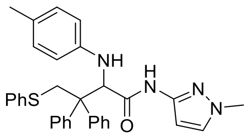

***N*-(1-methyl-1*H*-pyrazol-3-yl)-3,3-diphenyl-4-(phenylthio)-2-(*p*-tolylamino)butanamide (48)** According to the general procedure for the preparation of sulfanyl glycine derivative, *N*-(1-methyl-1*H*-pyrazol-3-yl)-2-(*p*-tolylamino)acetamide **1l** (0.3 mmol, 73.2 mg), 1,1-diphenylethylene **2** (0.36 mmol, 64.8 mg), diphenyl disulfide **3** (0.45 mmol, 98.1 mg) were used. The product was isolated by a silica gel column chromatography using petroleum ether / EtOAc = 3:1 as eluent to give **48** as a yellowish viscous oil (84.6 mg, 53% yield). ^{1}H NMR (400 MHz, $CDCl_3$) δ 8.54 (s, 1H), 7.39 – 7.37 (m, 2H), 7.34 – 7.27 (m, 3H), 7.23 – 7.04 (m, 13H), 6.69 (d, *J* = 2.4 Hz, 1H), 6.61 – 6.59 (m, 2H), 4.91 (s, 1H), 4.52 – 4.49 (m, 1H), 4.20 (s, 1H), 4.04 – 4.01 (m, 1H), 3.61 (s, 3H), 2.28 (s, 3H) ppm. ^{13}C NMR (101 MHz, $CDCl_3$) δ

169.1, 145.8, 143.7, 140.8, 140.6, 136.8, 130.2, 130.2, 129.8, 129.5, 128.7, 128.6, 128.2, 127.9, 127.8, 127.4, 127.0, 125.7, 113.9, 96.6, 63.7, 55.9, 46.0, 38.3, 20.2 ppm. HRMS (ESI) m/z: $[M + H]^+$ Calcd for $C_{33}H_{33}N_4OS$ 533.2370; Found 533.2379.

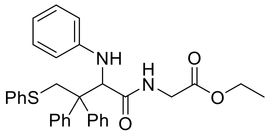

ethyl (3,3-diphenyl-2-(phenylamino)-4-(phenylthio)butanoyl)glycinate (49) According to the general procedure for the preparation of sulfanyl glycine derivative, ethyl phenylglycylglycinate **1m** (0.3 mmol, 70.8 mg), 1,1-diphenylethylene **2** (0.36 mmol, 64.8 mg), diphenyl disulfide **3** (0.45 mmol, 98.1 mg) were used. The product was isolated by a silica gel column chromatography using petroleum ether / EtOAc = 5:1 as eluent to give **49** as a yellowish viscous oil (83.3 mg, 53% yield). ^{1}H NMR (400 MHz, $CDCl_3$) δ 7.39 – 7.29 (m, 5H), 7.24 – 7.22 (m, 4H), 7.19 – 7.06 (m, 7H), 6.89 – 6.85 (m, 1H), 6.73 (t, *J* = 5.2 Hz, 1H), 6.68 – 6.65 (m, 2H), 4.86 (d, *J* = 3.6 Hz, 1H), 4.41 – 4.38 (m, 1H), 4.25 (d, *J* = 4.0 Hz, 1H), 4.11 – 4.03 (m, 3H), 3.88 – 3.82 (m, 1H), 3.53 – 3.47 (m, 1H), 1.19 (t, *J* = 7.2 Hz, 3H) ppm. ^{13}C NMR (101 MHz, $CDCl_3$) δ 171.4, 168.7, 146.3, 141.2, 141.0, 136.8, 130.1, 129.5, 129.3, 129.0, 128.4, 127.9, 127.9, 127.4, 127.2, 125.9, 119.5, 114.1, 63.3, 61.1, 55.6, 45.7, 41.1, 13.9 ppm. HRMS (ESI) m/z: $[M + H]^+$ Calcd for $C_{32}H_{33}N_2O_3S$ 525.2206; Found 525.2208.

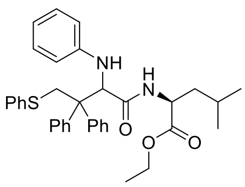

ethyl (3,3-diphenyl-2-(phenylamino)-4-(phenylthio)butanoyl)-*L*-leucinate (50) According to the general procedure for the preparation of sulfanyl glycine derivative, ethyl phenylglycyl-*L*-leucinate **1n** (0.3 mmol, 87.7 mg), 1,1-diphenylethylene **2** (0.36 mmol, 64.8 mg), diphenyl disulfide **3** (0.45 mmol, 98.1 mg) were used. The product was isolated by a silica gel column chromatography using petroleum ether / EtOAc = 5:1 as eluent to give **50** as a yellowish viscous oil (83.6 mg, 48% yield, 1.7:1 d.r.). d.r.

is determined by ^{1}H NMR. The data is written as observed. ^{1}H NMR (400 MHz, $CDCl_3$) δ 7.40 – 7.33 (m, 14H), 7.24 – 7.07 (m, 29H), 6.91 – 6.79 (m, 4H), 6.73 – 6.65 (m, 7H), 4.91 – 4.90 (m, 2H), 4.52 – 4.43 (m, 4H), 4.35 (s, 1H), 4.29 – 4.03 (m, 11H), 1.41 – 1.16 (m, 17H, including (1.26 (t, 5H, *J* = 7.2 Hz, major isomer), 1.18 (t, 3H, *J* = 7.2 Hz, minor isomer))), 1.07 – 0.93 (m, 3H), 0.89 – 0.76 (m, 16H) ppm. ^{13}C NMR (101 MHz, $CDCl_3$) δ 171.7, 171.4, 170.8, 170.4, 146.5, 145.9, 141.4, 141.3, 141.0, 140.9, 136.8, 136.5, 129.9, 129.5, 129.4, 129.3, 129.1, 129.1, 129.0, 128.9, 128.3, 128.2, 127.8, 127.8, 127.7, 127.7, 127.2, 127.2, 127.0, 126.9, 125.7, 125.6, 119.6, 119.2, 114.4, 113.7, 63.2, 63.1, 60.7, 55.4, 55.2, 50.7, 50.3, 45.6, 45.5, 41.4, 40.2, 24.2, 24.1, 22.3, 22.3, 21.8, 21.7, 13.9, 13.8 ppm. HRMS (ESI) m/z: $[M + H]^+$ Calcd for $C_{36}H_{41}N_2O_3S$ 581.2832; Found 581.2828.

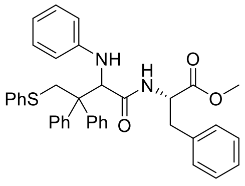

methyl (3,3-diphenyl-2-(phenylamino)-4-(phenylthio)butanoyl)-*L*-phenylalaninate (51) According to the general procedure for the preparation of sulfanyl glycine derivative, methyl phenylglycyl-*L*-phenylalaninate **1o** (0.3 mmol, 93.6 mg), 1,1-diphenylethylene **2** (0.36 mmol, 64.8 mg), diphenyl disulfide **3** (0.45 mmol, 98.1 mg) were used. The product was isolated by a silica gel column chromatography using petroleum ether / EtOAc = 5:1 as eluent to give **51** as a yellowish viscous oil (93.6 mg, 52% yield, 1.5:1 d.r.). d.r. is determined by ^{1}H NMR. The data is written as observed. ^{1}H NMR (400 MHz, $CDCl_3$) δ 7.43 – 7.30 (m, 18H), 7.25 – 7.13 (m, 25.5H), 7.08 – 7.05 (m, 3H), 6.98 – 6.81 (m, 11H), 6.68 (d, *J* = 8.0 Hz, 5H), 4.99 – 4.96 (m, 2.5H), 4.81 – 4.76 (m, 1.5H, major isomer), 4.67 – 4.62 (m, 1H, minor isomer), 4.59 – 4.56 (m, 1H, minor isomer), 4.49 – 4.4.6 (m, 1.5H, major isomer), 4.32 – 4.29 (m, 2.5H), 4.11 – 4.04 (m, 2.5H), 3.57 (s, 4.5H, major isomer), 3.53 (s, 3H, minor isomer), 2.92 (d, *J* = 6.0 Hz, 3H), 2.76 – 2.61 (m, 2H) ppm. ^{13}C NMR (101 MHz, $CDCl_3$) δ 171.0, 170.6, 170.6, 170.2, 146.1, 145.9, 141.5, 141.1, 141.1, 140.8, 136.7, 136.4, 135.3, 135.1,

130.0, 129.6, 129.3, 129.3, 129.1, 129.0, 128.8, 128.7, 128.6, 128.3, 128.3, 128.2, 128.0, 127.8, 127.7, 127.7, 127.3, 127.1, 126.9, 126.7, 126.5, 125.7, 125.7, 119.3, 119.3, 114.2, 113.7, 62.7, 62.5, 55.3, 55.0, 52.8, 52.7, 51.6, 45.8, 45.3, 38.2, 37.1 ppm. HRMS (ESI) m/z: $[M + H]^+$ Calcd for $C_{38}H_{37}N_2O_3S$ 601.2519; Found 601.2516.

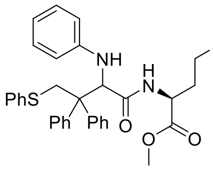

methyl (3,3-diphenyl-2-(phenylamino)-4-(phenylthio)butanoyl)-*L*-methioninate (52) According to the general procedure for the preparation of sulfanyl glycine derivative, methyl phenylglycyl-*L*-methioninate **1p** (0.3 mmol, 88.8 mg), 1,1-diphenylethylene **2** (0.36 mmol, 64.8 mg), diphenyl disulfide **3** (0.45 mmol, 98.1 mg) were used. The product was isolated by a silica gel column chromatography using petroleum ether / EtOAc = 5:1 as eluent to give **52** as a yellowish viscous oil (85.9 mg, 49% yield, 1.5:1 d.r.). d.r. is determined by ^{1}H NMR. The data is written as observed. ^{1}H NMR (400 MHz, $CDCl_3$) δ 7.38 – 7.31 (m, 12H), 7.26 – 7.19 (m, 11H), 7.15 – 7.06 (m, 17H), 6.98 – 6.96 (m, 1H, 6.89 – 6.83 (m, 3H), 6.80 – 6.78 (m, 1H), 6.68 – 6.62 (m, 5H), 4.86 – 4.84 (m, 2.5H), 4.46 – 4.38 (m, 4.5H), 4.35 (s, 1H), 4.29 (br, 1.5H, major isomer), 4.22 (br, 1H, minor isomer), 4.05 – 3.98 (m, 2.5H), 3.61 (s, 4.5H, major isomer), 3.55 (s, 3H, minor isomer), 2.16 – 2.02 (m, 4H), 2.00 – 1.98 (m, 2H), 1.96 (s, 3H, minor isomer), 1.94 – 1.89 (m, 2H), 1.88 (s, 4.5 H, major isomer), 1.74 – 1.65 (m, 3H), 1.60 – 1.51 (m, 1H) ppm. ^{13}C NMR (101 MHz, $CDCl_3$) δ 171.3, 171.2, 170.8, 170.8, 146.3, 146.0, 141.2, 141.1, 141.1, 140.9, 136.8, 136.7, 130.0, 129.8, 129.5, 129.5, 129.3, 129.2, 129.0, 128.9, 128.4, 128.4, 128.0, 127.9, 127.8, 127.5, 127.3, 127.1, 125.8, 125.8, 119.7, 119.5, 114.4, 113.8, 63.4, 63.2, 55.5, 55.4, 52.1, 52.0, 51.2, 51.1, 45.8, 31.5, 30.6, 29.4, 29.2, 15.1, 15.0 ppm. HRMS (ESI) m/z: $[M + H]^+$ Calcd for $C_{34}H_{37}N_2O_3S_2$ 585.2240; Found 585.2244.

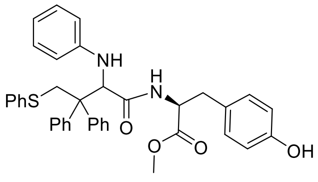

methyl (3,3-diphenyl-2-(phenylamino)-4-(phenylthio)butanoyl)-*L*-tyrosinate (53)

According to the general procedure for the preparation of sulfanyl glycine derivative, methyl phenylglycyl-*L*-tyrosinate **1q** (0.3 mmol, 98.4 mg), 1,1-diphenylethylene **2** (0.36 mmol, 64.8 mg), diphenyl disulfide **3** (0.45 mmol, 98.1 mg) were used. The product was isolated by a silica gel column chromatography using petroleum ether / EtOAc = 2:1 as eluent to give **53** as a yellowish viscous oil (86.9 mg, 47% yield, 1.2:1 d.r.). d.r. is determined by ^{1}H NMR. The data is written as observed. ^{1}H NMR (400 MHz, $CDCl_3$) δ 7.37 – 7.32 (m, 6H), 7.24 – 7.05 (m, 31.3H), 6.90 – 6.79 (m, 5H), 6.59 – 6.53 (m, 10.5H), 6.40 – 6.38 (m, 3H), 6.17 (s, 1H), 4.88 – 4.86 (m, 2H), 4.68 – 4.63 (m, 1.2H), 4.51 – 4.44 (m, 2H), 4.35 – 4.32 (m, 1.2H), 4.23 – 4.19 (m, 2H), 4.01 – 3.95 (m, 2.2H), 3.53 (s, 3.6H, major isomer), 3.47 (s, 3H, minor isomer), 2.73 – 2.72 (m, 2H), 2.58 – 2.43 (m, 2.2H), 1.92 (br,1H) ppm. ^{13}C NMR (101 MHz, $CDCl_3$) δ 171.4, 171.2, 171.1, 170.8, 155.1, 154.8, 146.2, 146.1, 141.6, 141.2, 140.9, 136.8, 136.5, 130.2, 130.1, 129.8, 129.7, 129.5, 129.5, 129.2, 128.9, 128.5, 128.5, 128.0, 128.0, 127.9, 127.9, 127.6, 127.3, 127.2, 126.8, 126.6, 125.9, 125.9, 119.6, 119.5, 115.4, 115.2, 114.4, 113.9, 63.0, 62.8, 55.5, 55.3, 53.3, 53.1, 52.0, 52.0, 45.9, 45.5, 37.7, 36.5 ppm. HRMS (ESI) m/z: $[M + H]^+$ Calcd for $C_{38}H_{37}N_2O_4S$ 617.2469; Found 617.2465.

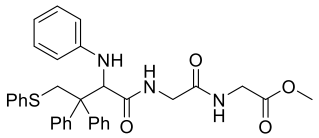

methyl (3,3-diphenyl-2-(phenylamino)-4-(phenylthio)butanoyl)glycylglycinate (54)

According to the general procedure for the preparation of sulfanyl glycine derivative, methyl phenylglycylglycylglycinate **1r** (0.3 mmol, 83.7 mg), 1,1-diphenylethylene **2** (0.36 mmol, 64.8 mg), diphenyl disulfide **3** (0.45 mmol, 98.1 mg) were used. The product was isolated by a silica gel column chromatography using petroleum ether / EtOAc = 2:1 as eluent to give **54** as a yellowish viscous oil (71.5 mg, 42% yield). ^{1}H NMR (400 MHz, $CDCl_3$) δ 7.37 – 7.31 (m, 5H), 7.24 – 7.20 (m, 4H), 7.16 – 7.05 (m, 7H), 6.86 – 6.82 (m, 1H), 6.72 (t, J = 6.0 Hz, 1H), 6.65 – 6.63 (m, 2H), 6.16 (t, J = 6.0 Hz, 1H), 4.91 (s, 1H), 4.33 – 4.30 (m, 2H), 4.02 – 3.98 (m, 1H), 3.82 – 3.76 (m, 3H), 3.69 (s, 3H), 3.42 – 3.36 (m, 1H) ppm. ^{13}C NMR (101 MHz, $CDCl_3$) δ 172.2, 169.7,

168.6, 146.0, 141.1, 140.9, 136.6, 130.2, 129.6, 129.5, 129.0, 128.5, 128.0, 127.6, 127.3, 126.0, 119.7, 113.9, 63.2, 55.4, 52.2, 45.8, 42.8, 40.7 ppm. HRMS (ESI) m/z: $[M + H]^+$ Calcd for $C_{33}H_{34}N_3O_4S$ 568.2265; Found 568.2264.

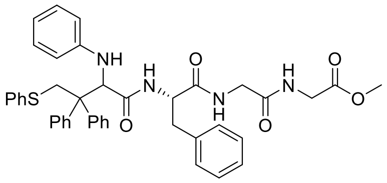

methyl (3,3-diphenyl-2-(phenylamino)-4-(phenylthio)butanoyl)-*L*-phenylalanylglycylglycinate (55) According to the general procedure for the preparation of sulfanyl glycine derivative, methyl phenylglycyl-*L*-phenylalanylglycylglycinate **1s** (0.3 mmol, 127.9 mg), 1,1-diphenylethylene **2** (0.36 mmol, 64.8 mg), diphenyl disulfide **3** (0.45 mmol, 98.1 mg) were used. The product was isolated by a silica gel column chromatography using petroleum ether / MeOH = 20:1 as eluent to give **55** as a yellowish solid (92.1 mg, 43% yield, 2:1 d.r.). d.r. is determined by ^{1}H NMR. The data is written as observed. m.p. 89 – 90 ℃. ^{1}H NMR (400 MHz, $CDCl_3$) δ 7.45 – 7.41 (m, 4H), 7.39 – 7.31 (m, 11H), 7.23 – 7.18 (m, 6H), 7.17 – 7.12 (m, 17H), 7.11 – 7.06 (m, 13H), 7.05 – 7.02 (m, 5H), 7.00 – 6.97 (m, 3H), 6.89 – 6.85 (m, 4H), 6.73 – 6.68 (m, 5H), 6.62 – 6.56 (m, 3H), 6.49 – 6.47 (m, 4H), 5.92 (t, *J* = 6.4 Hz, 1H, minor isomer), 5.65 (t, *J* = 6.4 Hz, 2H, major isomer), 5.03 (d, *J* = 4.8 Hz, 1H, minor isomer), 4.84 (d, *J* = 3.2 Hz, 2H, major isomer), 4.41 – 4.38 (m, 1H), 4.31 – 4.25 (m, 6H), 4.19 – 4.13 (m, 2H), 4.02 – 3.78 (m, 13H), 3.69 (s, 3H, minor isomer), 3.65 (s, 6H, major isomer), 3.59 – 3.53 (m, 1H), 2.80 – 2.67 (m, 5H), 2.41 – 2.36 (m, 1H), 2,09 (br, 1H) ppm. ^{13}C NMR (101 MHz, $CDCl_3$) δ 172.9, 172.3, 170.8, 170.6, 170.0, 169.9, 169.2, 169.1, 146.0, 145.4, 141.6, 141.4, 141.2, 141.1, 136.6, 136.5, 135.2, 135.1, 130.3, 129.8, 129.6, 129.5, 129.4, 129.4, 129.1, 128.7, 128.6, 128.5, 128.5, 128.5, 128.1, 128.1, 128.0, 128.0, 127.8, 127.6, 127.3, 127.1, 127.0, 126.1, 126.0, 119.9, 113.9, 113.8, 63.6, 61.6, 55.3, 55.1, 54.8, 54.7, 52.1, 46.1, 45.6, 42.8, 42.5, 40.9, 40.8, 37.1, 36.2 ppm. HRMS (ESI) m/z: $[M + H]^+$ Calcd for $C_{42}H_{43}N_4O_5S$ 715.2949; Found 715.2956.

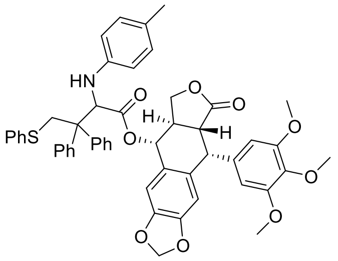

(5*R*,5a*R*,8a*R*,9*R*)-8-oxo-9-(3,4,5-trimethoxyphenyl)-5,5a,6,8,8a,9-hexahyd.r.ofuro[3',4':6,7]naphtho[2,3-*d*][1,3]dioxol-5-yl 3,3-diphenyl-4-(phenylthio)-2-(*p*-tolylamino)butanoate (56) According to the general procedure for the preparation of sulfanyl glycine derivative, (5*R*,5a*R*,8a*R*,9*R*)-9-(4-hydroxy-3,5-dimethoxyphenyl)-8-oxo-5,5a,6,8,8a,9-hexahydrofuro[3',4':6,7]naphtho[2,3-*d*][1,3]dioxol-5-yl phenylglycinate **1t** (0.3 mmol, 160.0 mg), 1,1-diphenylethylene **2** (0.36 mmol, 64.8 mg), diphenyl disulfide **3** (0.45 mmol, 98.1 mg) were used. The product was isolated by a silica gel column chromatography using petroleum ether / EtOAc = 1:1 as eluent to give **56** as a yellowish solid (124.8 mg, 49% yield, 1:1 d.r.). d.r. is determined by ^{1}H NMR. The data is written as observed. m.p. 114 – 115 ℃. ^{1}H NMR (400 MHz, $CDCl_3$) δ 7.43 – 7.39 (m, 4H), 7.33 – 7.26 (m, 14H), 7.24 – 7.10 (m, 14H), 7.06 – 7.04 (m, 2H), 6.92 – 6.90 (m, 2H), 6.75 – 6.73 (m, 2H), 6.64 – 6.62 (m, 2H), 6.45 (d, *J* = 2.4 Hz, 2H), 6.32 – 6.31 (m, 4H), 6.04 – 5.91 (m, 6H), 5.73 (s, 1H), 5.60 (d, *J* = 9.2 Hz, 1H, isomer-1), 5.54 (d, *J* = 9.6 Hz, 1H, isomer-2), 5.43 (d, *J* = 11.6 Hz, 1H, isomer-1), 5.35 (d, *J* = 10.8 Hz, 1H, isomer-2), 4.76 (d, *J* = 10.8 Hz, 1H), 4.49 (d, *J* = 4.4 Hz, 2H), 4.37 – 4.34 (m, 1H), 4.29 (d, *J* = 11.6 Hz, 1H), 4.19 – 4.10 (m, 3H), 3.89 (s, 3H), 3.85 (s, 3H), 3.77 (s, 6H), 3.74 (s, 6H), 3.61 – 3.57 (m, 1H), 2.74 – 2.68 (m, 2H), 2.27 (s, 3H), 2.17 (s, 3H) ppm. ^{13}C NMR (101 MHz, $CDCl_3$) δ 173.5, 173.5, 173.2, 172.8, 152.5, 147.9, 147.3, 147.2, 143.5, 143.4, 142.9, 142.3, 142.2, 142.2, 137.0, 136.3, 136.3, 134.8, 134.8, 132.0, 130.2, 130.2, 130.0, 129.8, 129.3, 129.2, 129.2, 129.0, 128.9, 128.8, 128.7, 128.6, 128.0, 127.9, 127.9, 127.6, 127.5, 127.5, 127.3, 127.2, 126.4, 126.3, 115.0, 114.2, 109.3, 109.2, 108.0, 107.9, 107.4, 107.4, 101.4, 101.4, 74.4, 74.2, 71.3, 71.1, 62.6, 62.4, 60.6, 60.6, 56.1, 56.1, 53.6, 53.0, 45.4, 45.3, 45.0, 44.3, 43.3, 38.4, 38.1, 20.4, 20.3 ppm. HRMS (ESI) m/z: $[M + H]^+$ Calcd for $C_{51}H_{48}NO_9S$ 850.3044; Found 850.3045.

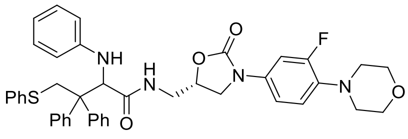

***N*-(((*S*)-3-(3-fluoro-4-morpholinophenyl)-2-oxooxazolidin-5-yl)methyl)-3,3-diphenyl-2-(phenylamino)-4-(phenylthio)butanamide (57)** According to the general procedure for the preparation of sulfanyl glycine derivative, (*S*)-*N*-((3-(3-fluoro-4-morpholinophenyl)-2-oxooxazolidin-5-yl)methyl)-2-(phenylamino)acetamide **1u** (0.3 mmol, 128.5 mg), 1,1-diphenylethylene **2** (0.36 mmol, 64.8 mg), diphenyl disulfide **3** (0.45 mmol, 98.1 mg) were used. The product was isolated by a silica gel column chromatography using petroleum ether / EtOAc = 2:1 as eluent to give **57** as a yellowish solid (92.4 mg, 43% yield, 1:1 d.r.). d.r. is determined by ^{19}F NMR. The data is written as observed. m.p. 110 – 111 ℃. ^{1}H NMR (400 MHz, $CDCl_3$) δ 7.39 – 7.29 (m, 5H), 7.22 – 7.06 (m, 11H), 7.00 – 6.87 (m, 3H), 6.79 – 6.58 (m, 3H), 6.52 – 6.50 (m, 1H), 4.83 (dd, *J* = 15.2, 4.0 Hz, 1H), 4.35 – 4.20 (m, 3H), 4.04 – 3.98 (m, 1H), 3.88 – 3.85 (m, 4H), 3.70 (dt, *J* = 19.2, 9.6 Hz, 1H), 3.42 – 3.32 (m, 2H), 3.08 – 3.04 (m, 4H) ppm. ^{13}C NMR (101 MHz, $CDCl_3$) δ 172.3, 172.2, 155.3 (d, $J_{C\text{-}F}$ = 247.2 Hz), 155.3 (d, $J_{C\text{-}F}$ = 247.1 Hz), 153.8, 153.5, 146.0, 145.6, 141.2, 141.1, 140.9, 140.7, 136.7, 136.7, 136.2, 136.1, 136.0, 133.0 (d, $J_{C\text{-}F}$ = 10.4 Hz), 133.0 (d, $J_{C\text{-}F}$ = 10.3 Hz), 130.0, 130.0, 129.5, 129.4, 129.4, 129.3, 128.9, 128.8, 128.5, 128.4, 128.0, 128.0, 127.9, 127.6, 127.6, 127.3, 127.3, 126.0, 125.9, 119.7, 119.7, 118.6 (d, $J_{C\text{-}F}$ = 12.7 Hz), 118.5 (d, $J_{C\text{-}F}$ = 12.5 Hz), 113.9, 113.6, 113.6, 113.4, 113.4, 107.1 (d, $J_{C\text{-}F}$ = 26.4 Hz), 107.1 (d, $J_{C\text{-}F}$ = 26.6 Hz), 71.1, 71.0, 66.8, 63.5, 63.3, 55.5, 55.2, 50.9, 50.9, 50.9, 47.6, 47.1, 45.9, 45.7, 41.4, 40.7 ppm. ^{19}F NMR (376 MHz, $CDCl_3$) δ –120.3 (isomer-1), –120.4 (isomer-2) ppm. HRMS (ESI) m/z: $[M + H]^+$ Calcd for $C_{42}H_{42}FN_4O_4S$ 717.2905; Found 717.2901.

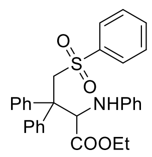

ethyl 3,3-diphenyl-2-(phenylamino)-4-(phenylsulfonyl)butanoate (58) According to the general procedure for the preparation of sulfonyl glycine derivative, *N*-phenylglycine ethyl ester **1** (0.3 mmol, 53.7 mg), 1,1-diphenylethylene **2** (0.36 mmol, 64.8 mg), *S*-phenyl benzenesulfonothioate **f1** (0.45 mmol, 112.5 mg) were used. The product was isolated by a silica gel column chromatography using petroleum ether / EtOAc = 5:1 as eluent to give **58** as a white solid (110.8 mg, 74% yield). m.p. 95 – 96 ℃. ^{1}H NMR (400 MHz, $CDCl_3$) δ 7.55 – 7.45 (m, 3H), 7.34 – 7.22 (m, 9H), 7.21 – 7.15 (m, 5H), 6.91 – 6.83 (m, 3H), 6.13 (s, 1H), 4.63 – 4.51 (m, 2H), 4.21 (s, 1H), 4.06 – 3.91 (m, 2H), 1.07 – 1.03 (m, 3H) ppm. ^{13}C NMR (101 MHz, $CDCl_3$) δ 171.5, 146.2,

141.2, 140.6, 140.5, 132.5, 129.4, 129.1, 128.9, 128.6, 127.6, 127.5, 127.4, 127.1, 126.9, 119.0, 114.4, 64.0, 60.9, 59.1, 52.5, 13.6 ppm. HRMS (ESI) m/z: $[M + H]^+$ Calcd for $C_{30}H_{30}NO_4S$ 500.1890; Found 500.1889.

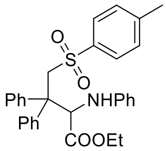

ethyl 3,3-diphenyl-2-(phenylamino)-4-tosylbutanoate (59) According to the general procedure for the preparation of sulfonyl glycine derivative, *N*-phenylglycine ethyl ester **1** (0.3 mmol, 53.7 mg), 1,1-diphenylethylene **2** (0.36 mmol, 64.8 mg), *S*-phenyl 4-methylbenzenesulfonothioate **f2** (0.45 mmol, 118.8 mg) were used. The product was isolated by a silica gel column chromatography using petroleum ether / EtOAc = 5:1 as eluent to give **59** as a yellowish viscous oil (120.1 mg, 78% yield). ^1H NMR (400 MHz, $CDCl_3$) δ 7.46 – 7.44 (m, 2H), 7.30 – 7.26 (m, 4H), 7.25 – 7.13 (m, 10H), 6.88 – 6.81 (m, 3H), 6.07 (d, *J* = 11.6 Hz, 1H), 4.55 – 4.44 (m, 2H), 4.22 (d, *J* = 11.6 Hz, 1H), 4.04 – 3.89 (m, 2H), 2.38 (s, 3H), 1.03 (t, *J* = 7.2 Hz, 3H) ppm. ^{13}C NMR (101 MHz, $CDCl_3$) δ 171.7, 146.4, 143.5, 140.9, 140.8, 138.7, 129.5, 129.3, 129.2, 129.0, 127.7, 127.5, 127.5, 127.2, 119.0, 114.5, 64.1, 61.0, 59.3, 52.6, 21.3, 13.7 ppm. HRMS (ESI) m/z: $[M + H]^+$ Calcd for $C_{31}H_{32}NO_4S$ 514.2047; Found 514.2041.

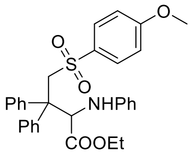

ethyl 4-((4-methoxyphenyl)sulfonyl)-3,3-diphenyl-2-(phenylamino)butanoate (60) According to the general procedure for the preparation of sulfonyl glycine derivative, *N*-phenylglycine ethyl ester **1** (0.3 mmol, 53.7 mg), 1,1-diphenylethylene **2** (0.36 mmol, 64.8 mg), *S*-phenyl 4-methoxybenzenesulfonothioate **f3** (0.45 mmol, 126.0 mg) were used. The product was isolated by a silica gel column chromatography using petroleum ether / EtOAc = 5:1 as eluent to give **60** as a yellowish solid (85.7 mg, 54% yield). m.p. 153 – 154 ℃. ^1H NMR (400 MHz, $CDCl_3$) δ 7.47 – 7.43 (m, 2H), 7.28 – 7.26 (m, 3H), 7.24 – 7.11 (m, 9H), 6.85 – 6.77 (m, 5H), 6.03 (s, 1H), 4.51 – 4.39 (m, 2H), 4.02 – 3.87

(m, 2H), 3.82 (s, 3H), 1.01 (t, J = 7.2 Hz, 3H) ppm. ^{13}C NMR (101 MHz, $CDCl_3$) δ 171.8, 162.9, 146.4, 141.0, 140.9, 133.4, 129.6, 129.4, 129.3, 129.1, 127.8, 127.6, 127.6, 127.3, 119.1, 114.6, 114.0, 64.3, 61.1, 59.3, 55.6, 52.7, 13.8 ppm. HRMS (ESI) m/z: $[M + H]^+$ Calcd for $C_{31}H_{32}NO_5S$ 530.1996; Found 530.1992.

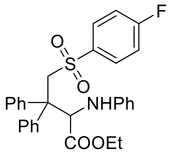

ethyl 4-((4-fluorophenyl)sulfonyl)-3,3-diphenyl-2-(phenylamino)butanoate (61) According to the general procedure for the preparation of sulfonyl glycine derivative, *N*-phenylglycine ethyl ester **1** (0.3 mmol, 53.7 mg), 1,1-diphenylethylene **2** (0.36 mmol, 64.8 mg), *S*-phenyl 4-fluorobenzenesulfonothioate **f4** (0.45 mmol, 120.6 mg) were used. The product was isolated by a silica gel column chromatography using petroleum ether / EtOAc = 5:1 as eluent to give **61** as a yellowish viscous oil (94.6 mg, 61% yield). ^{1}H NMR (400 MHz, $CDCl_3$) δ 7.51 – 7.48 (m, 2H), 7.29 – 7.12 (m, 12H), 6.99 – 6.95 (m, 2H), 6.88 – 6.82 (m, 3H), 6.08 (d, J = 11.6 Hz, 1H), 4.62 – 4.46 (m, 2H), 4.12 (d, J = 12.0 Hz, 1H), 4.06 – 3.90 (m, 2H), 1.04 (t, J = 7.2 Hz, 3H) ppm. ^{13}C NMR (101 MHz, $CDCl_3$) δ 171.6, 164.9 (d, $J_{C\text{-}F}$ = 256.2 Hz), 146.3, 140.6 (d, $J_{C\text{-}F}$ = 7.4 Hz), 137.4 (d, $J_{C\text{-}F}$ = 3.0 Hz), 130.0, 129.9, 129.5, 129.3, 129.0, 127.8, 127.7, 127.5, 127.3, 119.1, 115.8 (d, $J_{C\text{-}F}$ = 22.8 Hz), 114.6, 64.4, 61.1, 59.1, 52.5, 13.7 ppm. ^{19}F NMR (376 MHz, $CDCl_3$) δ -104.8 ppm. HRMS (ESI) m/z: $[M + H]^+$ Calcd for $C_{30}H_{29}FNO_4S$ 518.1796; Found 518.1805.

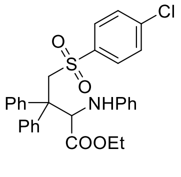

ethyl 4-((4-chlorophenyl)sulfonyl)-3,3-diphenyl-2-(phenylamino)butanoate (62) According to the general procedure for the preparation of sulfonyl glycine derivative, *N*-phenylglycine ethyl ester **1** (0.3 mmol, 53.7 mg), 1,1-diphenylethylene **2** (0.36 mmol, 64.8 mg), *S*-phenyl 4-chlorobenzenesulfonothioate **f5** (0.45 mmol, 127.8 mg) were used. The product was isolated by a silica gel column chromatography using petroleum ether / EtOAc = 5:1 as eluent to give **62** as a yellowish viscous oil (92.8 mg, 58% yield). ^{1}H

NMR (400 MHz, $CDCl_3$) δ 7.43 – 7.40 (m, 2H), 7.29 – 7.21 (m, 10H), 7.18 – 7.11 (m, 4H), 6.87 – 6.82 (m, 3H), 6.06 (d, *J* = 12.0 Hz, 1H), 4.61 – 4.46 (m, 2H), 4.10 (d, *J* = 12.0 Hz, 1H), 4.06 – 3.90 (m, 2H), 1.03 (t, *J* = 7.2 Hz, 3H) ppm. ^{13}C NMR (101 MHz, $CDCl_3$) δ 171.6, 146.2, 140.6, 140.5, 139.8, 139.1, 129.5, 129.3, 129.0, 128.9, 128.6, 127.8, 127.7, 127.5, 127.3, 119.2, 114.6, 64.4, 61.1, 59.1, 52.5, 13.7 ppm. HRMS (ESI) m/z: $[M + H]^+$ Calcd for $C_{30}H_{29}ClNO_4S$ 534.1500; Found 534.1497.

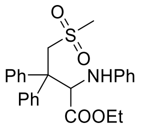

ethyl 4-(methylsulfonyl)-3,3-diphenyl-2-(phenylamino)butanoate (63) According to the general procedure for the preparation of sulfonyl glycine derivative, *N*-phenylglycine ethyl ester **1** (0.3 mmol, 53.7 mg), 1,1-diphenylethylene **2** (0.36 mmol, 64.8 mg), *S*-phenyl methanesulfonothioate **f6** (0.45 mmol, 84.6 mg) were used. The product was isolated by a silica gel column chromatography using petroleum ether / EtOAc = 5:1 as eluent to give **63** as a yellowish viscous oil (89.2 mg, 68% yield). ^{1}H NMR (400 MHz, $CDCl_3$) δ 7.39 – 7.31 (m, 8H), 7.28 – 7.26 (m, 2H), 7.21 – 7.17 (m, 2H), 6.81 – 6.77 (m, 3H), 5.97 (s, 1H), 4.34 – 4.18 (m, 2H), 4.05 – 3.86 (m, 3H), 1.87 (s, 3H), 1.02 (t, *J* = 7.2 Hz, 3H) ppm. ^{13}C NMR (101 MHz, $CDCl_3$) δ 171.4, 146.3, 141.0, 140.7, 129.7, 129.3, 129.1, 128.2, 128.0, 128.0, 127.6, 119.3, 114.8, 63.6, 61.2, 58.7, 52.7, 42.5, 13.7 ppm. HRMS (ESI) m/z: $[M + H]^+$ Calcd for $C_{25}H_{28}NO_4S$ 438.1734; Found 438.1730.

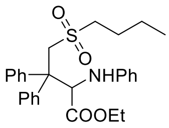

ethyl 4-(*tert*-butylsulfonyl)-3,3-diphenyl-2-(phenylamino)butanoate (64) According to the general procedure for the preparation of sulfonyl glycine derivative, *N*-phenylglycine ethyl ester **1** (0.3 mmol, 53.7 mg), 1,1-diphenylethylene **2** (0.36 mmol, 64.8 mg), *S*-phenyl butane-1-sulfonothioate **f7** (0.45 mmol, 103.5 mg) were used. The

product was isolated by a silica gel column chromatography using petroleum ether / EtOAc = 5:1 as eluent to give **64** as a yellowish viscous oil (96.3 mg, 67% yield). ^{1}H NMR (400 MHz, $CDCl_3$) δ 7.40 – 7.37 (m, 5H), 7.34 – 7.27 (m, 5H), 7.21 – 7.17 (m, 2H), 6.84 – 6.77 (m, 3H), 6.05 (br, 1H), 4.27 – 4.17 (m, 2H), 4.04 – 3.87 (m, 3H), 1.81 – 1.68 (m, 2H), 1.58 – 1.46 (m, 2H), 1.19 – 1.09 (m, 2H), 1.03 (t, *J* = 7.2 Hz, 3H), 0.78 (t, *J* = 7.2 Hz, 3H) ppm. ^{13}C NMR (101 MHz, $CDCl_3$) δ 171.4, 146.3, 141.1, 140.7, 129.6, 129.1, 129.0, 127.9, 127.8, 127.4, 119.1, 114.7, 61.0, 60.8, 58.8, 53.9, 52.6, 23.7, 21.2, 13.6, 13.2 ppm. HRMS (ESI) m/z: $[M + H]^+$ Calcd for $C_{28}H_{34}NO_4S$ 480.2203; Found 480.2208.

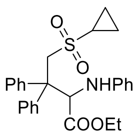

ethyl 4-(cyclopropylsulfonyl)-3,3-diphenyl-2-(phenylamino)butanoate (65)

According to the general procedure for the preparation of sulfonyl glycine derivative, *N*-phenylglycine ethyl ester **1** (0.3 mmol, 53.7 mg), 1,1-diphenylethylene **2** (0.36 mmol, 64.8 mg), *S*-phenyl cyclopropanesulfonothioate **f8** (0.45 mmol, 96.3 mg) were used. The product was isolated by a silica gel column chromatography using petroleum ether / EtOAc = 5:1 as eluent to give **65** as a yellowish viscous oil (101.4 mg, 73% yield). ^{1}H NMR (400 MHz, $CDCl_3$) δ 7.40 – 7.29 (m, 10H), 7.22 – 7.18 (m, 2H), 6.84 – 6.78 (m, 3H), 6.03 (d, *J* = 12.0 Hz, 1H), 4.36 – 4.24 (m, 2H), 4.09 – 3.87 (m, 3H), 1.13 – 0.97 (m, 5H), 0.87 – 0.80 (m, 1H), 0.66 – 0.56 (m, 2H) ppm. ^{13}C NMR (101 MHz, $CDCl_3$) δ 171.5, 146.4, 141.4, 141.0, 129.8, 129.2, 129.2, 128.0, 127.8, 127.8, 127.5, 119.2, 114.7, 62.4, 61.1, 58.8, 52.5, 31.2, 13.7, 5.7, 5.4 ppm. HRMS (ESI) m/z: $[M + H]^+$ Calcd for $C_{27}H_{30}NO_4S$ 464.1890; Found 464.1891.

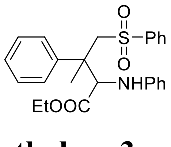

ethyl 3-methyl-3-phenyl-2-(phenylamino)-4-(phenylsulfonyl)butanoate (66)

According to the general procedure for the preparation of sulfonyl glycine derivative,

N-phenylglycine ethyl ester **1** (0.3 mmol, 53.7 mg), prop-1-en-2-ylbenzene **2e** (0.36 mmol, 42.5 mg), *S*-phenyl benzenesulfonothioate **f1** (0.45 mmol, 112.5 mg) were used. The product was isolated by a silica gel column chromatography using petroleum ether / EtOAc = 5:1 as eluent to give **66** as a yellowish solid (82.6 mg, 63% yield, 1.5:1 d.r.). d.r. is determined by ^{1}H NMR. The data is written as observed. m.p. 133 – 134 ℃. ^{1}H NMR (400 MHz, $CDCl_3$) δ 7.72 – 7.70 (m, 2H), 7.66 – 7.63 (m, 3H), 7.57 – 7.48 (m, 2.5H), 7.43 – 7.34 (m, 5H), 7.25 – 7.21 (m, 8H), 7.20 – 7.15 (m, 9.5H), 6.81 – 6.77 (m, 2.5H), 6.73 – 6.67 (m, 5H), 4.65 (s, 1H), 4.39 (s, 1.5H), 4.15 – 4.05 (m, 3.5H), 4.03 – 3.97 (m, 2H), 3.91 – 3.82 (m, 3H), 3.74 (q, *J* = 7.2 Hz, 3H), 2.03 (s, 3H, minor isomer), 1.94 (s, 4.5H, major isomer), 1.10 (t, *J* = 7.2 Hz, 3H, minor isomer), 0.78 (t, *J* = 7.2 Hz, 4.5H, major isomer) ppm. ^{13}C NMR (101 MHz, $CDCl_3$) δ 171.4, 171.1, 146.8, 146.8, 141.4, 141.1, 139.8, 139.6, 133.2, 133.0, 129.3, 129.2, 129.0, 128.9, 128.2, 128.1, 127.5, 127.5, 127.4, 127.2, 126.7, 126.5, 119.3, 114.8, 114.5, 65.6, 65.2, 63.9, 63.0, 61.1, 60.8, 44.8, 44.6, 21.2, 18.4, 13.9, 13.4 ppm. HRMS (ESI) m/z: $[M + H]^+$ Calcd for $C_{25}H_{28}NO_4S$ 438.1734; Found 438.1731.

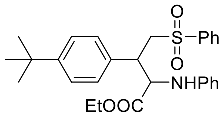

ethyl 3-(4-(tert-butyl)phenyl)-2-(phenylamino)-4-(phenylsulfonyl)butanoate (67)

According to the general procedure for the preparation of sulfonyl glycine derivative, *N*-phenylglycine ethyl ester **1** (0.3 mmol, 53.7 mg), 1-(*tert*-butyl)-4-vinylbenzene **2l** (0.36 mmol, 57.6 mg), *S*-phenyl benzenesulfonothioate **f1** (0.45 mmol, 112.5 mg) were used. The product was isolated by a silica gel column chromatography using petroleum ether / EtOAc = 5:1 as eluent to give **67** as a yellowish viscous oil (73.3 mg, 51% yield, 1.1:1 d.r.). d.r. is determined by ^{1}H NMR of the crude product. ^{1}H NMR (400 MHz, $CDCl_3$) δ major isomer 7.85 – 7.82 (m, 2H), 7.60 – 7.56 (m, 1H), 7.48 – 7.44 (m, 2H), 7.25 – 7.18 (m, 4H), 6.95 – 6.93 (m, 2H), 6.83 – 6.79 (m, 3H), 4.86 – 4.84 (m, 1H), 4.11 (q, *J* = 7.2 Hz, 2H), 4.04 – 3.94 (m, 2H), 3.88 – 3.85 (m, 1H), 3.52 – 3.48 (m, 1H), 1.29 (s, 9H), 1.23 (t, *J* = 7.2 Hz, 3H) ppm. ^{13}C NMR (101 MHz, $CDCl_3$) δ major isomer 171.6, 150.8, 147.3, 139.3, 133.7, 133.6, 129.3, 129.1, 128.0, 127.7, 125.5, 119.3, 114.7, 61.3, 60.2, 57.6, 41.8, 34.4, 31.2, 14.1 ppm. HRMS (ESI) m/z: $[M + H]^+$ Calcd for $C_{28}H_{34}NO_4S$ 480.2203; Found 480.2212. ^{1}H NMR (400 MHz, $CDCl_3$) δ minor isomer

7.69 – 7.67 (m, 2H), 7.51 – 7.46 (m, 1H), 7.37 – 7.33 (m, 2H), 7.19 – 7.13 (m, 4H), 6.91 – 6.89 (m, 2H), 6.79 – 6.75 (m, 1H), 6.64 – 6.62 (m, 2H), 4.35 – 4.24 (m, 2H), 4.05 – 4.00 (m, 1H), 3.92 – 3.84 (m, 2H), 3.69 – 3.58 (m, 2H), 1.25 (s, 9H), 0.90 (t, J = 7.2 Hz, 3H) ppm. ^{13}C NMR (101 MHz, $CDCl_3$) δ minor isomer 171.6, 150.6, 146.1, 139.4, 133.8, 133.3, 129.4, 128.9, 127.9, 127.9, 125.4, 119.0, 114.0, 61.4, 61.1, 58.3, 43.3, 34.4, 31.2, 13.7 ppm. HRMS (ESI) m/z: $[M + H]^+$ Calcd for $C_{28}H_{34}NO_4S$ 480.2203; Found 480.2207.

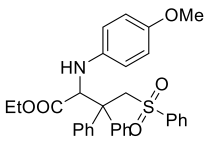

ethyl 2-((4-methoxyphenyl)amino)-3,3-diphenyl-4-(phenylsulfonyl)butanoate (68)

According to the general procedure for the preparation of sulfonyl glycine derivative, ethyl (4-methoxyphenyl)glycinate **1b** (0.3 mmol, 62.7 mg), 1,1-diphenylethylene **2** (0.36 mmol, 64.8 mg), *S*-phenyl benzenesulfonothioate **f1** (0.45 mmol, 112.5 mg) were used. The product was isolated by a silica gel column chromatography using petroleum ether / EtOAc = 5:1 as eluent to give **68** as a yellowish viscous oil (128.6 mg, 81% yield). ^{1}H NMR (400 MHz, $CDCl_3$) δ 7.52 – 7.49 (m, 2H), 7.46 – 7.43 (m, 1H), 7.32 – 7.22 (m, 7H), 7.20 – 7.12 (m, 5H), 6.88 – 6.82 (m, 4H), 6.00 (s, 1H), 4.63 – 4.42 (m, 2H), 4.04 – 3.82 (m, 3H), 3.76 (s, 3H), 1.03 (t, J = 7.2 Hz, 3H) ppm. ^{13}C NMR (101 MHz, $CDCl_3$) δ 171.7, 153.1, 141.3, 140.9, 140.4, 140.2, 132.4, 129.4, 129.1, 128.6, 127.5, 127.5, 127.3, 127.1, 126.9, 116.3, 114.6, 64.0, 60.9, 60.5, 55.4, 52.7, 13.6 ppm. HRMS (ESI) m/z: $[M + H]^+$ Calcd for $C_{31}H_{32}NO_5S$ 530.1996; Found 530.1993.

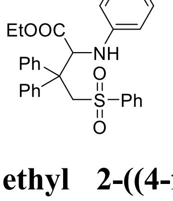

ethyl 2-((4-fluorophenyl)amino)-3,3-diphenyl-4-(phenylsulfonyl)butanoate (69)

According to the general procedure for the preparation of sulfonyl glycine derivative, ethyl (4-fluorophenyl)glycinate **1c** (0.3 mmol, 59.1 mg), 1,1-diphenylethylene **2** (0.36

mmol, 64.8 mg), *S*-phenyl benzenesulfonothioate **f1** (0.45 mmol, 112.5 mg) were used. The product was isolated by a silica gel column chromatography using petroleum ether / EtOAc = 5:1 as eluent to give **69** as a yellowish viscous oil (128.8 mg, 83% yield). ^{1}H NMR (400 MHz, $CDCl_3$) δ 7.50 – 7.43 (m, 3H), 7.34 – 7.29 (m, 2H), 7.24 – 7.21 (m, 5H), 7.20 – 7.14 (m, 3H), 7.11 – 7.08 (m, 2H), 6.95 – 6.90 (m, 2H), 6.84 – 6.80 (m, 2H), 5.99 (d, *J* = 12.0 Hz, 1H), 4.57 – 4.46 (m, 2H), 4.04 – 3.89 (m, 3H), 1.02 (t, *J* = 7.2 Hz, 3H) ppm. ^{13}C NMR (101 MHz, $CDCl_3$) δ 171.6, 156.7 (d, $J_{C\text{-}F}$ = 237.9 Hz), 142.7 (d, $J_{C\text{-}F}$ = 2.1 Hz), 141.4, 140.8, 140.4, 132.6, 129.4, 129.1, 128.8, 127.7, 127.7, 127.6, 127.4, 127.1, 116.0 (d, $J_{C\text{-}F}$ = 7.6 Hz), 115.7 (d, $J_{C\text{-}F}$ = 22.4 Hz), 64.1, 61.1, 60.2, 52.7, 13.7 ppm. ^{19}F NMR (376 MHz, $CDCl_3$) δ -125.6 ppm. HRMS (ESI) m/z: $[M + H]^+$ Calcd for $C_{30}H_{29}FNO_4S$ 518.1796; Found 518.1791.

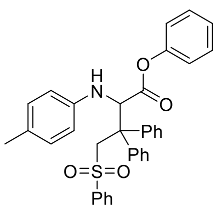

phenyl 3,3-diphenyl-4-(phenylsulfonyl)-2-(*p*-tolylamino)butanoate (70) According to the general procedure for the preparation of sulfonyl glycine derivative, phenyl *p*-tolylglycinate **1g** (0.3 mmol, 72.3 mg), 1,1-diphenylethylene **2** (0.36 mmol, 64.8 mg), *S*-phenyl benzenesulfonothioate **f1** (0.45 mmol, 112.5 mg) were used. The product was isolated by a silica gel column chromatography using petroleum ether / EtOAc = 5:1 as eluent to give **70** as a yellowish viscous oil (126.3 mg, 75% yield). ^{1}H NMR (400 MHz, $CDCl_3$) δ 7.61 – 7.59 (m, 2H), 7.51 – 7.46 (m, 1H), 7.38 – 7.29 (m, 8H), 7.28 – 7.23 (m, 6H), 7.19 – 7.16 (m, 1H), 7.15 – 7.12 (m, 2H), 6.93 – 6.91 (m, 2H), 6.75 – 6.72 (m, 2H), 6.34 (d, *J* = 11.6 Hz, 1H), 4.66 – 4.55 (m, 2H), 4.26 – 4.23 (m, 1H), 2.33 (s, 3H) ppm. ^{13}C NMR (101 MHz, $CDCl_3$) δ 170.8, 150.1, 143.7, 141.3, 140.6, 140.6, 132.7, 129.9, 129.6, 129.1, 129.1, 128.8, 128.7, 128.0, 127.8, 127.7, 127.4, 127.2, 125.8, 121.1, 114.8, 64.0, 59.8, 52.6, 20.4 ppm. HRMS (ESI) m/z: $[M + H]^+$ Calcd for $C_{35}H_{32}NO_4S$ 562.2047; Found 562.2041.

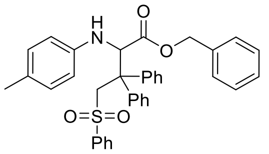

benzyl 3,3-diphenyl-4-(phenylsulfonyl)-2-(*p*-tolylamino)butanoate (71) According to the general procedure for the preparation of sulfonyl glycine derivative, benzyl *p*-tolylglycinate **1h** (0.3 mmol, 76.5 mg), 1,1-diphenylethylene **2** (0.36 mmol, 64.8 mg), *S*-phenyl benzenesulfonothioate **f1** (0.45 mmol, 112.5 mg) were used. The product was isolated by a silica gel column chromatography using petroleum ether / EtOAc = 5:1 as eluent to give **71** as a yellowish viscous oil (127.7 mg, 74% yield). ^{1}H NMR (400 MHz, $CDCl_3$) δ 7.54 – 7.46 (m, 3H), 7.34 – 7.31 (m, 5H), 7.24 – 7.18 (m, 4H), 7.16 – 7.07 (m, 10H), 6.83 – 6.81 (m, 2H), 6.18 (d, *J* = 11.2 Hz, 1H), 5.12 – 4.91 (m, 2H), 4.56 – 4.45 (m, 2H), 4.00 (d, *J* = 11.6 Hz, 1H), 2.33 (s, 3H) ppm. ^{13}C NMR (101 MHz, $CDCl_3$) δ 171.7, 144.0, 141.4, 140.6, 140.5, 134.9, 132.5, 129.8, 129.5, 129.0, 128.7, 128.6, 128.4, 128.2, 128.1, 127.7, 127.5, 127.4, 127.2, 127.0, 114.9, 66.9, 64.1, 59.5, 52.7, 20.4 ppm. HRMS (ESI) m/z: $[M + H]^+$ Calcd for $C_{36}H_{34}NO_4S$ 576.2203; Found 576.2199.

5. Studies on Synthetic Applications

5.1 Gram-scale synthesis

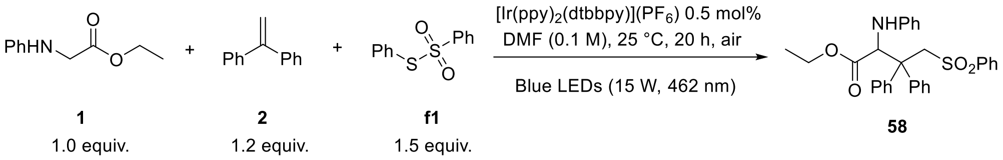

An oven-dried round-bottom flask (100 mL) was equipped with a magnetic stir bar and charged with the $[Ir(ppy)_2(dtbbpy)](PF_6)$ (0.028 mmol, 0.5 mol%), *N*-phenylglycine ethyl ester **1** (5.60 mmol, 1.0 g), 1,1-diphenylethylene **2** (6.72 mmol, 1.2 g), *S*-phenyl benzenesulfonothioate (8.40 mmol, 2.1 g) and DMF (30 mL). The reaction bottom was closed under air, placed in a home-made photochemical (LED) reactor (Figure S1), magnetically stirred and irradiated with LED (15 W, 462 nm) at 25 °C for 20 h (with a cooling fan to keep the reaction temperature). After quenching the reaction with water (20 mL), the resulting mixture was separated and extracted with EtOAc (20

mL x 3). The combined organic layers were dried over Na_2SO_4, concentrated under reduced pressure, and purified through a silica gel column (using petroleum ether / EtOAc = 5:1 as eluent) to give the pure desired product **58** (1.7 g, 61%) as a white solid.

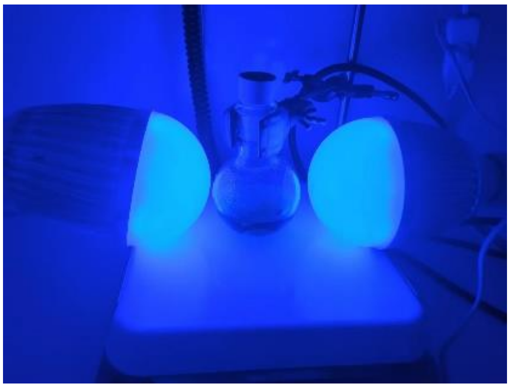

Figure S6 Gram-scale reaction

5.2 Removal of PMP group

5.2.1 The preparation of sulfonyl glycine derivative 72

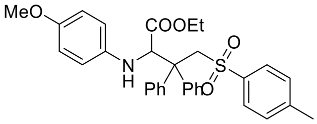

ethyl 2-((4-methoxyphenyl)amino)-3,3-diphenyl-4-tosylbutanoate (72) According to the general procedure for the preparation of sulfonyl glycine derivative, ethyl (4-methoxyphenyl)glycinate **1b** (0.3 mmol, 62.7 mg), 1,1-diphenylethylene **2** (0.36 mmol, 64.8 mg), *S*-phenyl 4-methylbenzenesulfonothioate **f2** (0.45 mmol, 118.8 mg) were used. The product was isolated by a silica gel column chromatography using petroleum ether / EtOAc = 5:1 as eluent to give **72** as a yellowish viscous oil (128.7 mg, 79% yield). ^{1}H NMR (400 MHz, $CDCl_3$) δ 7.42 – 7.40 (m, 2H), 7.26 – 7.16 (m, 8H), 7.13 – 7.01 (m, 4H), 6.86 – 6.80 (m, 4H), 5.98 (s, 1H), 4.54 – 4.39 (m, 2H), 4.02 – 3.86 (m, 2H), 3.77 (s, 3H), 2.37 (s, 3H), 1.02 (t, J = 7.2 Hz, 3H) ppm. ^{13}C NMR (101 MHz, $CDCl_3$) δ 171.9, 153.1, 143.5, 141.1, 140.6, 140.3, 138.6, 131.2, 129.5, 129.3, 129.2, 127.6, 127.5, 127.4, 127.1, 116.3, 114.6, 64.1, 61.0, 60.5, 55.5, 52.8, 21.4, 13.7 ppm. HRMS (ESI) m/z: $[M - H]^-$ Calcd for $C_{32}H_{32}NO_5S$ 542.2007; Found 542.2006.

5.2.2 Removal of PMP group experiments

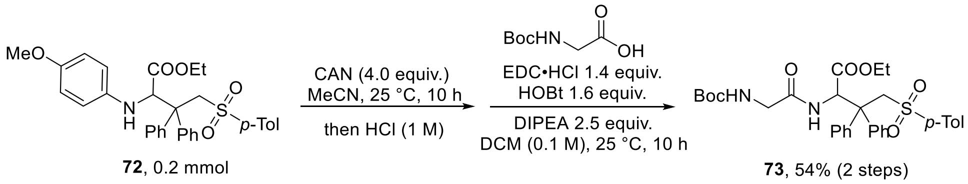

To a solution of CAN (ceric ammonium nitrate, 0.8 mmol, dissolved in 1.5 mL of H_2O) cooled in an ice bath was slowly added a solution of PMP-product **72** (0.2 mmol dissolved in 1.0 mL CH_3CN) in 0.5 h. The reaction was kept stirring for 2 h at that temperature before it was quenched with saturated aqueous Na_2CO_3. The mixture was extracted with EtOAc (10 mL x 3), washed with brine, dried over Na_2SO_4, and concentrated in vacuo. The crude product was mixed with Et_2O (5 mL), and HCl (5 mL, 1 M aq.) was then slowly added into the above solution under vigorous stirring. After the separation of organic phase, the aqueous layer was washed with Et_2O (5 mL x 3). Water was removed in vacuo and the residue was further dried to afford the amino ester hydrochloride as a beige powder. Then, in a 5 mL reaction tube, Boc-Gly (0.24 mmol, 42.0 mg), EDC•HCl (0.28 mmol, 53.7 mg) and HOBt (0.32 mmol, 43.2 mg,) were dissolved in d.r.y DCM (2 mL) under N_2. After the tube was cooled in an ice-water bath, DIPEA (0.5 mmol, 64.6 mg) was slowly added in 5 min. The reaction mixture was allowed to stir for 30 min before amino ester hydrochloride was added. The reaction was warmed to 25 °C and stirred for additional 16 h. Then, the mixture was quenched with water (4 mL), and extracted with EtOAc (2 mL x 3). The combined organic layers were washed with brine (2 mL), dried over Na_2SO_4, concentrated under reduced pressure, and purified through a silica gel column (using petroleum ether / EtOAc = 1:1 as eluent) to give **73** as a yellowish oil (54.3 mg, 54% yield for two steps).

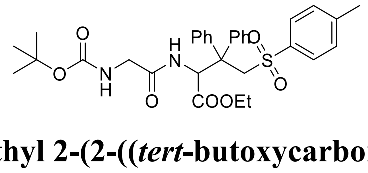

ethyl 2-(2-((*tert*-butoxycarbonyl)amino)acetamido)-3,3-diphenyl-4-tosylbutanoate (73) According to the procedure of removal of PMP group experiments, purified through a silica gel column (using petroleum ether / EtOAc = 1:1 as eluent) to give **73** as a yellowish oil (54.3 mg, 54% yield for two steps). ^{1}H NMR (400 MHz, $CDCl_3$) δ 7.43 – 7.41 (m, 2H), 7.26 – 7.24 (m, 3H), 7.17 – 7.08 (m, 10H), 6.07 – 6.05 (m, 1H),

5.08 (br, 1H), 4.67 – 4.63 (m, 1H), 4.33 – 4.30 (m, 1H), 3.96 – 3.88 (m, 2H), 3.79 (d, *J* = 5.6 Hz, 2H), 2.36 (s, 3H), 1.35 (s, 9H), 1.00 – 0.96 (m, 3H) ppm. ^{13}C NMR (101 MHz, $CDCl_3$) δ 170.1, 169.5, 155.6, 143.8, 140.4, 138.4, 129.4, 129.0, 128.3, 128.2, 127.9, 127.7, 127.4, 127.3, 79.9, 63.6, 61.5, 56.1, 52.3, 44.3, 28.1, 21.5, 13.6 ppm. HRMS (ESI) m/z: [M + H]$^+$ Calcd for $C_{32}H_{39}N_2O_7S$ 595.2472; Found 595.2470.

5.3 Synthesis of β-amino alcohol

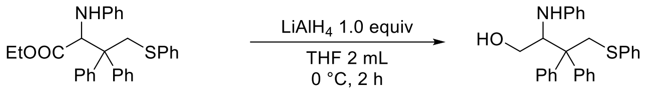

4, 0.2 mmol **74**

To a cooled (ice-bath) suspension of $LiAlH_4$ (7.6 mg, 0.20 mmol, in 1 mL of anhyd.r.ous THF) was slowly added a solution of **4** (93.4 mg, 0.2 mmol dissolved in 1 mL THF). The reaction was kept stirring for 2 h at that temperature before quenching with saturated aqueous $NaHCO_3$ (2 mL). The mixture was extracted with EtOAc (10 mL x 3) three times, washed with brine, dried over Na_2SO_4, concentrated under reduced pressure, and purified through a silica gel column (using petroleum ether / EtOAc = 3:1 as eluent) to give **74** as a colourless oil (46.8 mg, 55% yield).

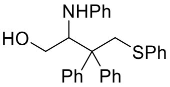

3,3-diphenyl-2-(phenylamino)-4-(phenylthio)butan-1-ol (74) ^{1}H NMR (400 MHz, $CDCl_3$) δ 7.40 – 7.27 (m, 8H), 7.25 – 7.23 (m, 2H), 7.19 – 7.16 (m, 2H), 7.08 – 7.02 (m, 3H), 6.91 – 6.89 (m, 2H), 6.86 – 6.84 (m, 2H), 6.75 – 6.71 (m, 1H), 4.88 – 4.82 (m, 1H), 4.13 – 4.09 (m, 1H), 3.77 (s, 2H), 3.47 – 3.43 (m, 1H), 3.25 (dd, *J* = 11.2, 8.0 Hz, 1H), 1.79 (br, 1H) ppm. ^{13}C NMR (101 MHz, $CDCl_3$) δ 148.3, 142.6, 141.8, 136.8, 129.6, 129.5, 129.4, 129.1, 128.5, 128.0, 128.0, 127.3, 127.1, 125.8, 118.2, 113.9, 64.3, 58.5, 55.8, 45.8 ppm. HRMS (ESI) m/z: [M + H]$^+$ Calcd for $C_{28}H_{28}NOS$ 426.1886; Found 426.1879.

5.4 Compatibility with biomolecules

5.4.1 General procedure for the test of compatibility with biomolecules

The reactions were carried out in 8 mL transparent vials. Each vial was equipped with a magnetic stir bar and charged with the [Ir(ppy)$_2$(dtbbpy)](PF$_6$) (0.0015 mmol, 0.5 mol%), *N*-phenylglycine ethyl ester **1** (0.3 mmol, 53.7 mg), 1,1-diphenylethylene **2** (0.36 mmol, 64.8 mg), diphenyl disulfide **3** (0.45 mmol, 98.1 mg), biomolecule (0.3 mmol, 1.0 equiv.) and MeCN/H_2O = 4/1 (v/v, 3 mL). The reaction vial was closed under air, placed in a home-made photochemical (LED) reactor (Figure S1), magnetically stirred and irradiated with a 15 W blue LED (462 nm) at 25 °C for 10 h. After quenching the reaction with water (5 mL), the resulting mixture was separated and extracted with EtOAc (10 mL x 3). The combined organic layers were dried over Na_2SO_4, concentrated under reduced pressure, and purified through a silica gel column (EtOAc and petro ether as the elution).

5.4.2 Results of the test of compatibility with biomolecules

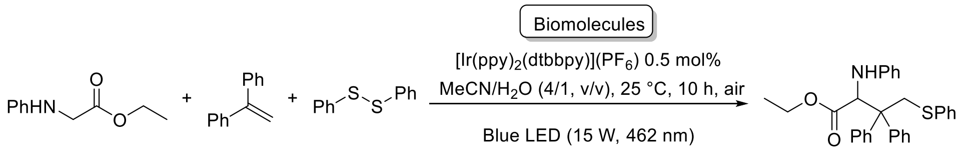

1 1.0 equiv. **2** 1.2 equiv. **3** 1.5 equiv. **4**

Table S10 Compatibility with biomolecules

Entry	Biomolecules	Yield (%)^b	Entry	Biomolecules	Yield (%)^b
1	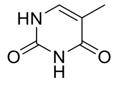 Thymine (1 equiv.)	61	5	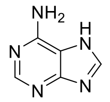 Adenine (1 equiv.)	57
2	Bovine serum albumin (10 mg/mL)	53	6	Casein (10 mg/mL)	57
3	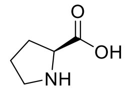 L-Proline (1 equiv.)	57	7	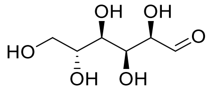 D(+)-Glucose (1 equiv.)	60
4	Trypsin (10 mg/mL)	55	8	ATP (1 equiv.)	58

^{a} Reaction conditions: **1** (0.3 mmol, 1.0 equiv.), **2** (0.36 mmol, 1.2 equiv.), **3** (0.45 mmol, 1.5 equiv.), photocatalyst (0.0015 mmol, 0.5 mol%), biomolecule (0.3 mmol, 1.0 equiv.) MeCN/H_2O (3 mL, 4/1, v/v), irradiation with a 15 W blue LED under air, 25 °C, 10 h. ^{b} Isolated yields are given.

6. Mechanistic studies

6.1 Radical-trapping experiments

6.1.1 Radical-trapping experiments of carbosulfanylation

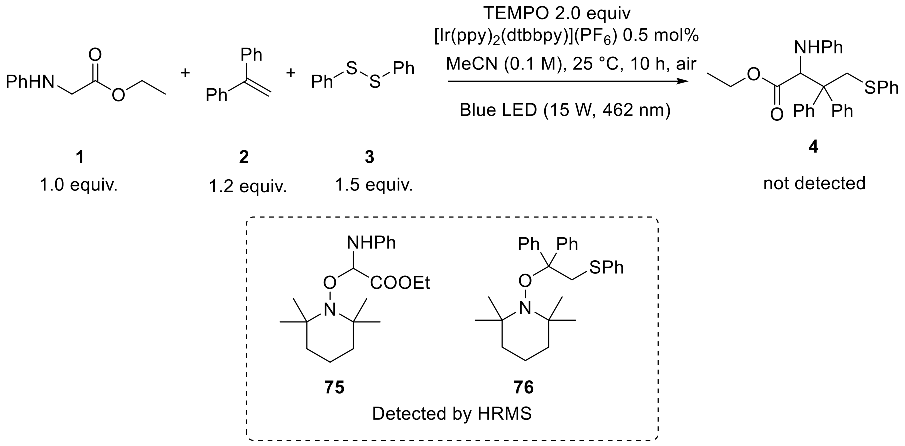

A transparent vial (8 mL) was equipped with a magnetic stir bar and charged with the [Ir(ppy)$_2$(dtbbpy)](PF$_6$) (0.0015 mmol, 0.5 mol%), *N*-phenylglycine ethyl ester **1** (0.3 mmol, 53.7 mg), 1,1-diphenylethylene **2** (0.36 mmol, 64.8 mg), diphenyl disulfide **3** (0.45 mmol, 98.1 mg), TEMPO (0.60 mmol, 93.8 mg) and MeCN (3 mL). The reaction vial was closed under air, placed in a home-made photochemical (LED) reactor (Figure S1), magnetically stirred and irradiated with a 15 W blue LED (462 nm) at 25 °C for 10 h.

75, HRMS (ESI) m/z: $[M + H]^+$ Calcd for $C_{19}H_{31}N_2O_3$ 335.2329; Found 335.2336.

76, HRMS (ESI) m/z: $[M + H]^+$ Calcd for $C_{29}H_{36}NOS$ 446.2512; Found 446.2520.

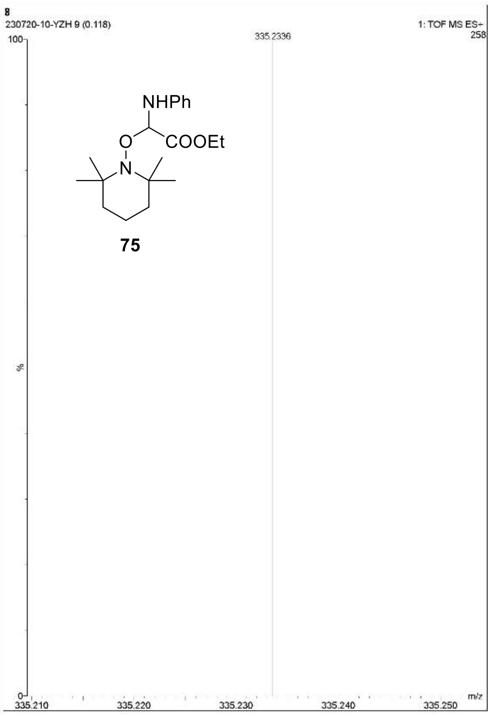

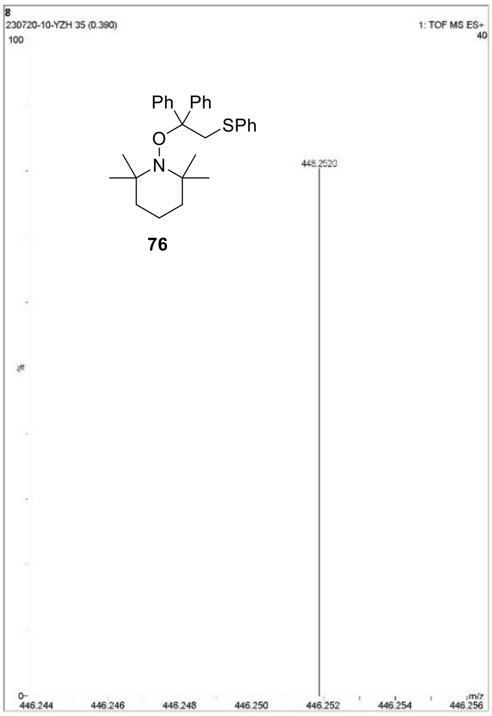

Figure S7 HRMS spectra of radical-trapping experiment of carbosulfanylation

6.1.2 Radical-trapping experiments of carbosulfonylation

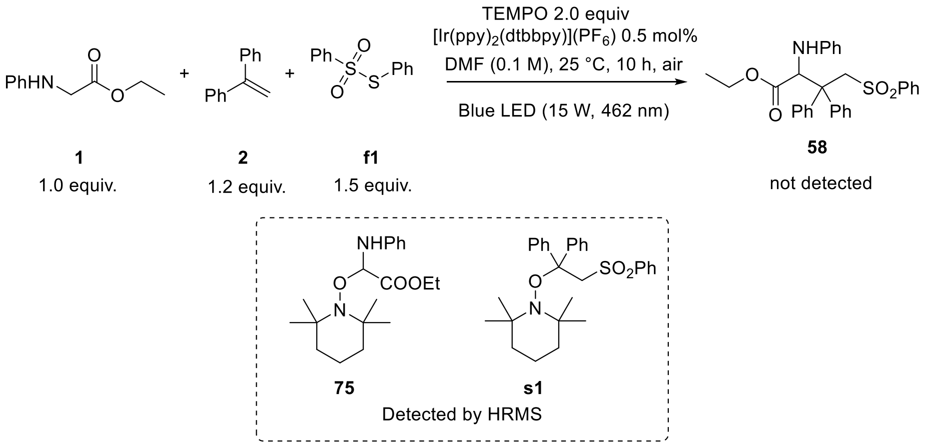

A transparent vial (8 mL) was equipped with a magnetic stir bar and charged with the [Ir(ppy)$_2$(dtbbpy)](PF$_6$) (0.0015 mmol, 0.5 mol%), *N*-phenylglycine ethyl ester **1** (0.3 mmol, 53.7 mg), 1,1-diphenylethylene **2** (0.36 mmol, 64.8 mg), *S*-phenyl benzenethiosulfonate **f1** (0.45 mmol, 112.7 mg) , TEMPO (0.60 mmol, 93.8 mg) and DMF (3 mL). The reaction vial was closed under air, placed in a home-made photochemical (LED) reactor (Figure S1), magnetically stirred and irradiated with a 15 W blue LED (462 nm) at 25 °C for 10 h.

75, HRMS (ESI) m/z: $[M + H]^+$ Calcd for $C_{19}H_{31}N_2O_3$ 335.2329; Found 335.2328.

s1, HRMS (ESI) m/z: $[M + Na]^+$ Calcd for $C_{29}H_{35}NO_3SNa$ 500.2230; Found 500.2233.

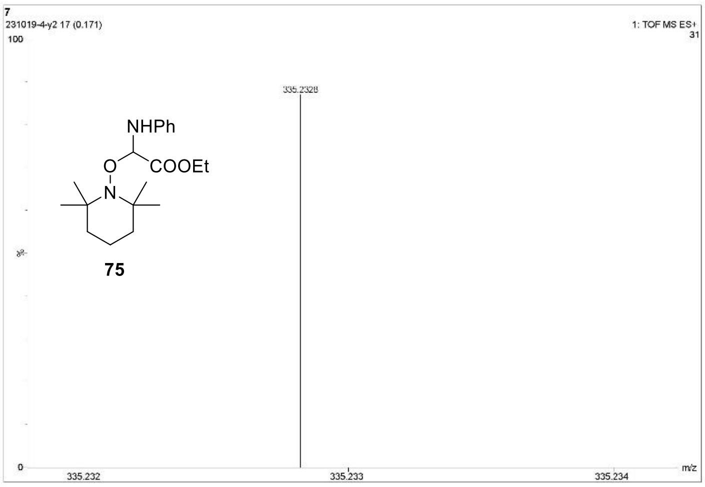

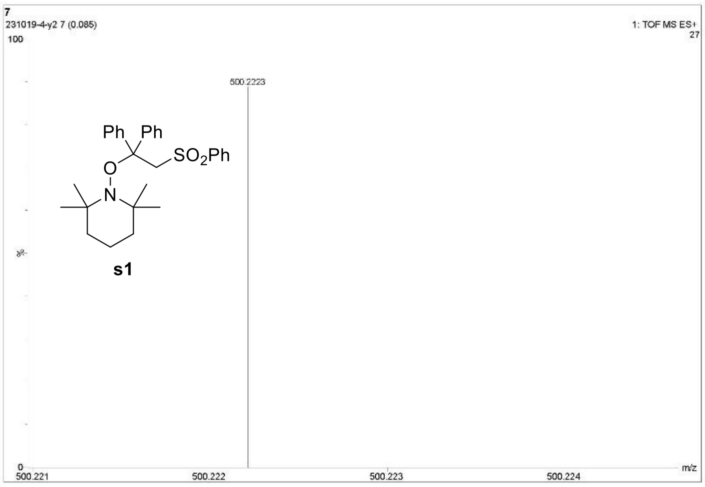

Figure S8 HRMS spectra of radical-trapping experiment of carbosulfonylation

6.2 By-products and absence of photocatalyst

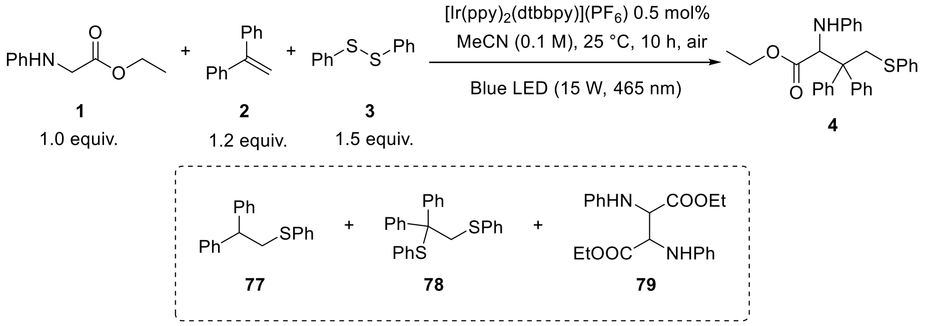

Table S11 Conversion rate of by-products and absence of photocatalyst

Entry	Variation to standard conditions	Yield[a] of **4** (%)	Yield[b] of **77** and **78** (%)	Yield[a] of **79** (%)
1	/	69	23 (1:1)	13
2	Without photocatalyst	<10	N.D.	N.D.
3	Without **1**	-	35 (1:5)	N.D.
4	Without photocatalyst and **1**	-	<5	N.D.

Reaction conditions: **1** (0.3 mmol, 1.0 equiv.), **2** (0.36 mmol, 1.2 equiv.), **3** (0.45 mmol, 1.5 equiv.), [Ir(ppy)$_2$(dtbbpy)](PF$_6$) (0.0015 mmol, 0.5 mol%), MeCN (3 mL), Irradiation with a 15 W blue LED (462 nm) under air, 25 °C, 10 h. [a] Isolated yields are given (according to **1**). [b] Mixtures of **77** and **78** are obtained by silica gel column and the ratios are determined by ^{1}H NMR (according to **2**).

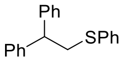

(2,2-diphenylethyl)(phenyl)sulfane (77)[16], yellow oil. ^{1}H NMR (400 MHz, CDCl$_3$) δ 7.35 – 7.27 (m, 8H), 7.26 – 7.16 (m, 7H), 4.21 (t, *J* = 8.0 Hz, 1H), 3.60 (d, *J* = 8.0 Hz, 2H) ppm. ^{13}C NMR (101 MHz, CDCl$_3$) δ 143.0, 136.5, 129.4, 128.9, 128.6, 127.9, 126.7, 126.0, 50.5, 39.6 ppm.

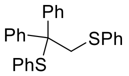

(1,1-diphenylethane-1,2-diyl)bis(phenylsulfane) (78), colorless oil. ^{1}H NMR (400 MHz, CDCl$_3$) δ 7.39 – 7.37 (m, 4H), 7.29 – 7.27 (m, 3H), 7.26 – 7.23 (m, 4H), 7.18 – 7.09 (m, 5H), 7.06 – 6.98 (m, 4H), 3.83 (s, 2H) ppm. ^{13}C NMR (101 MHz, CDCl$_3$) δ ^{13}C NMR (101 MHz, CDCl$_3$) δ 143.1, 137.3, 137.2, 130.7, 129.9, 129.2, 129.1, 128.6, 128.3, 127.6, 127.0, 126.0, 63.2, 46.7 ppm. HRMS (ESI) m/z: [M + H]$^+$ Calcd for $C_{26}H_{23}S_2$ 399.1236; Found 399.1236.

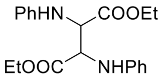

diethyl 2,3-bis(phenylamino)succinate (79)[17], white solid, 13.9 mg, 13% yield. ^{1}H NMR (400 MHz, $CDCl_3$) δ 7.23 – 7.19 (m, 4H), 6.83 – 6.79 (m, 2H), 6.73 – 6.70 (m, 4H), 4.70 – 4.66 (m, 2H), 4.30 – 4.17 (m, 4H), 1.33 – 1.23 (m, 6H) ppm. ^{13}C NMR (101 MHz, $CDCl_3$) δ 171.0, 170.7, 146.5, 146.4, 129.3, 129.3, 119.2, 119.1, 114.4, 114.2, 62.0, 61.8, 59.3, 59.1, 14.1, 14.0 ppm.

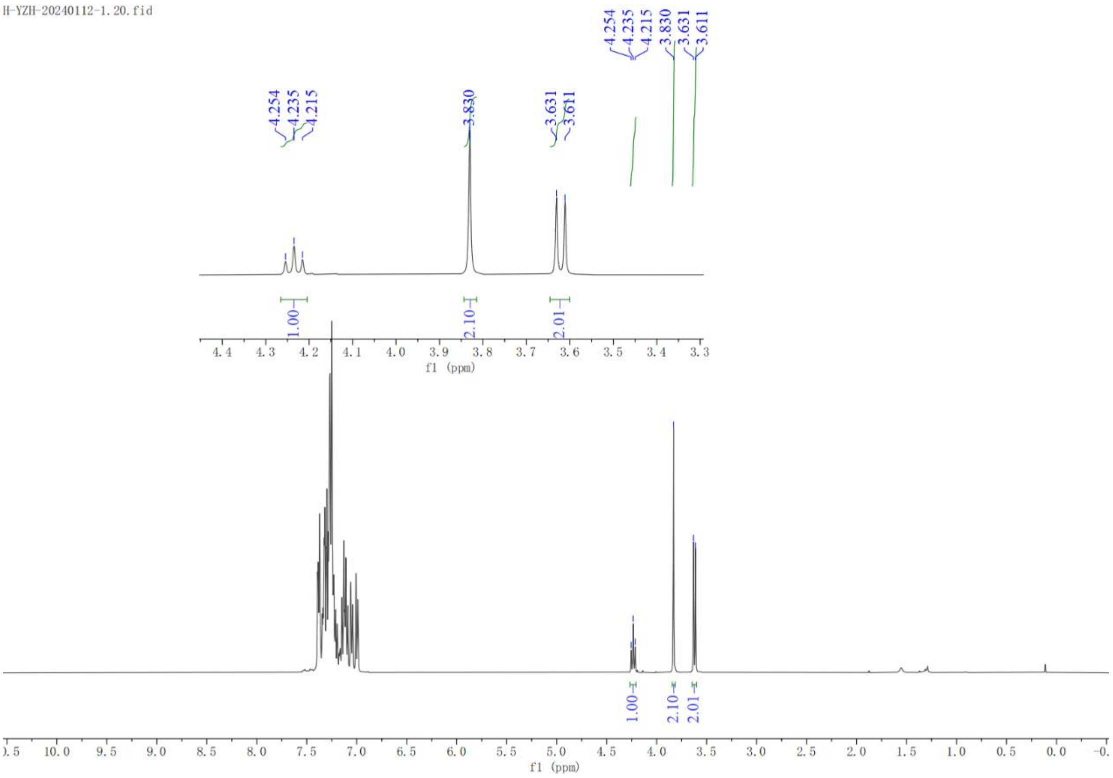

Figure S9 ^{1}H NMR spectrum of **77** and **78** for entry 1, Table S11 (**77**:**78** = 1:1)

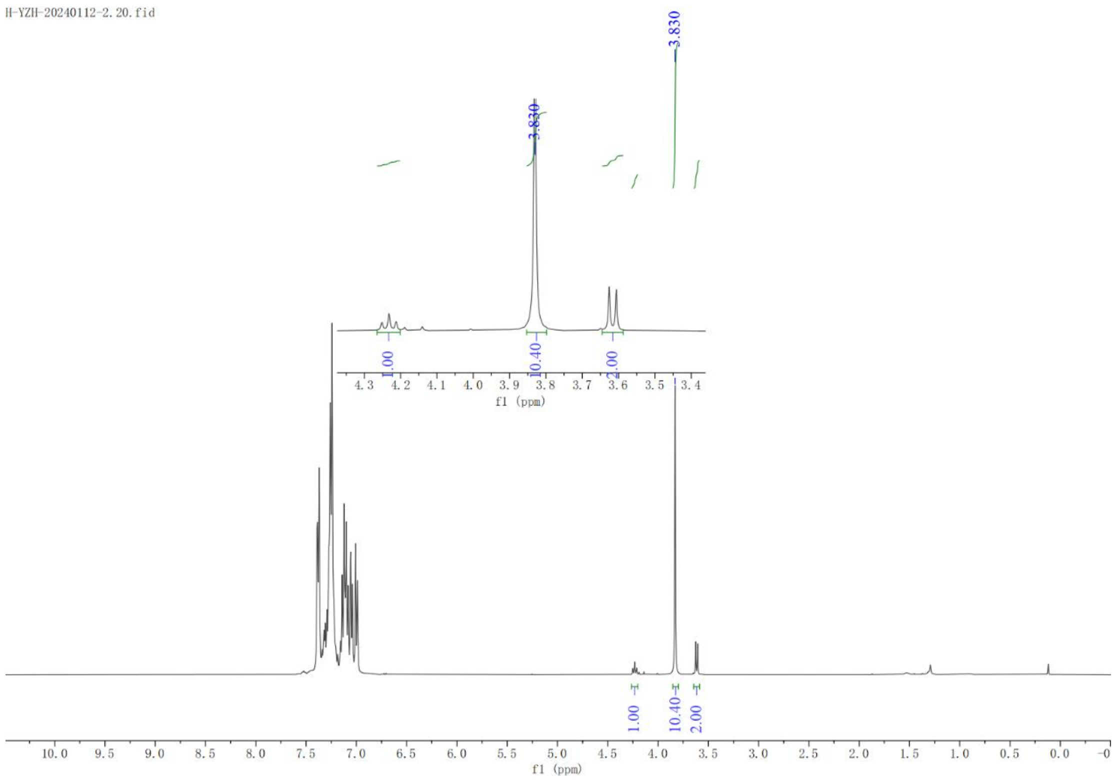

Figure S10 ^{1}H NMR spectrum of **77** and **78** for entry 3, Table S11 **77**:**78** = 1:5

6.3 Stern-Volmer fluorescence quenching

Fluorescence spectra were collected on Thermo Scientific spectrofluorimeter. [Ir(ppy)$_2$(dtbbpy)](PF$_6$) was irradiated at 420 nm and the emission intensity at approximate 590 nm was observed.

Stern-Volmer Emission Quenching Experiment: A quartz cuvette was charged with a 1.25 x 10^{-6} M solution of [Ir(ppy)$_2$(dtbbpy)](PF$_6$) in dry MeCN (1 mL) and the initial fluorescence emission was measured. Then new samples were prepared by mixing [Ir(ppy)$_2$(dtbbpy)](PF$_6$) (1.25 x 10^{-6} M) and a known amount of other reactant (5 mM, 10 mM, 15 mM) in MeCN (total volume = 1 mL).

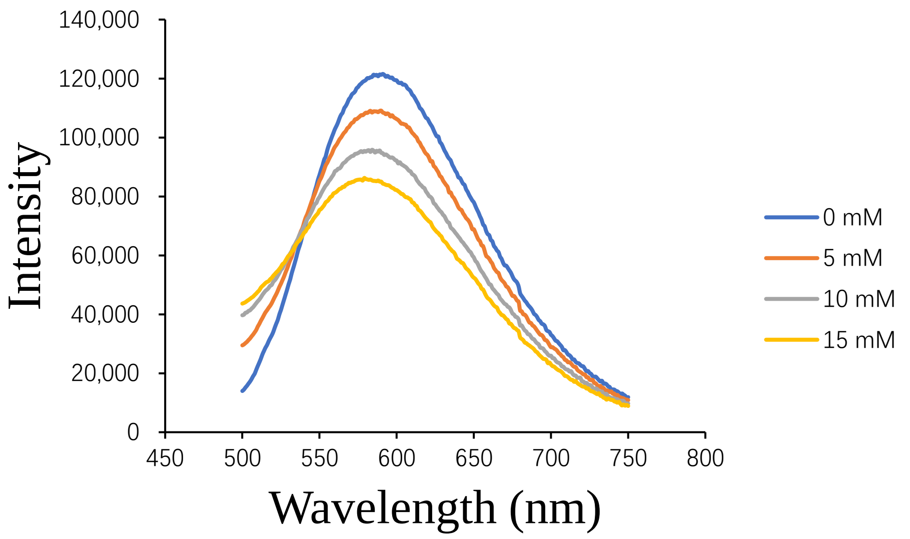

Figure S11 Fluorescence spectra of [Ir(ppy)$_2$(dtbbpy)](PF$_6$) with different concentration of *N*-phenylglycine ethyl ester (**1**)

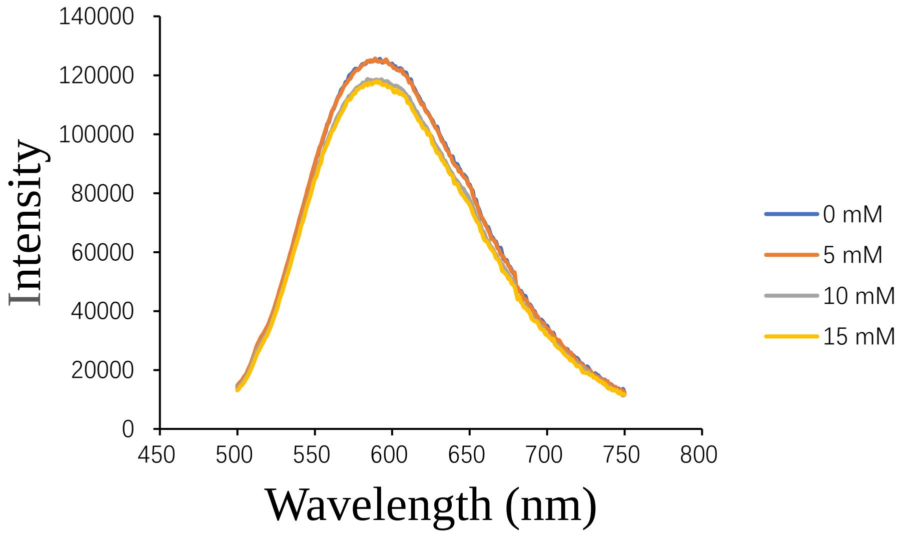

Figure S12 Fluorescence spectra of [Ir(ppy)$_2$(dtbbpy)](PF$_6$) with different concentration of styrene (**2j**)

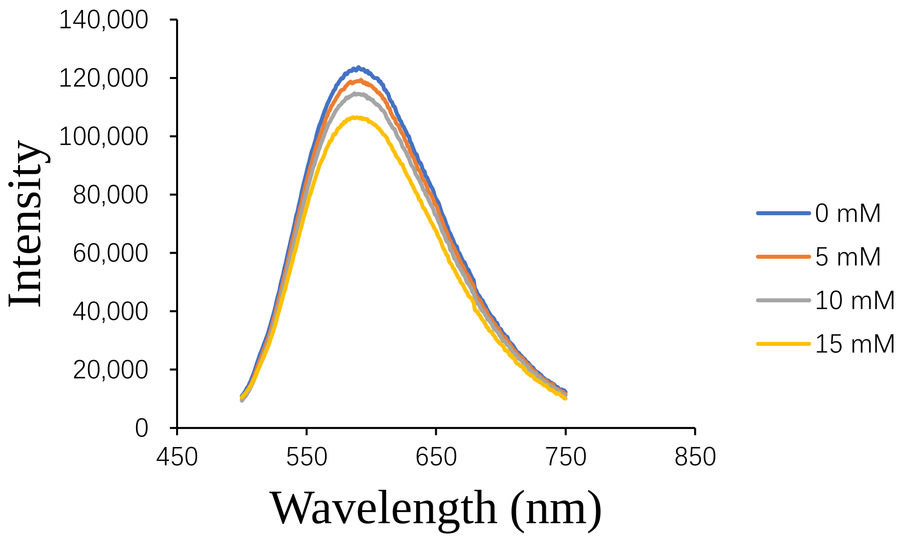

Figure S13 Fluorescence spectra of [Ir(ppy)$_2$(dtbbpy)](PF$_6$) with different concentration of diphenyl disulfide (**3**)

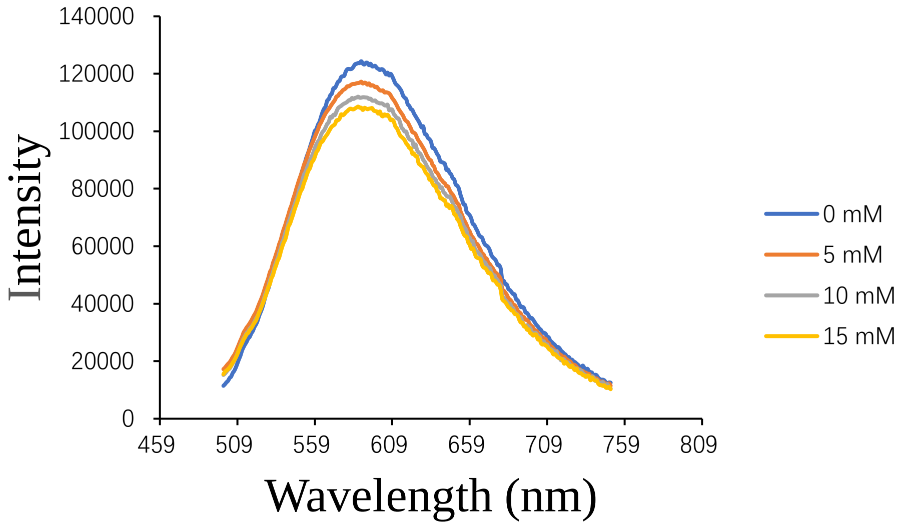

Figure S14 Fluorescence spectra of [Ir(ppy)$_2$(dtbbpy)](PF$_6$) with different concentration of butyl disulfide (**3p**)

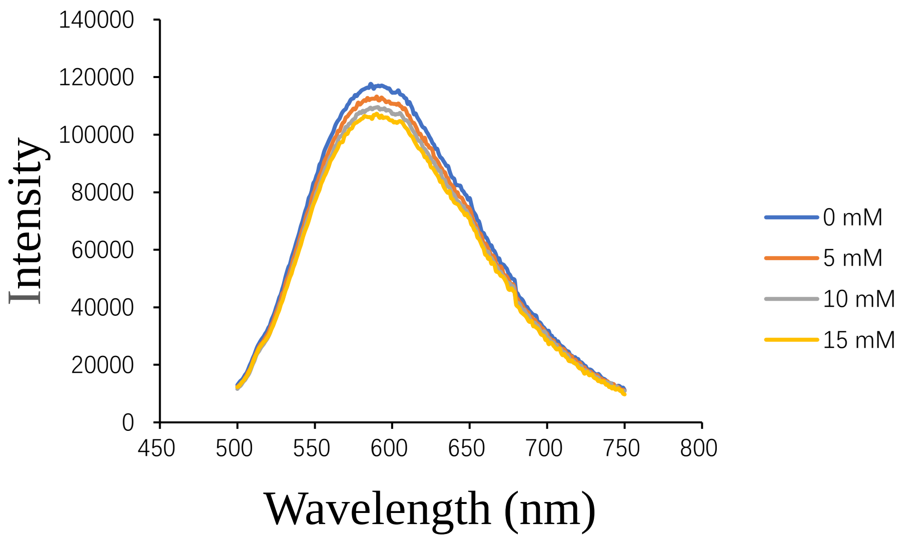

Figure S15 Fluorescence spectra of $[Ir(ppy)_2(dtbbpy)](PF_6)$ with different concentration of *S*-phenyl benzenethiosulfonate (**f1**)

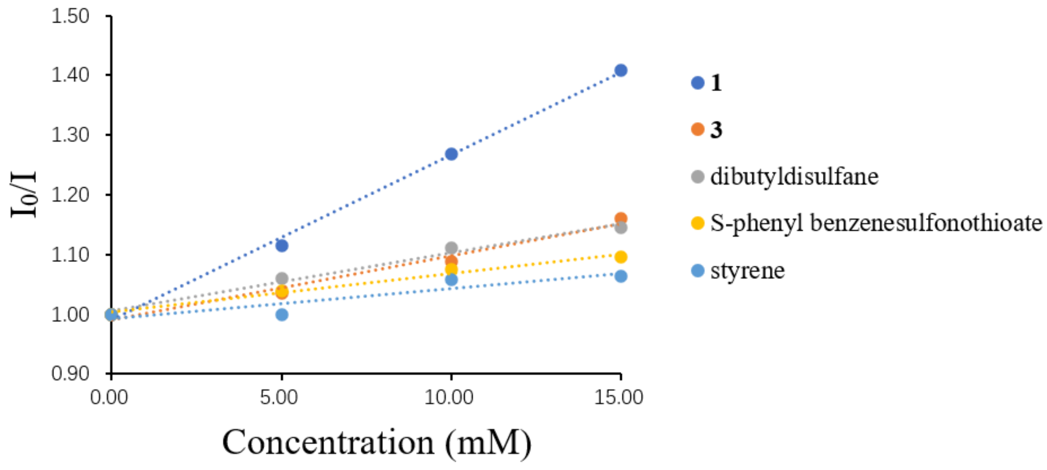

Figure S16 Linear fitting of different quenchers

6.4 Light-dark cycle experiment

According to the general procedure for the preparation of sulfanyl glycine derivative products, six identical reactions were performed in parallel and place them in the same environment. Take out one reaction bottle every two hours and perform a light on/off operation. Perform post-processing and purified through a silica gel column to give the product.

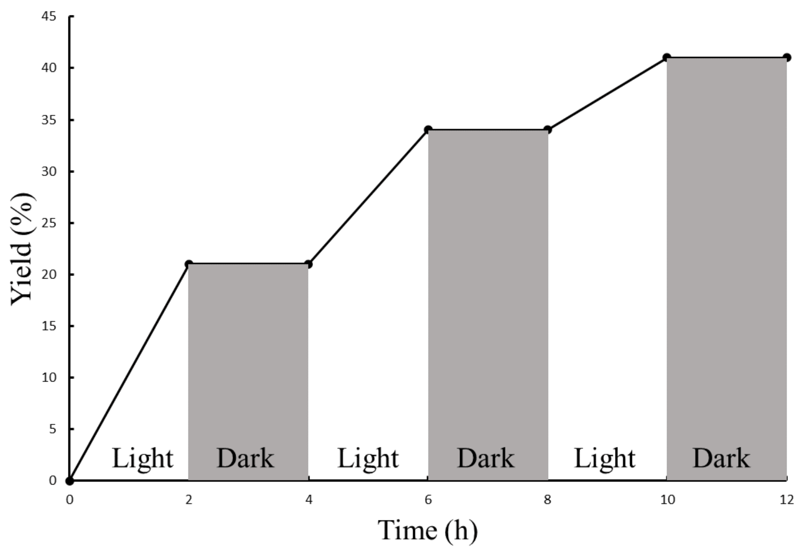

Figure S17 Light on-off experiments

6.5 Radical crossover experiments

Procedure for scrambling experiments: The reactions were carried out in 8 mL transparent vials. Each vial was equipped with a magnetic stir bar and charged with referred disulfides (both 0.10 mmol) and MeCN (2 mL). The reaction vial was closed under air, placed in a home-made photochemical (LED) reactor (Figure S1), magnetically stirred and irradiated with a 15 W blue LED (462 nm) at 25 °C for 10 h. Subsequently, the reaction quenched with water (5 mL), the resulting mixture was separated and extracted with EtOAc (10 mL x 3). The combined organic layers were dried over Na_2SO_4 and concentrated under reduced pressure. The residue was analyzed by ^{19}F NMR (376 MHz, $CDCl_3$).

6.5.1 Scrambling experiment between disulfide 3a and 3c

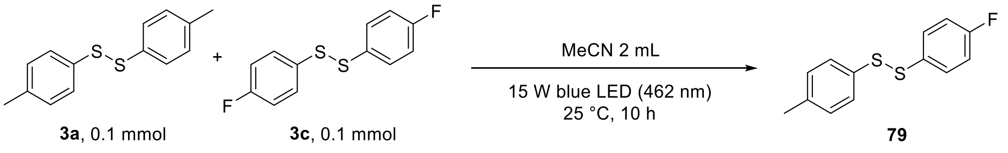

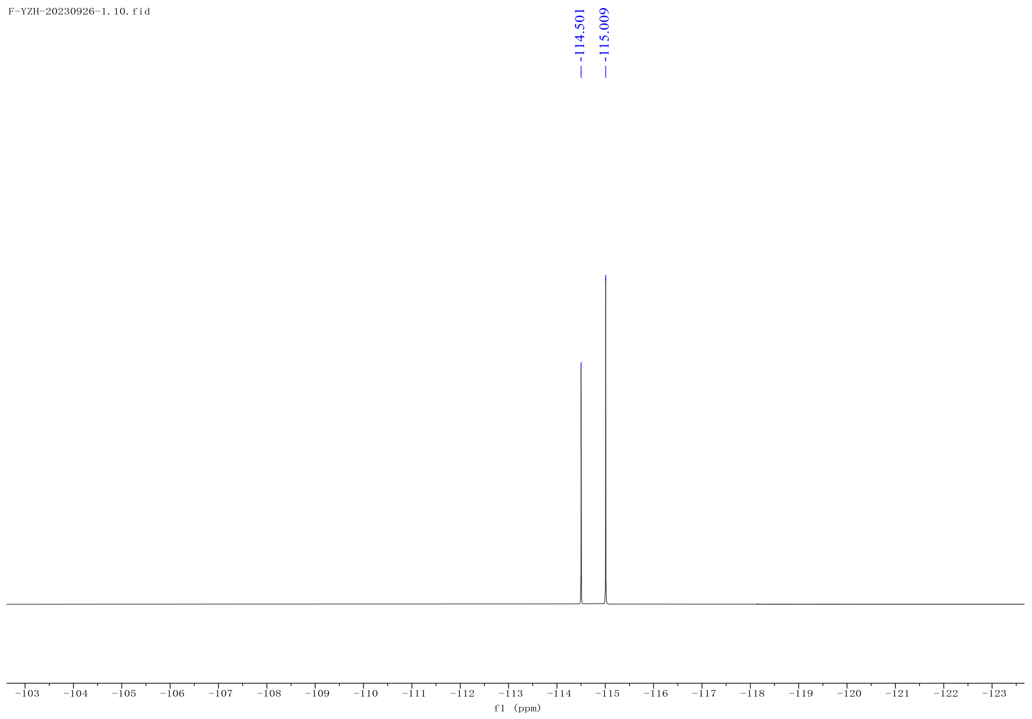

Figure S18 ^{19}F NMR of scrambling experiment between disulfide **3a** and **3c**

6.5.2 Scrambling experiment between thiosulfonates f9 and f10

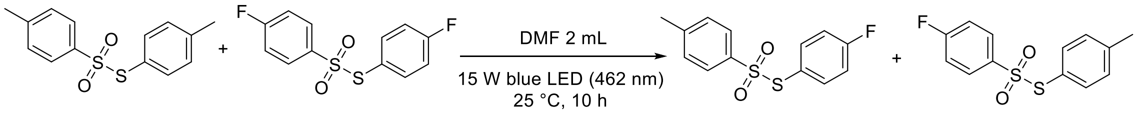

f9, 0.1 mmol **f10**, 0.1 mmol Detected by ^{19}F NMR

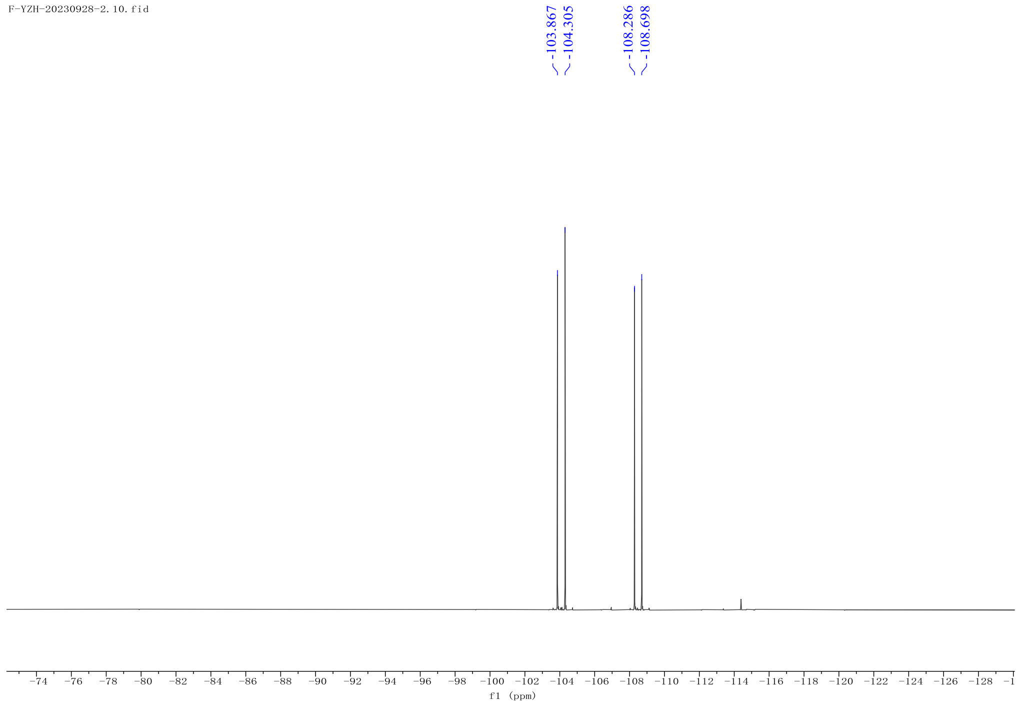

Figure S19 ^{19}F NMR of scrambling experiment between thiosulfonates **f9** and **f10**

6.6 Imine analogue instead of glycinate experiments

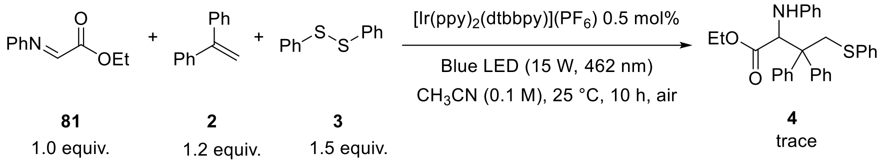

Imine analogue instead of glycinate experiments: A transparent vial (8 mL) was equipped with a magnetic stir bar and charged with the [Ir(ppy)$_2$(dtbbpy)](PF$_6$) (0.0015 mmol, 0.5 mol%), Imine **81** (0.3 mmol, 53.1 mg), 1,1-diphenylethylene **2** (0.36 mmol, 64.8 mg), diphenyl disulfide **3** (0.45 mmol, 98.1 mg), and MeCN (3 mL). The reaction vial was closed under air, placed in a home-made photochemical (LED) reactor (Figure S1), magnetically stirred and irradiated with a 15 W blue LED (462 nm) at 25 °C for 10 h. TLC (thin—layer chromatography) was used for the test of the product formation.

6.7 UV light experiment

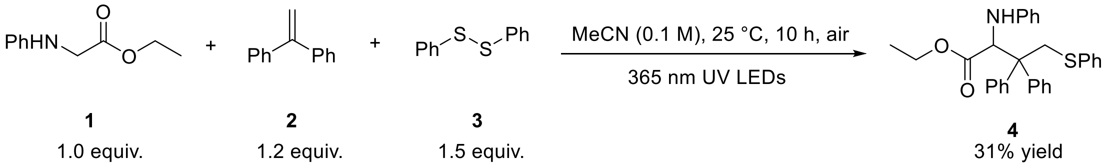

A transparent vial (8 mL) was equipped with a magnetic stir bar and charged with the *N*-phenylglycine ethyl ester **1** (0.3 mmol, 53.7 mg), 1,1-diphenylethylene **2** (0.36 mmol, 64.8 mg), diphenyl disulfide **3** (0.45 mmol, 98.1 mg) and MeCN (3 mL). The reaction vial was closed under air, placed in a home-made photochemical (LED) reactor (Figure S1), magnetically stirred and irradiated with 365 nm LEDs at 25 °C for 10 h. After quenching the reaction with water (5 mL), the resulting mixture was separated and extracted with EtOAc (10 mL x 3). The combined organic layers were dried over Na_2SO_4, concentrated under reduced pressure. The product was isolated by a silica gel column chromatography using petroleum ether / EtOAc = 20:1 as eluent to give **4** as a white solid (43.5 mg, 31% yield).

When the reaction was performed under the irradiation of UV light in the absence of photocatalyst, 31% yield of **4** was obtained, indicating that a possible hydrogen atom

transfer between *N*-phenylglycinate ethyl ester and thiyl radical might happen as a minor pathway along with the proposed main mechanism.

7. X-ray crystallographic data for 4

Single-crystal X-ray diffraction measurement for **4** was carried out at 150 K on Bruker APEX II CCD diffractometer operating at 50 KV and 30 mA using Mo-$K\alpha$ radiation (λ = 0.71073Å). Single-crystal of **4** was determined at 213 K on Bruker D8 VENTURE diffractometer with a PHOTON 100 CMOS detector equipped with METALJET-X-ray Source (Ga, λ = 1.34138 Å). Crystals were mounted on a loop using Parabar 10312 oil for data collection. Data was collected with a series of φ and/or ω scans. Data was integrated using SAINT and scaled with either a numerical or multi-scan absorption correction using SADABS. Structures were solved using SHELXT and refined by full-matrix least squares on F^2 using the SHELXL and OLEX2 program. All non-hyd.r.ogen atoms were refined anisotropically, and all hyd.r.ogen atoms were added in idealized positions and refined using a riding model. The atomic coordinates and structure factors have been deposited in the Cambridge Structural Database (CSD) of the Cambridge Crystallographic Data Centre (CCDC), https://www.ccdc.cam.ac.uk/structures/ [CCDC number 2270550 for **4**].

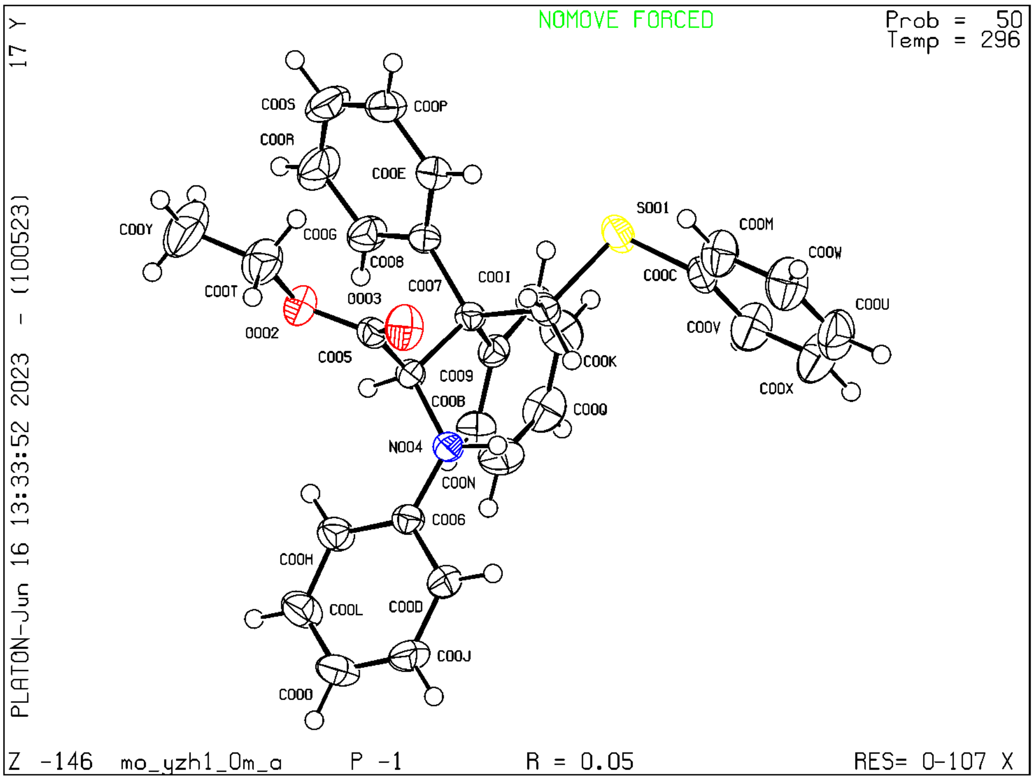

Figure S20 X-Ray crystallographic spectrum of **4** (50% thermal ellipsoids)

Table S12 Crystal data and structure refinement for **4**

Identification code	2270550
Empirical formula	$C_{30}H_{29}NO_2S$
Formula weight	467.60
Temperature/K	296.15
Crystal system	triclinic
Space group	P-1
a/Å	9.712(3)
b/Å	11.760(4)
c/Å	12.338(4)
α/°	108.416(4)
β/°	110.622(4)
γ/°	91.533(4)
Volume/Å^3	1236.2(6)

Z	2
ρ_{calc}g/cm^3	1.256
μ/mm^{-1}	0.158
F(000)	496.0
Crystal size/mm^3	0.05 × 0.03 × 0.01
Radiation	MoKα (λ = 0.71073)
2Θ range for data collection/°	3.696 to 55.19
Index ranges	-12 ≤ h ≤ 12, -15 ≤ k ≤ 15, -16 ≤ l ≤ 15
Reflections collected	17537
Independent reflections	5654 [R_{int} = 0.0287, R_{sigma} = 0.0304]
Data/restraints/parameters	5654/0/308
Goodness-of-fit on F^2	1.040
Final R indexes [I>=2σ (I)]	R_1 = 0.0539, wR_2 = 0.1601
Final R indexes [all data]	R_1 = 0.0669, wR_2 = 0.1715
Largest diff. peak/hole / e Å^{-3}	0.58/-0.39

8. References

[1] A. Kahnt, A. Rentmeister, D. Guldi, F. Glorius, *Nat. Chem.* **2018**, *10*, 981.

[2] J. Luo, J. Zhang, *ACS Catal.* **2016**, *6*, 873-877.

[3] D.-M. Yan, J.-R. Chen, W.-J. Xiao, *Angew. Chem. Int. Ed.* **2019**, *58*, 378.

[4] Y. Gao, J. Liu, C. Wei, Y. Li, K. Zhang, L. Song, L. Cai, *Nat. Commun.* **2022**, *13*, 7450.

[5] I. Okamura, S. Park, J. H. Han, S. Notsu, H. Sugiyama, *Chem. Lett.* **2017**, *46*, 1597–1600.

[6] R. Wang, J. Wang, Y. Zhang, B. Wang, Y. Xia, F. Xue, W. Jin, C. Liu, *Adv. Synth. Catal.* **2023**, *365*, 900.

[7] Y. Zhou, L. Zhao, M. Hu, X.-H. Duan, L. Liu, *Org. Lett.* **2023**, *25*, 5268−5272.

[8] C. Wan, R.-J. Song, J.-H. Li, *Org. Lett.* **2019,** *21*, 2800−2803.

[9] X. Qiu, X. Yang, Y. Zhang, S. Song, N. Jiao, *Org. Chem. Front.* **2019**, *6*, 2220.

[10] H. George, R. N. Charles, F. Harry, M. David, C. M. Colin, B. P. Matthew, W.

Karen, G. Yan, H. T. Keith, C. Peter (Argenta Discovery AG), WO2008074966, **2008**.

[11] P. Mampuys, Y. Zhu, S. Sergeyev, E. Ruijter, R. V. A. Orru, S. Van Doorslaer, B. U. W. Maes, *Org. Lett.* **2016**, *18*, 2808−2811.

[12] K. Gadde, P. Mampuys, A. Guidetti, H. Y. V. Ching, W. A. Herrebout, S. Van Doorslaer, K. A. Tehrani, B. U. W. Maes, *ACS Catal.* **2020**, *10*, 8765−8779.

[13] Z. Tan, F. Chen, G. Huang, Y. Li, H. Jiang, W. Zeng, *Org. Lett.* **2023**, *25*, 2846−2851.

[14] J. Tong, H. Li, Y. Zhu, P, Liu, P. Sun, *Green Chem*. **2022**, *24*, 1995.

[15] L. Cao, S.-H. Luo, K. Jiang, Z.-F. Hao, B.-W. Wang, C.-M. Pang, Z.-Y. Wang, *Org. Lett.* **2018**, *20*, 4754−4758.

[16] M. Miele, A. Citarella, T. Langer, E. Urban, M. Zehl, W. Holzer, L. Ielo, V. Pace *Org. Lett.* **2020**, *22*, 7629−7634.

[17] C. Wang, M. Guo, R. Qi, Q. Shang, Q. Liu, S. Wang, L. Zhao, R. Wang, Z. Xu, *Angew. Chem. Int. Ed.* **2018**, *57*, 15841.

9. ^{1}H NMR, ^{13}C NMR and ^{19}F NMR Spectra

^{1}H NMR (400 MHz, DMSO-d_6) spectrum of **1e**

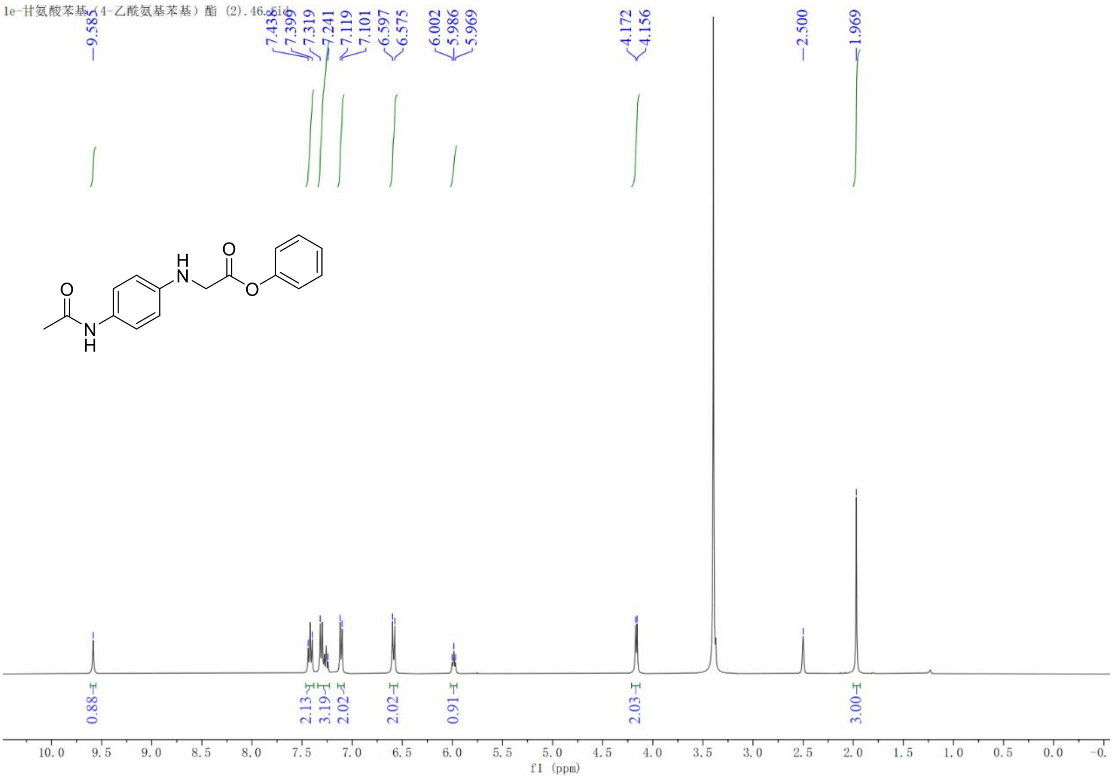

^{13}C NMR (101 MHz, DMSO-d_6) spectrum of **1e**

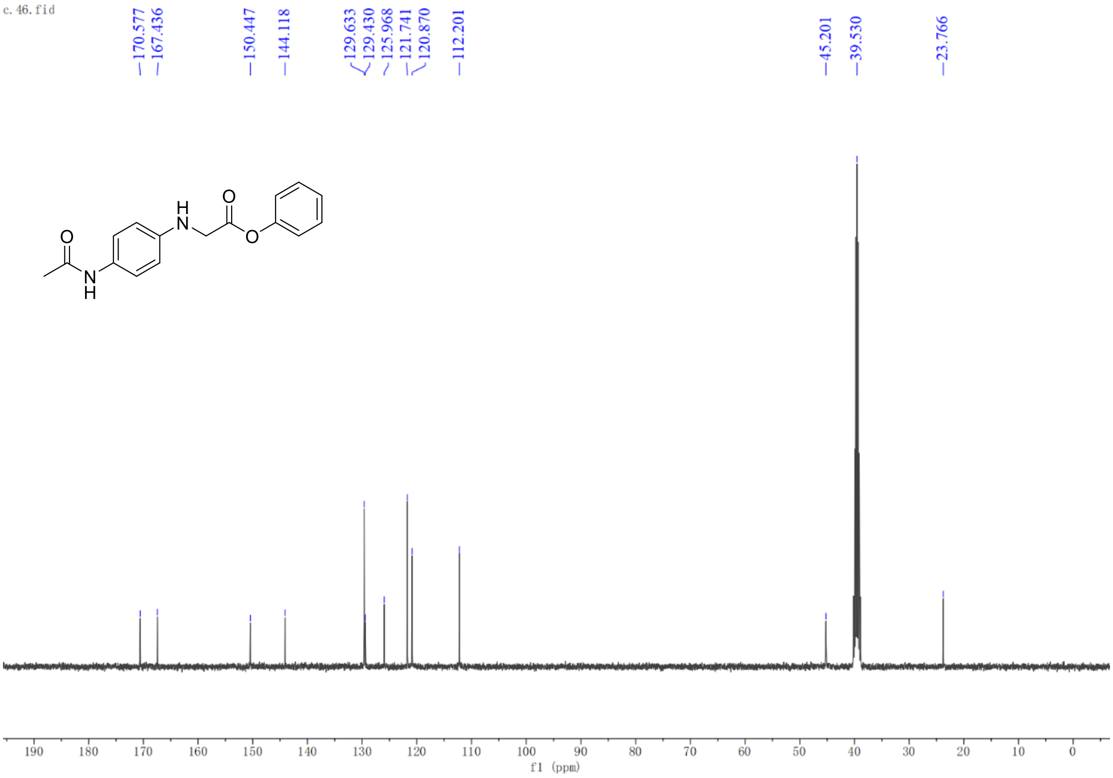

^{1}H NMR (400 MHz, $CDCl_3$) spectrum of **1f**

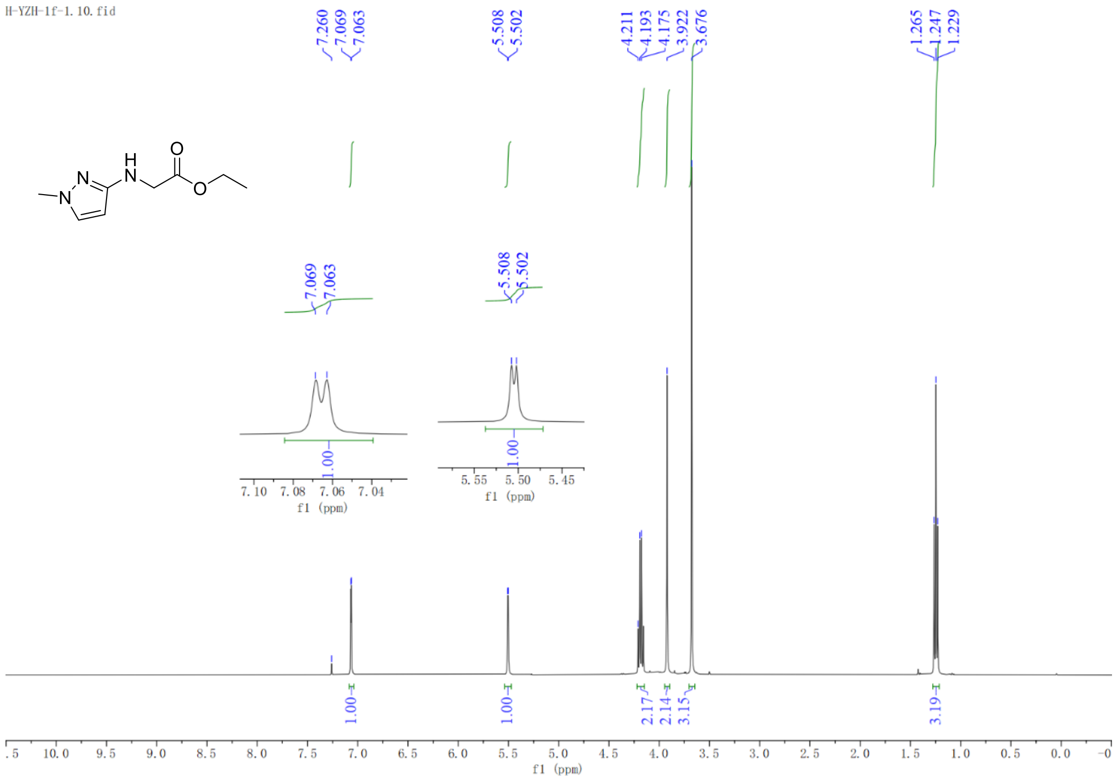

^{13}C NMR (101 MHz, $CDCl_3$) spectrum of **1f**

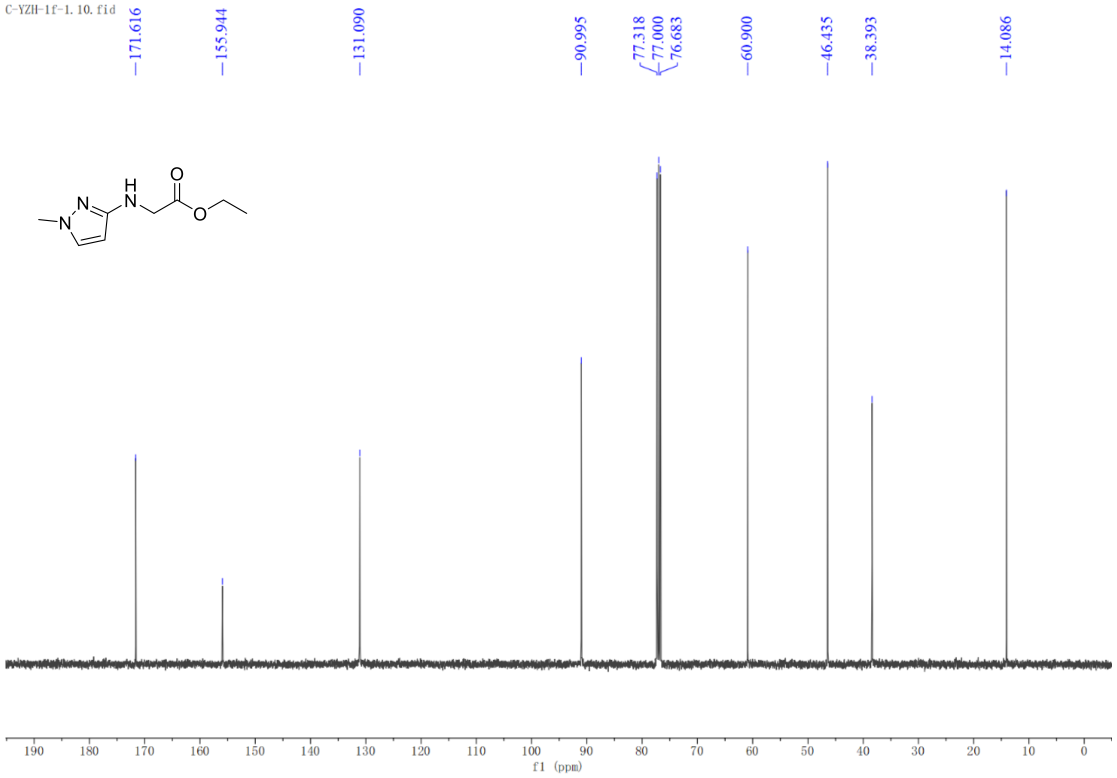

^{1}H NMR (400 MHz, $CDCl_3$) spectrum of **1i**

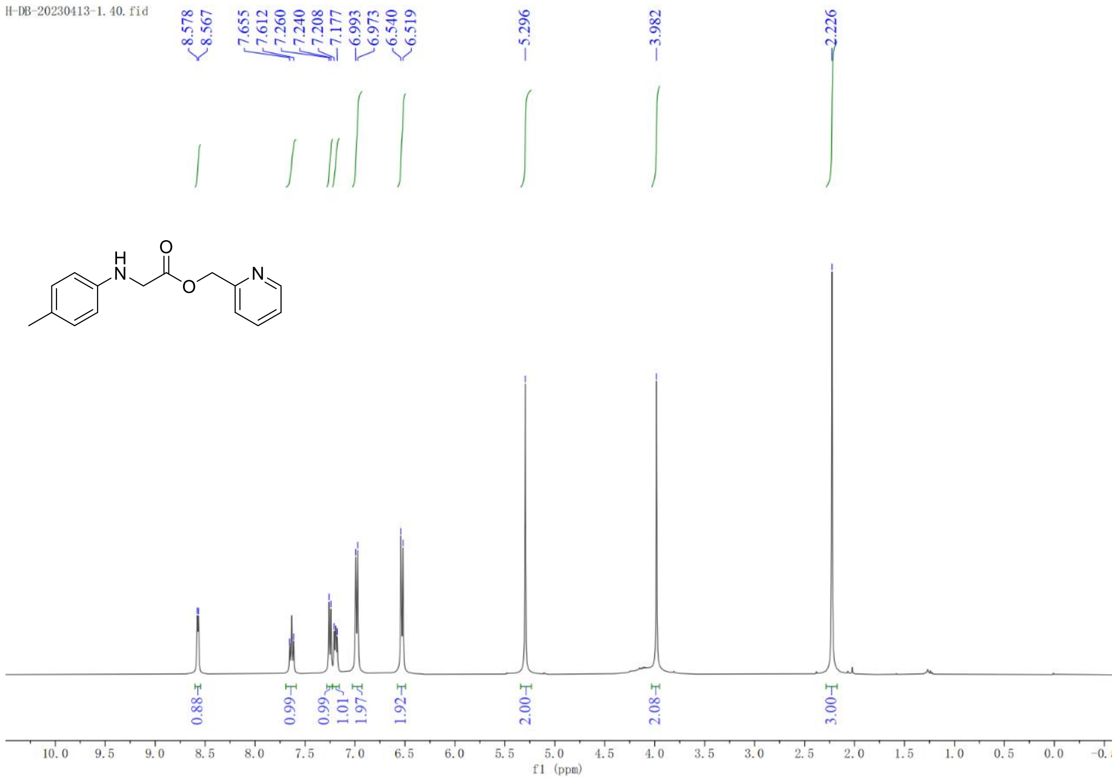

^{13}C NMR (101 MHz, $CDCl_3$) spectrum of **1i**

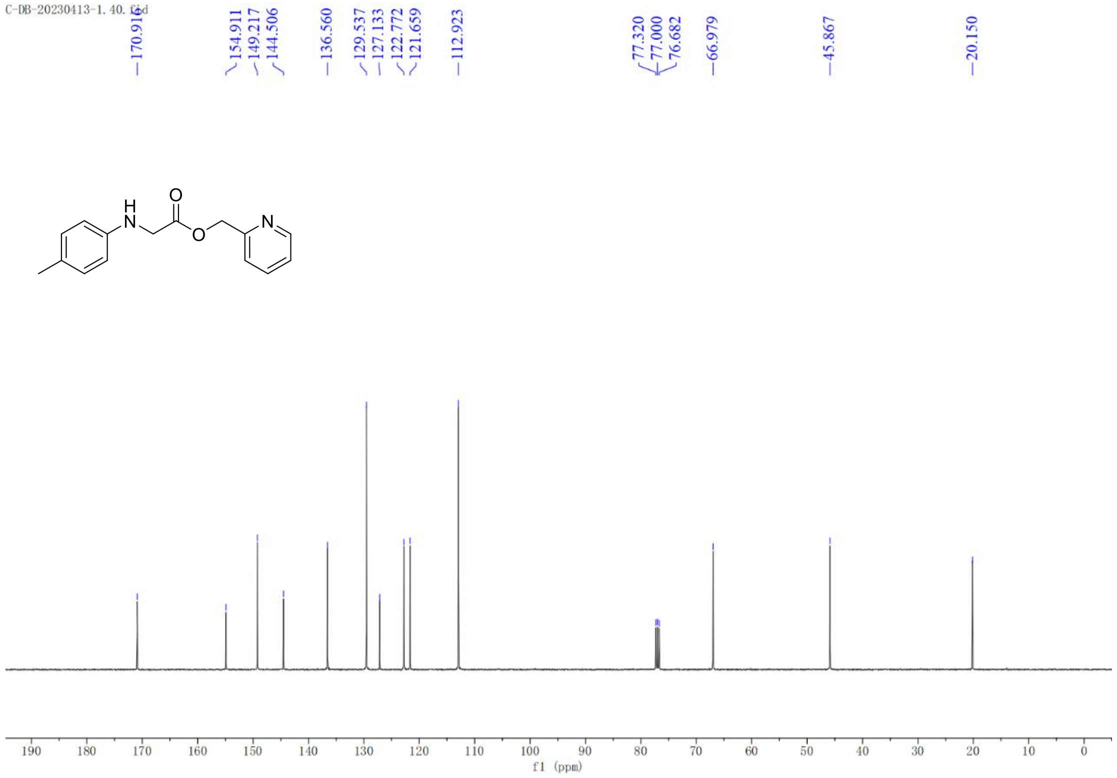

^{1}H NMR (400 MHz, $CDCl_3$) spectrum of **1l**

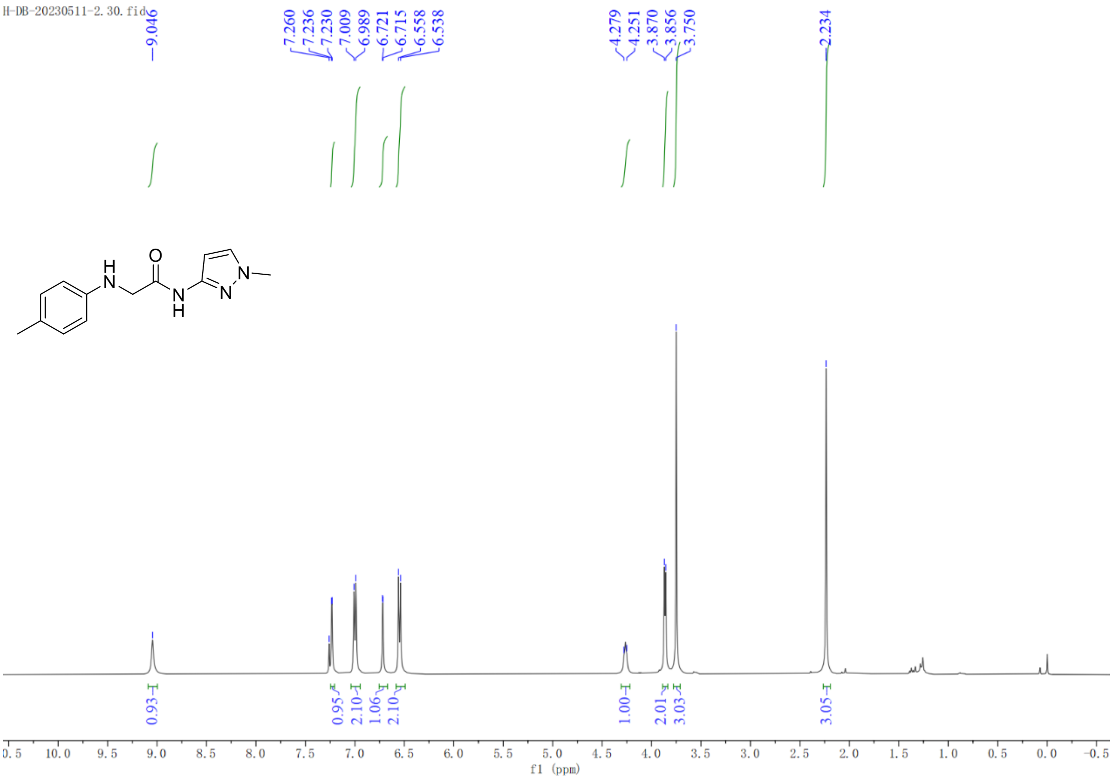

^{13}C NMR (101 MHz, $CDCl_3$) spectrum of **1l**

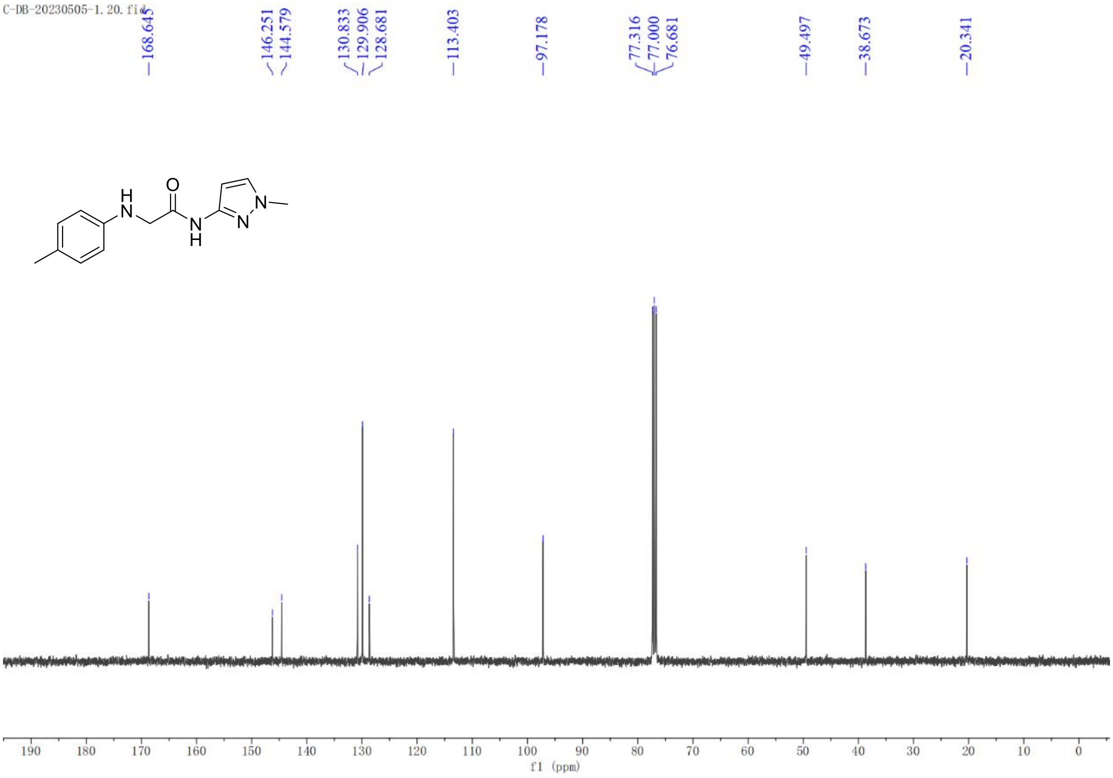

^{1}H NMR (400 MHz, $CDCl_3$) spectrum of **1n**

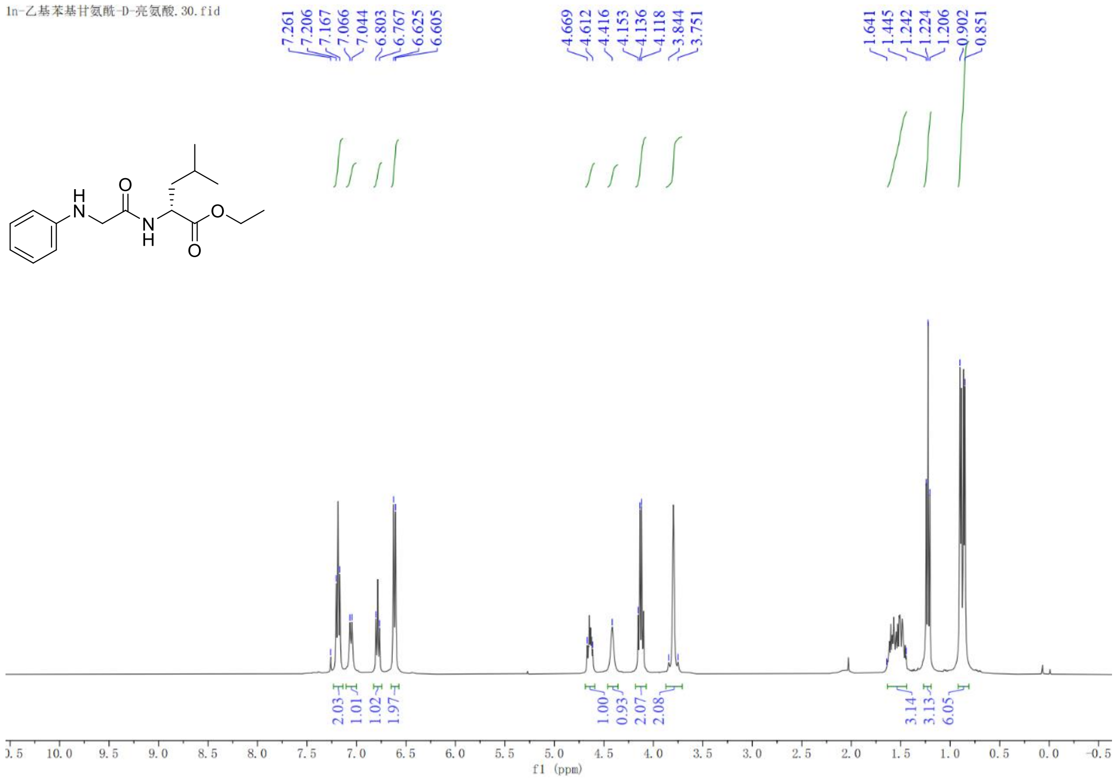

^{13}C NMR (101 MHz, $CDCl_3$) spectrum of **1n**

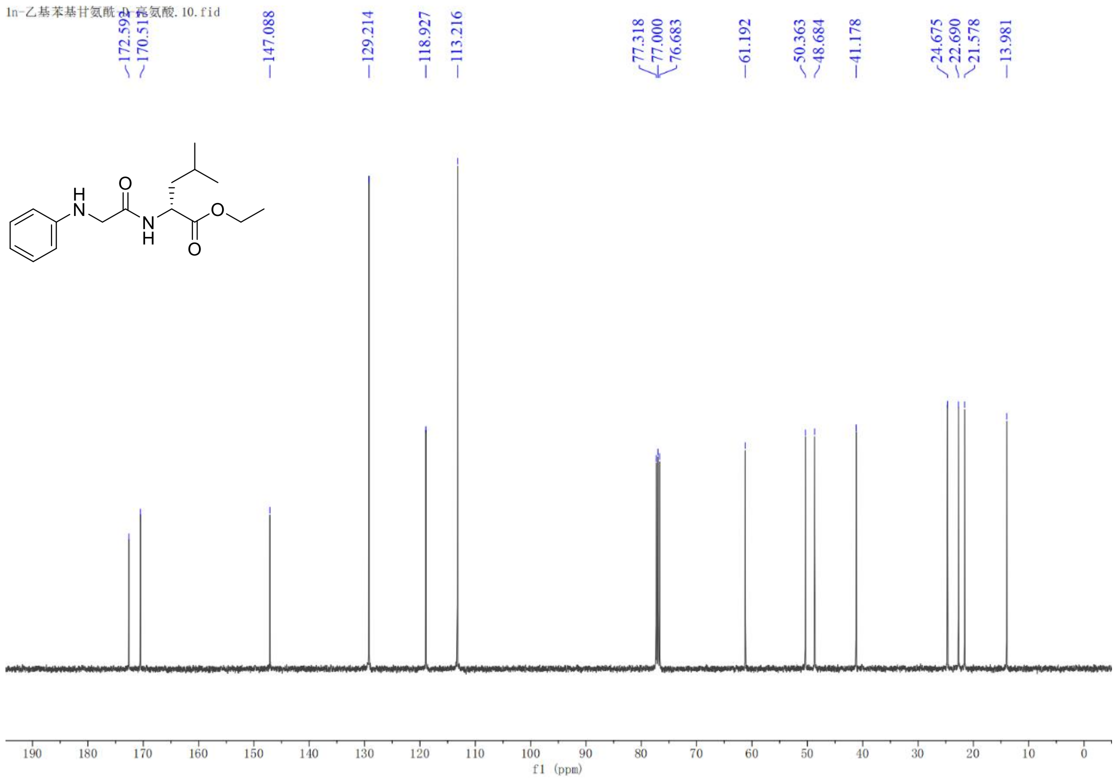

^{1}H NMR (400 MHz, $CDCl_3$) spectrum of **1p**

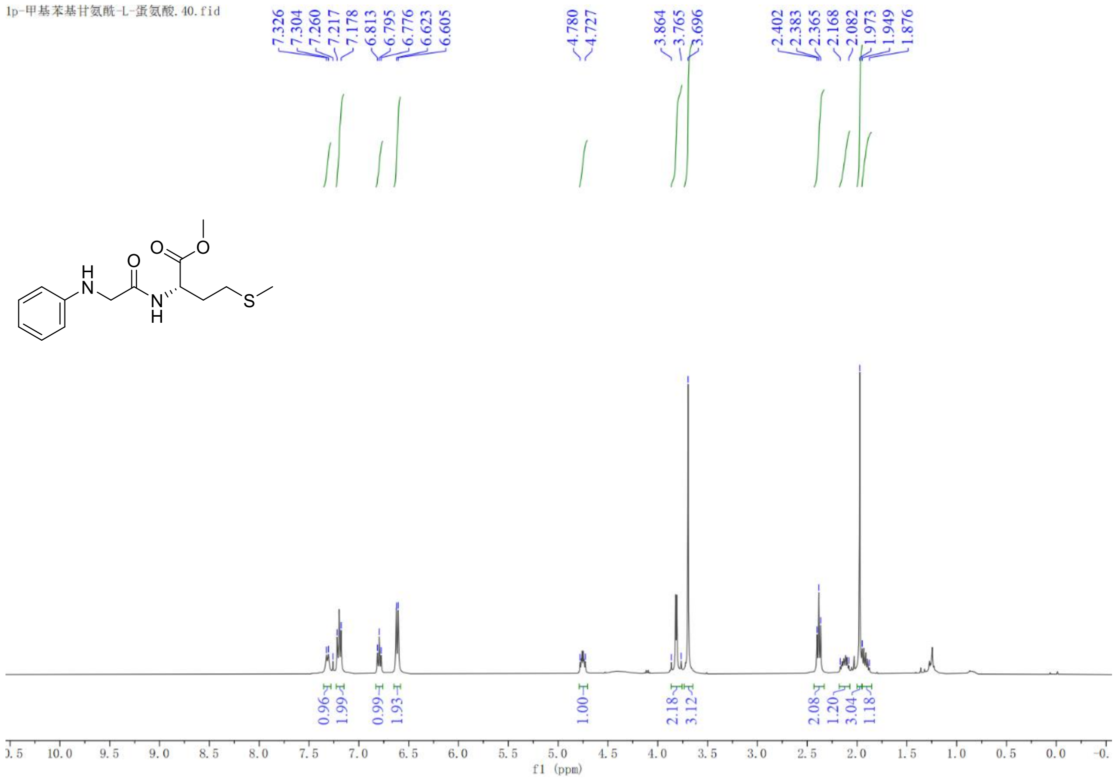

^{13}C NMR (101 MHz, $CDCl_3$) spectrum of **1p**

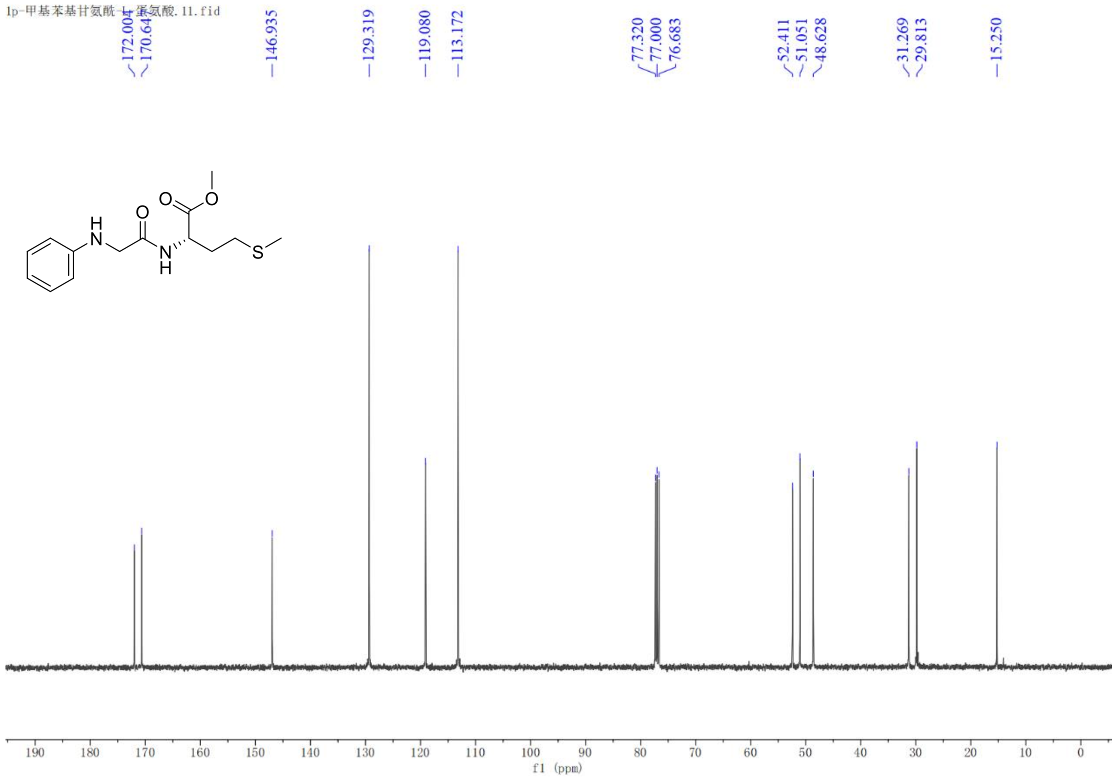

^{1}H NMR (400 MHz, $CDCl_3$) spectrum of **1q**

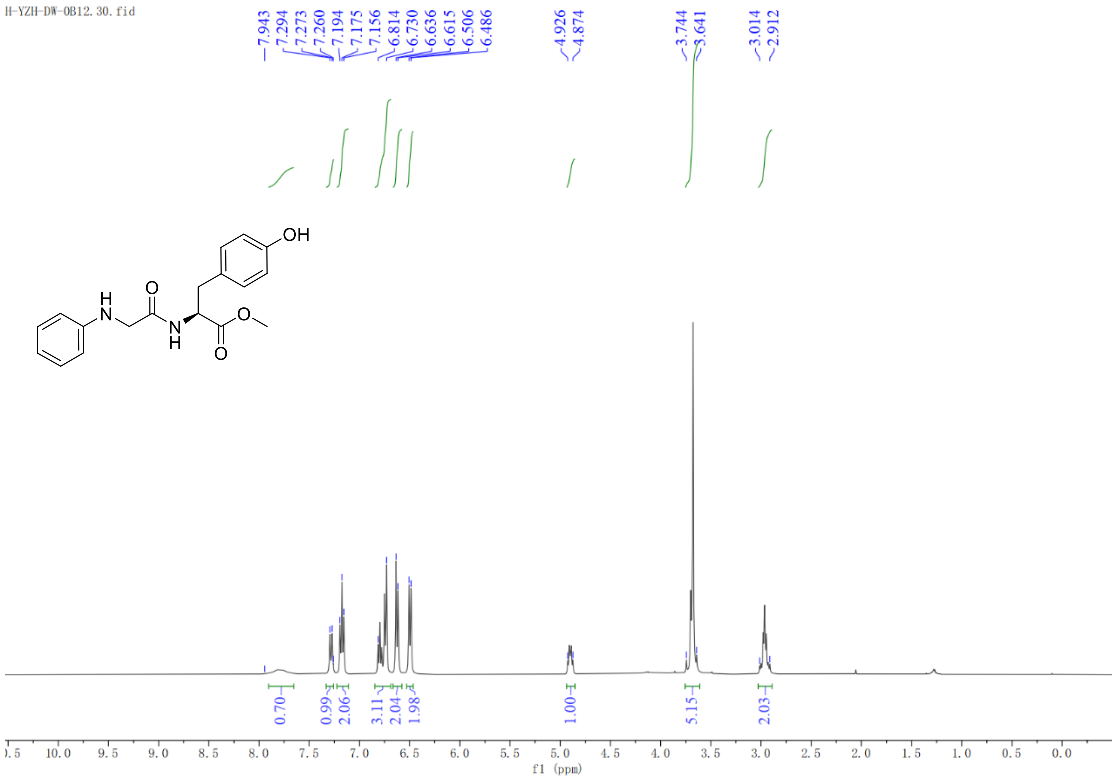

^{13}C NMR (101 MHz, $CDCl_3$) spectrum of **1q**

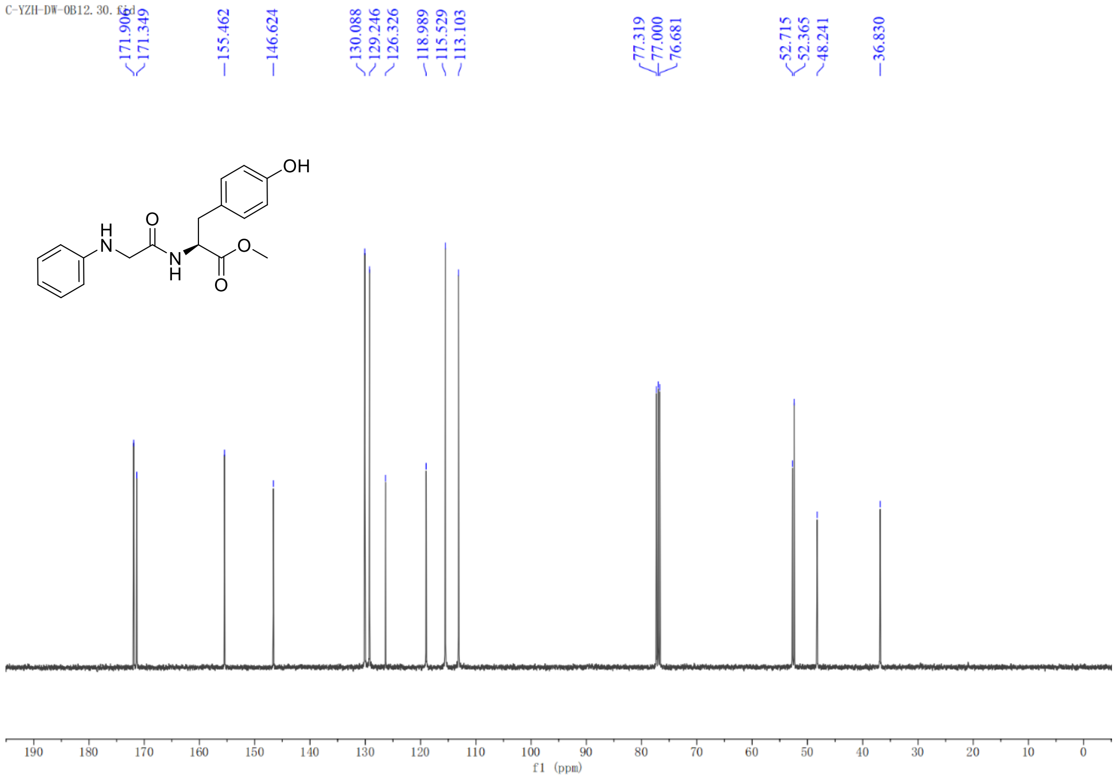

^{1}H NMR (400 MHz, $CDCl_3$) spectrum of **1r**

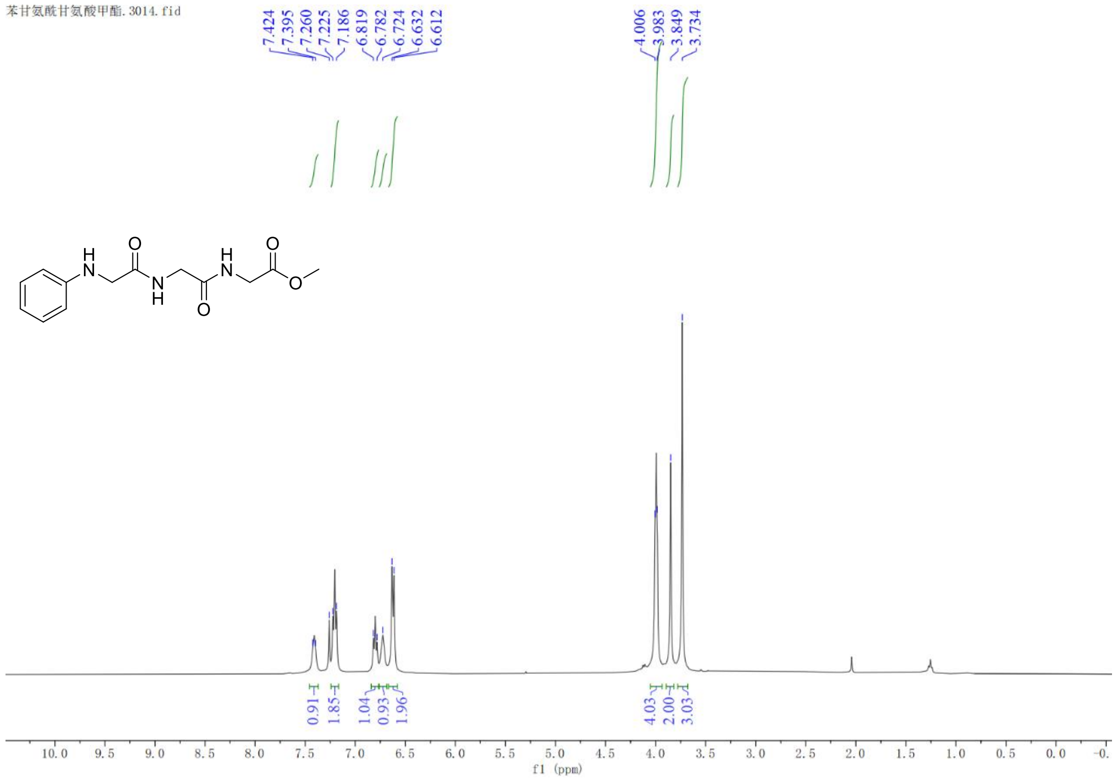

^{13}C NMR (101 MHz, $CDCl_3$) spectrum of **1r**

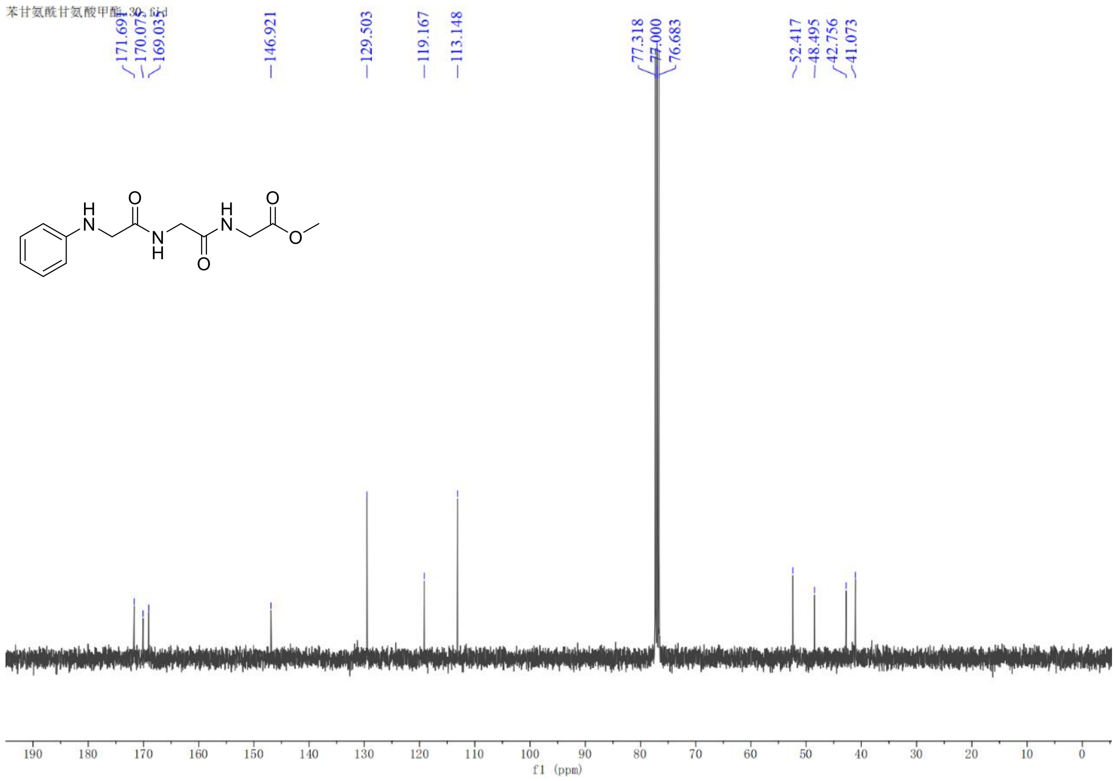

^{1}H NMR (400 MHz, $CDCl_3$) spectrum of **1s**

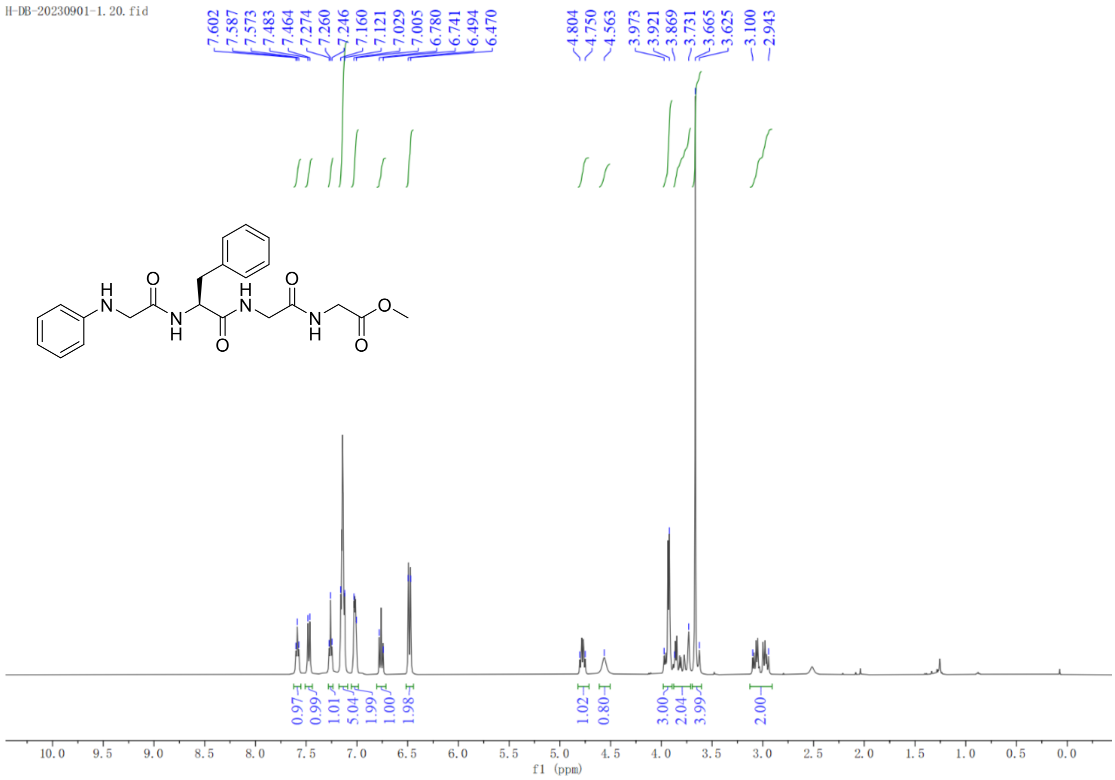

^{13}C NMR (101 MHz, $CDCl_3$) spectrum of **1s**

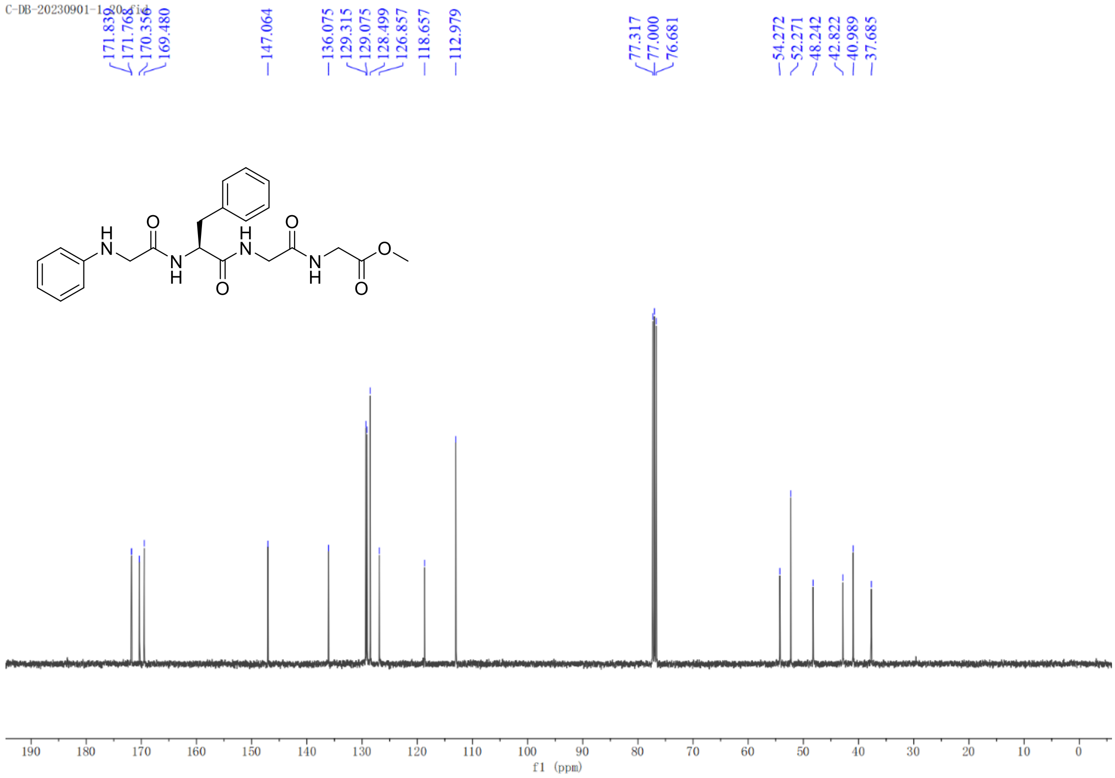

^{1}**H NMR** (400 MHz, $CDCl_3$) spectrum of **1t**

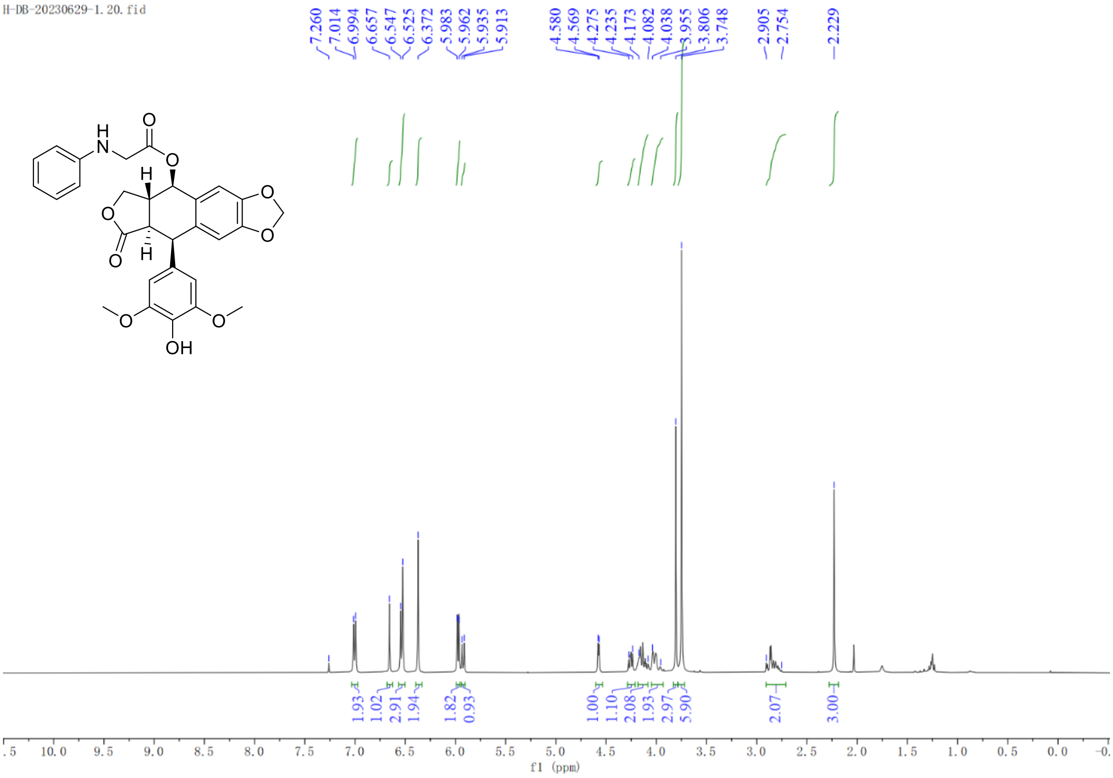

^{13}**C NMR** (101 MHz, $CDCl_3$) spectrum of **1t**

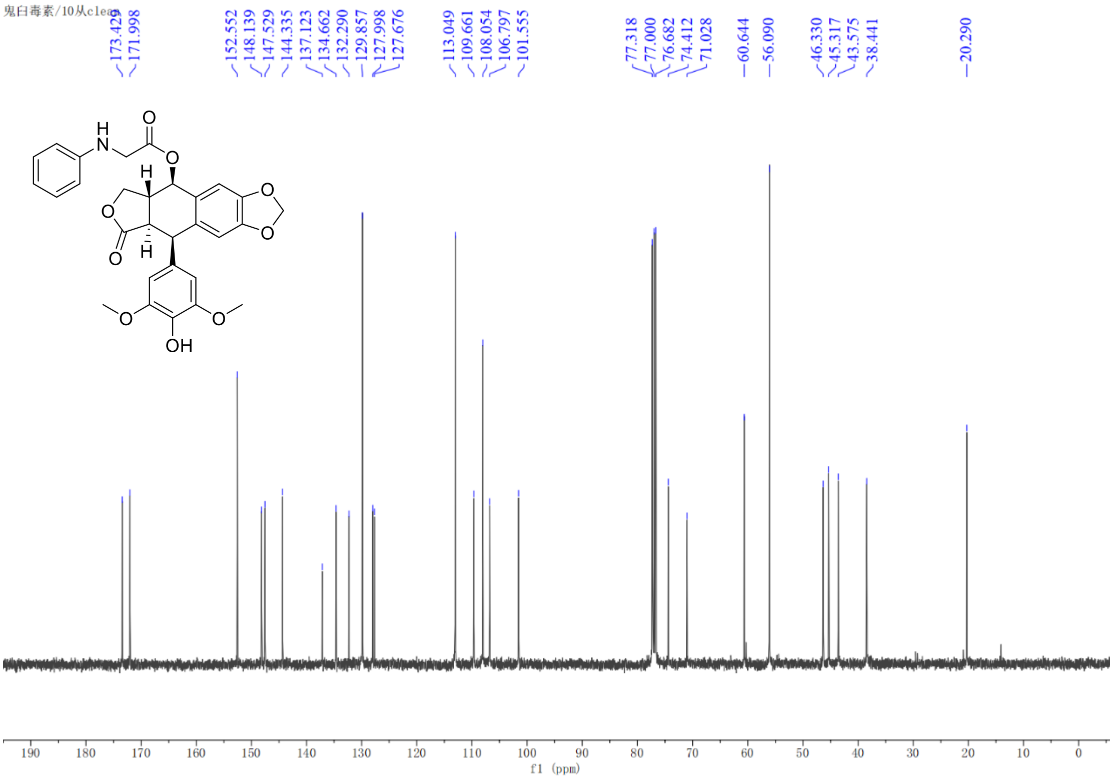

^{1}H NMR (400 MHz, $CDCl_3$) spectrum of **1u**

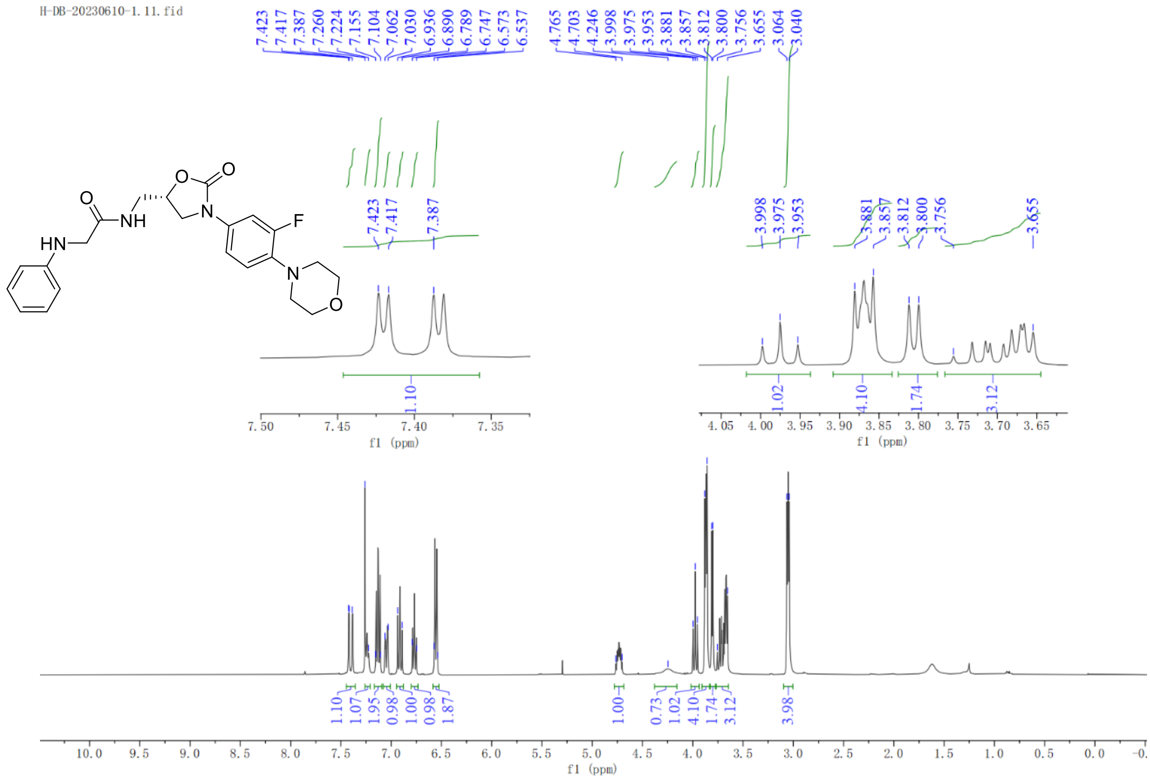

^{13}C NMR (101 MHz, $CDCl_3$) spectrum of **1u**

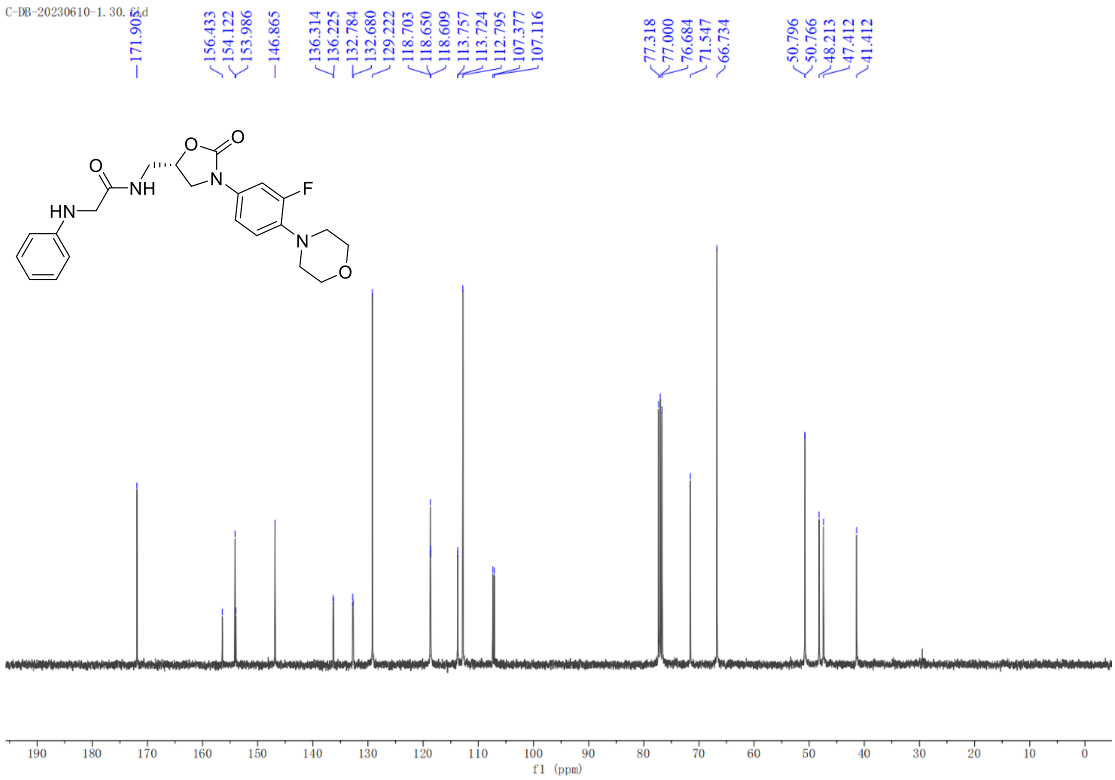

^{19}F NMR (376 MHz, $CDCl_3$) spectrum of **1u**

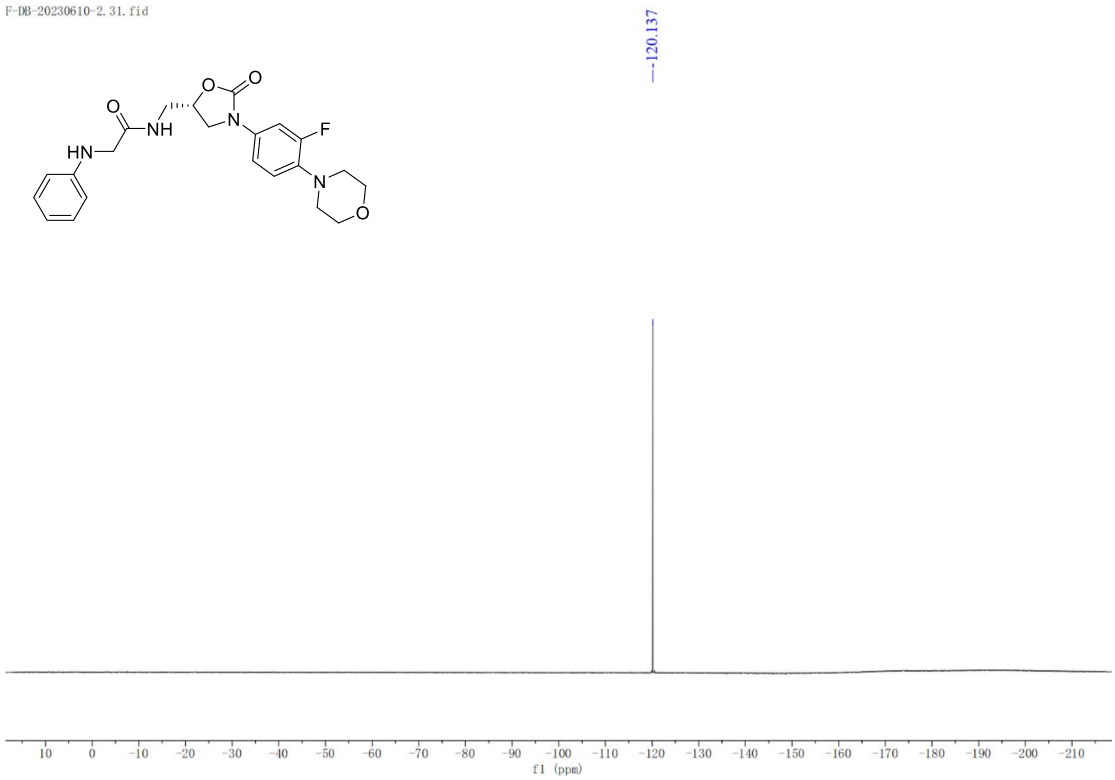

^{1}H NMR (400 MHz, $CDCl_3$) spectrum of **4**

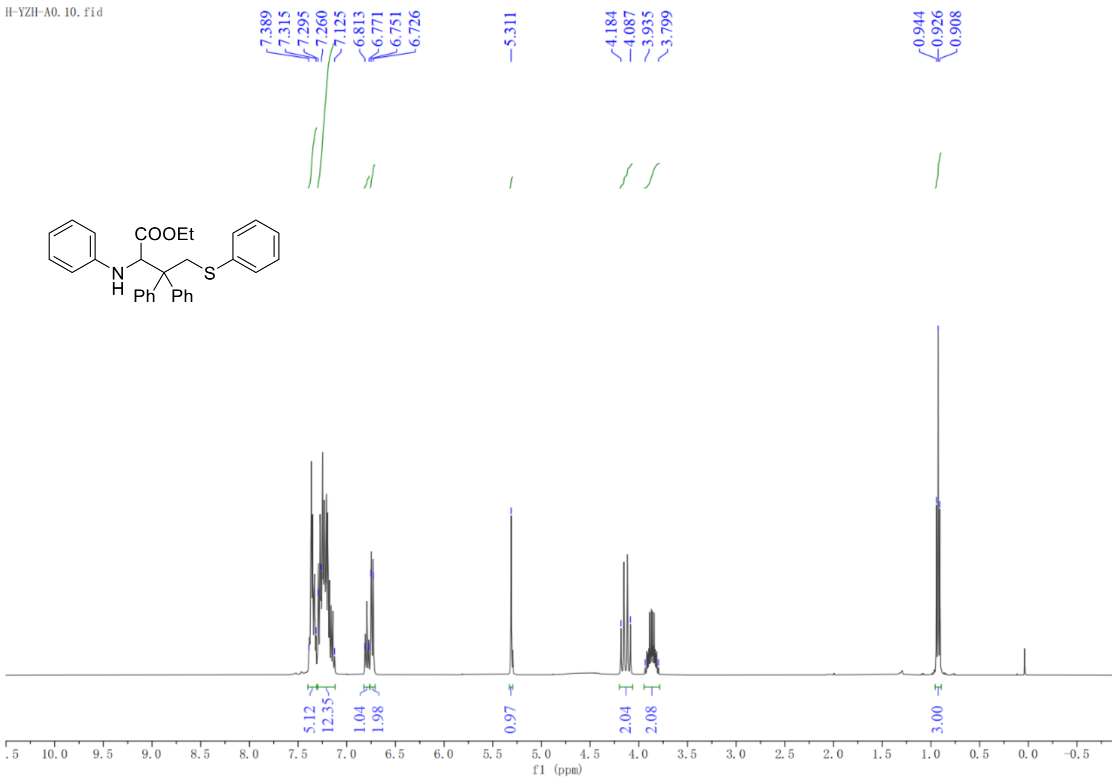

^{13}C NMR (101 MHz, $CDCl_3$) spectrum of **4**

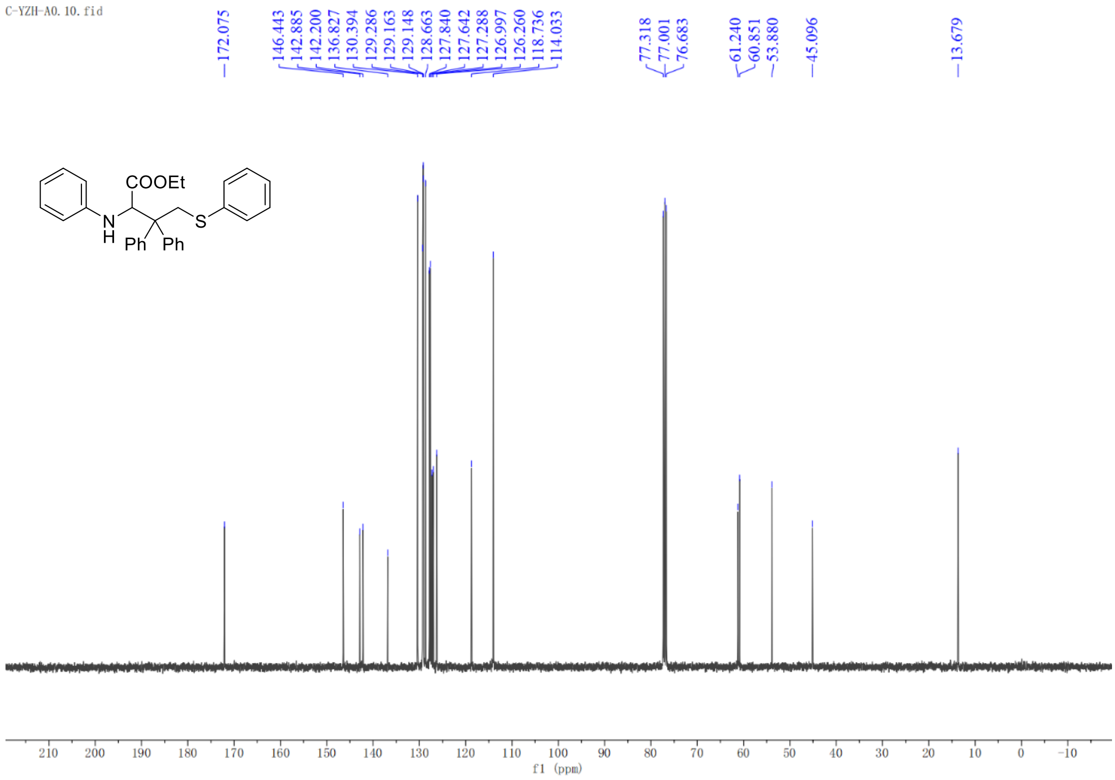

^{1}H NMR (400 MHz, $CDCl_3$) spectrum of **5**

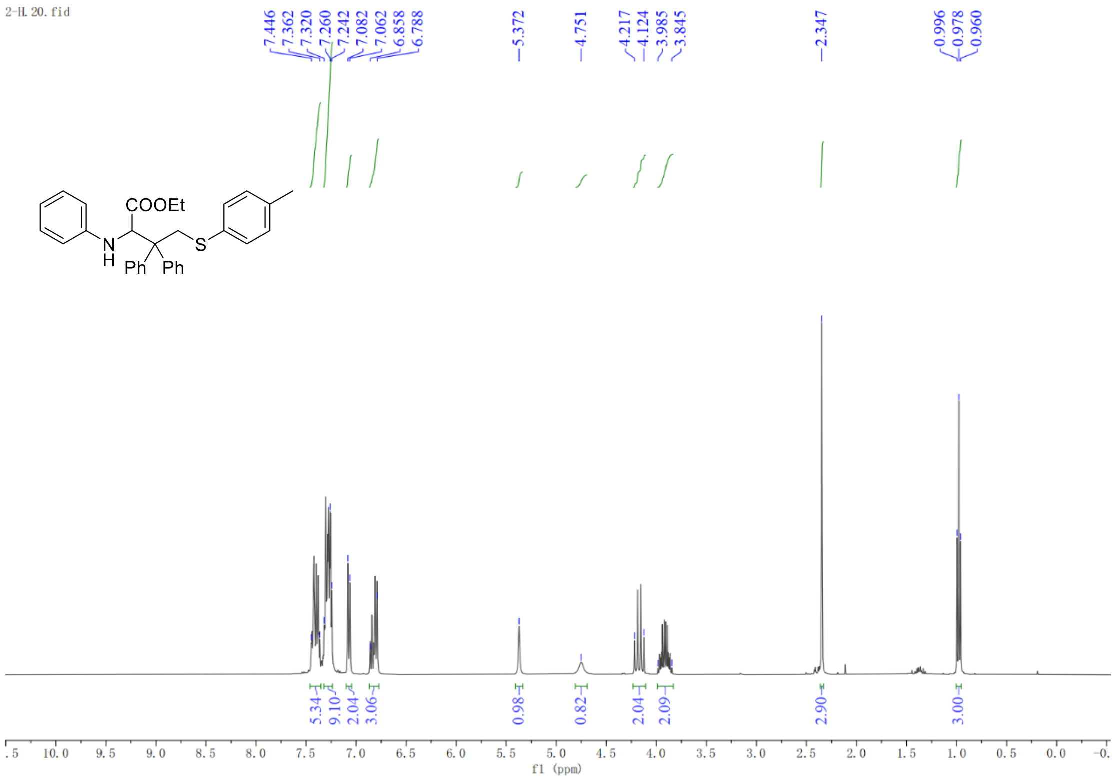

^{13}C NMR (101 MHz, $CDCl_3$) spectrum of **5**

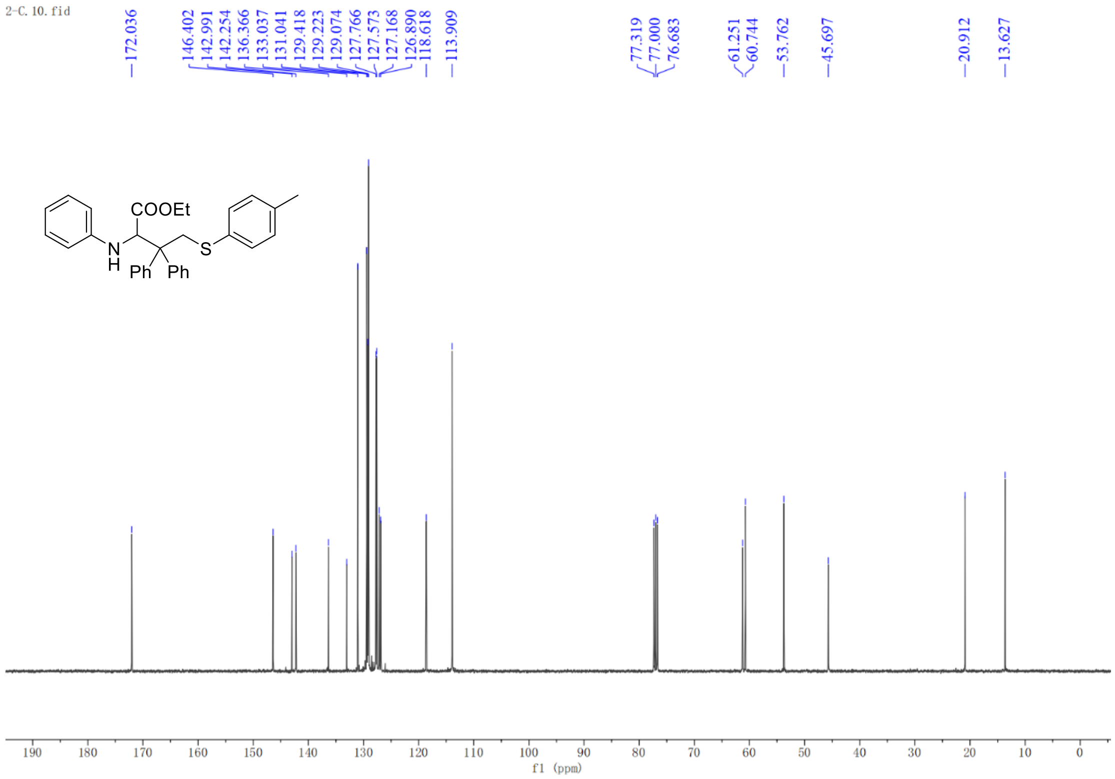

^{1}H NMR (400 MHz, $CDCl_3$) spectrum of **6**

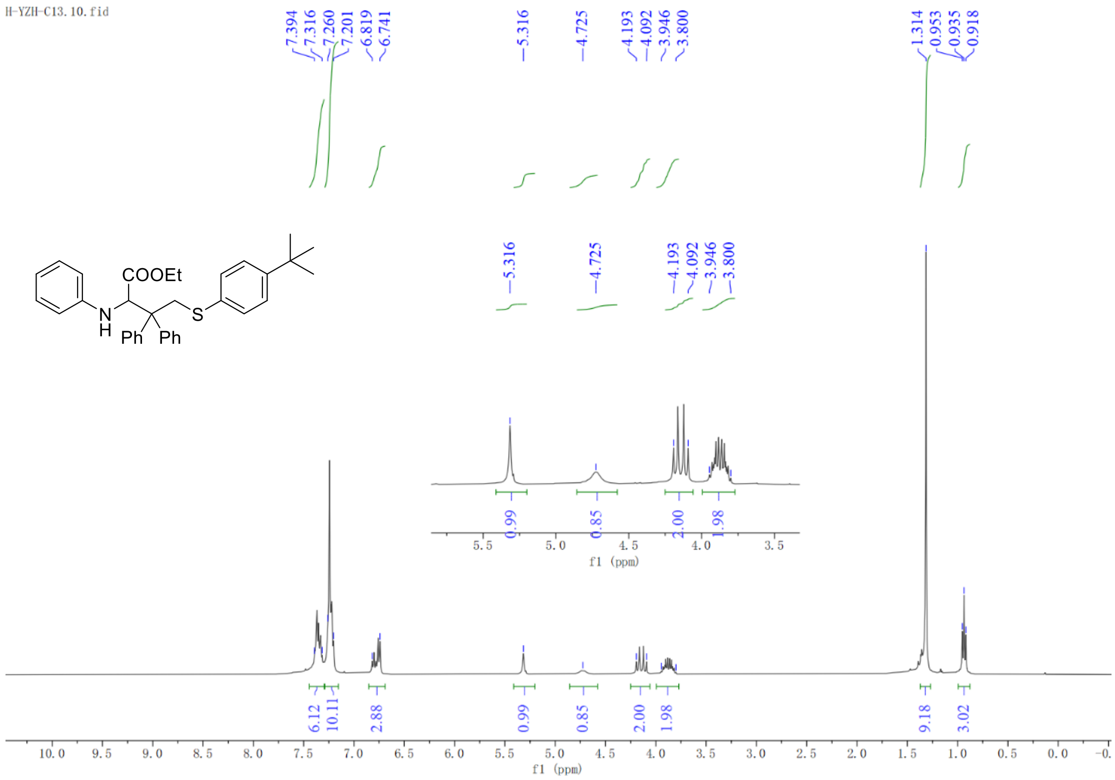

^{13}C NMR (101 MHz, $CDCl_3$) spectrum of **6**

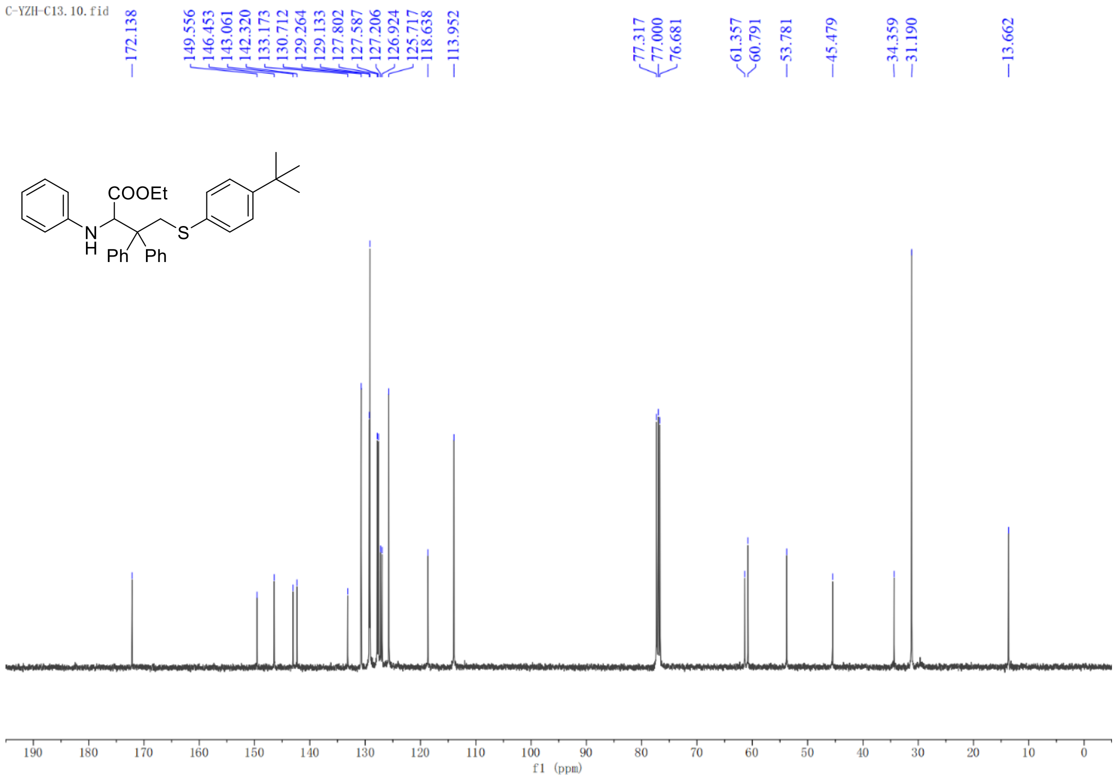

^{1}H NMR (400 MHz, $CDCl_3$) spectrum of **7**

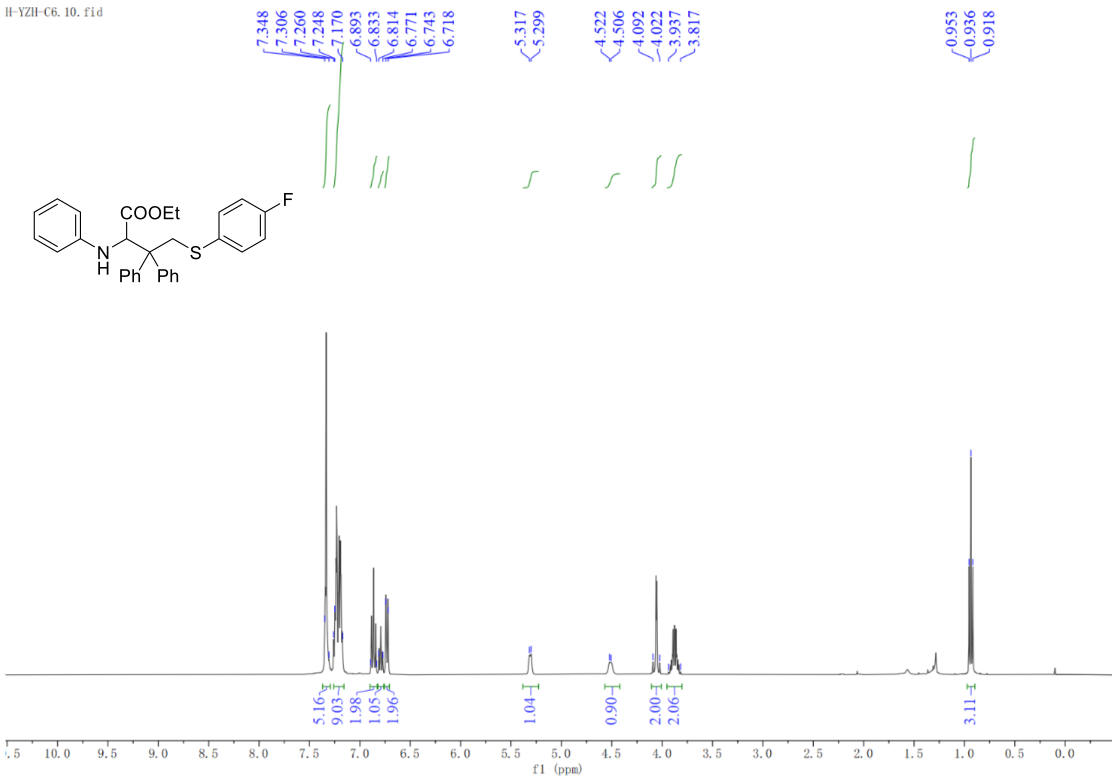

^{13}C NMR (101 MHz, $CDCl_3$) spectrum of **7**

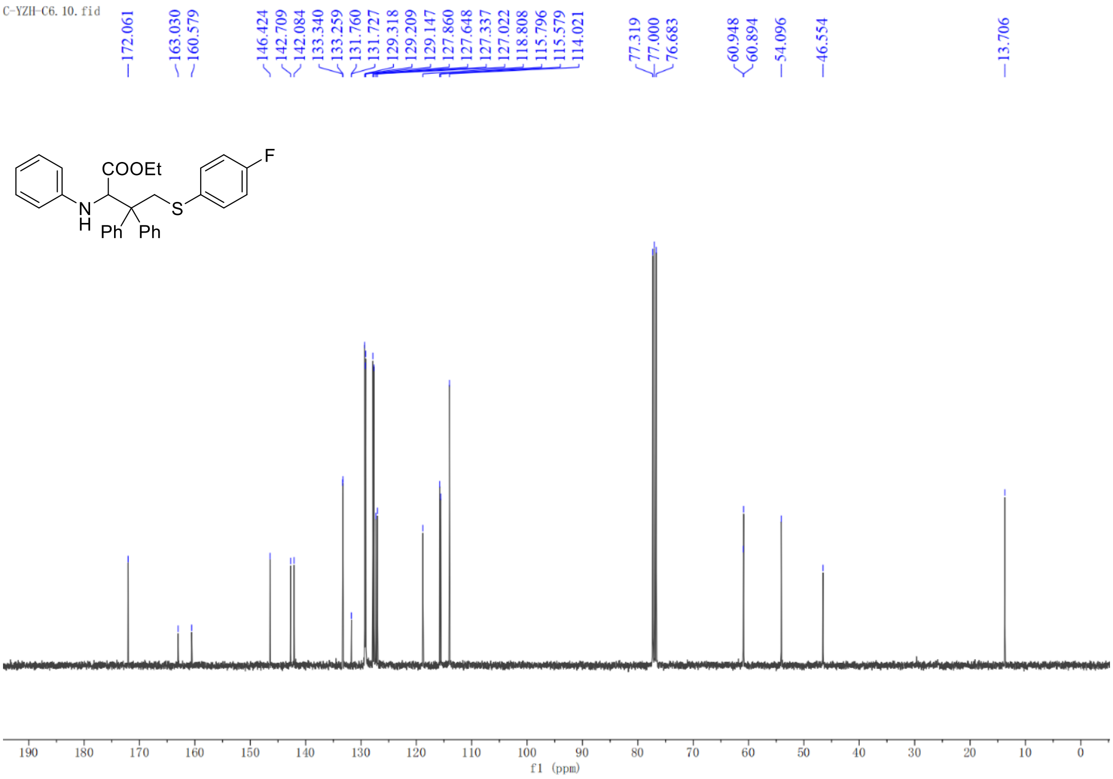

^{19}F NMR (376 MHz, $CDCl_3$) spectrum of **7**

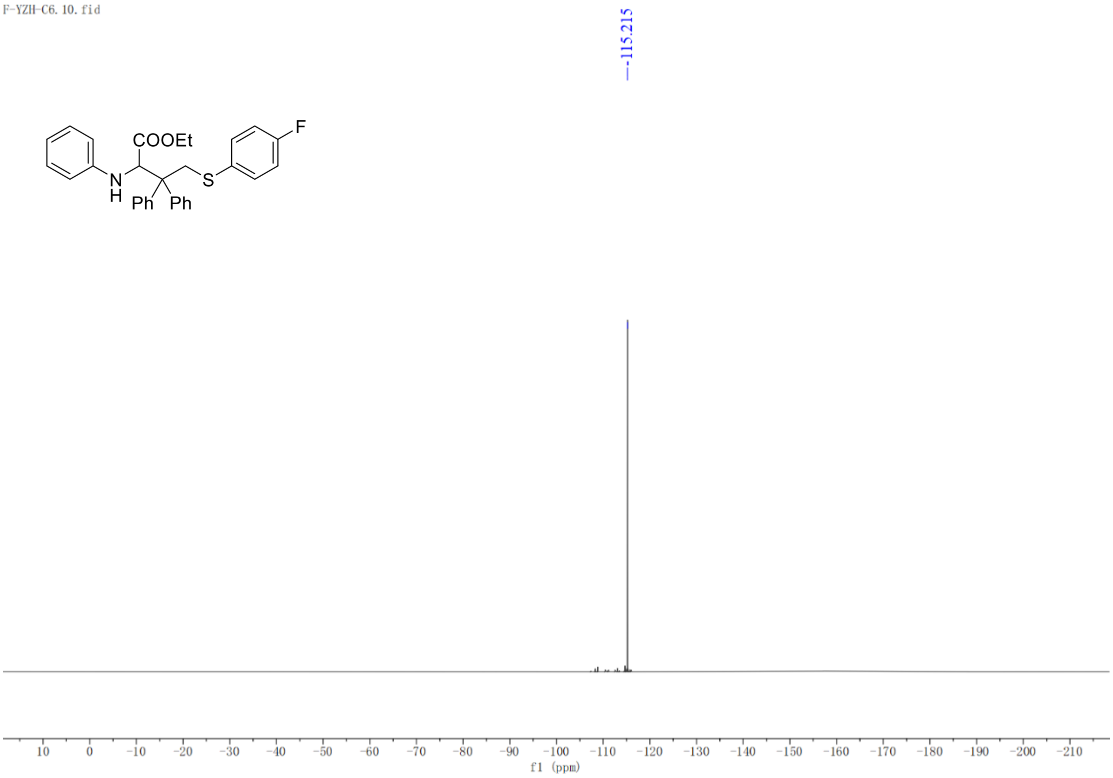

^{1}H NMR (400 MHz, $CDCl_3$) spectrum of **8**

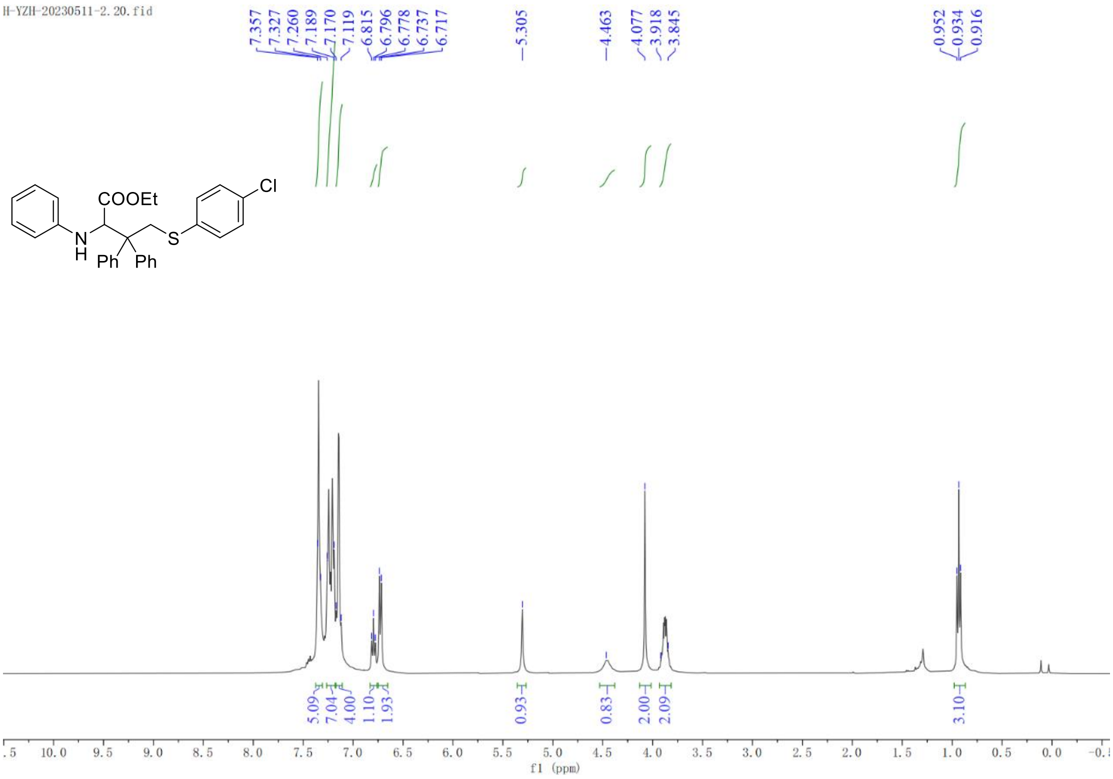

^{13}C NMR (101 MHz, $CDCl_3$) spectrum of **8**

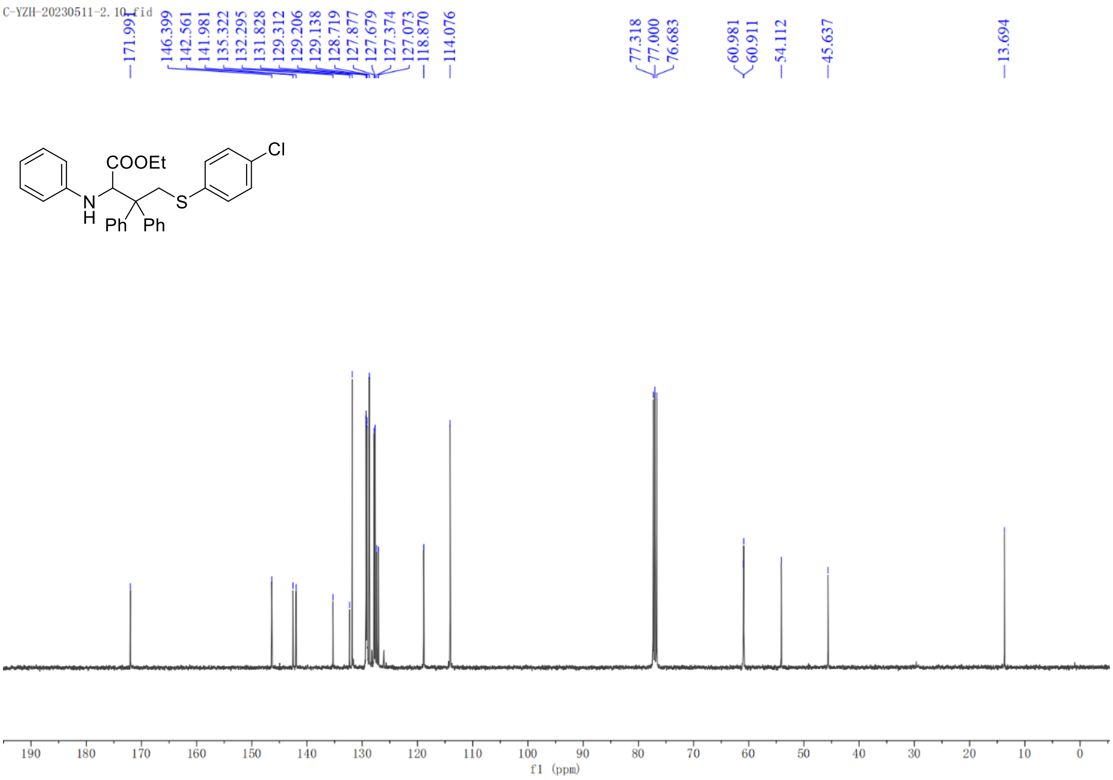

^{1}H NMR (400 MHz, CDCl$_3$) spectrum of **9**

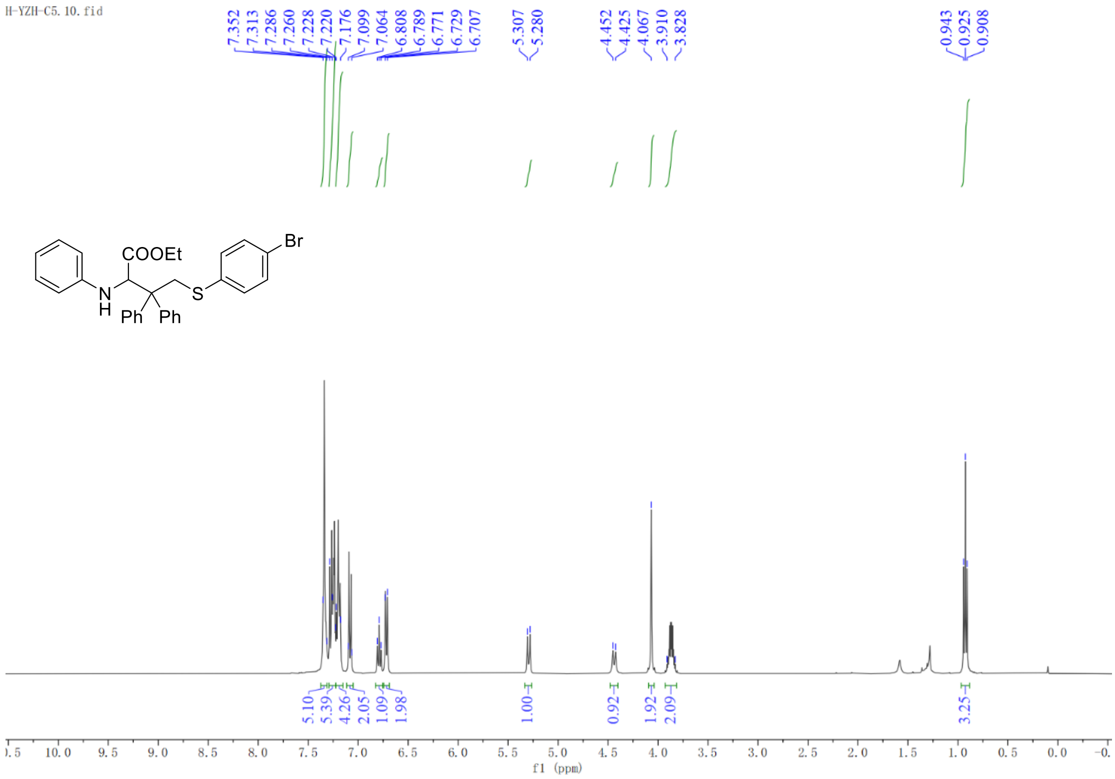

^{13}C NMR (101 MHz, CDCl$_3$) spectrum of **9**

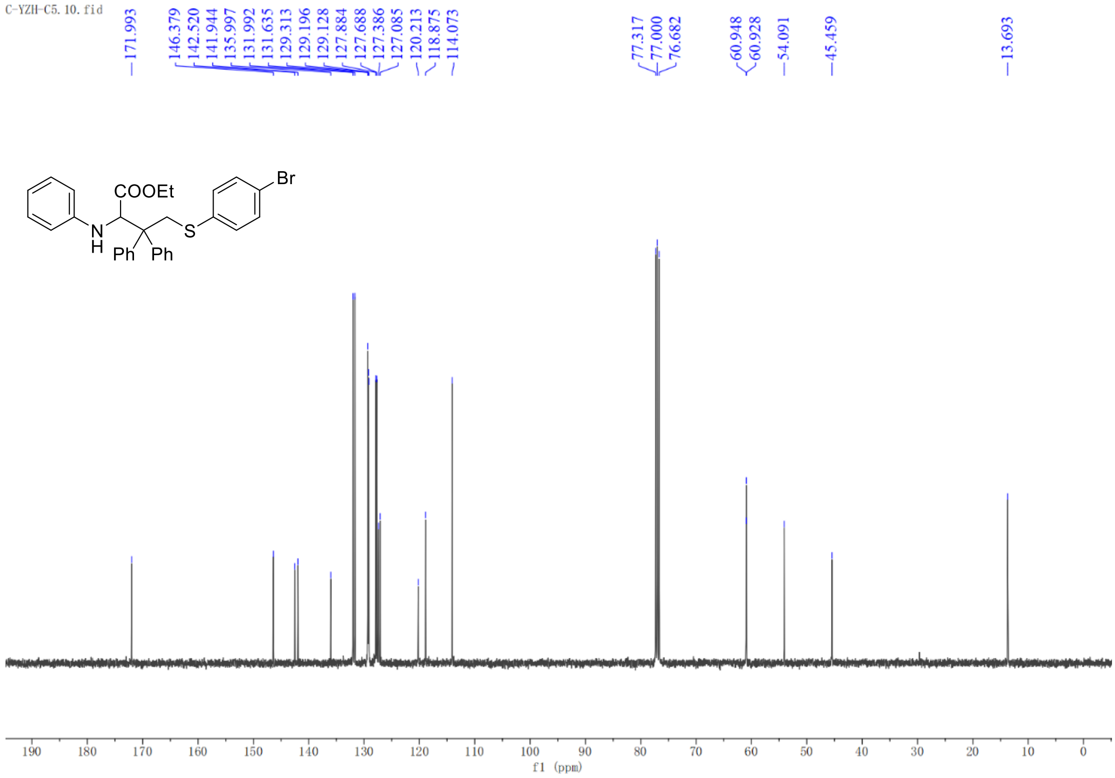

^{1}H NMR (400 MHz, $CDCl_3$) spectrum of **10**

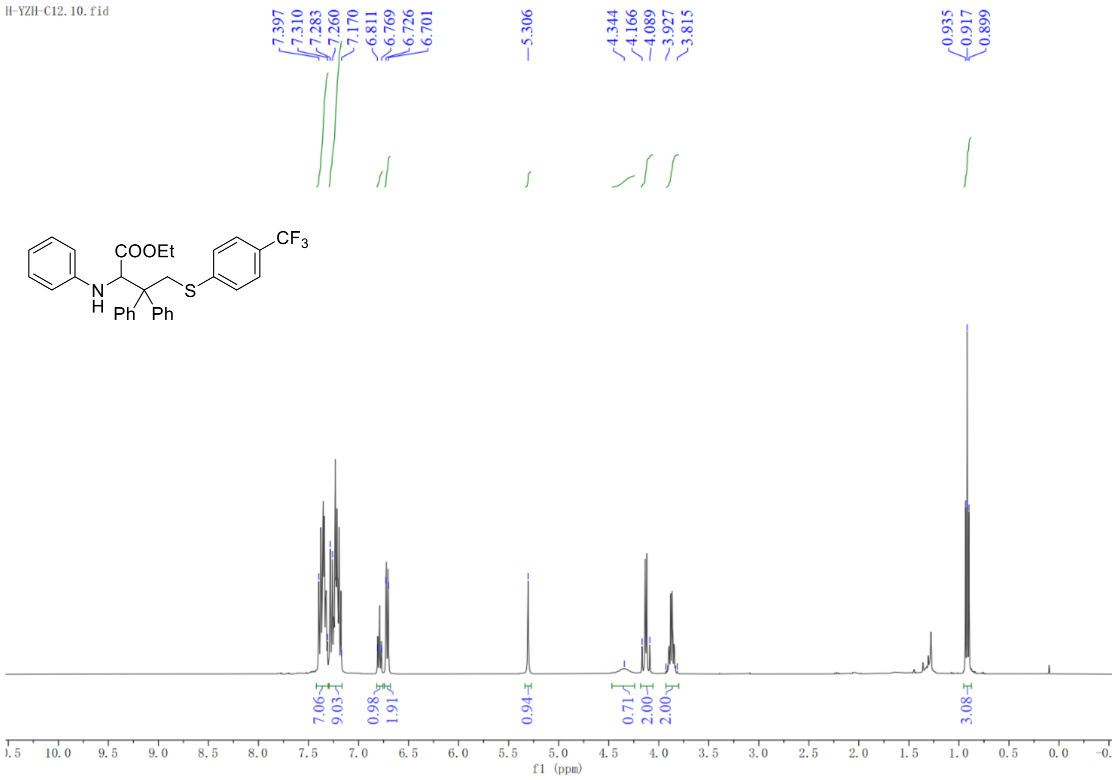

^{13}C NMR (101 MHz, $CDCl_3$) spectrum of **10**

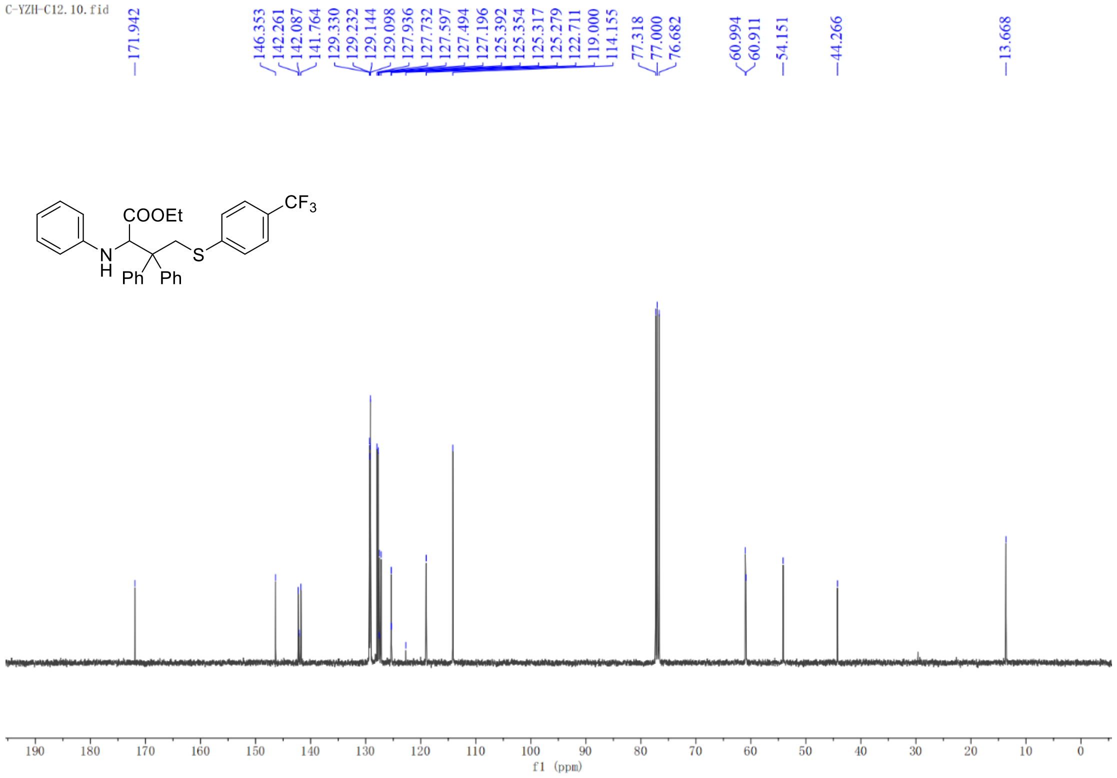

^{19}F NMR (376 MHz, $CDCl_3$) spectrum of **10**

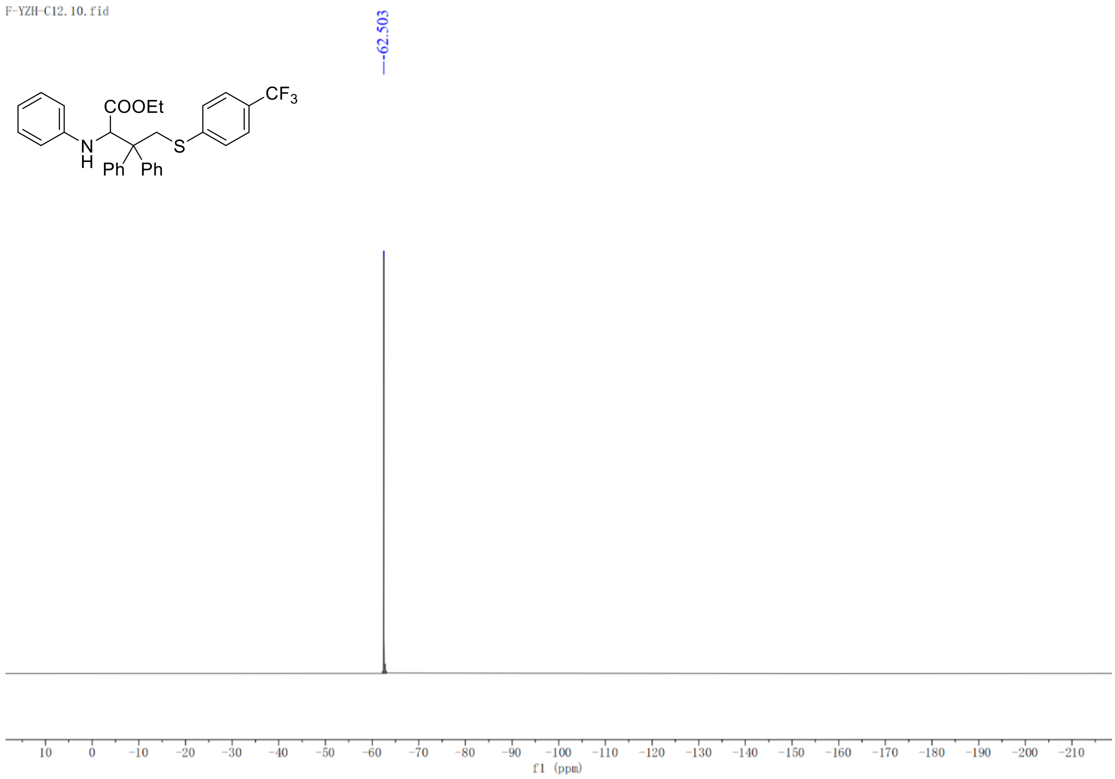

^{1}H NMR (400 MHz, $CDCl_3$) spectrum of **11**

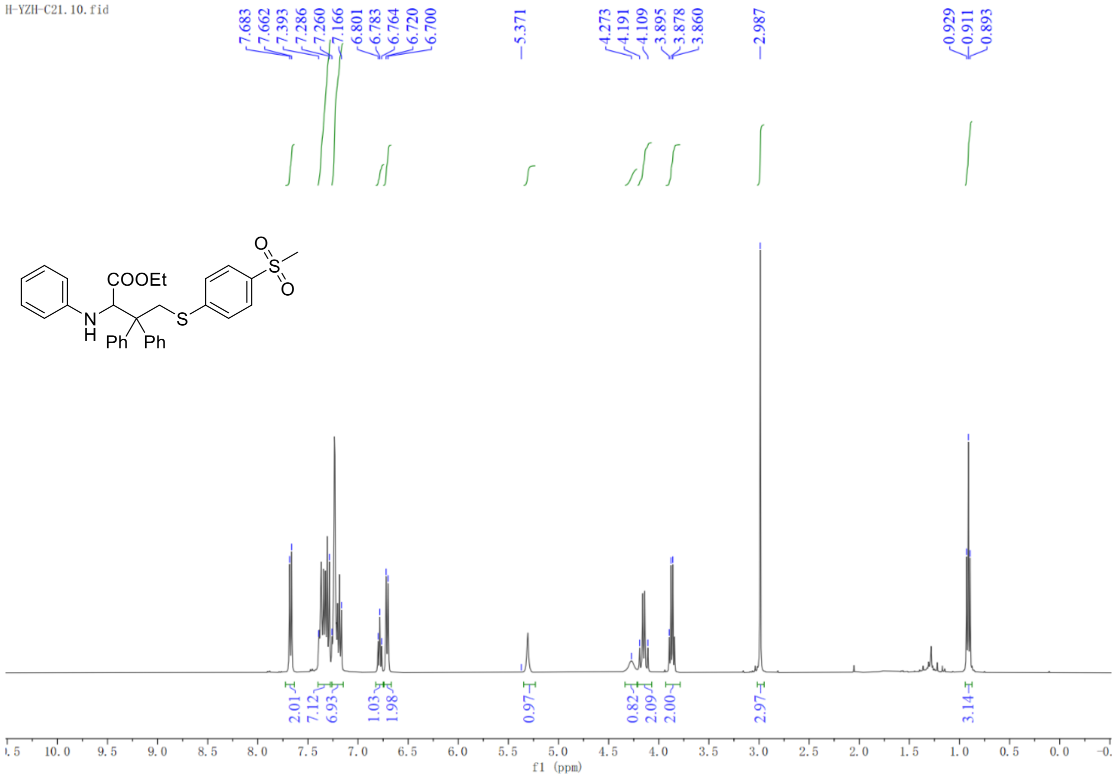

^{13}C NMR (101 MHz, $CDCl_3$) spectrum of **11**

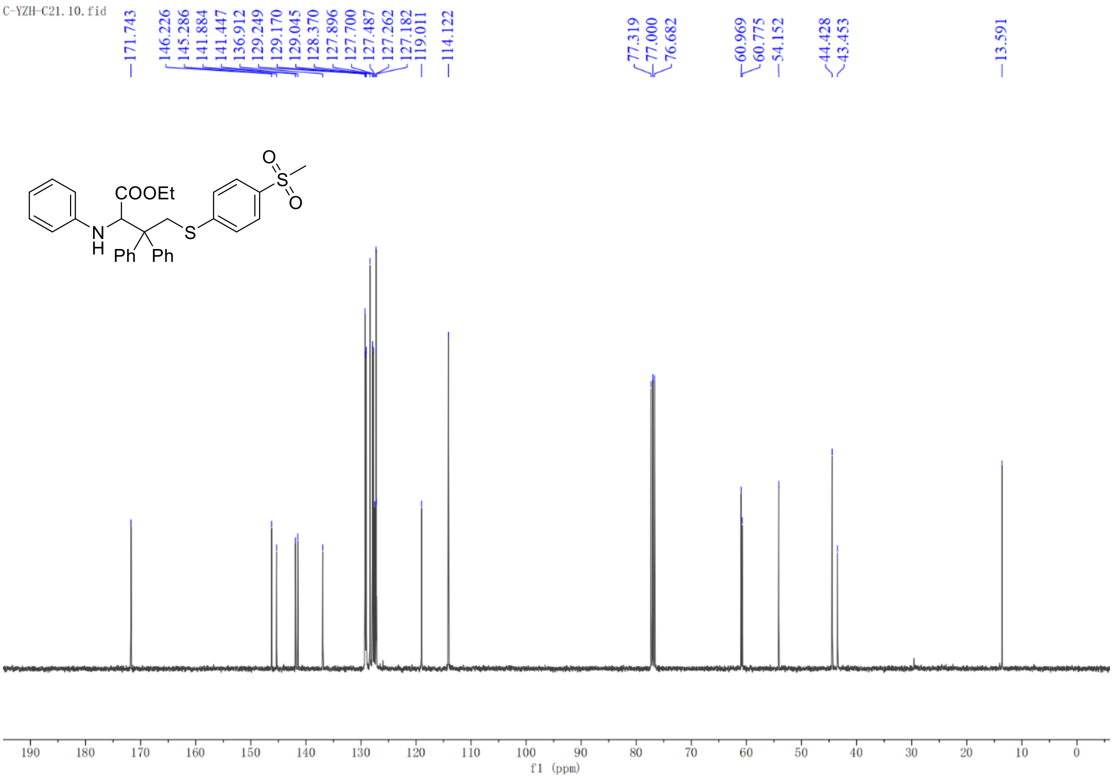

^{1}H NMR (400 MHz, $CDCl_3$) spectrum of **12**

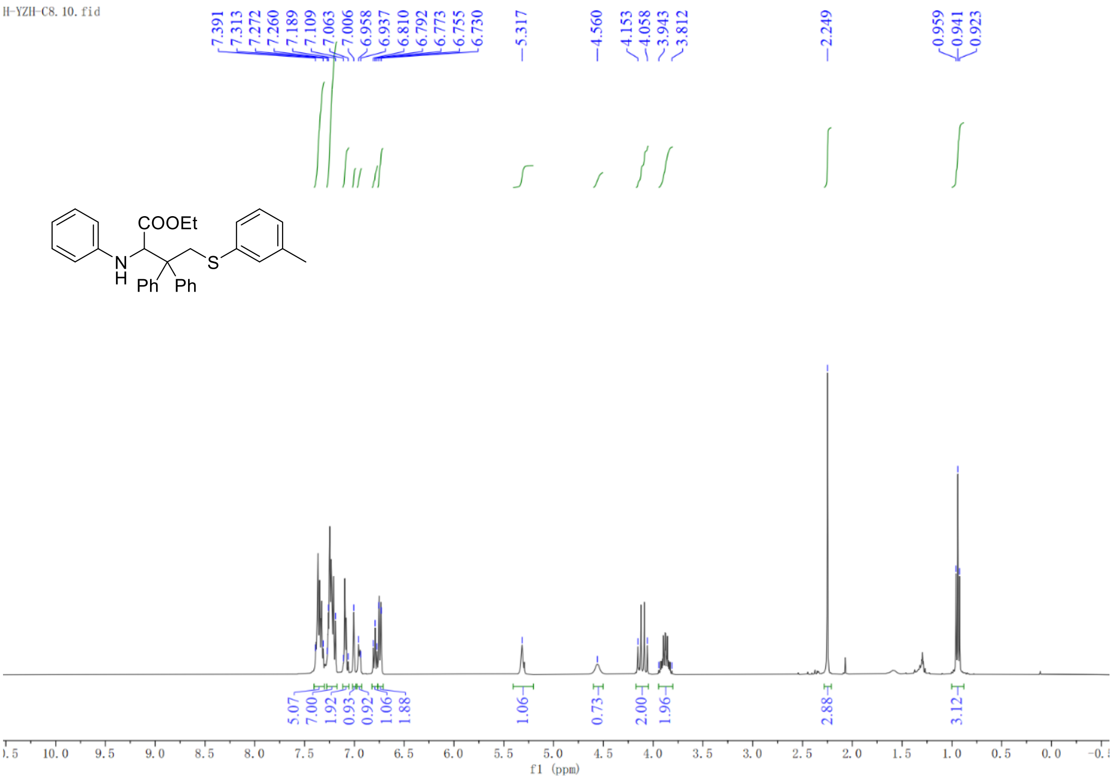

^{13}C NMR (101 MHz, $CDCl_3$) spectrum of **12**

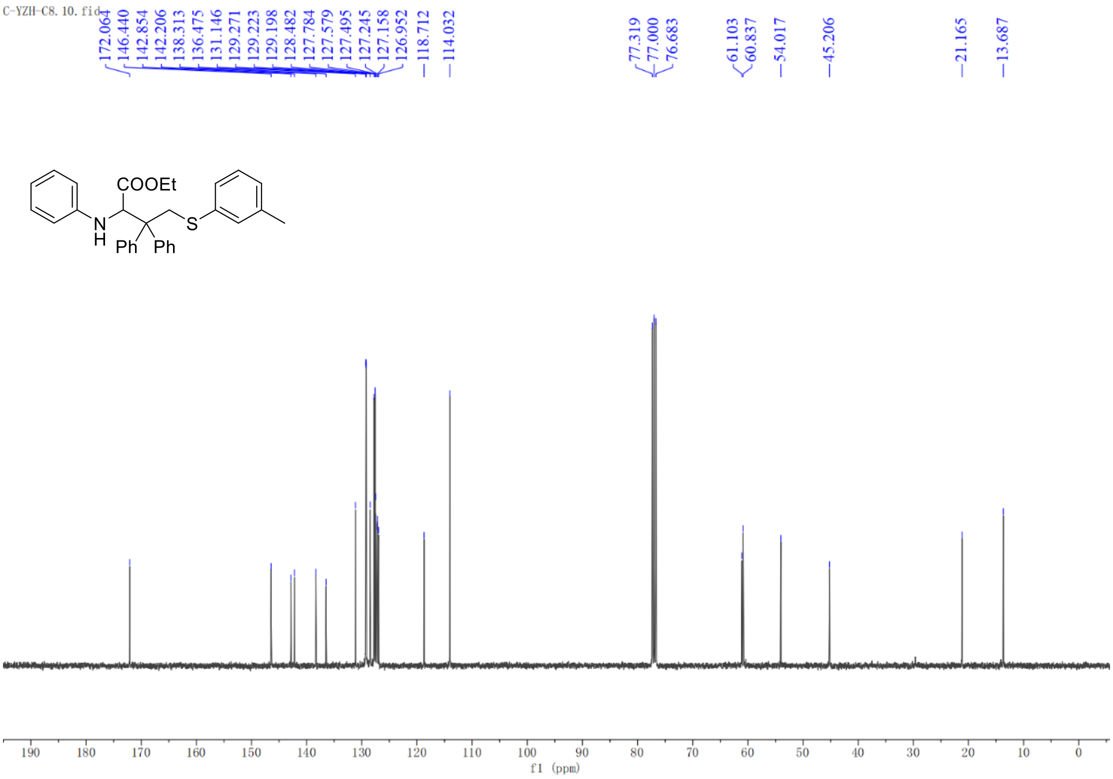

^{1}H NMR (400 MHz, $CDCl_3$) spectrum of **13**

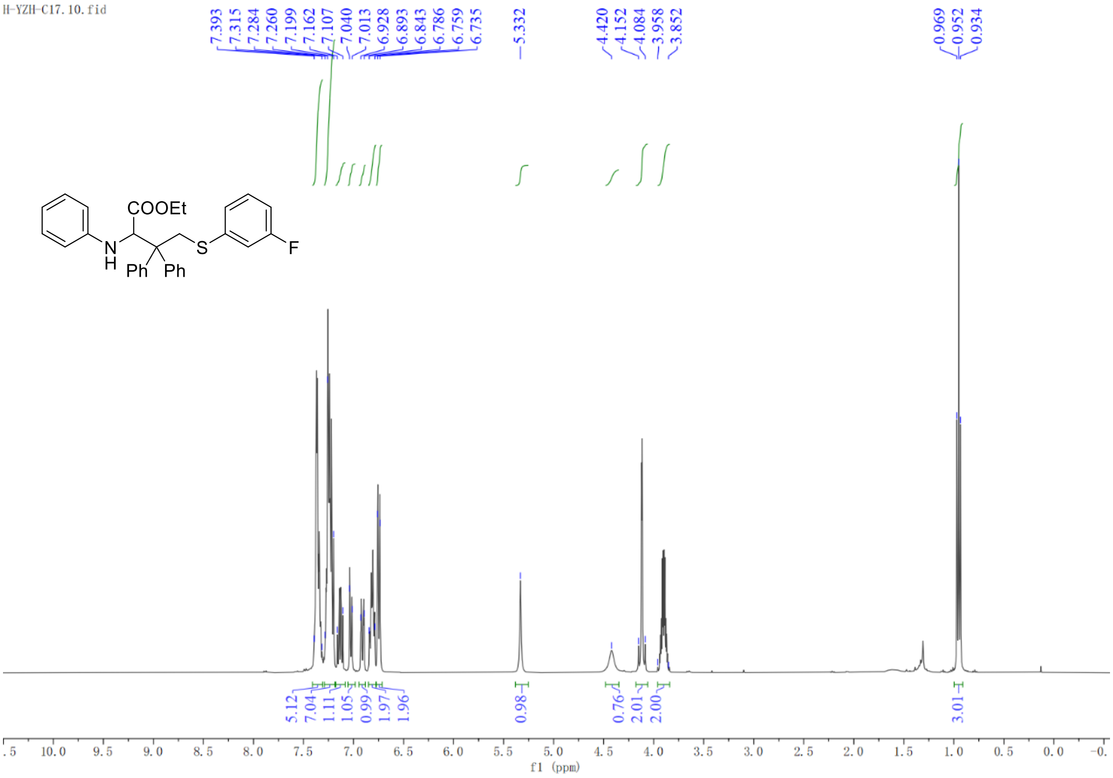

^{13}C NMR (101 MHz, $CDCl_3$) spectrum of **13**

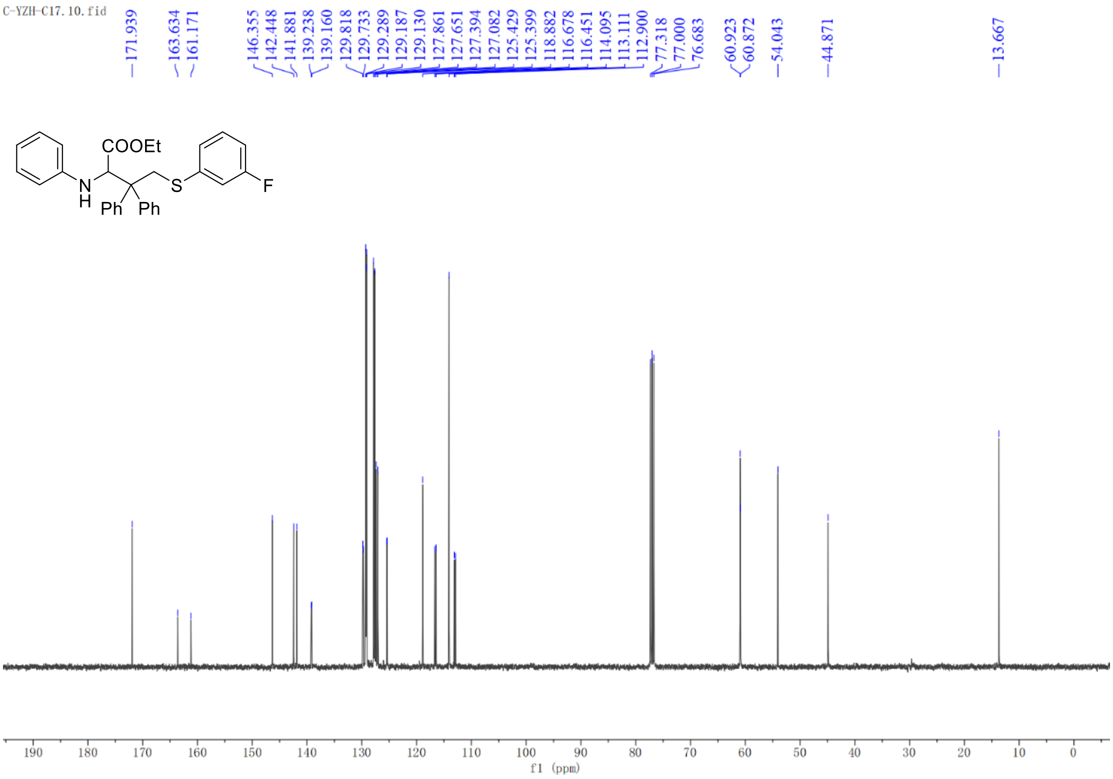

^{19}F NMR (376 MHz, $CDCl_3$) spectrum of **13**

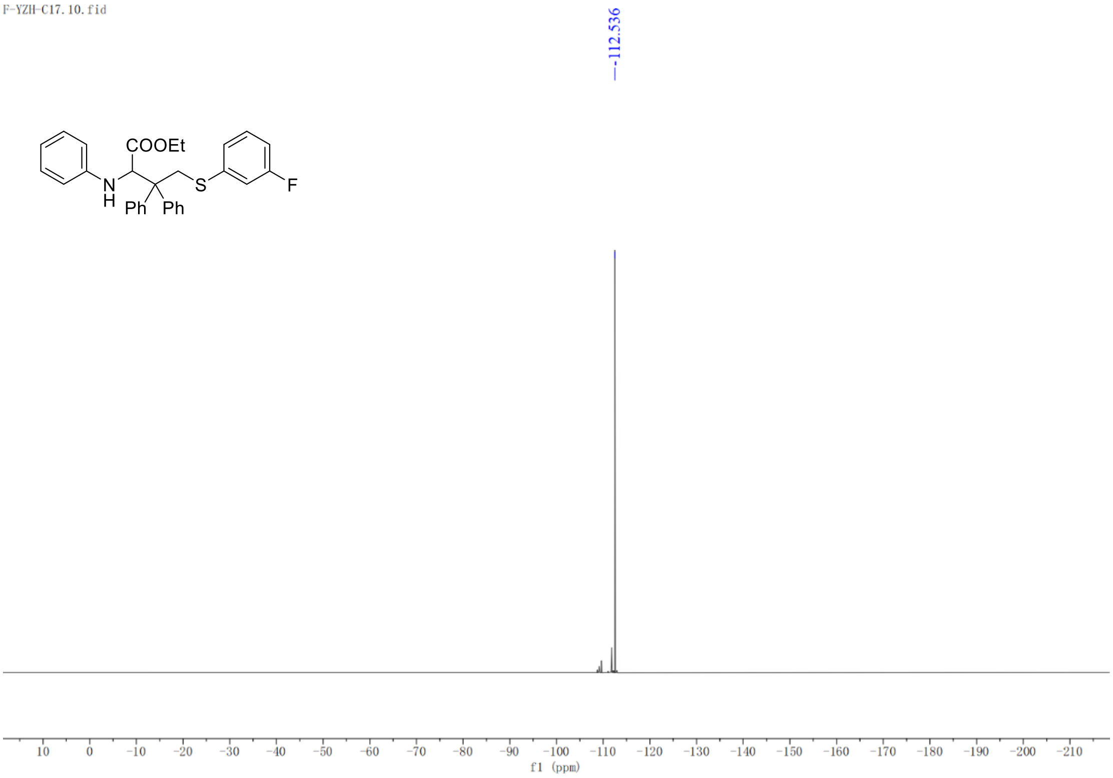

^{1}**H NMR** (400 MHz, $CDCl_3$) spectrum of **14**

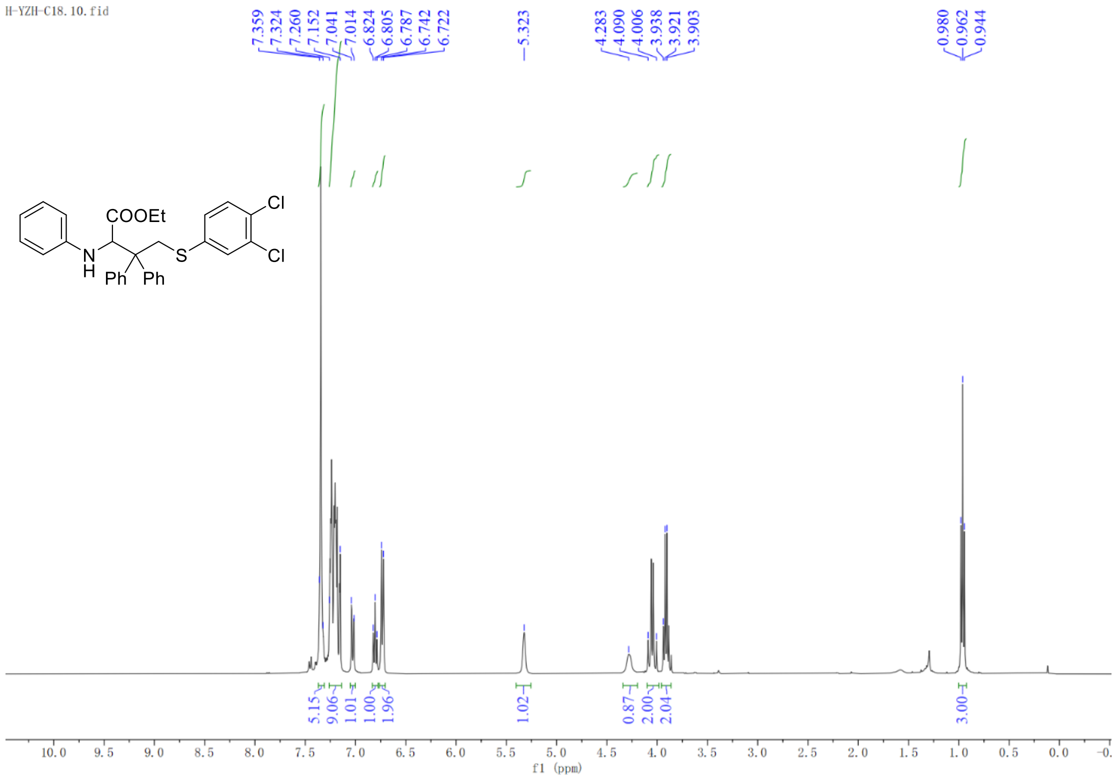

^{13}**C NMR** (101 MHz, $CDCl_3$) spectrum of **14**

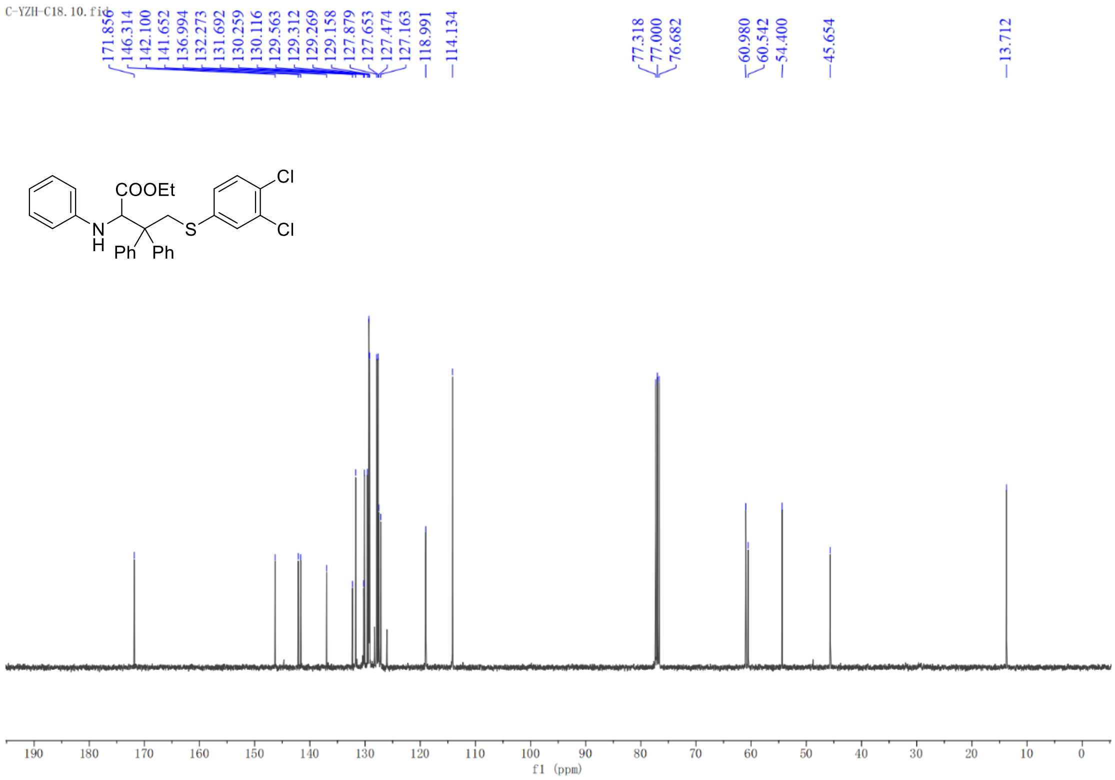

^{1}H NMR (400 MHz, $CDCl_3$) spectrum of **15**

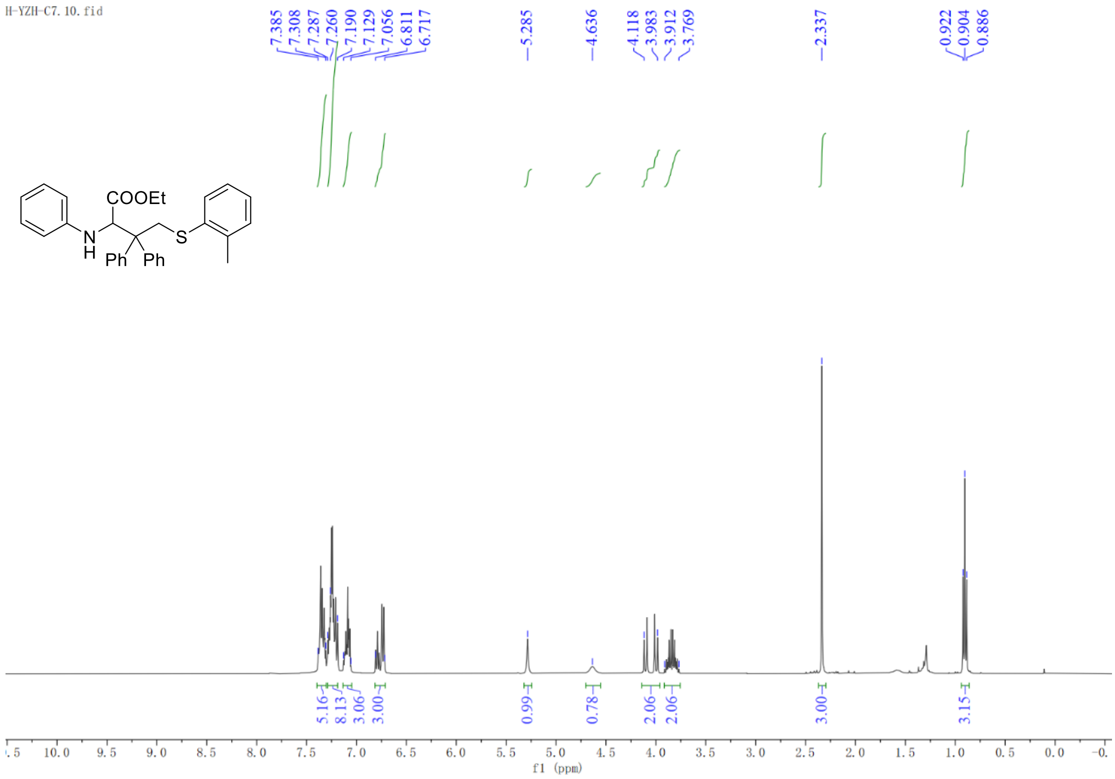

^{13}C NMR (101 MHz, $CDCl_3$) spectrum of **15**

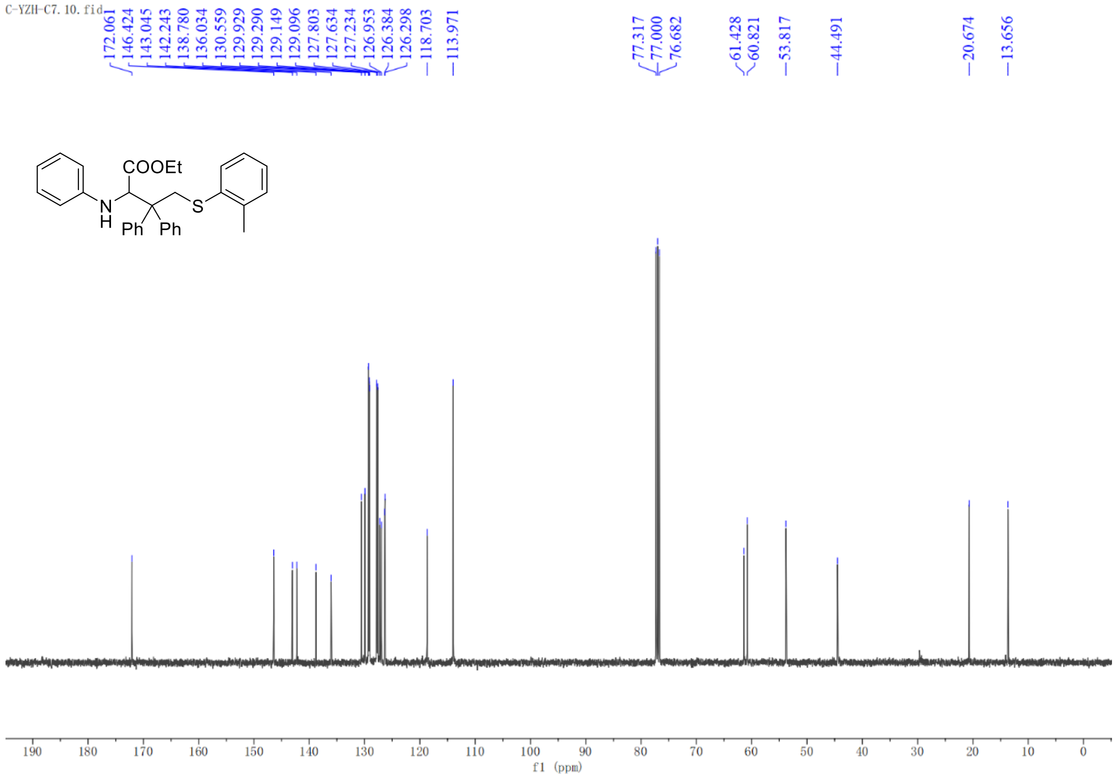

^{1}H NMR (400 MHz, $CDCl_3$) spectrum of **16**

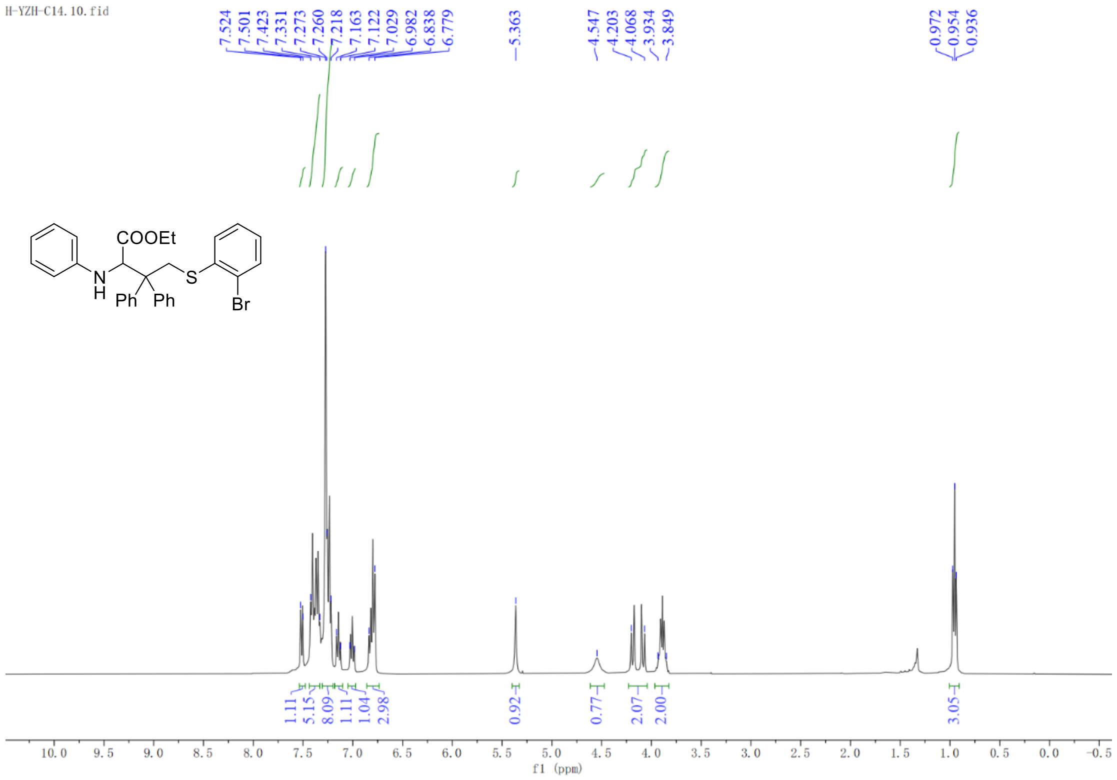

^{13}C NMR (101 MHz, $CDCl_3$) spectrum of **16**

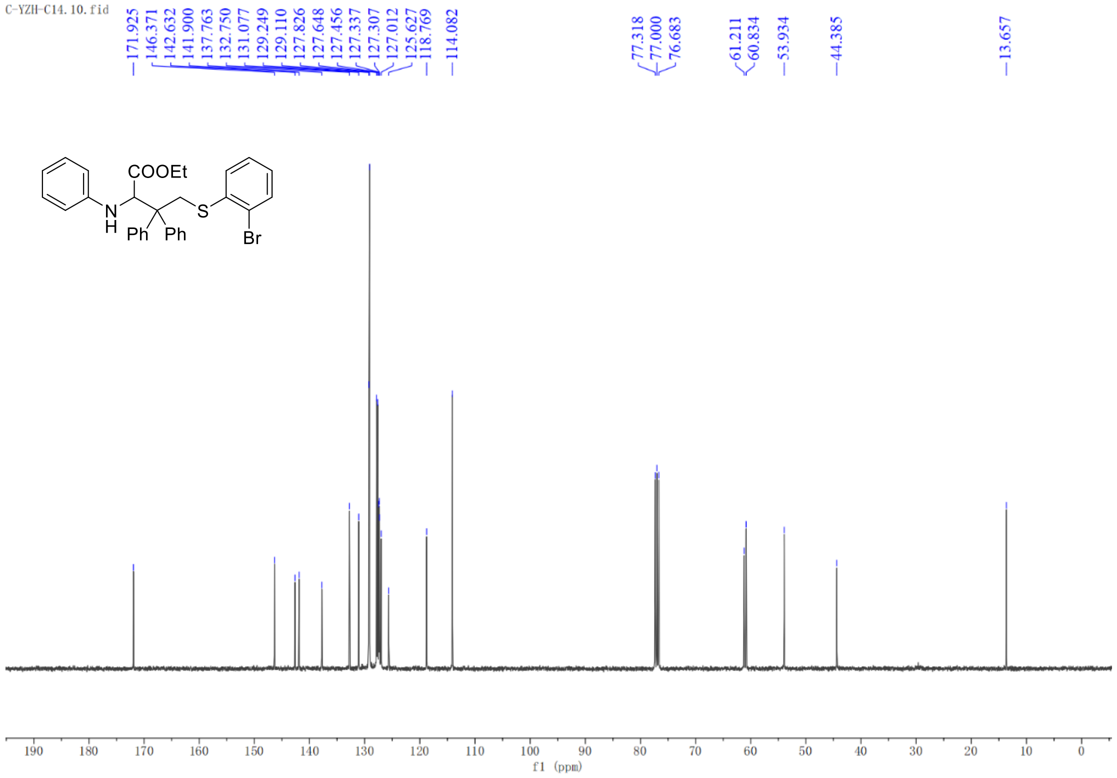

^{1}H NMR (400 MHz, $CDCl_3$) spectrum of **17**

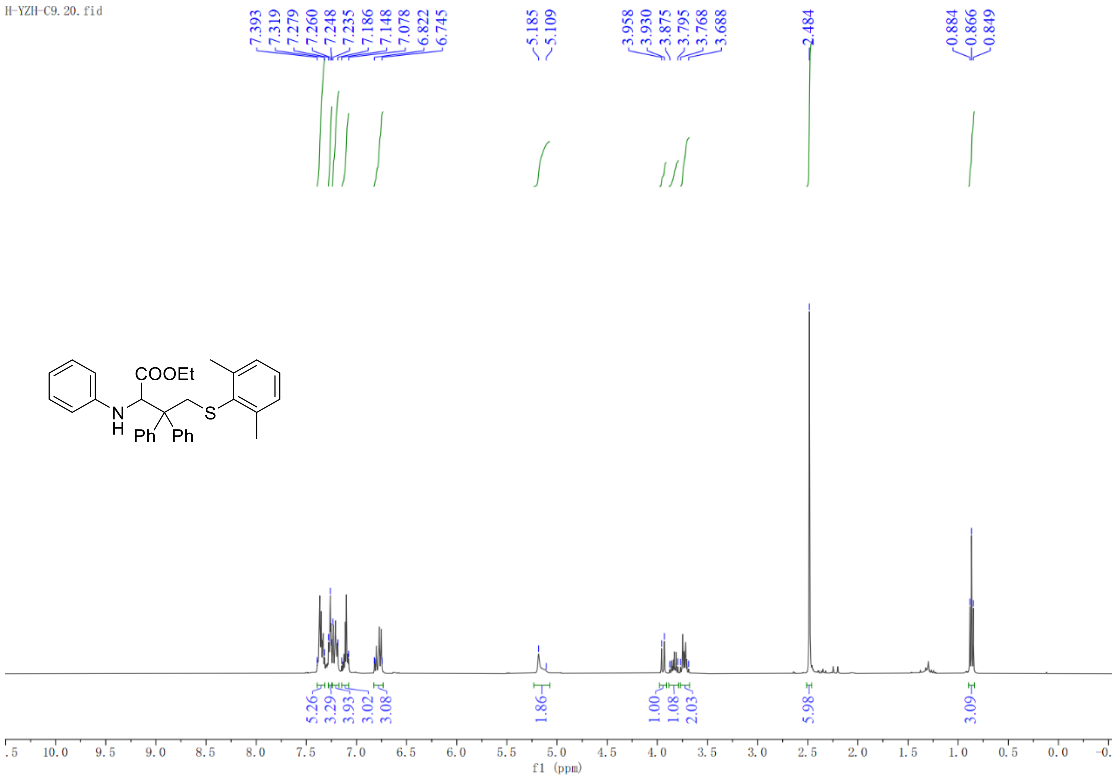

^{13}C NMR (101 MHz, $CDCl_3$) spectrum of **17**

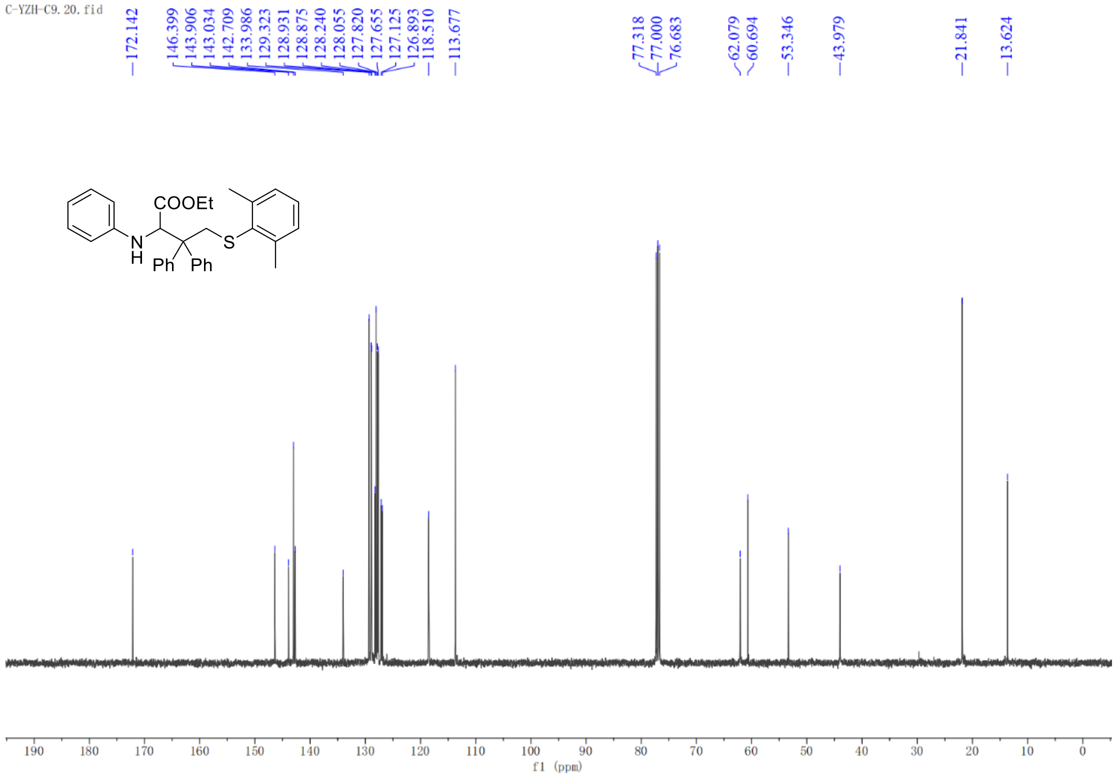

^{1}H NMR (400 MHz, $CDCl_3$) spectrum of **18**

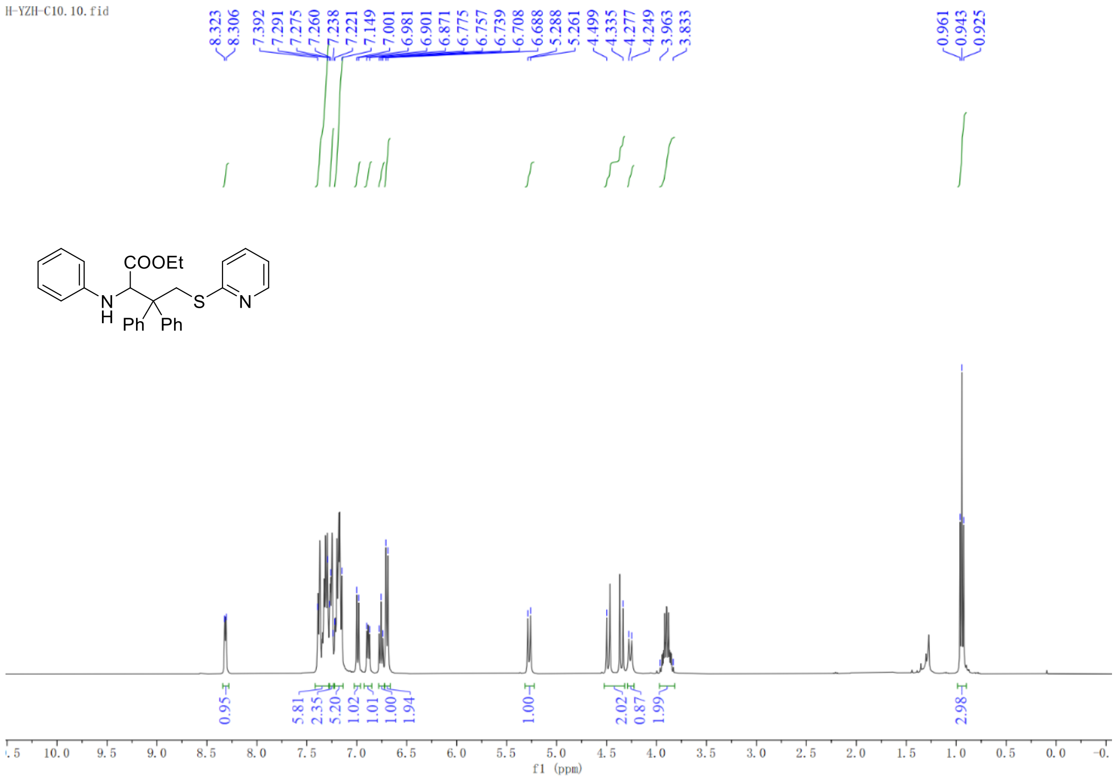

^{13}C NMR (101 MHz, $CDCl_3$) spectrum of **18**

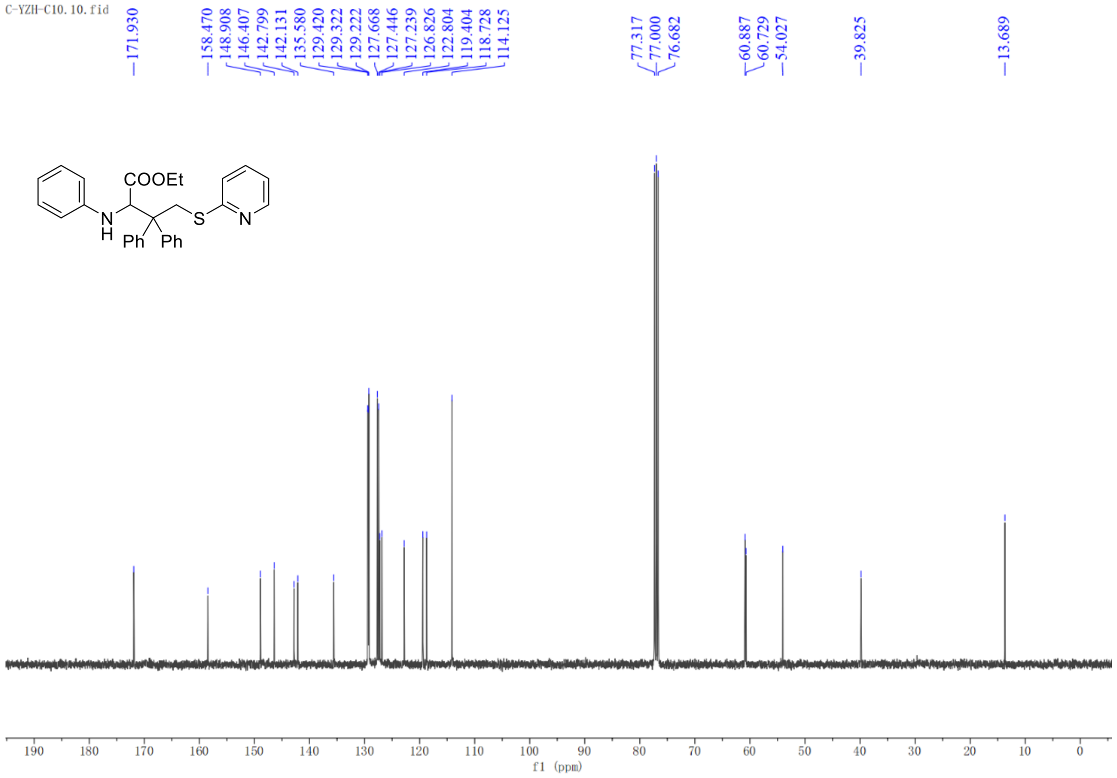

^{1}**H NMR** (400 MHz, $CDCl_3$) spectrum of **19**

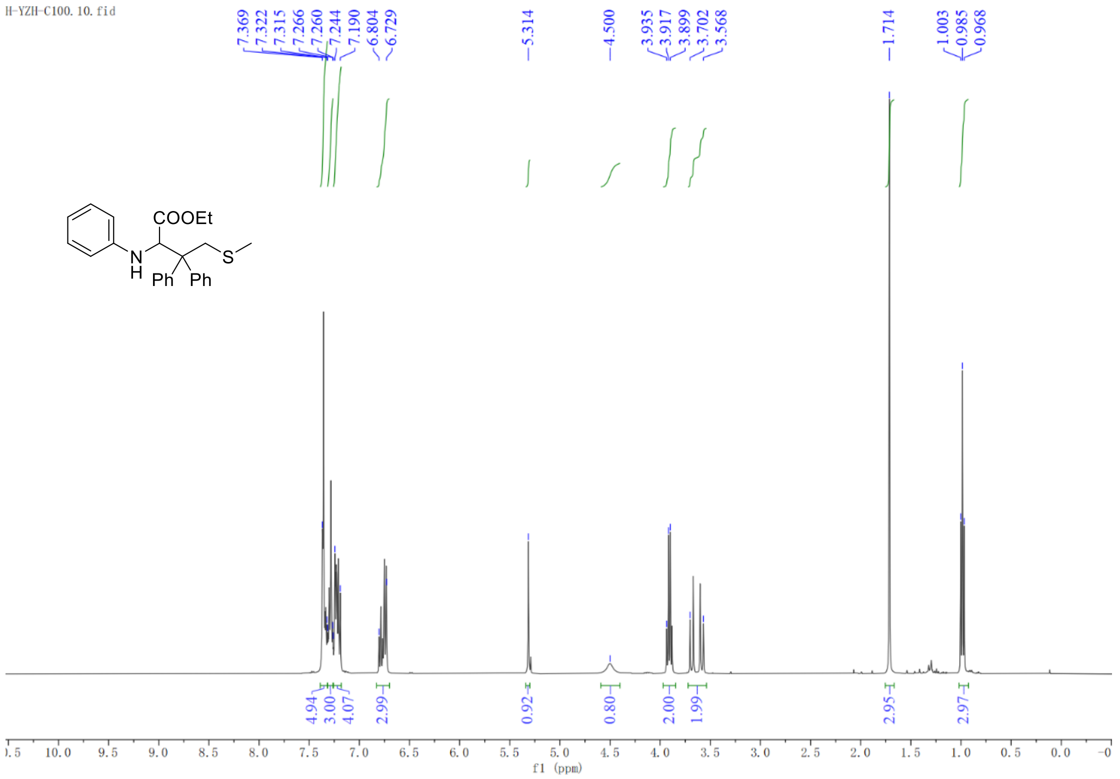

^{13}**C NMR** (101 MHz, $CDCl_3$) spectrum of **19**

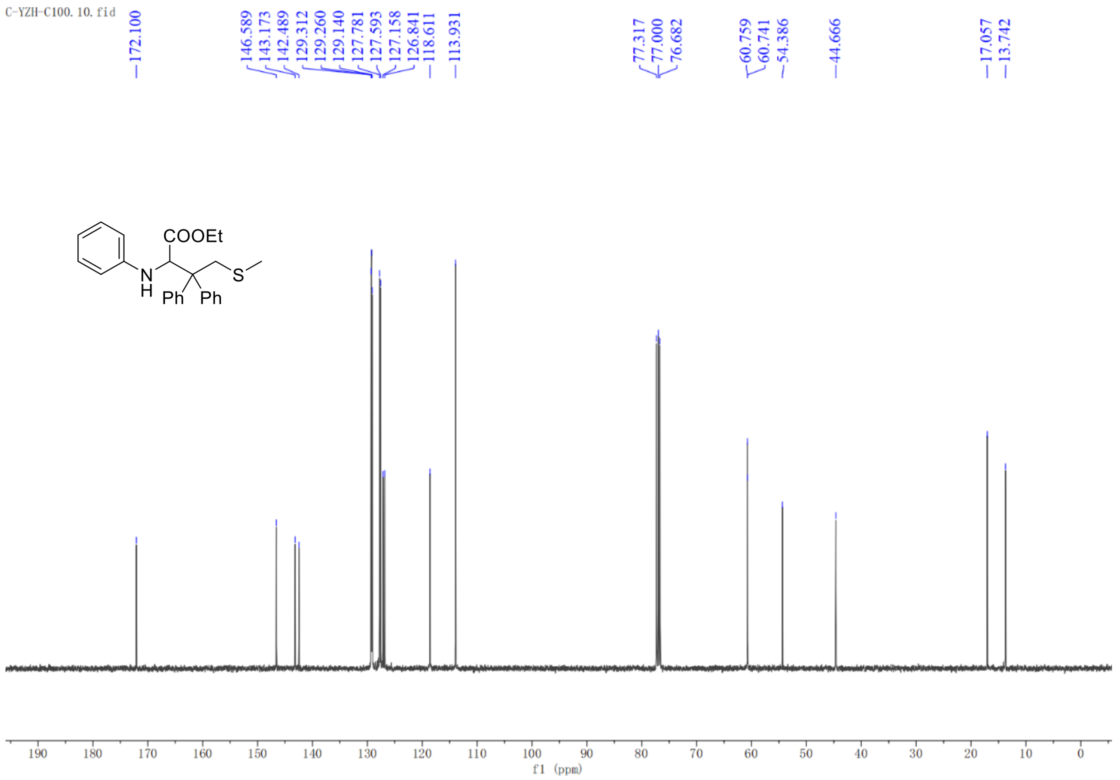

^{1}H NMR (400 MHz, $CDCl_3$) spectrum of **20**

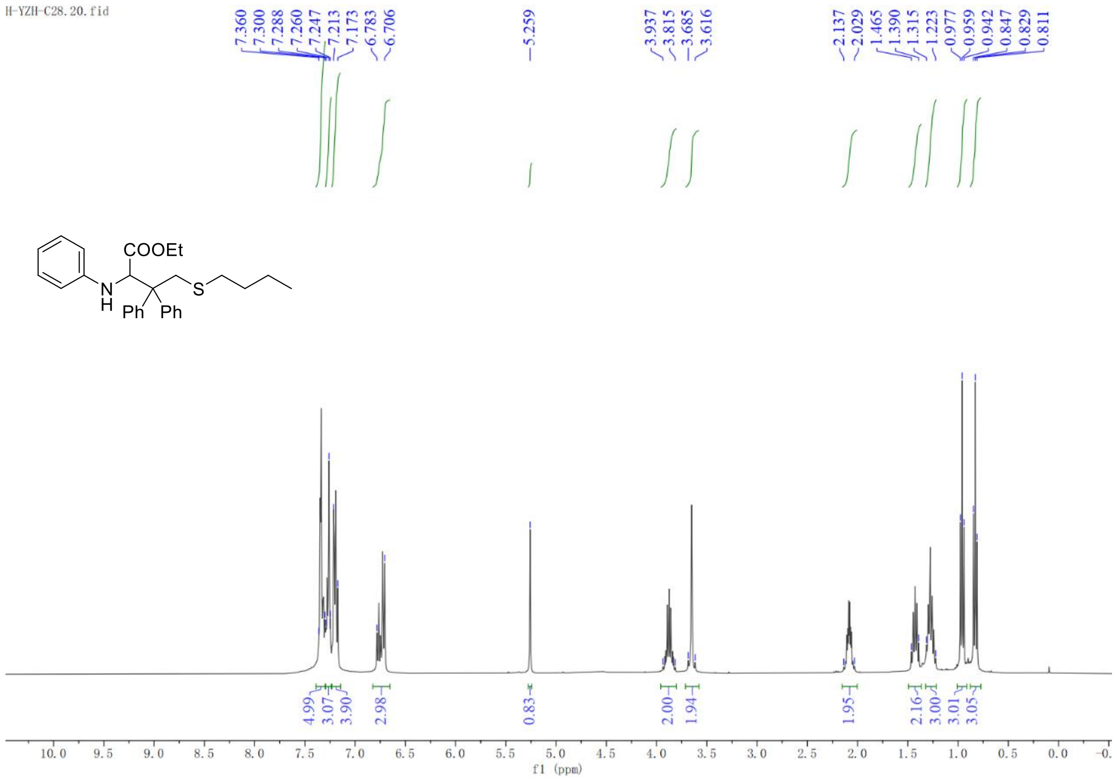

^{13}C NMR (101 MHz, $CDCl_3$) spectrum of **20**

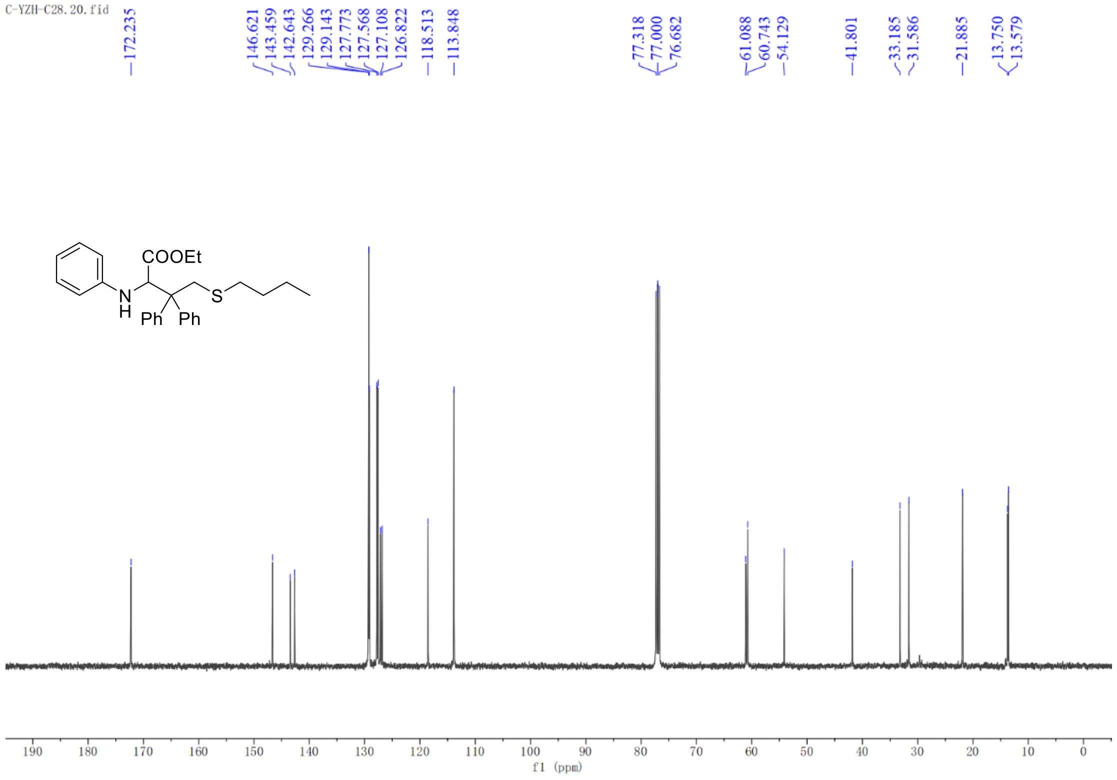

^{1}H NMR (400 MHz, $CDCl_3$) spectrum of **21**

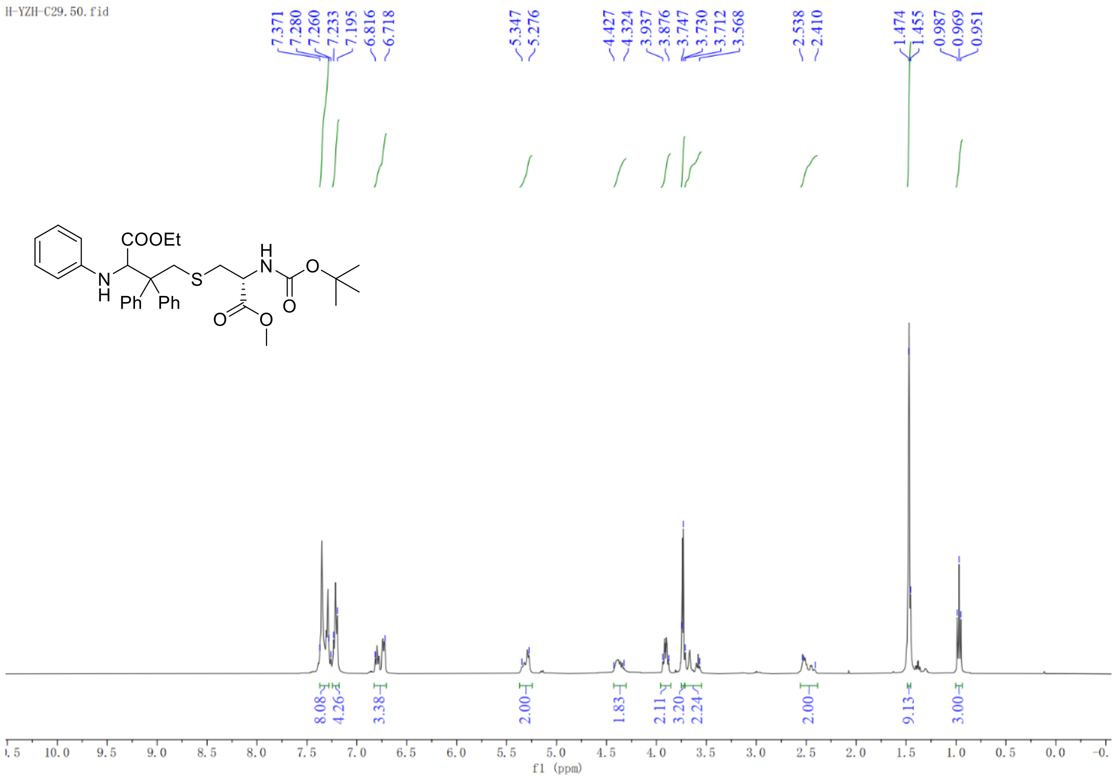

^{13}C NMR (101 MHz, $CDCl_3$) spectrum of **21**

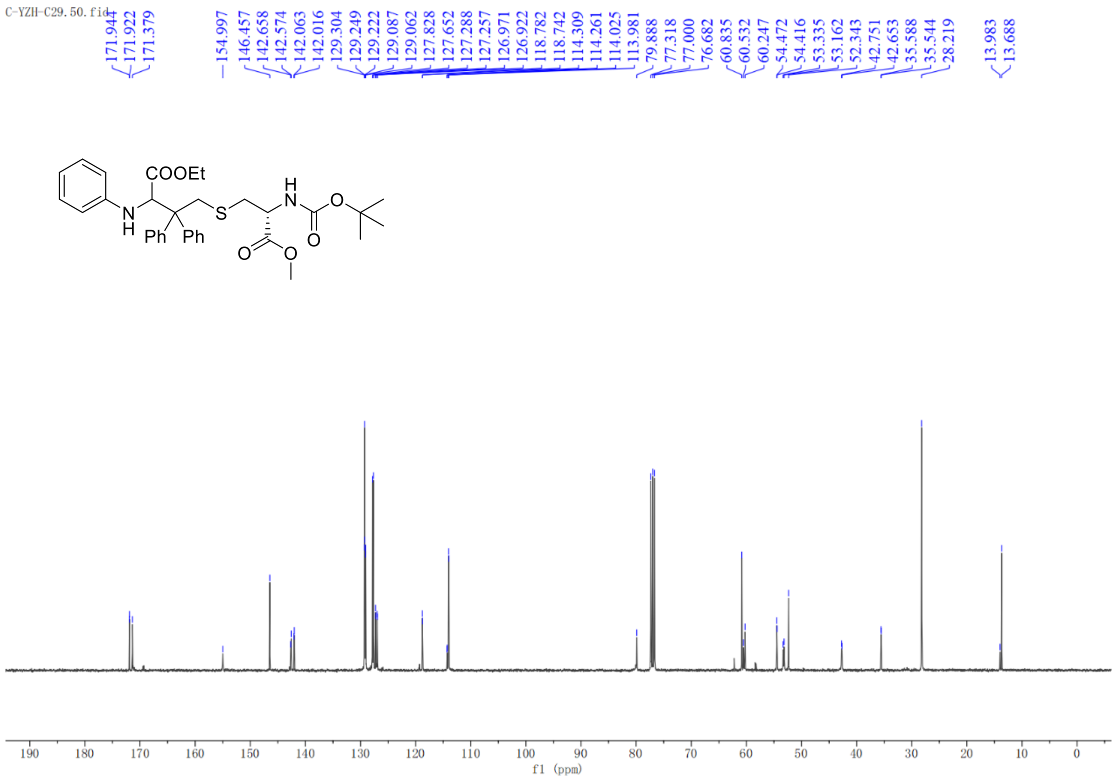

^{1}H NMR (400 MHz, $CDCl_3$) spectrum of **22**

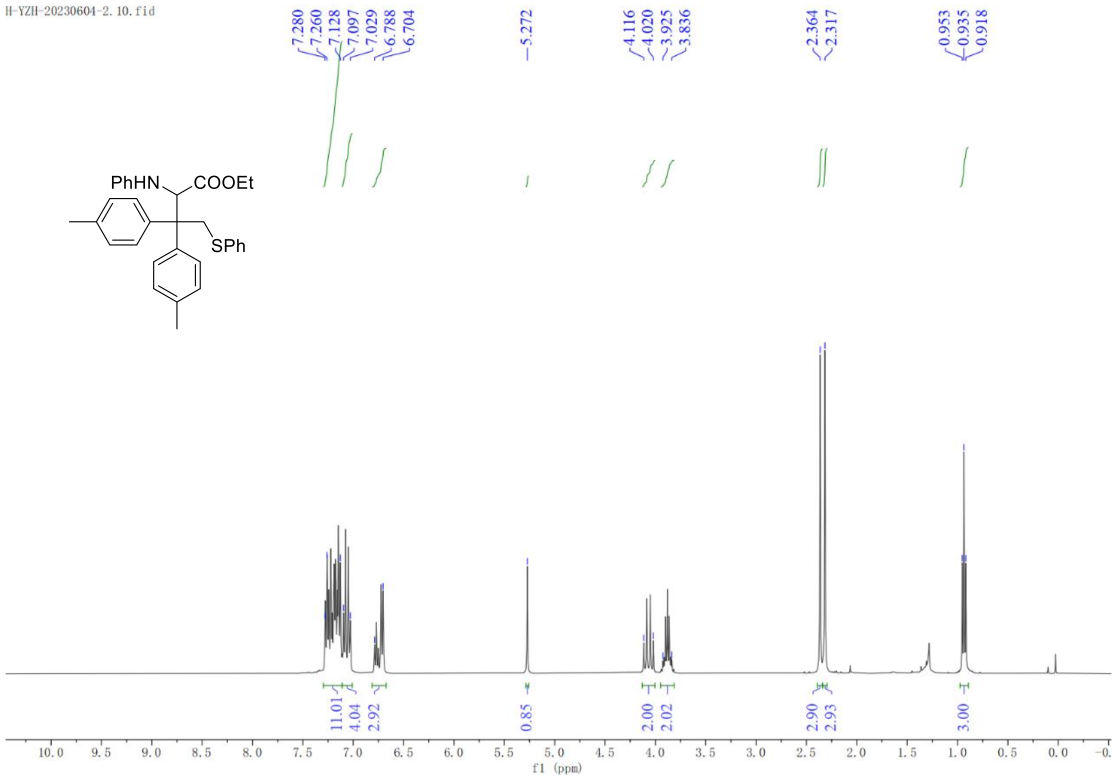

^{13}C NMR (101 MHz, $CDCl_3$) spectrum of **22**

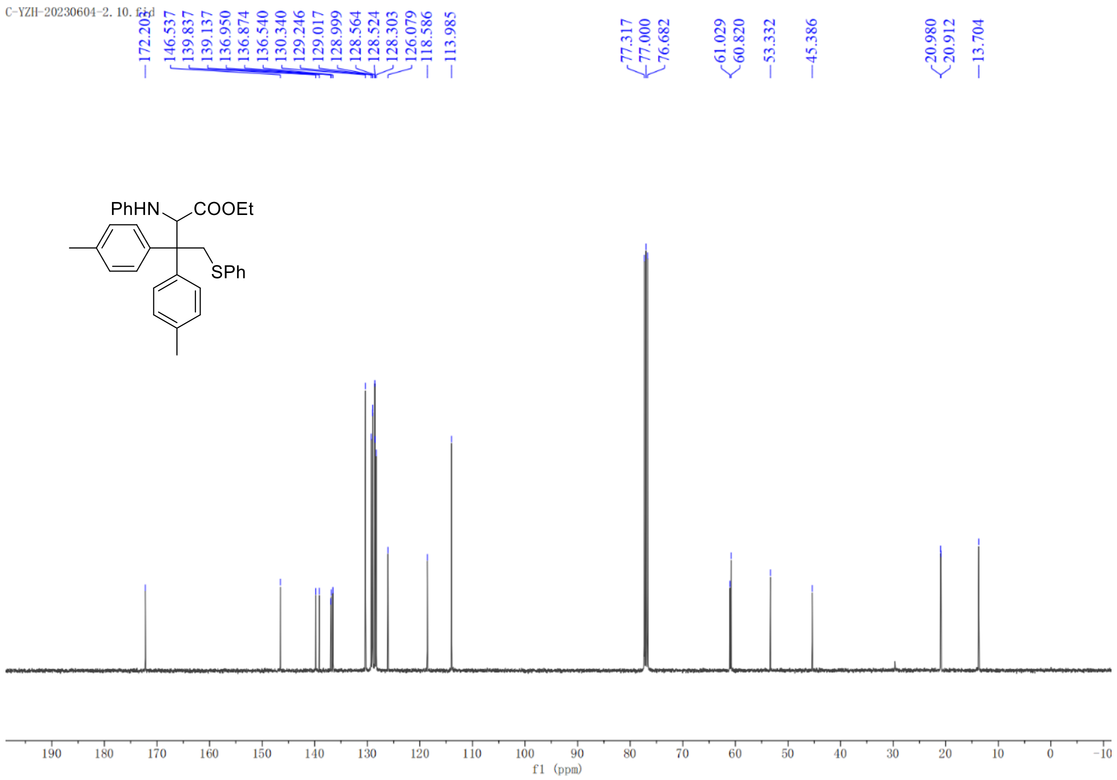

^{1}H NMR (400 MHz, $CDCl_3$) spectrum of **23**

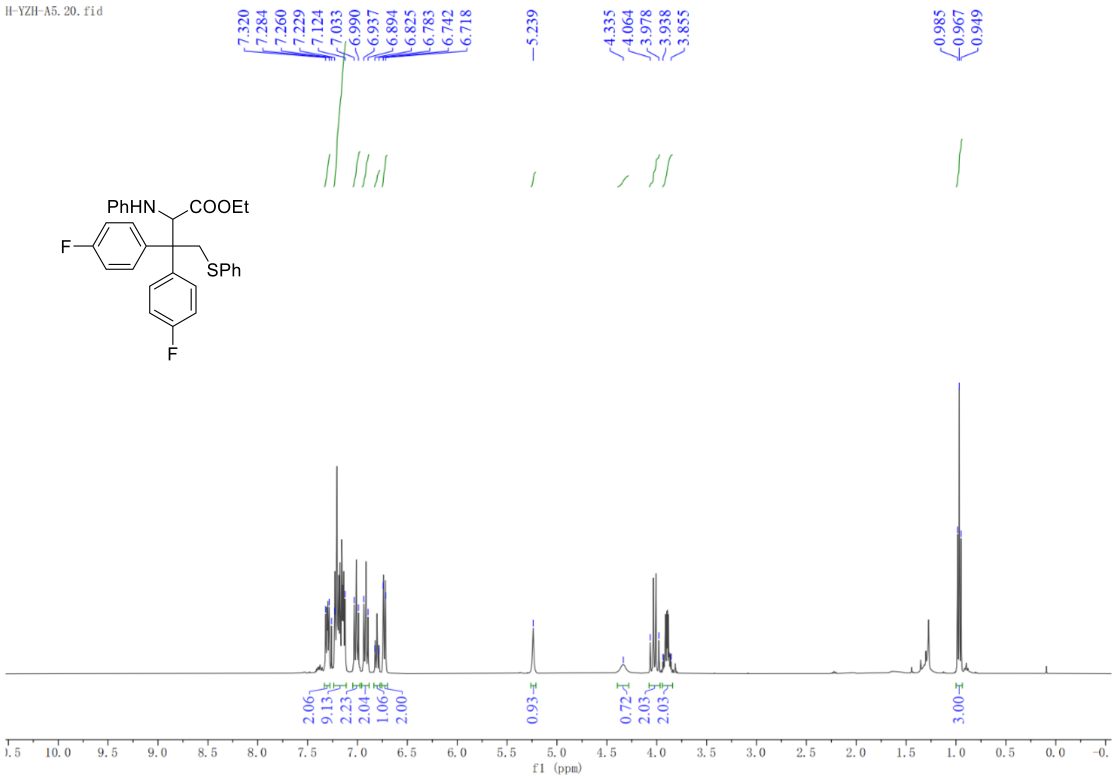

^{13}C NMR (101 MHz, $CDCl_3$) spectrum of **23**

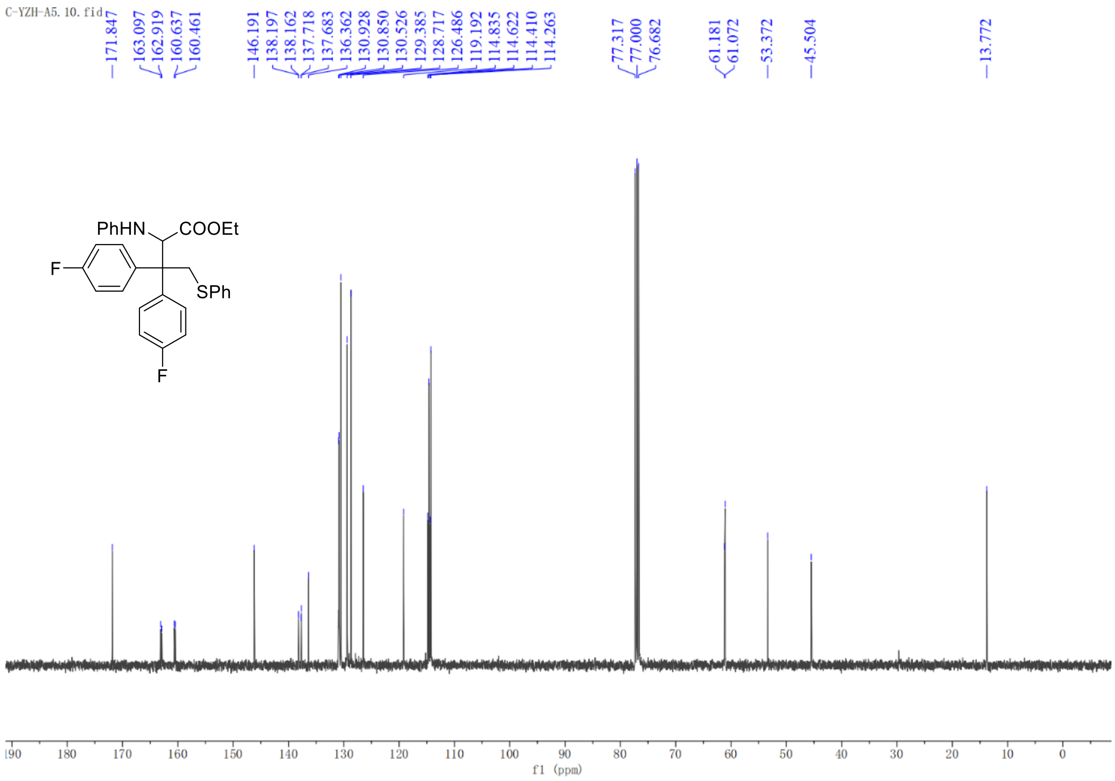

^{19}**F NMR** (376 MHz, $CDCl_3$) spectrum of **23**

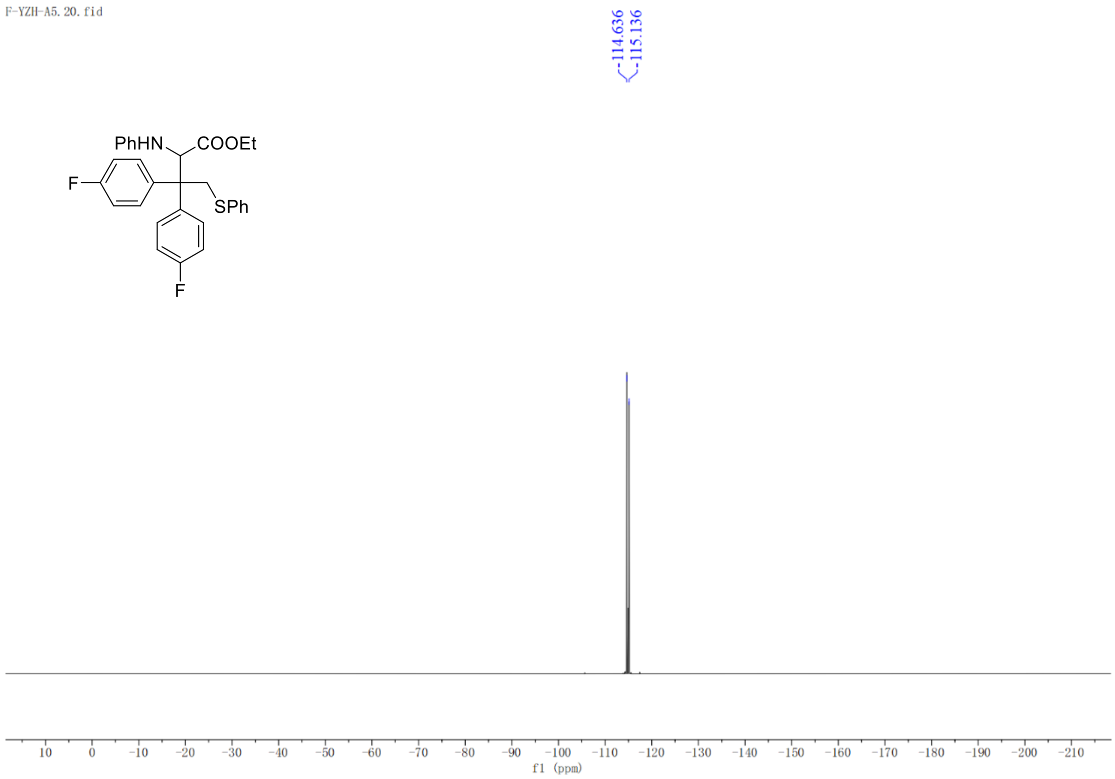

^{1}**H NMR** (400 MHz, $CDCl_3$) spectrum of **24**

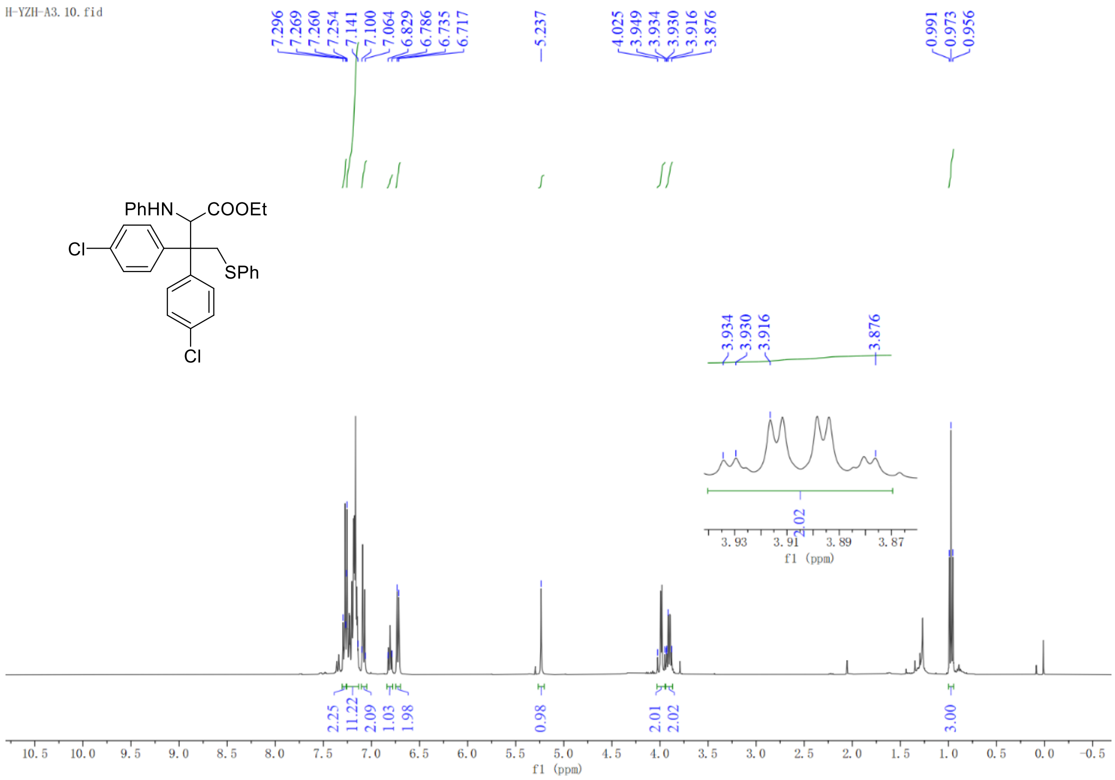

^{13}**C NMR** (101 MHz, $CDCl_3$) spectrum of **24**

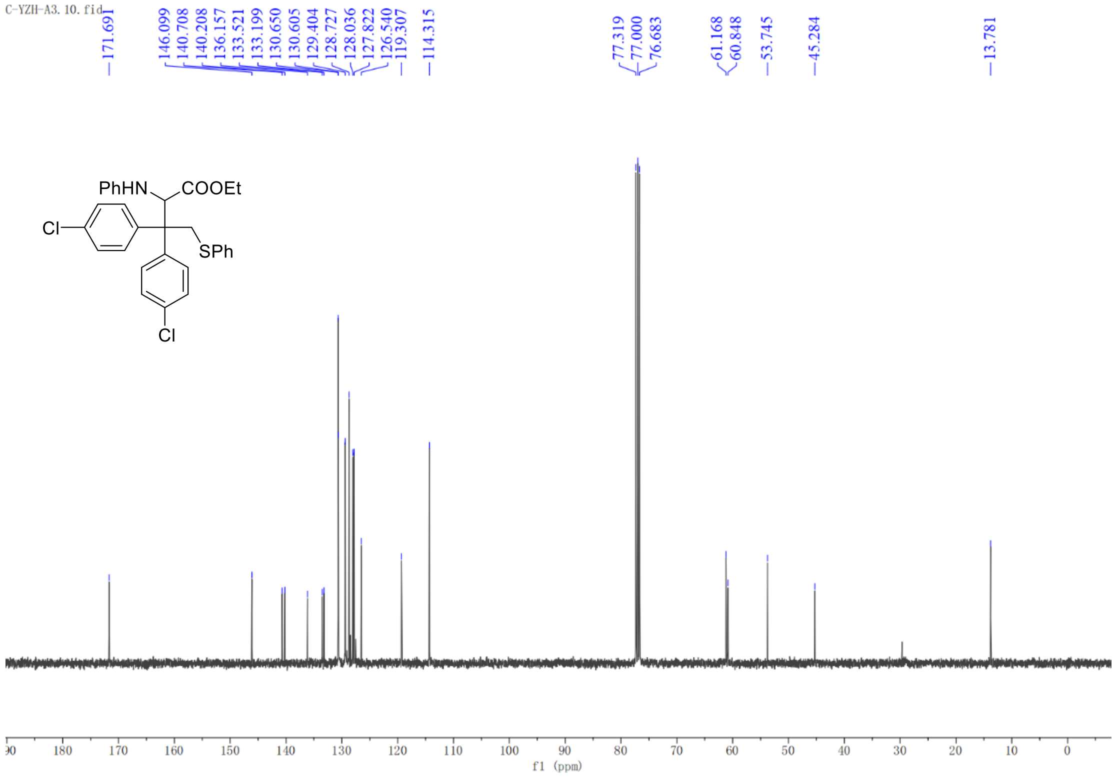

^{1}**H NMR** (400 MHz, $CDCl_3$) spectrum of **25**

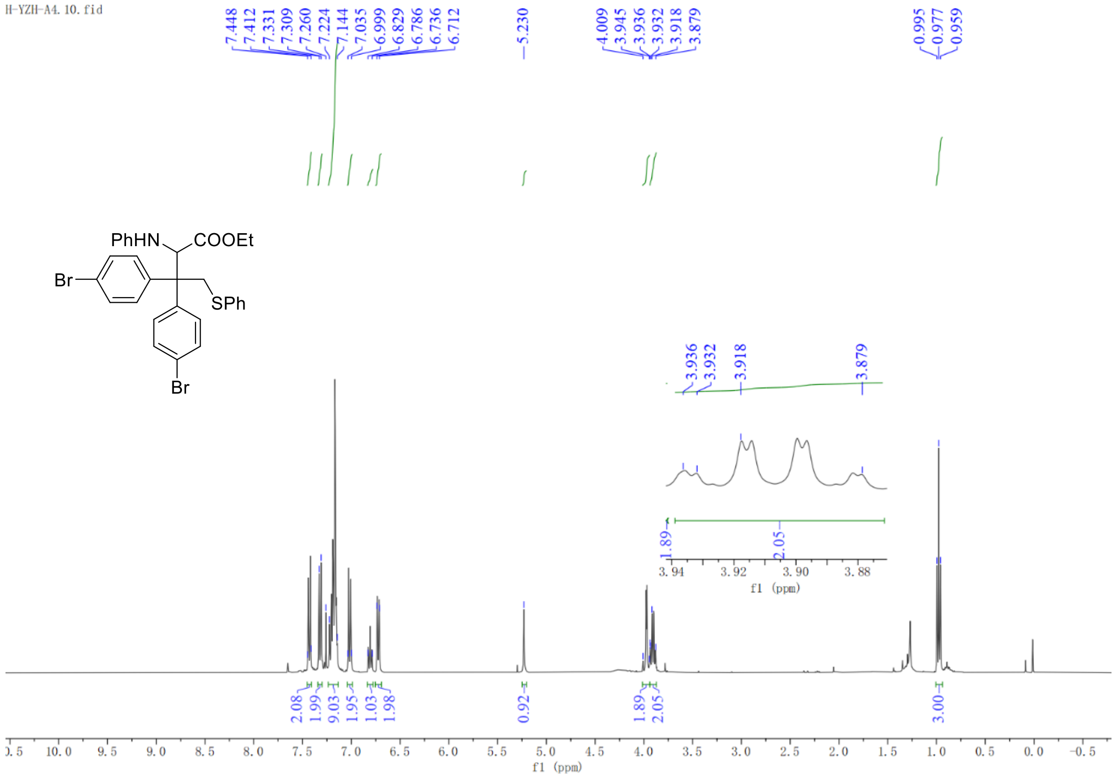

^{13}C NMR (101 MHz, $CDCl_3$) spectrum of **25**

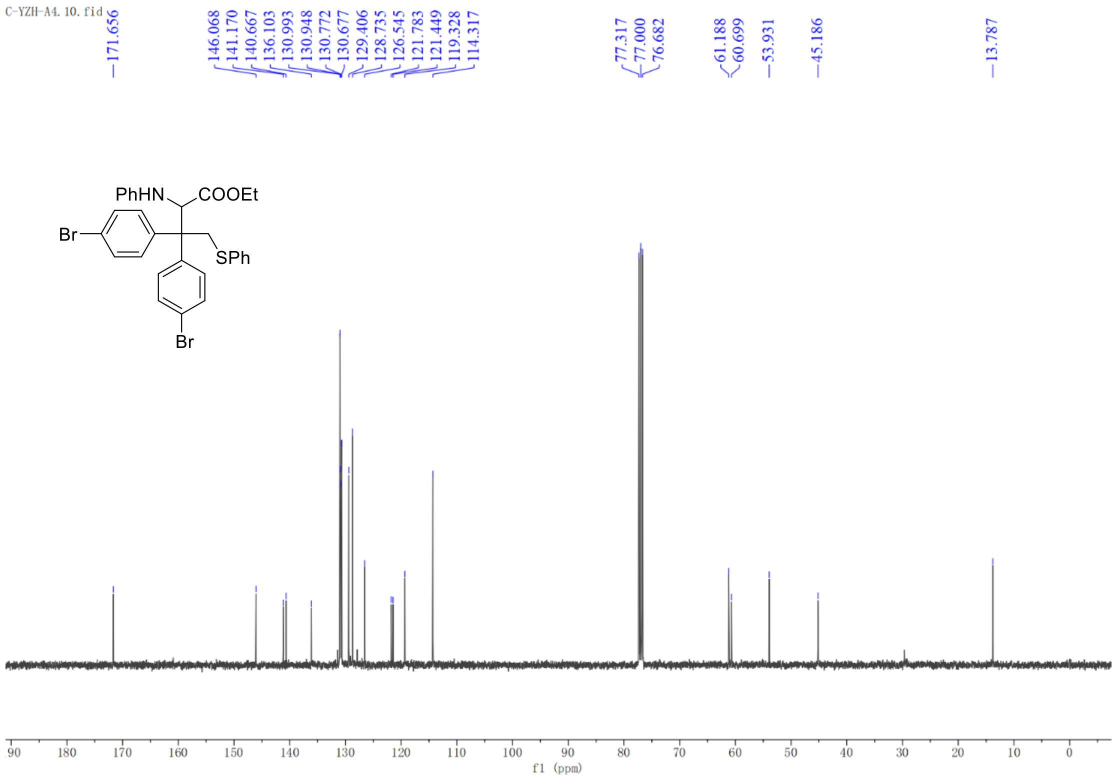

^{1}H NMR (400 MHz, $CDCl_3$) spectrum of **26**

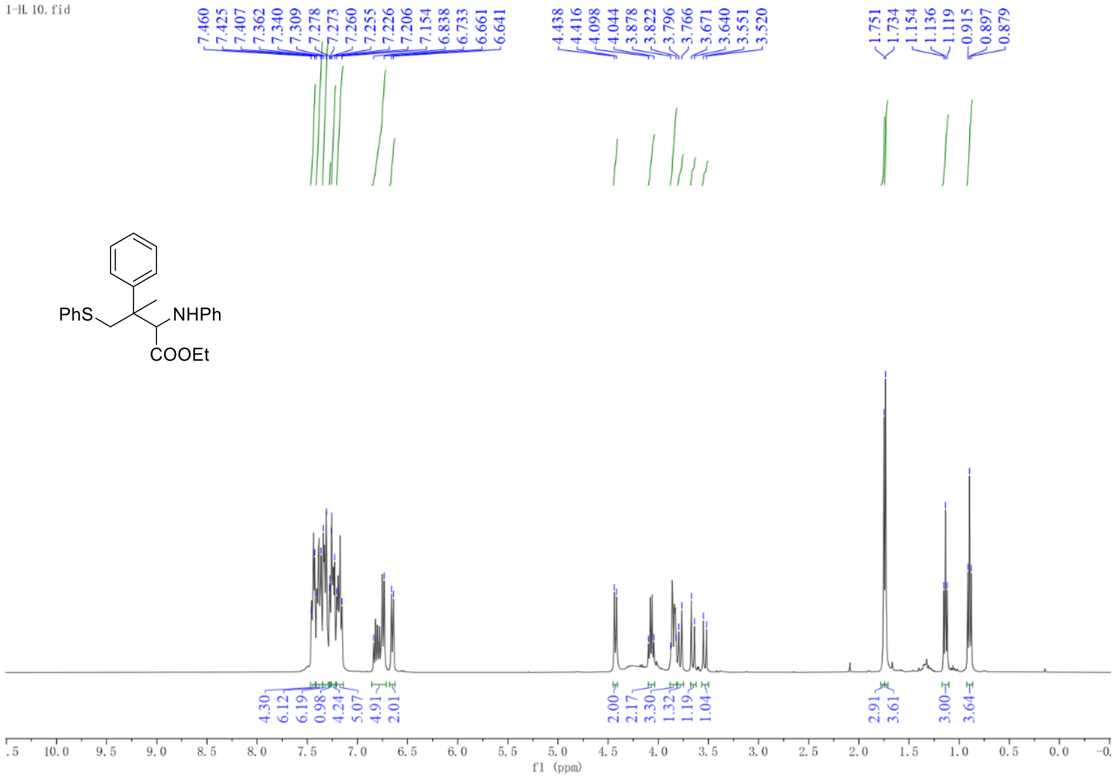

^{13}C NMR (101 MHz, $CDCl_3$) spectrum of **26**

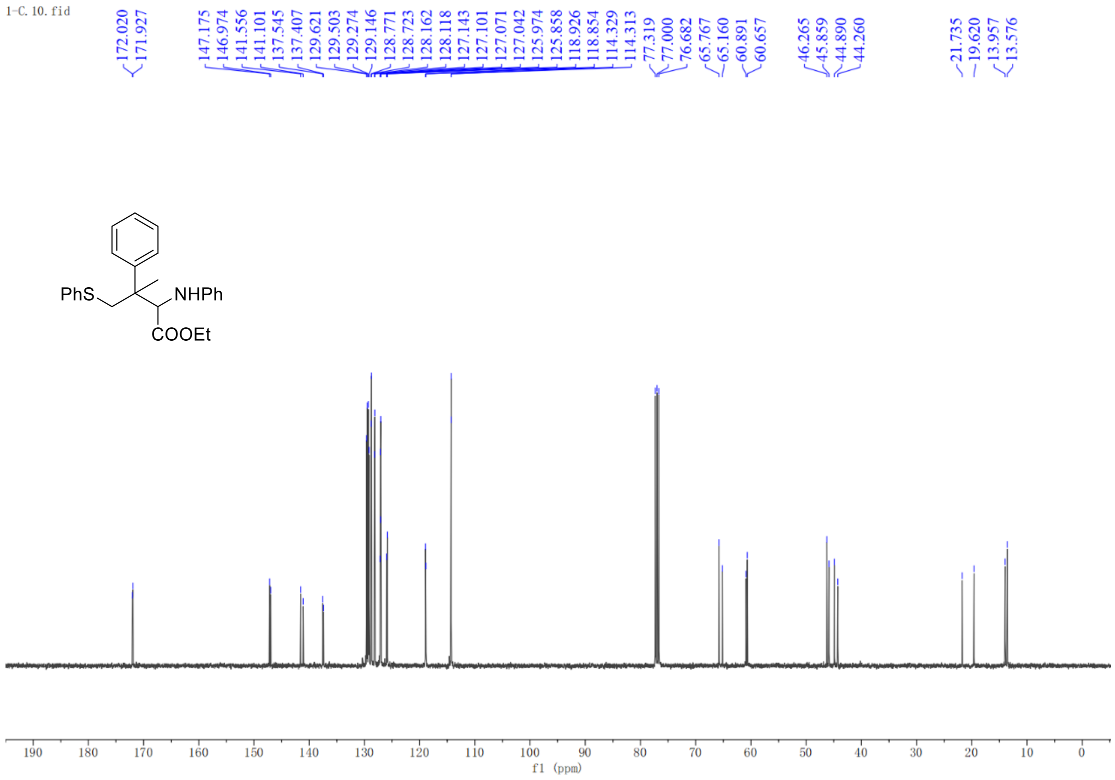

^{1}H NMR (400 MHz, $CDCl_3$) spectrum of **27**

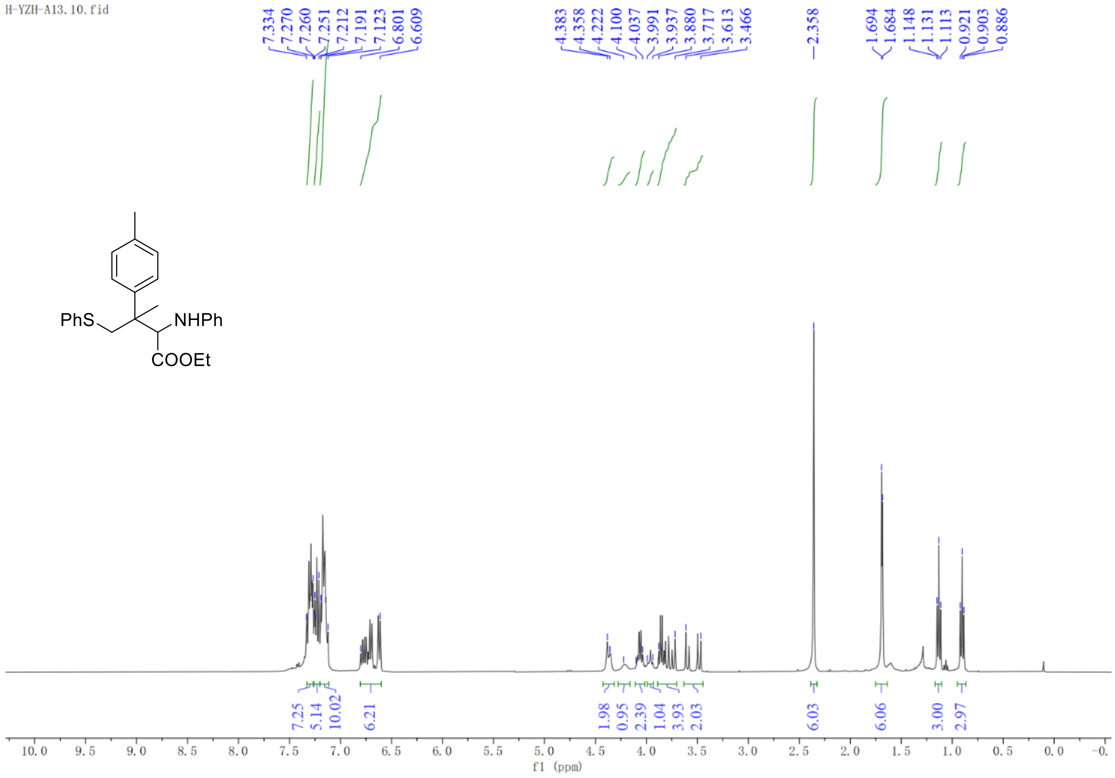

^{13}C NMR (101 MHz, $CDCl_3$) spectrum of **27**

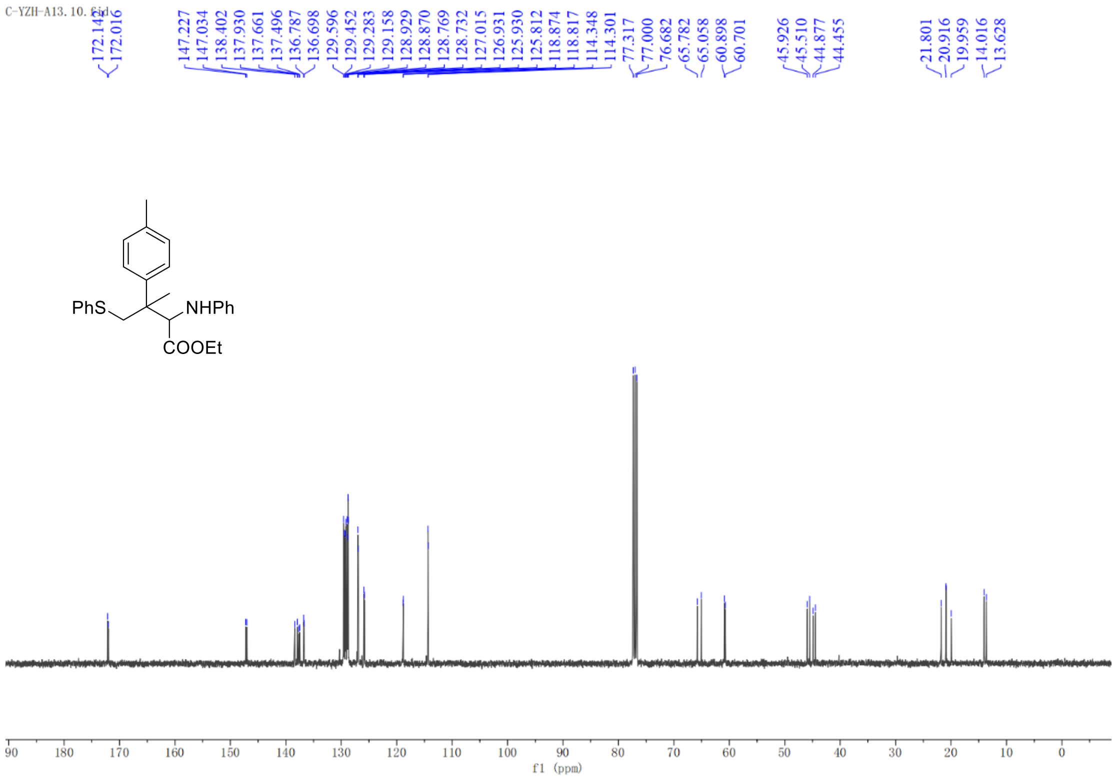

^{1}H NMR (400 MHz, $CDCl_3$) spectrum of **28**

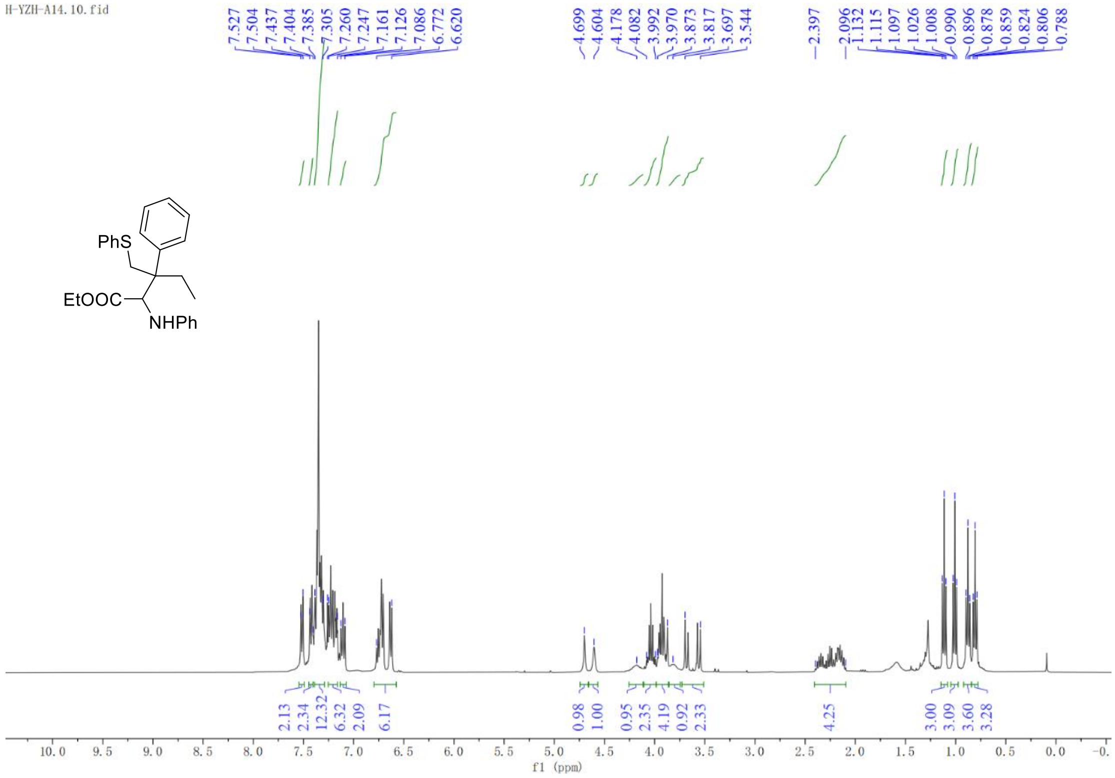

^{13}C NMR (101 MHz, $CDCl_3$) spectrum of **28**

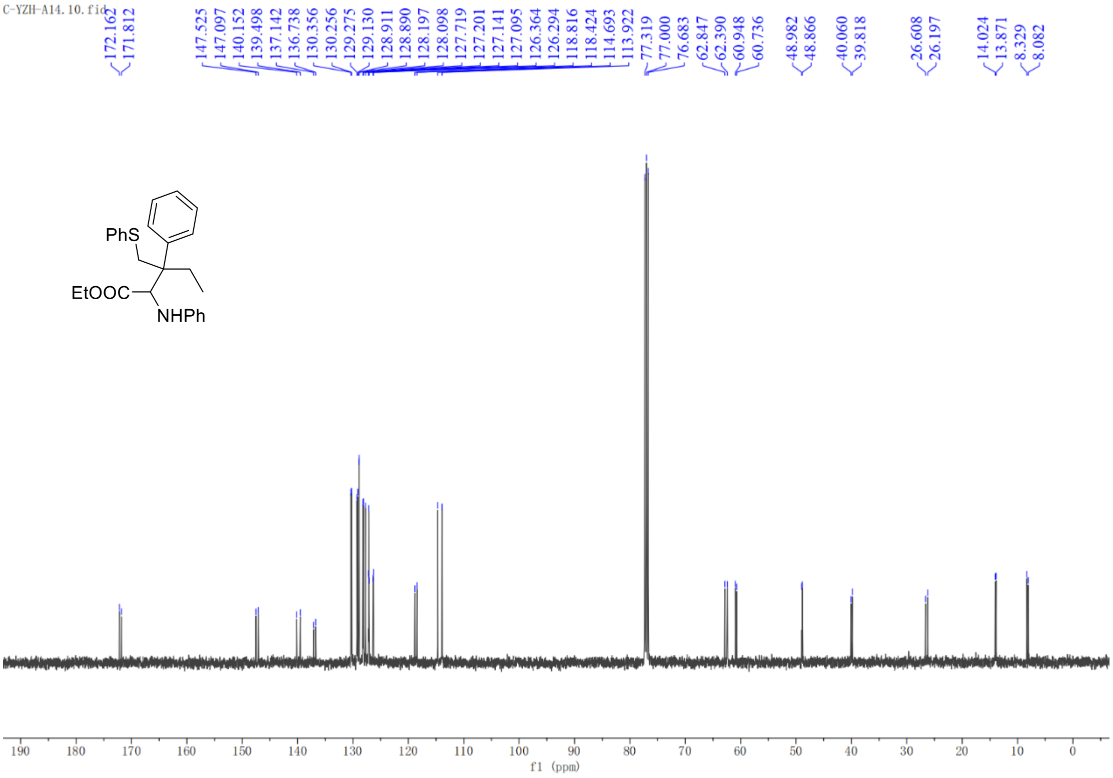

^{1}H NMR (400 MHz, $CDCl_3$) spectrum of **29**

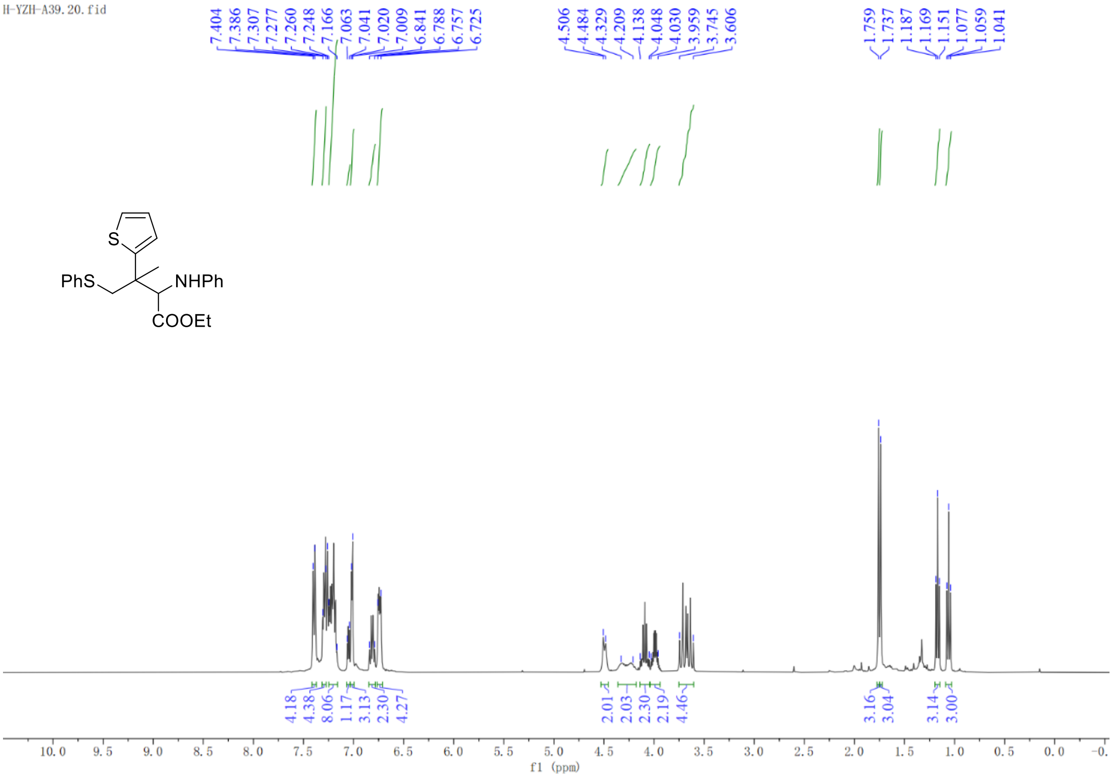

^{13}C NMR (101 MHz, $CDCl_3$) spectrum of **29**

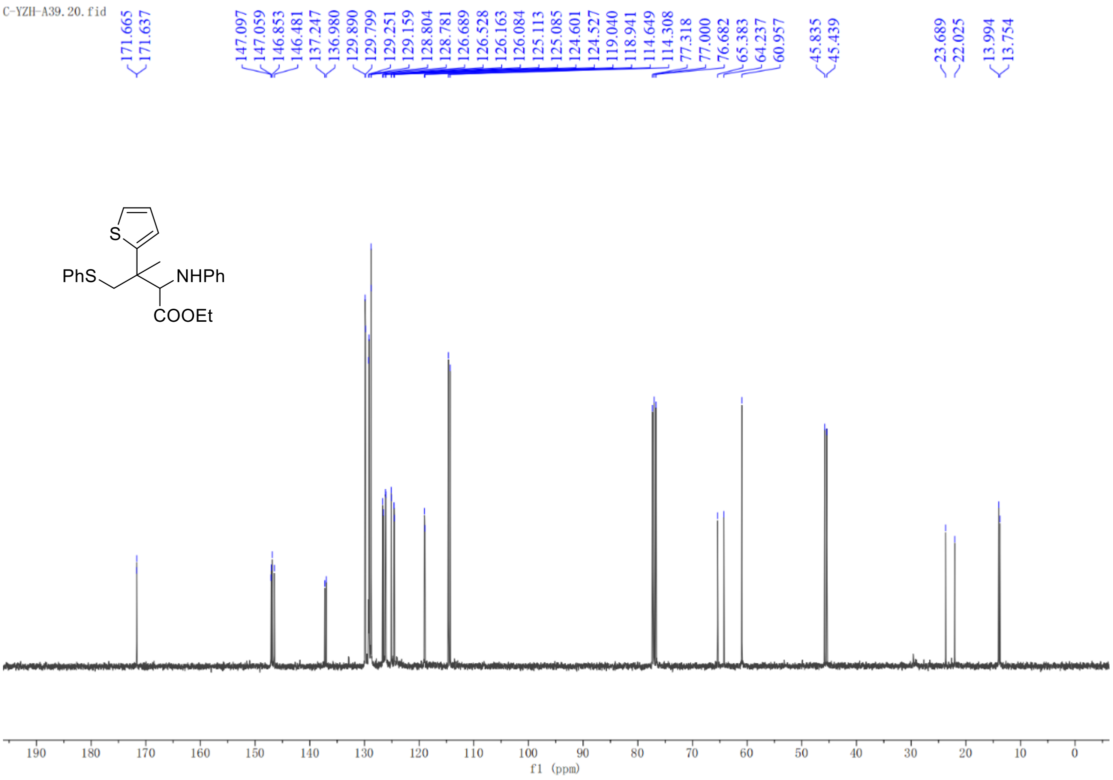

^{1}H NMR (400 MHz, $CDCl_3$) spectrum of **30** isomer-1

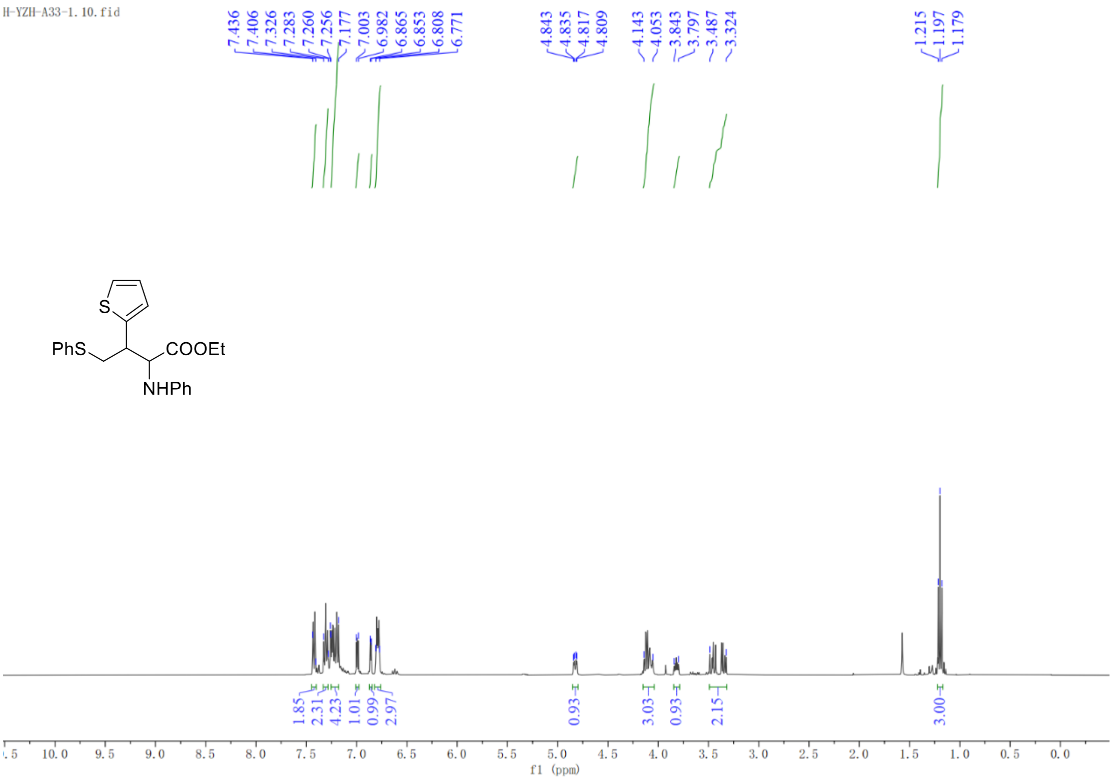

^{13}C **NMR** (101 MHz, $CDCl_3$) spectrum of **30** isomer-1

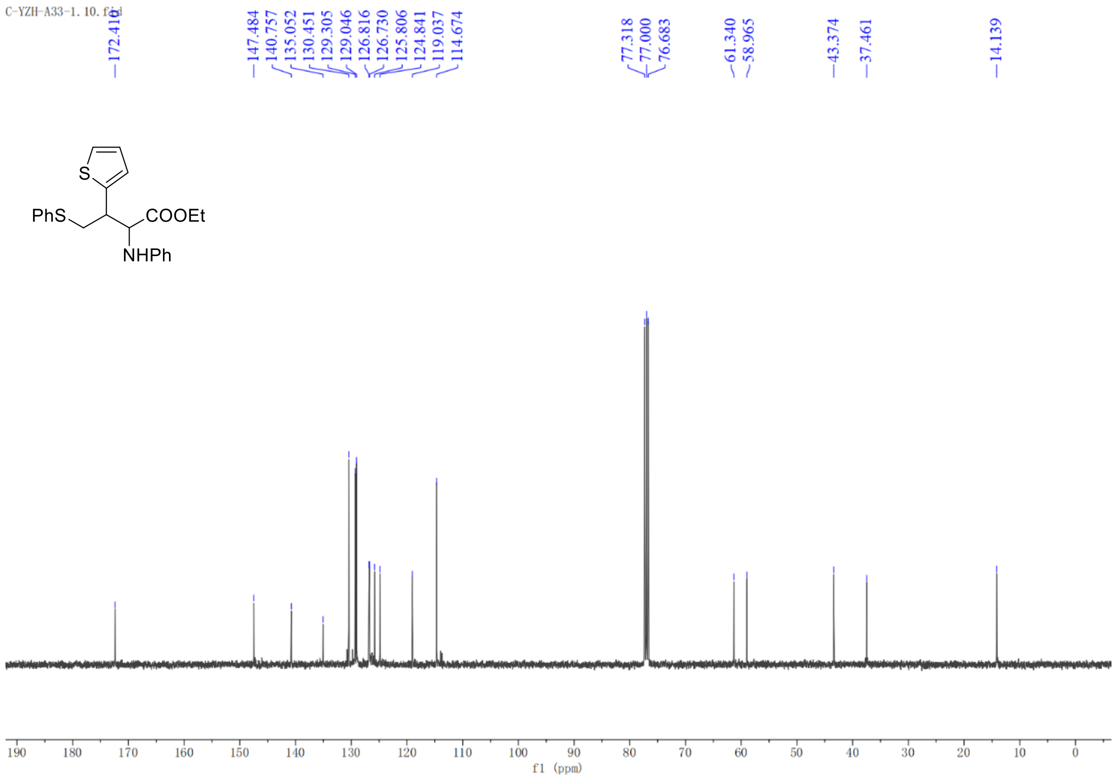

^{1}H **NMR** (400 MHz, $CDCl_3$) spectrum of **30** isomer-2

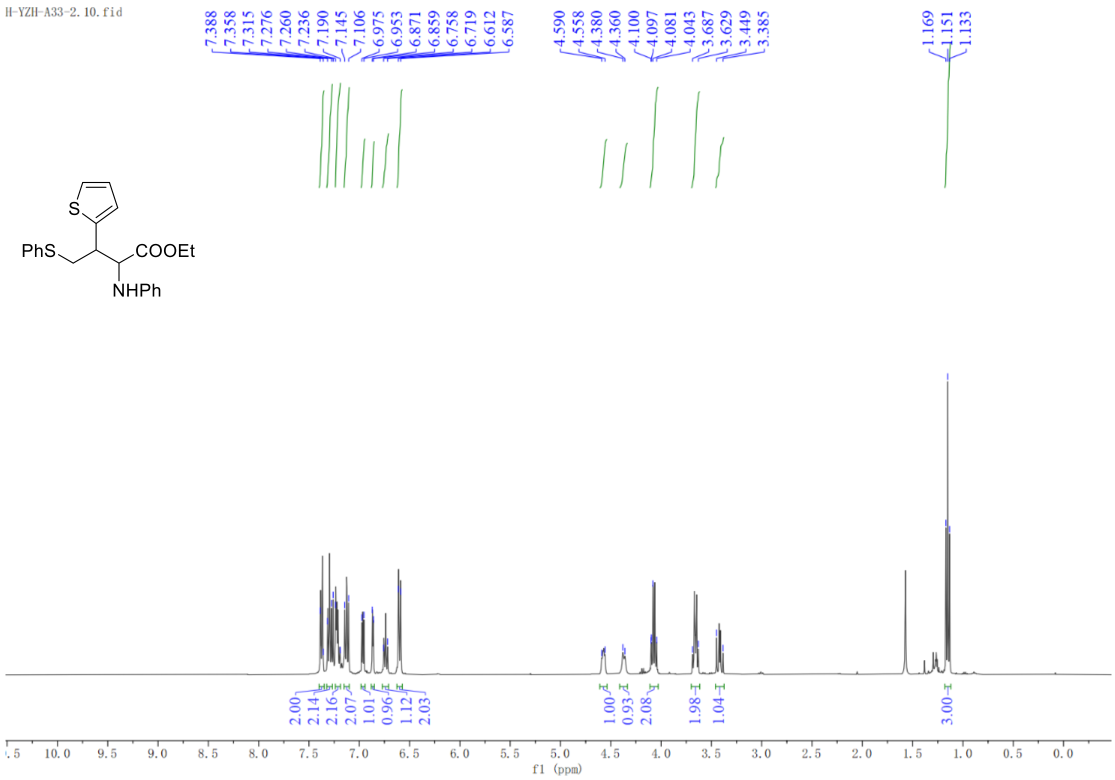

^{13}C NMR (101 MHz, CDCl3) spectrum of **30** isomer-2

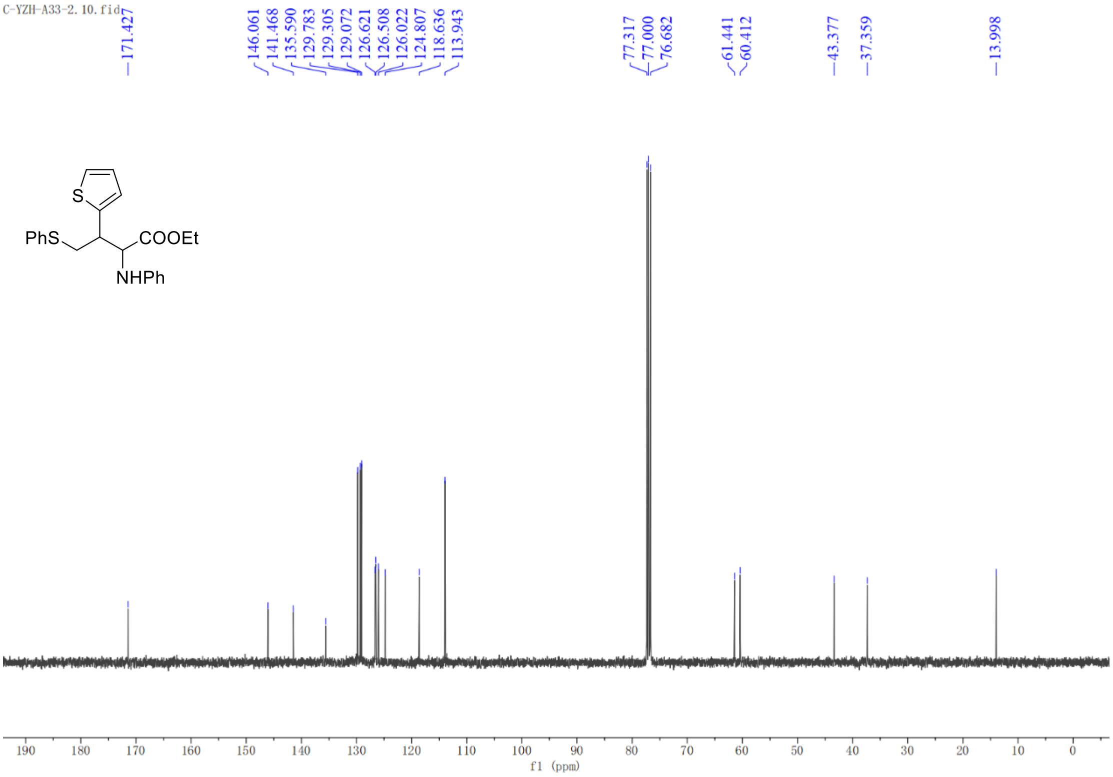

^{1}H NMR (400 MHz, CDCl3) spectrum of **31** major isomer

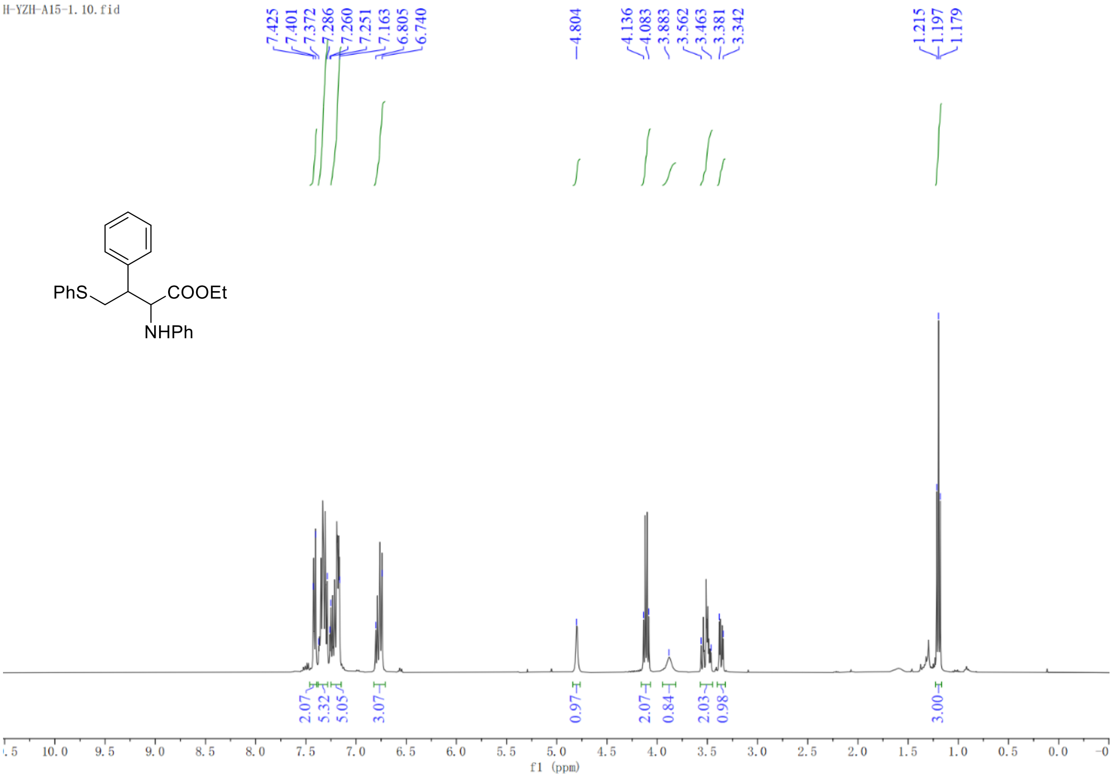

^{13}**C NMR** (101 MHz, $CDCl_3$) spectrum of **31** major isomer

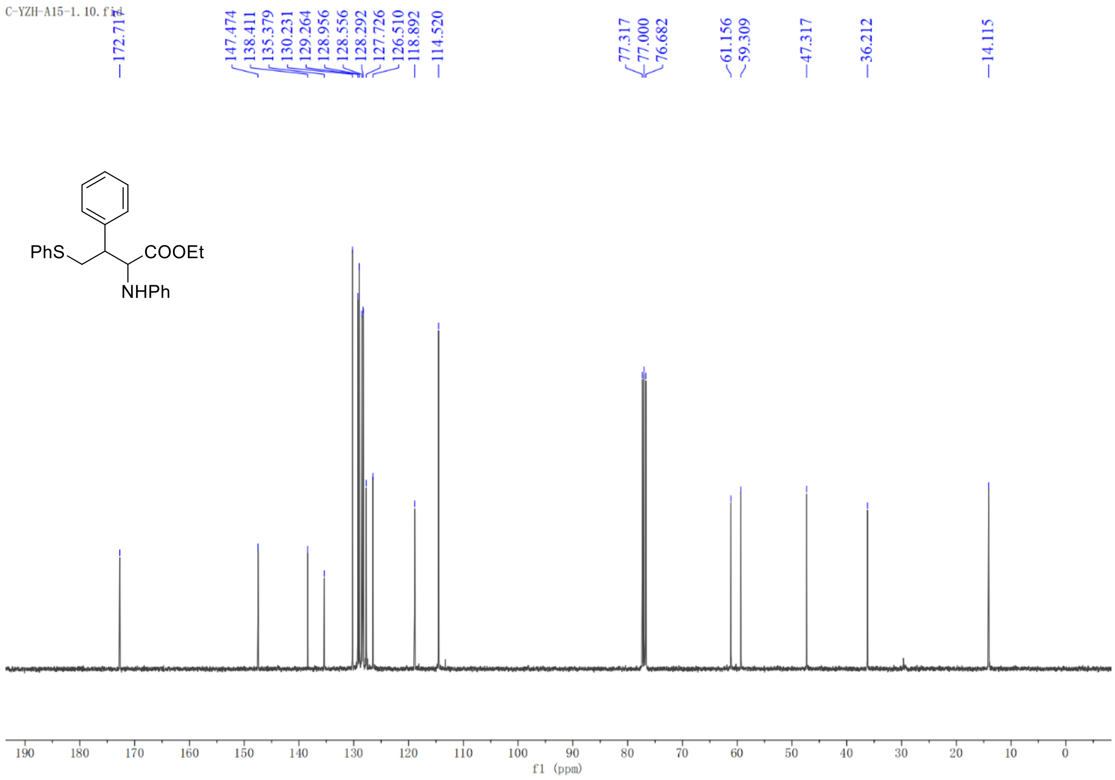

^{1}**H NMR** (400 MHz, $CDCl_3$) spectrum of **31** minor isomer

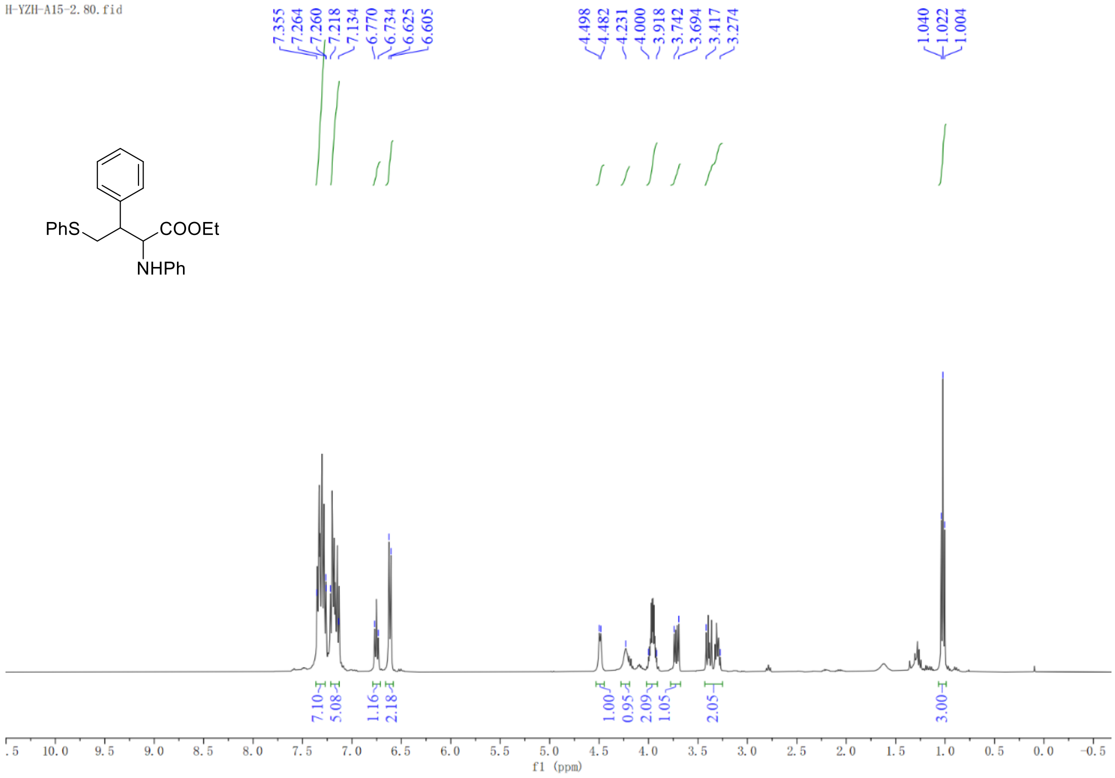

^{13}**C NMR** (101 MHz, $CDCl_3$) spectrum of **31** minor isomer

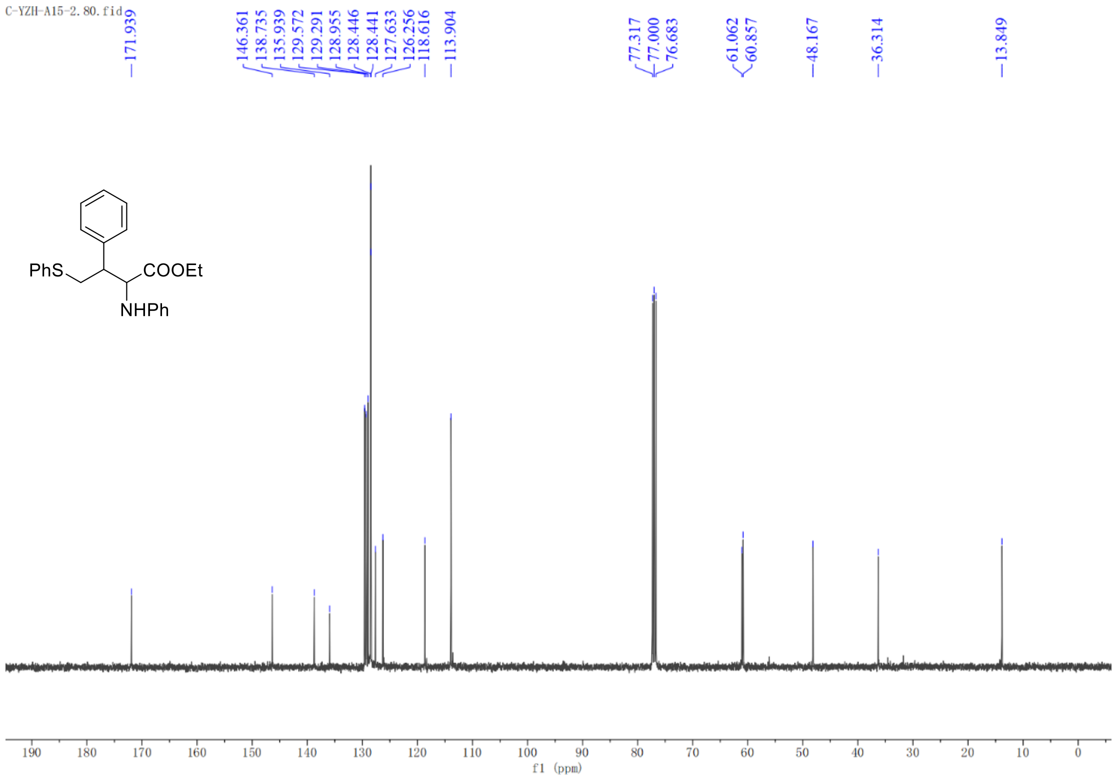

^{1}**H NMR** (400 MHz, $CDCl_3$) spectrum of **32** major isomer

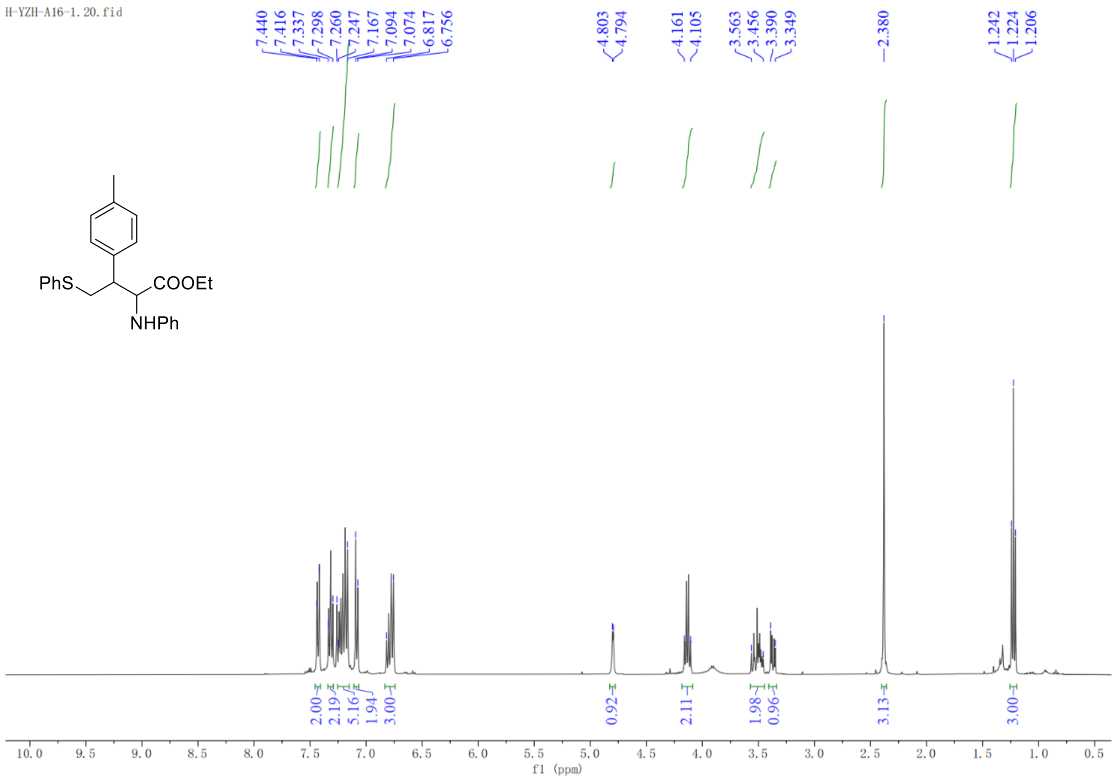

^{13}C NMR (101 MHz, $CDCl_3$) spectrum of **32** major isomer

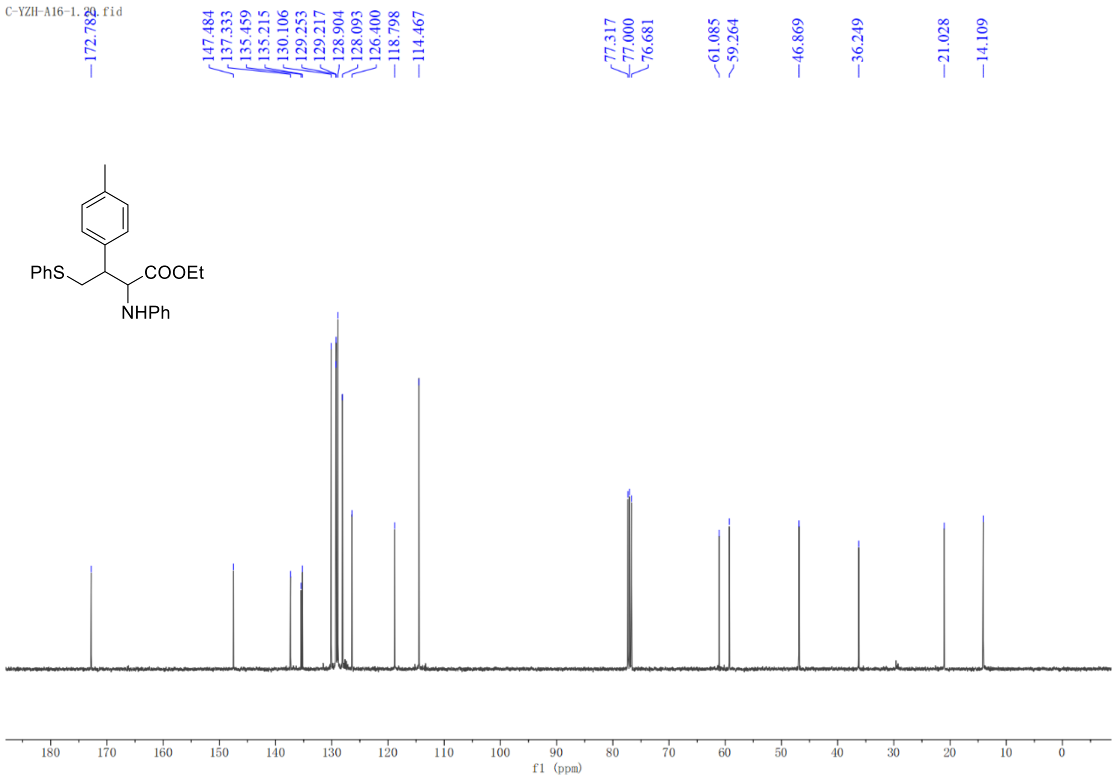

^{1}H NMR (400 MHz, $CDCl_3$) spectrum of **32** minor isomer

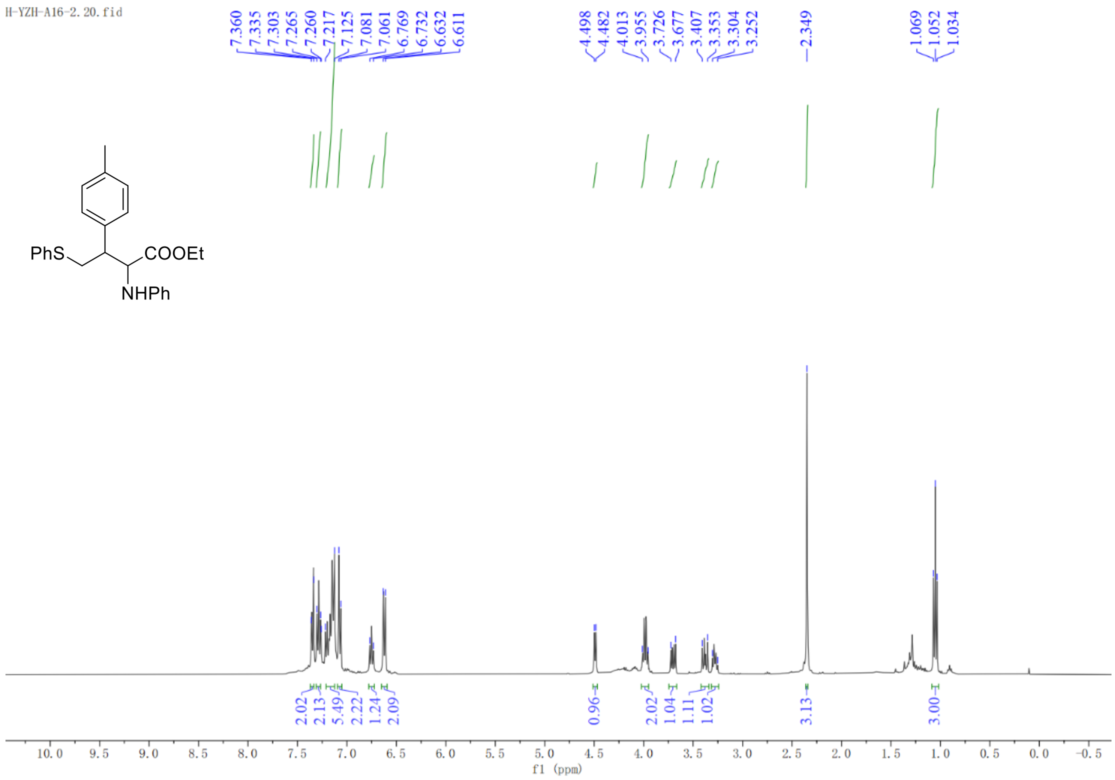

^{13}C NMR (101 MHz, $CDCl_3$) spectrum of **32** minor isomer

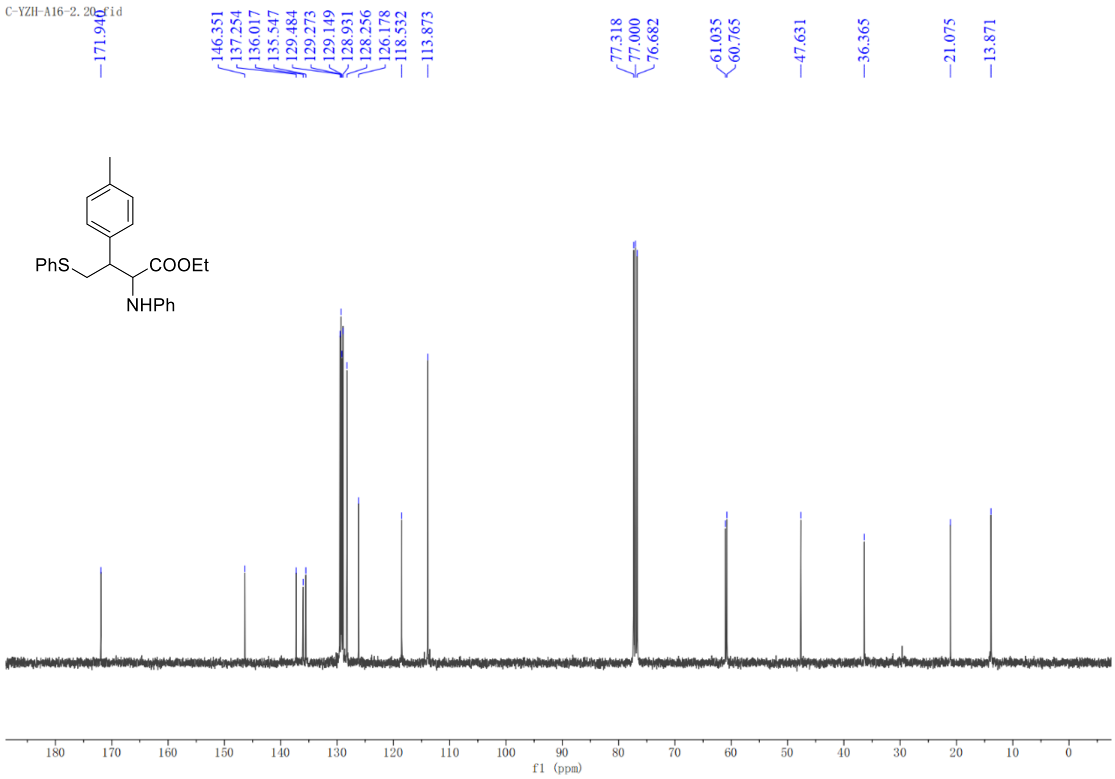

^{1}H NMR (400 MHz, $CDCl_3$) spectrum of **33** major isomer

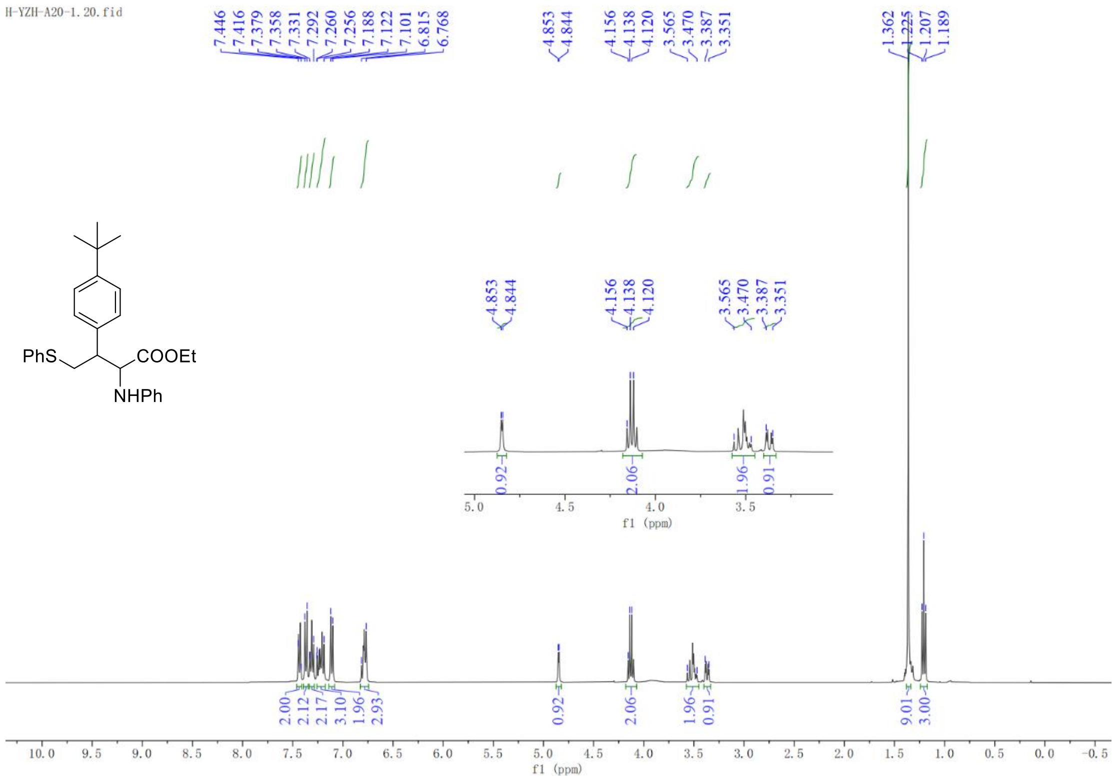

^{13}C NMR (101 MHz, $CDCl_3$) spectrum of **33** major isomer

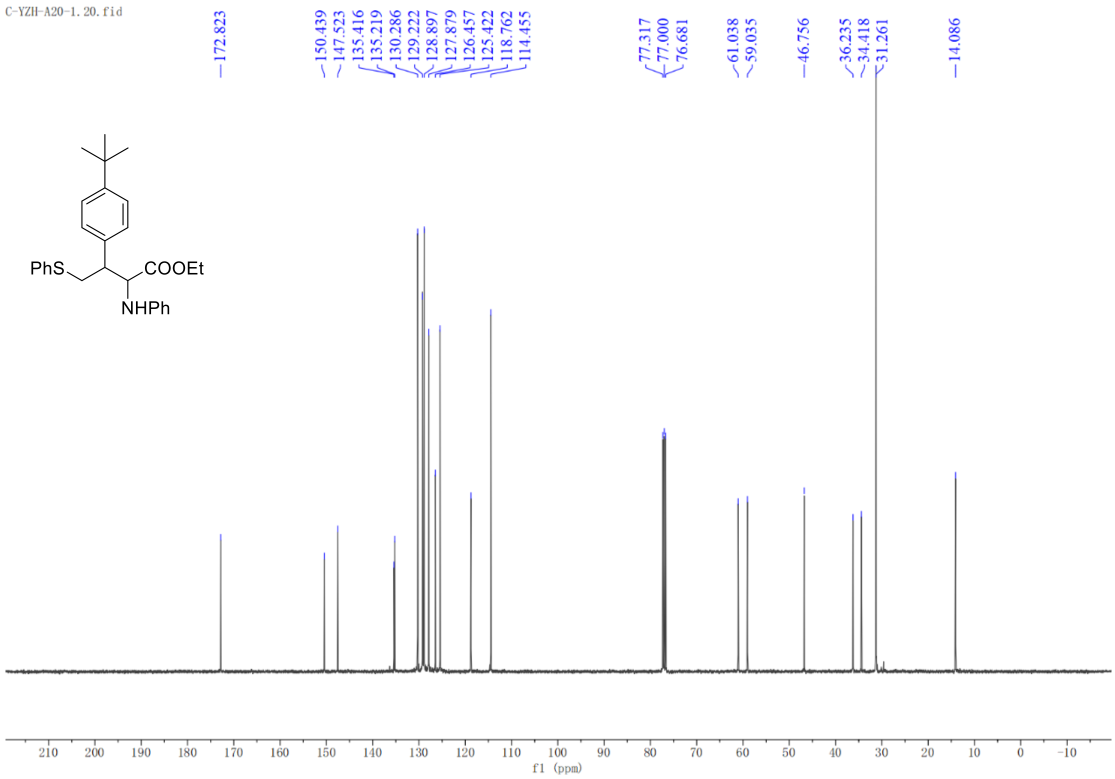

^{1}H NMR (400 MHz, $CDCl_3$) spectrum of **33** minor isomer

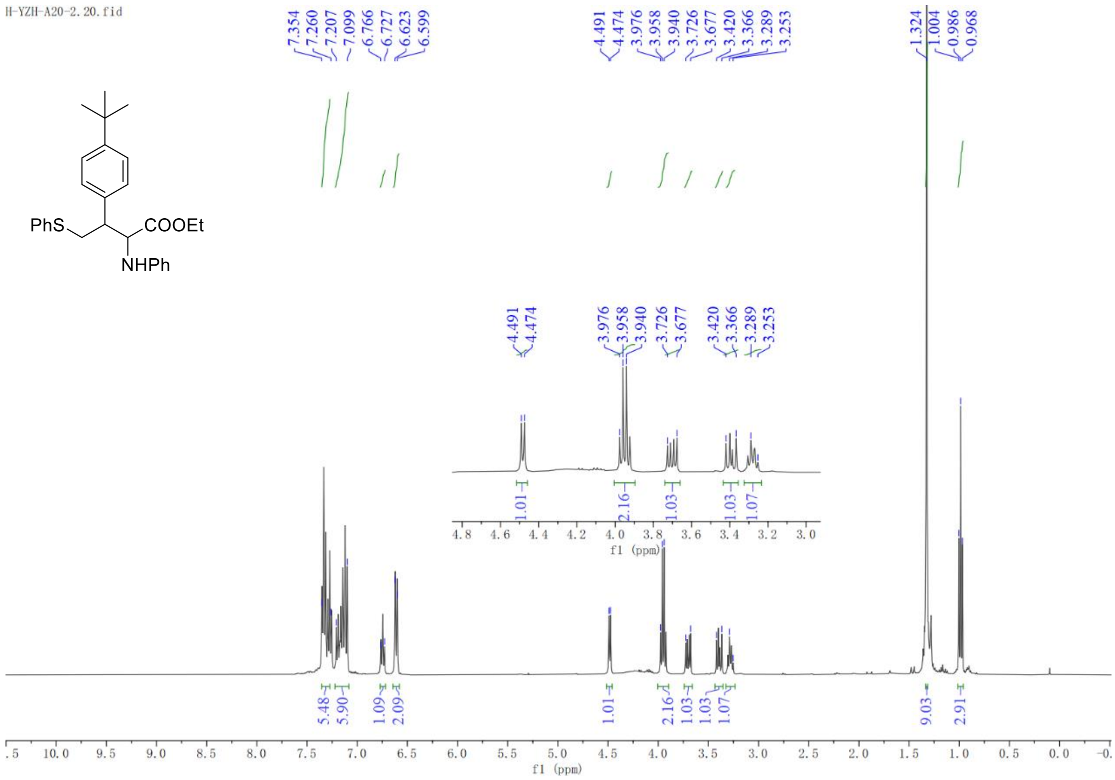

^{13}C **NMR** (101 MHz, $CDCl_3$) spectrum of **33** minor isomer

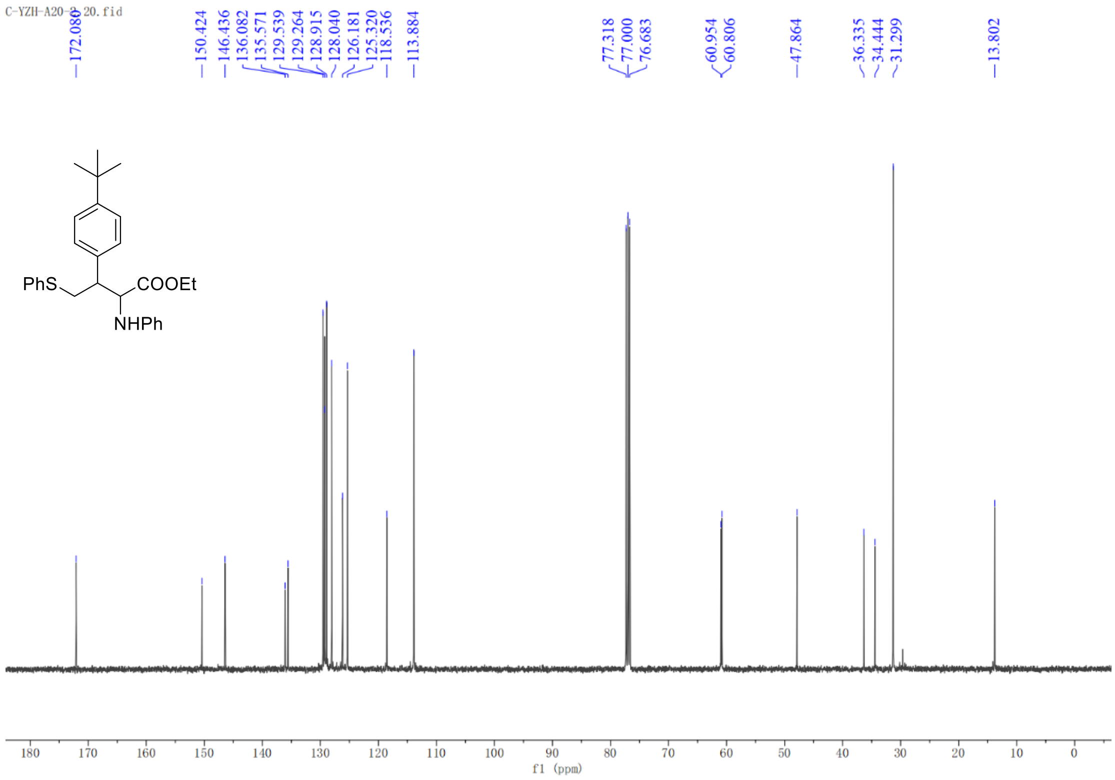

^{1}H **NMR** (400 MHz, $CDCl_3$) spectrum of **34** major isomer

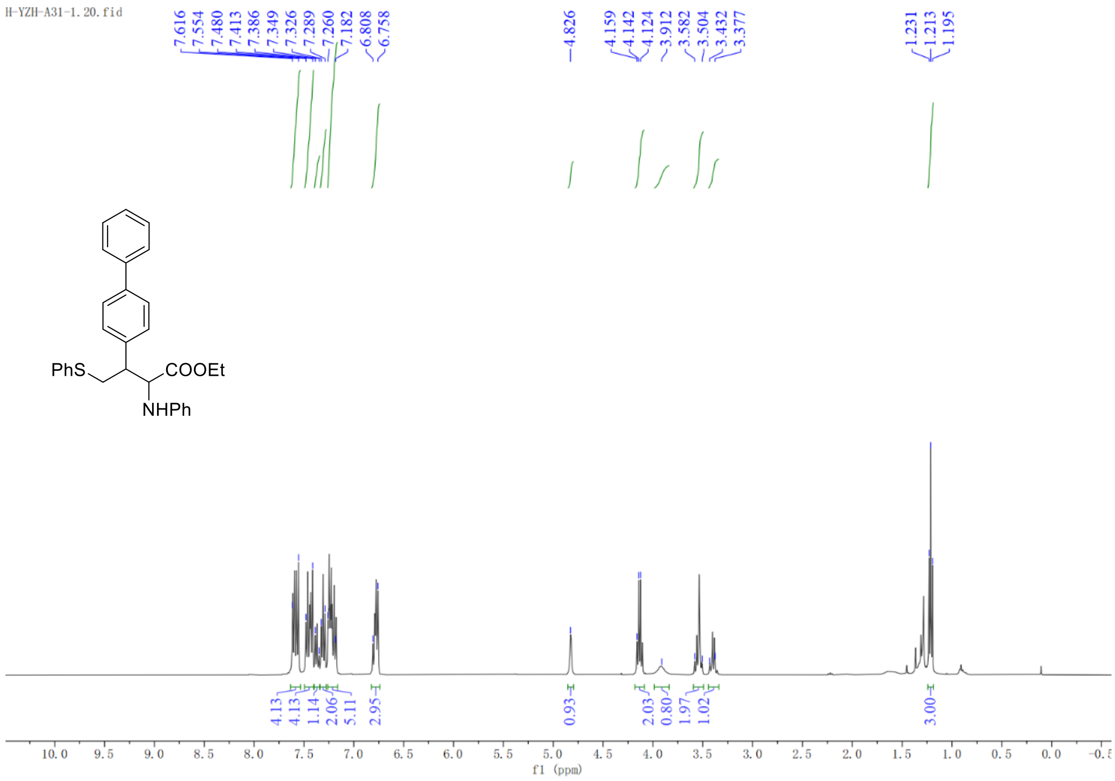

^{13}C NMR (101 MHz, $CDCl_3$) spectrum of **34** major isomer

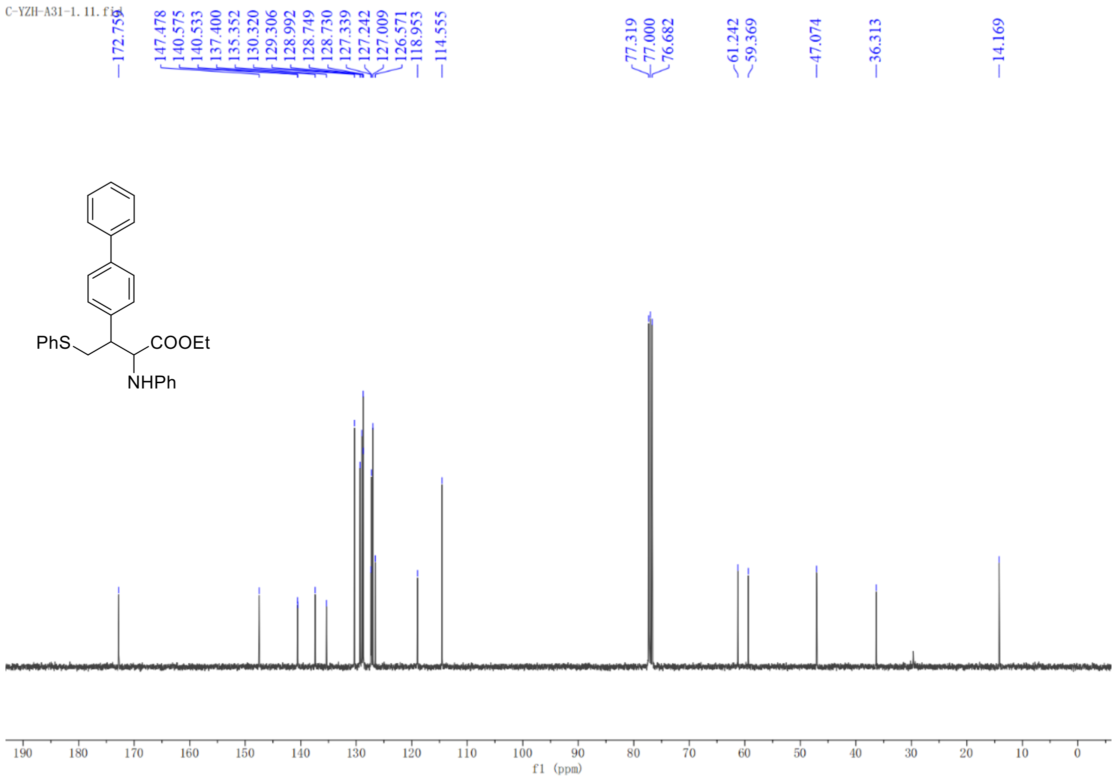

^{1}H NMR (400 MHz, $CDCl_3$) spectrum of **34** minor isomer

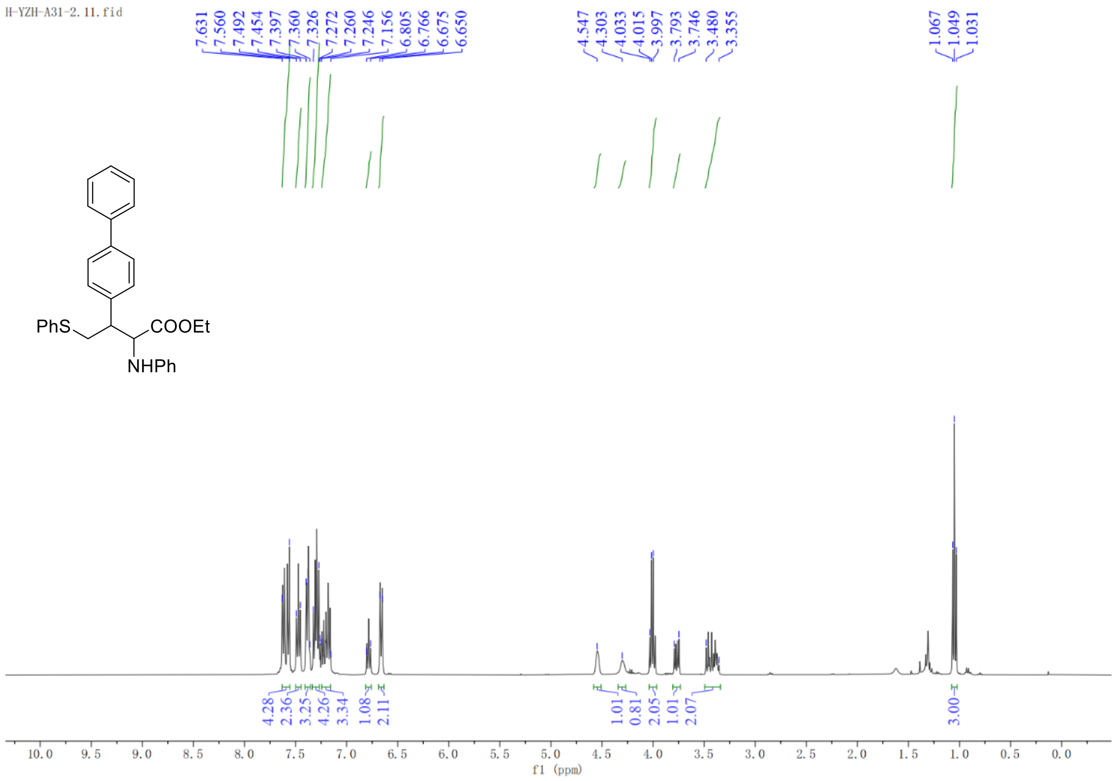

^{13}C NMR (101 MHz, $CDCl_3$) spectrum of **34** minor isomer

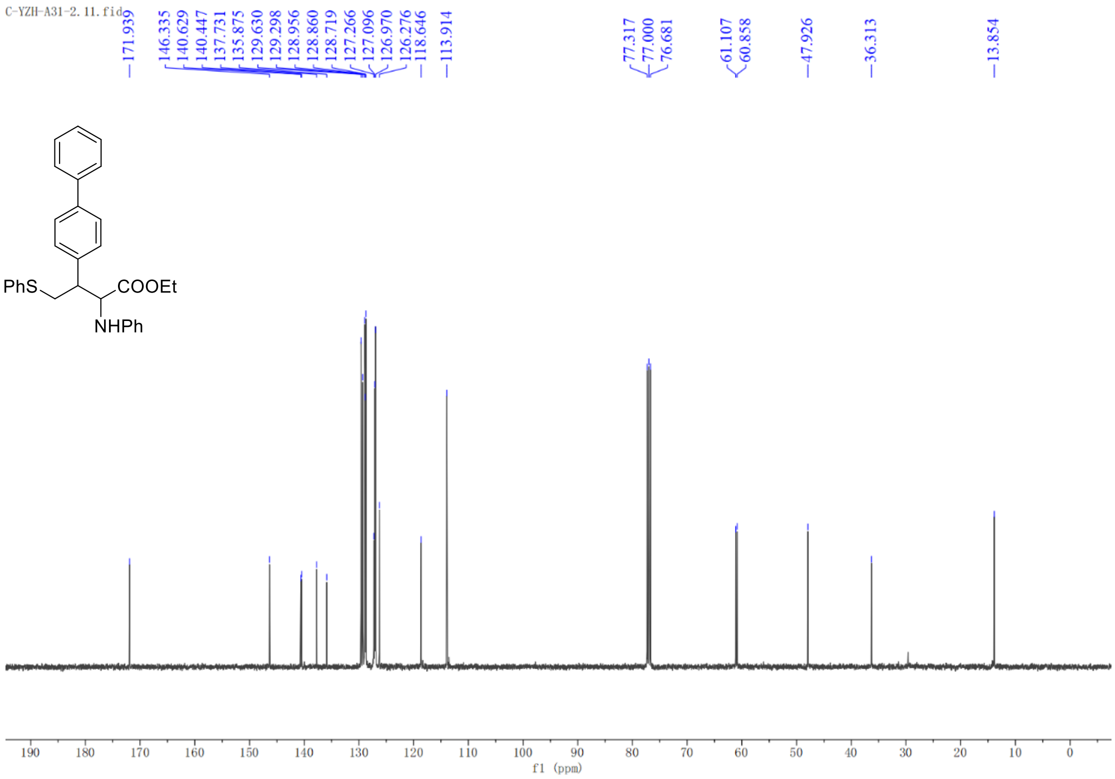

^{1}H NMR (400 MHz, $CDCl_3$) spectrum of **35** major isomer

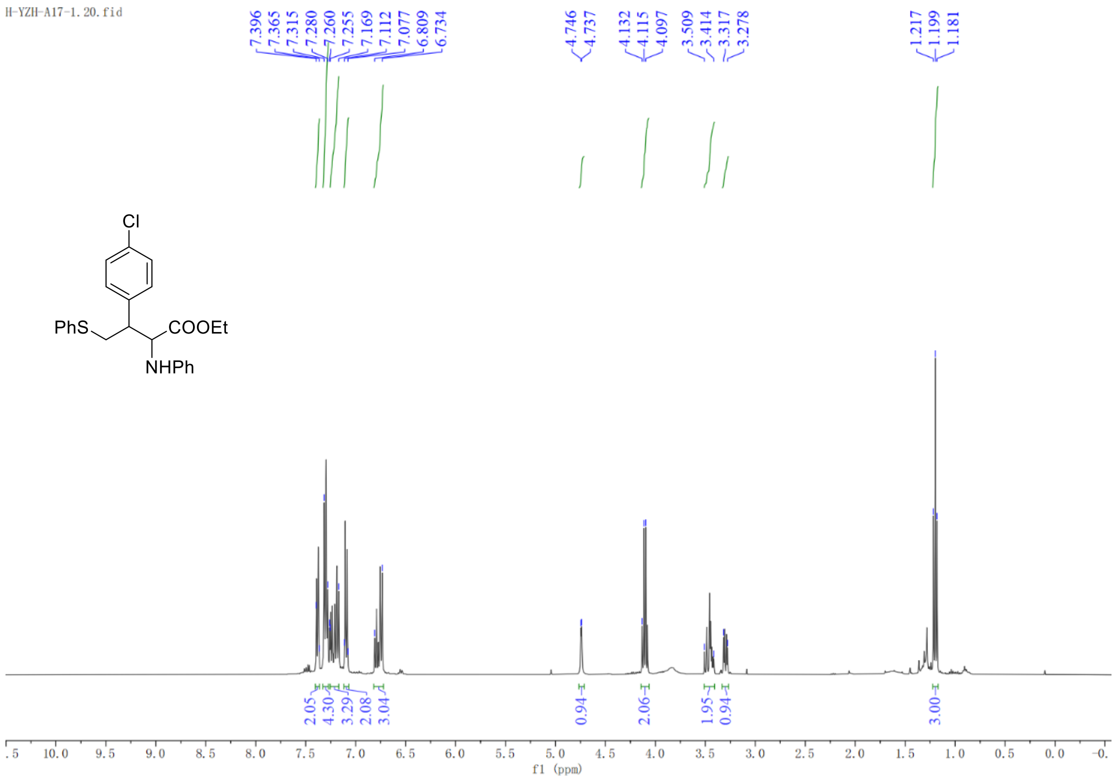

^{13}C NMR (101 MHz, $CDCl_3$) spectrum of **35** major isomer

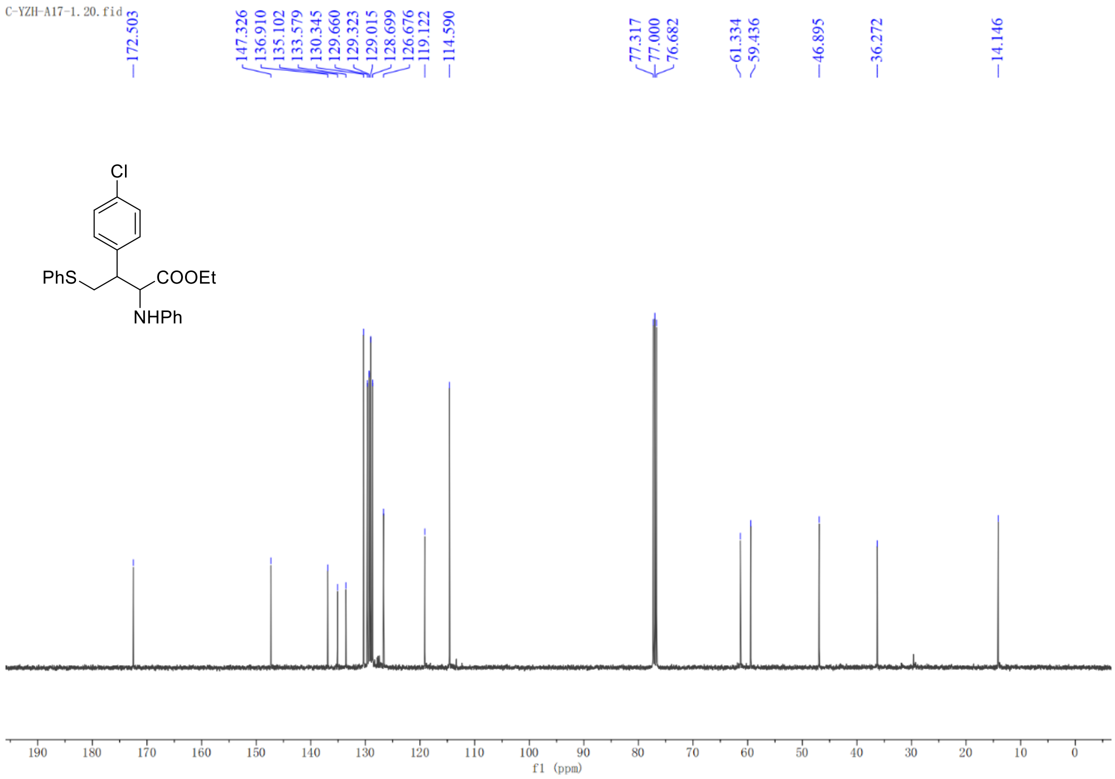

^{1}H NMR (400 MHz, $CDCl_3$) spectrum of **35** minor isomer

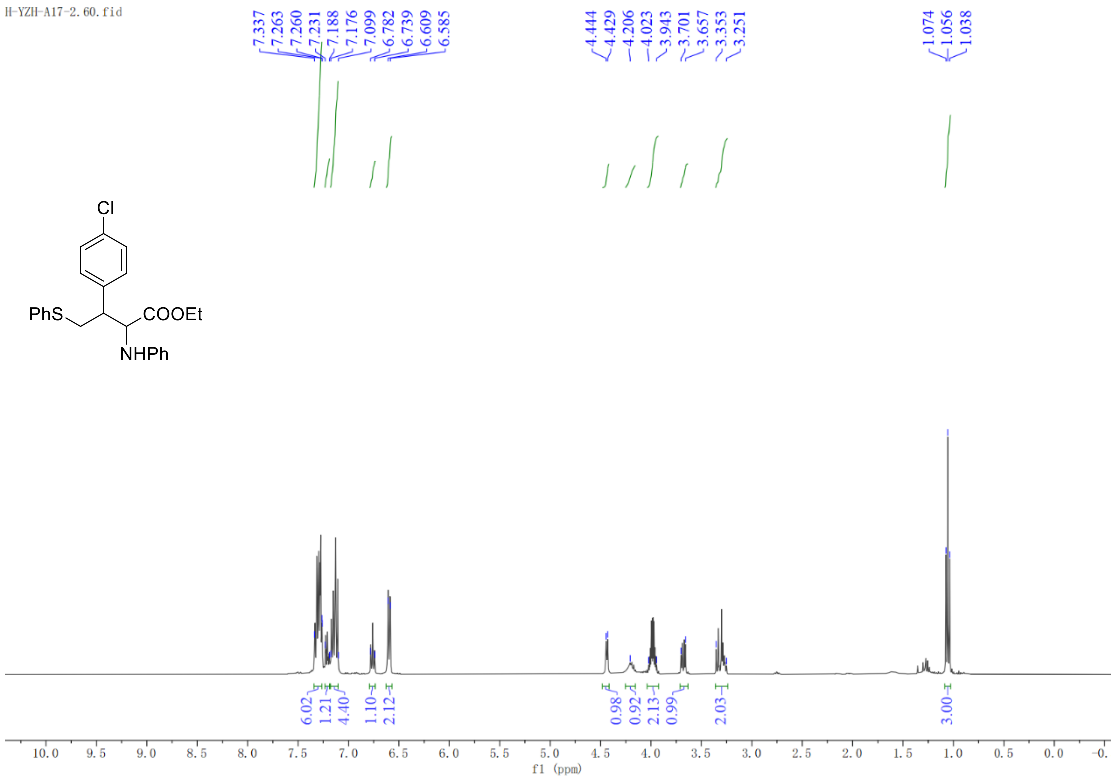

^{13}**C NMR** (101 MHz, $CDCl_3$) spectrum of **35** minor isomer

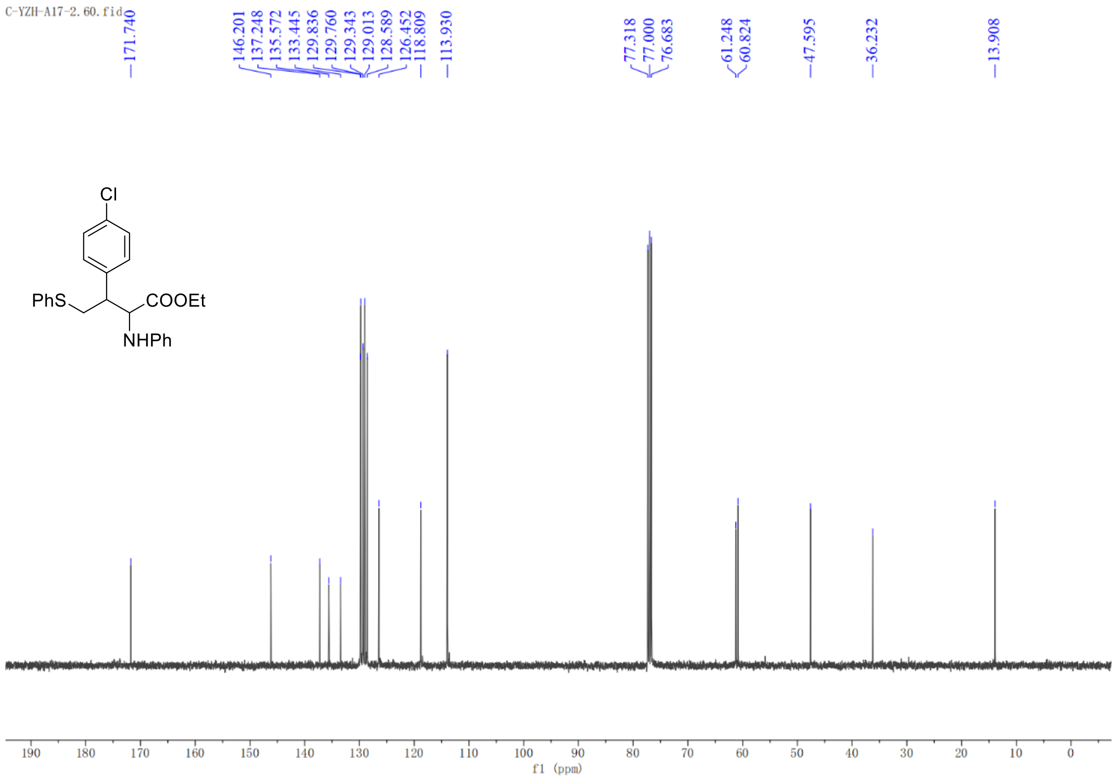

^{1}**H NMR** (400 MHz, $CDCl_3$) spectrum of **36** isomer-1

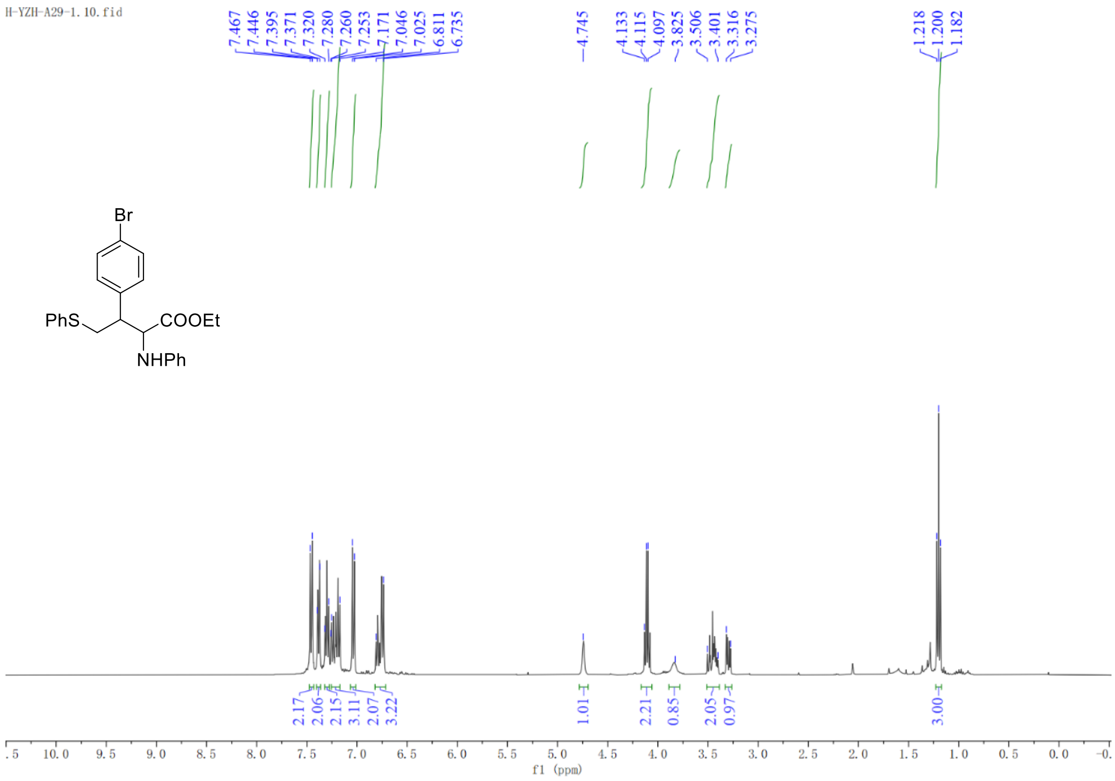

^{13}**C NMR** (101 MHz, CDCl$_3$) spectrum of **36** isomer-1

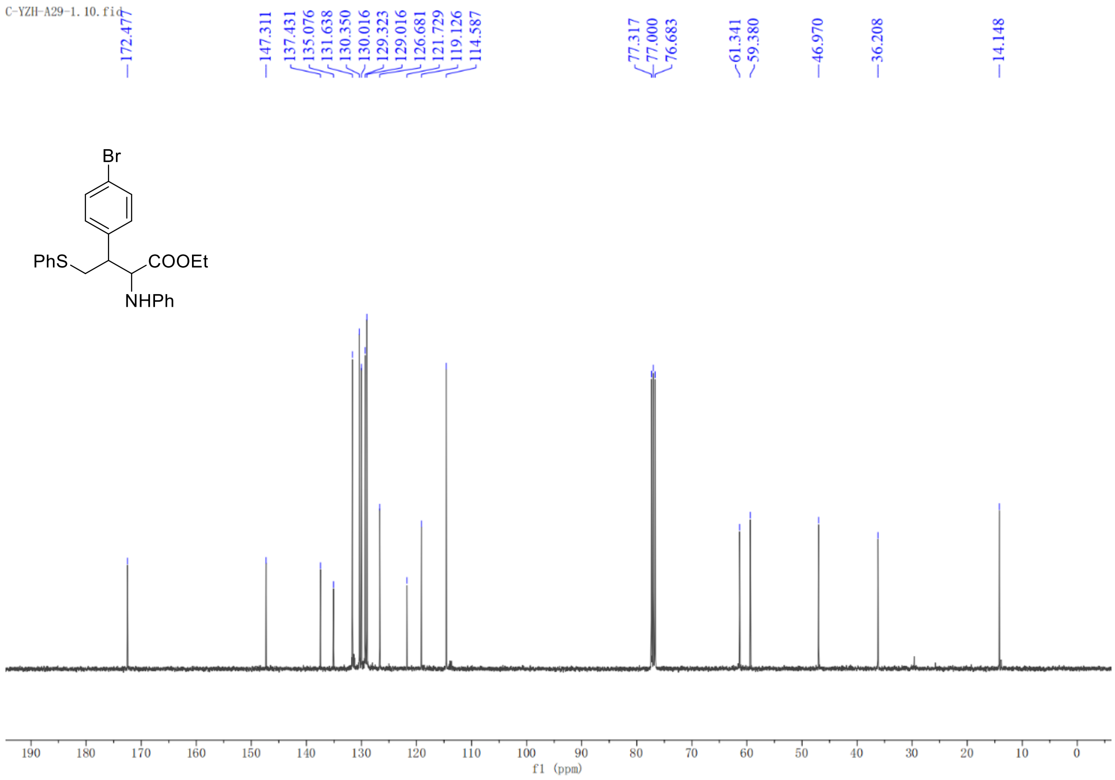

^{1}**H NMR** (400 MHz, CDCl$_3$) spectrum of **36** isomer-2

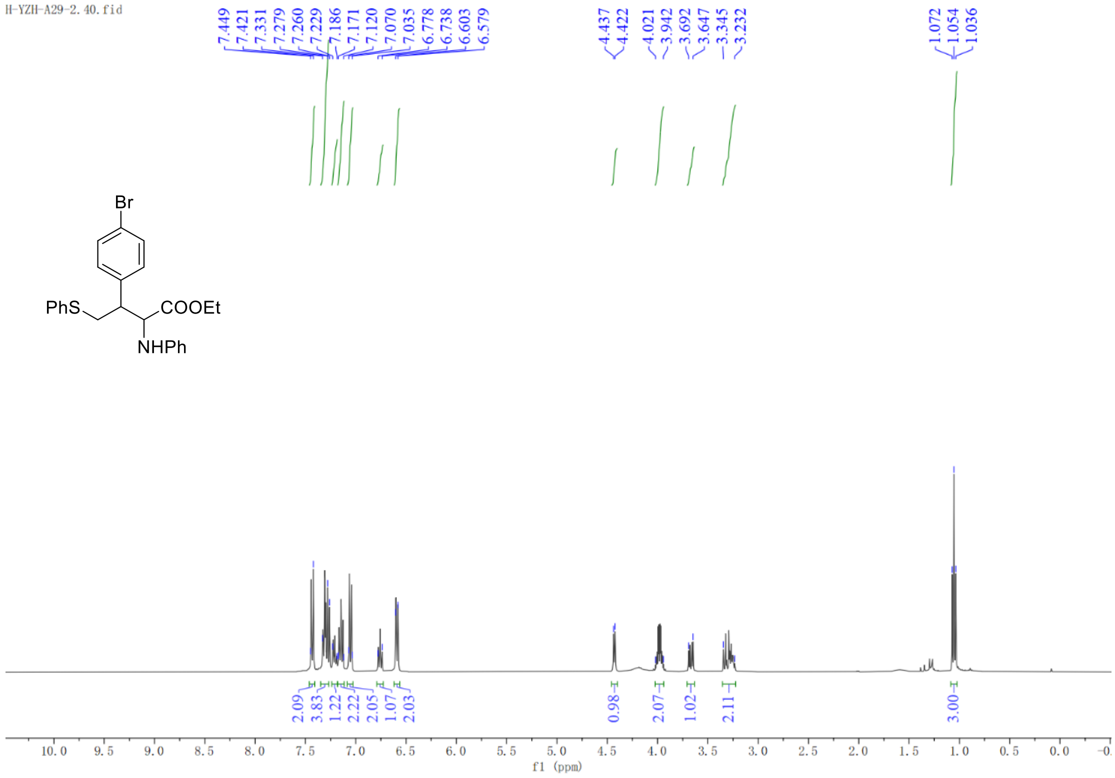

^{13}C NMR (101 MHz, $CDCl_3$) spectrum of **36** isomer-2

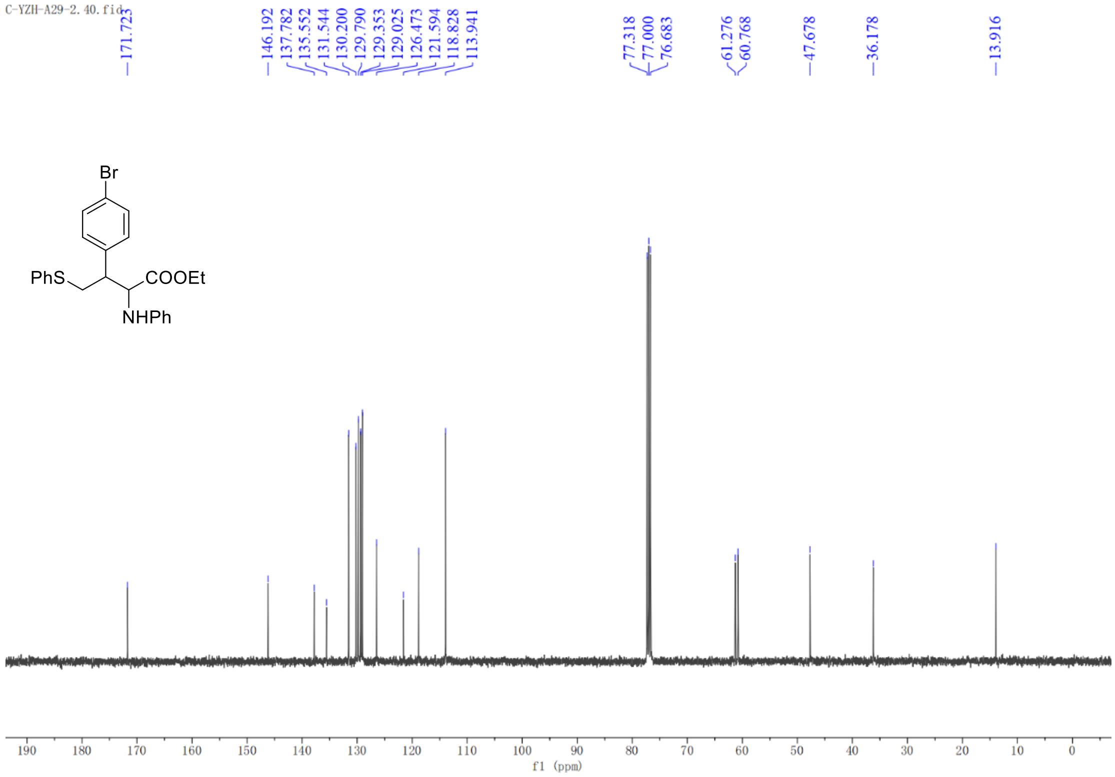

^{1}H NMR (400 MHz, $CDCl_3$) spectrum of **37**

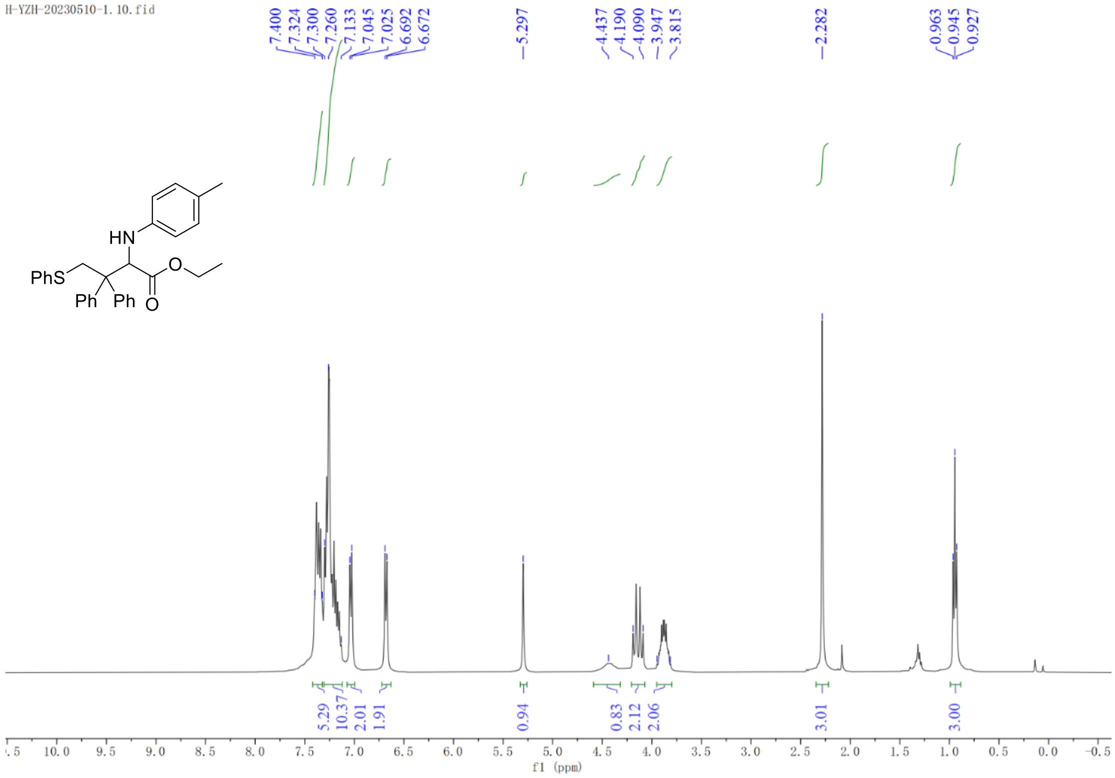

^{13}C NMR (101 MHz, $CDCl_3$) spectrum of **37**

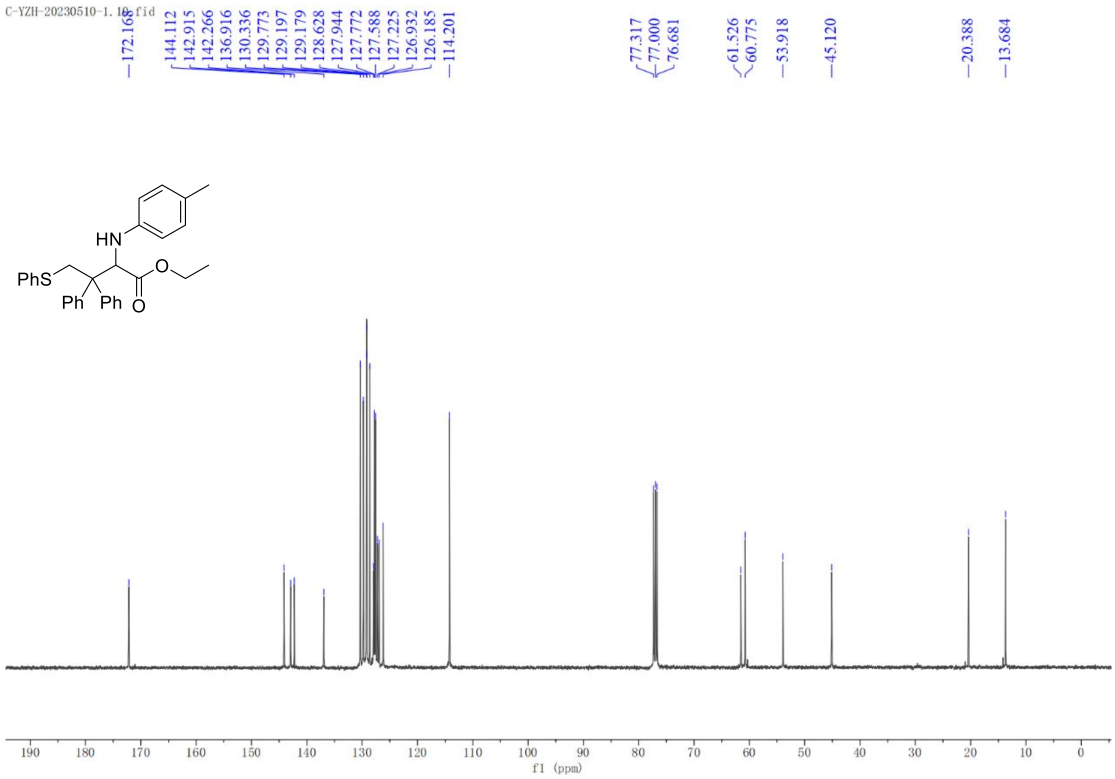

^{1}H NMR (400 MHz, $CDCl_3$) spectrum of **38**

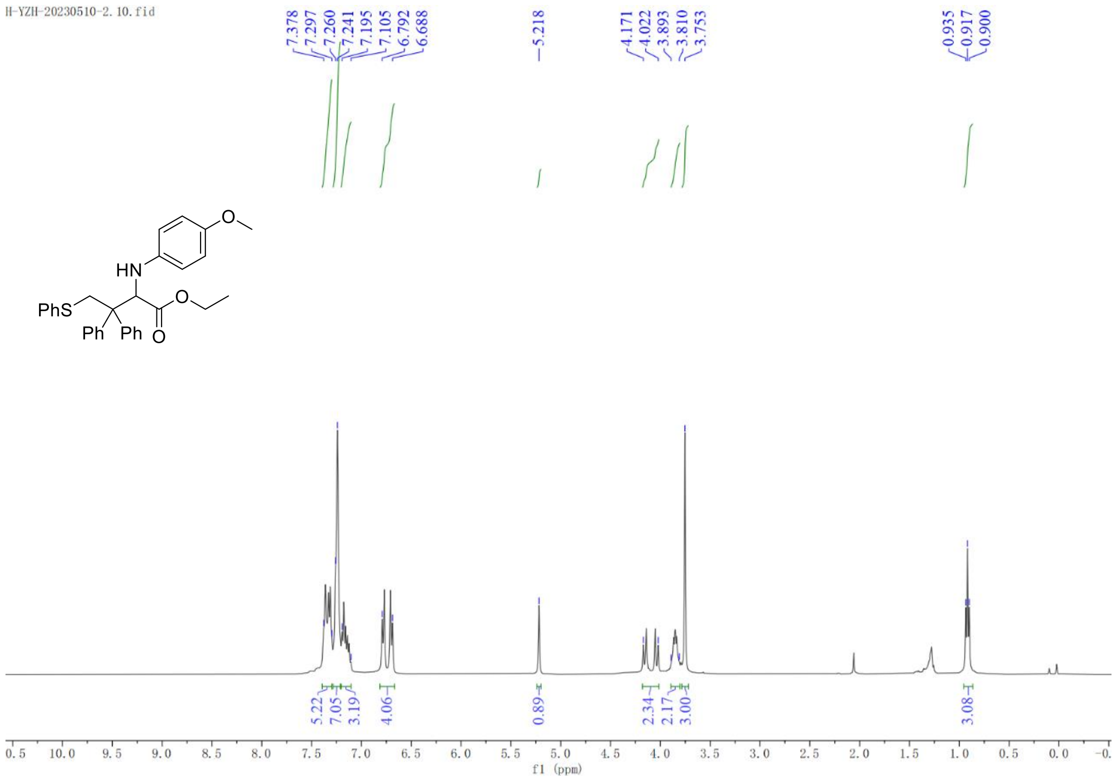

^{13}C NMR (101 MHz, $CDCl_3$) spectrum of **38**

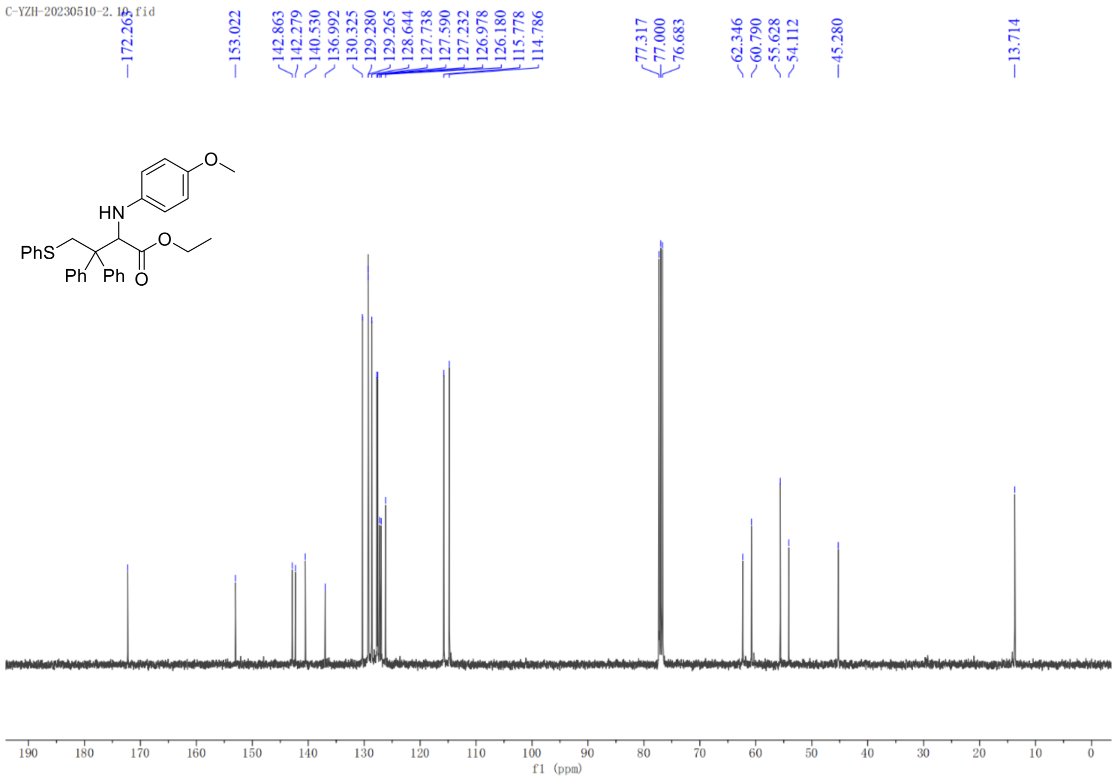

^{1}H NMR (400 MHz, $CDCl_3$) spectrum of **39**

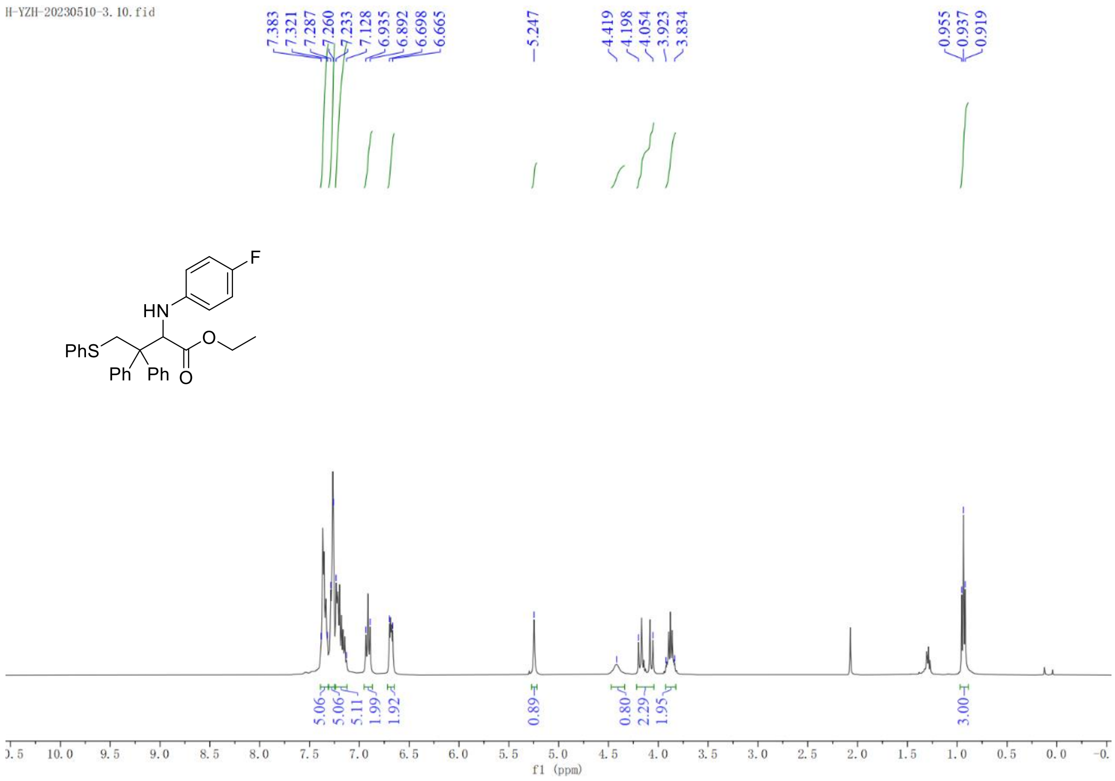

^{13}**C NMR** (101 MHz, $CDCl_3$) spectrum of **39**

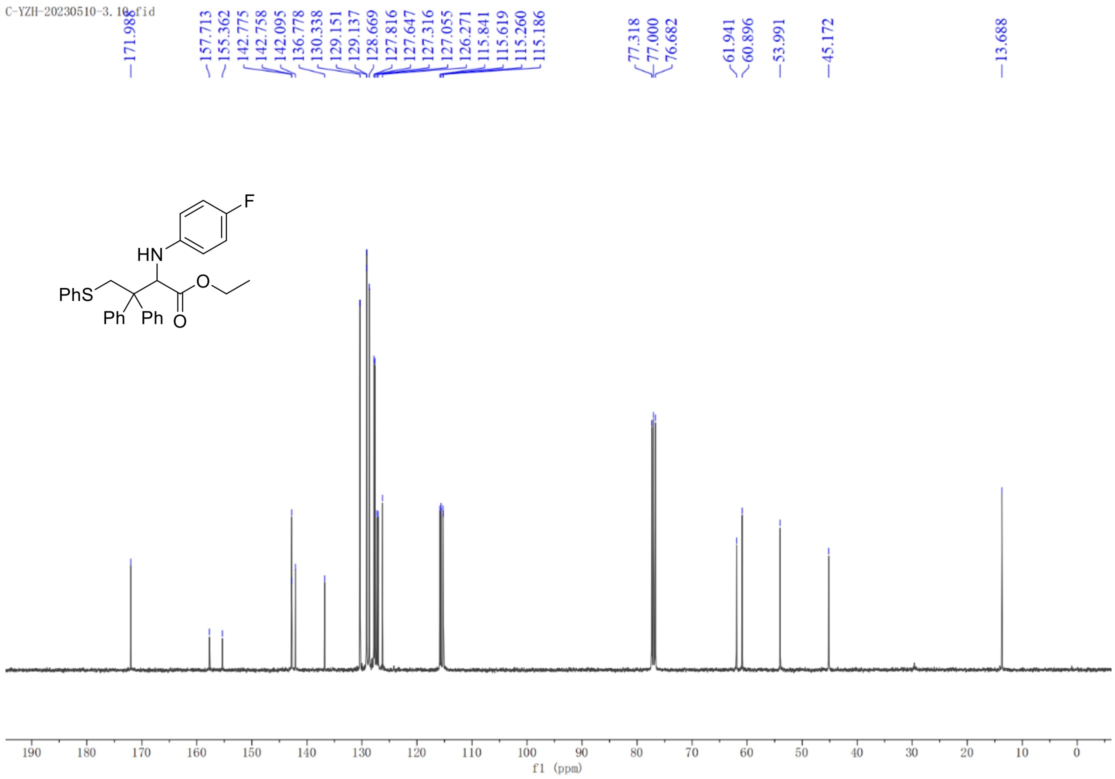

^{19}**F NMR** (376 MHz, $CDCl_3$) spectrum of **39**

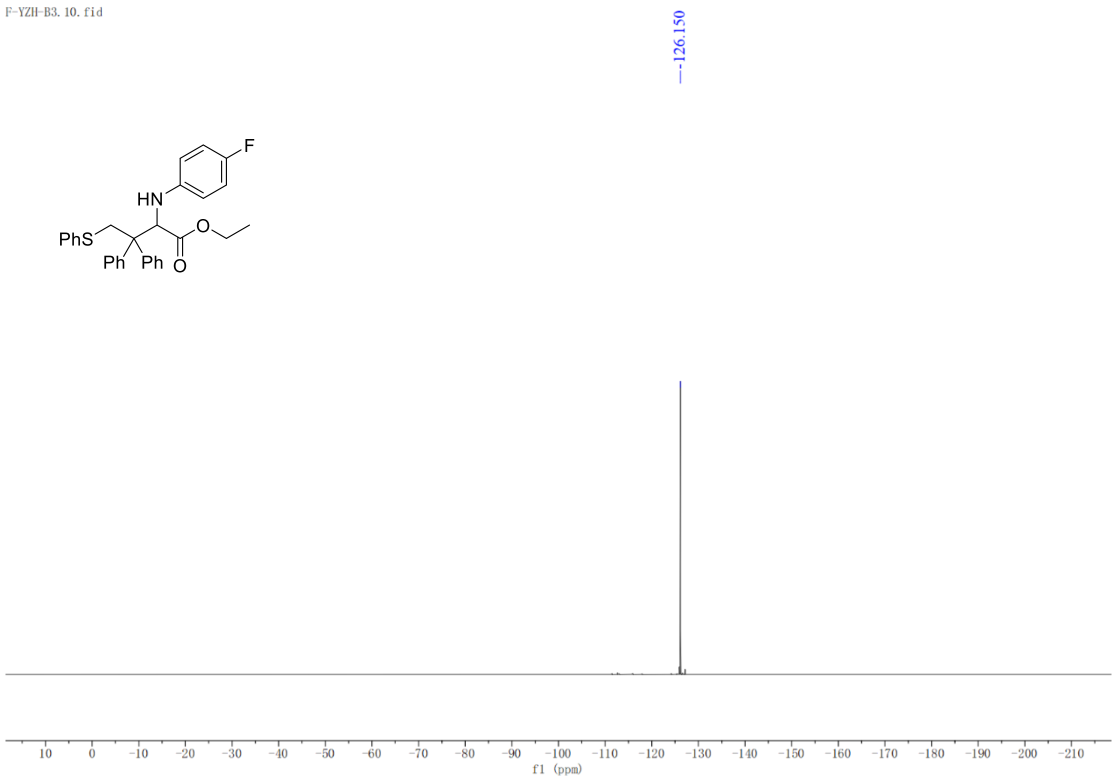

^{1}H NMR (400 MHz, $CDCl_3$) spectrum of **40**

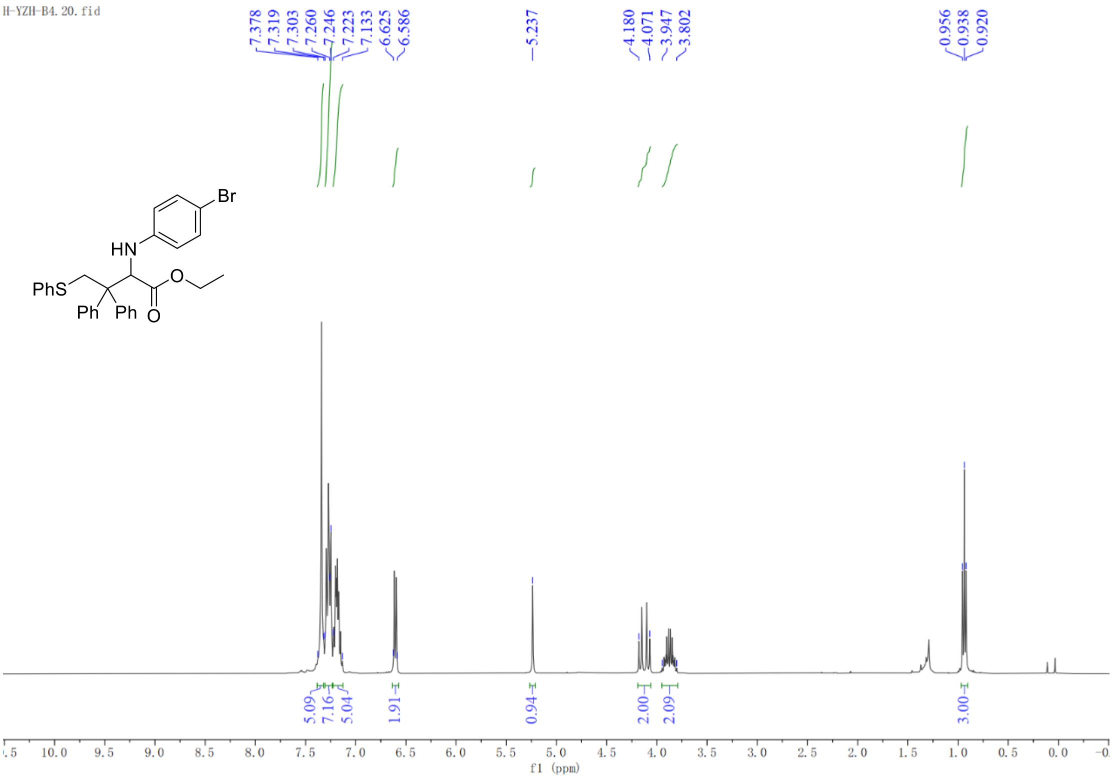

^{13}C NMR (101 MHz, $CDCl_3$) spectrum of **40**

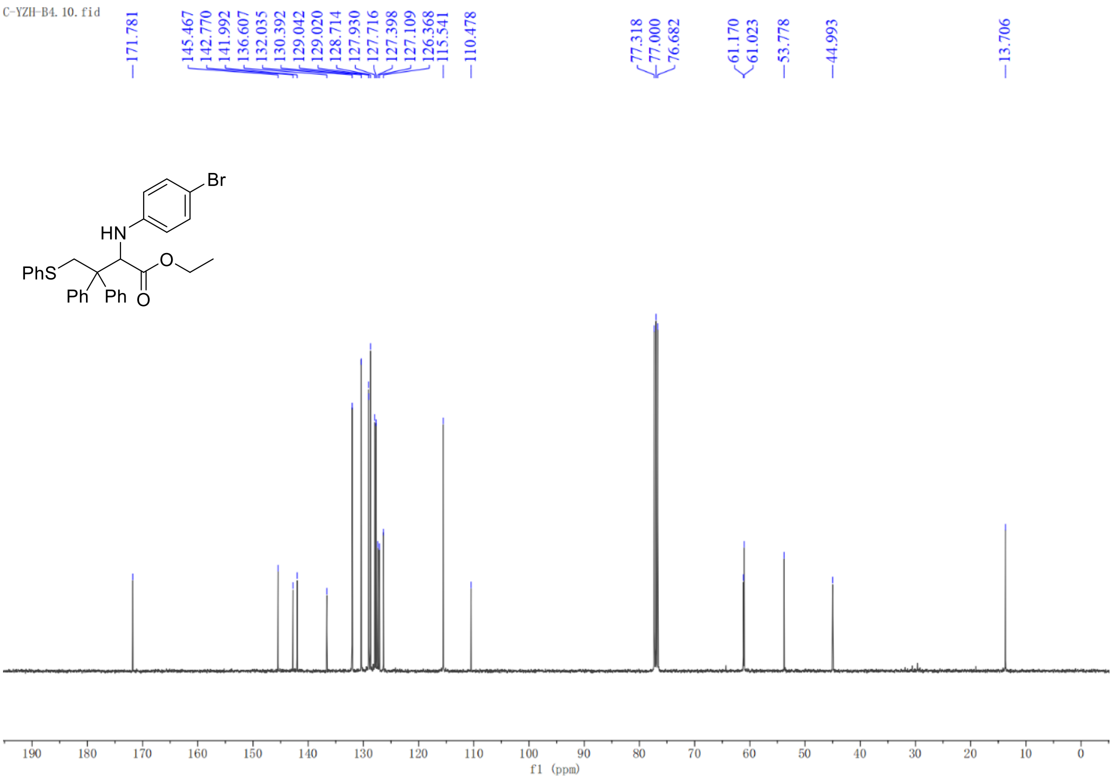

^{1}H NMR (400 MHz, CDCl$_3$) spectrum of **41**

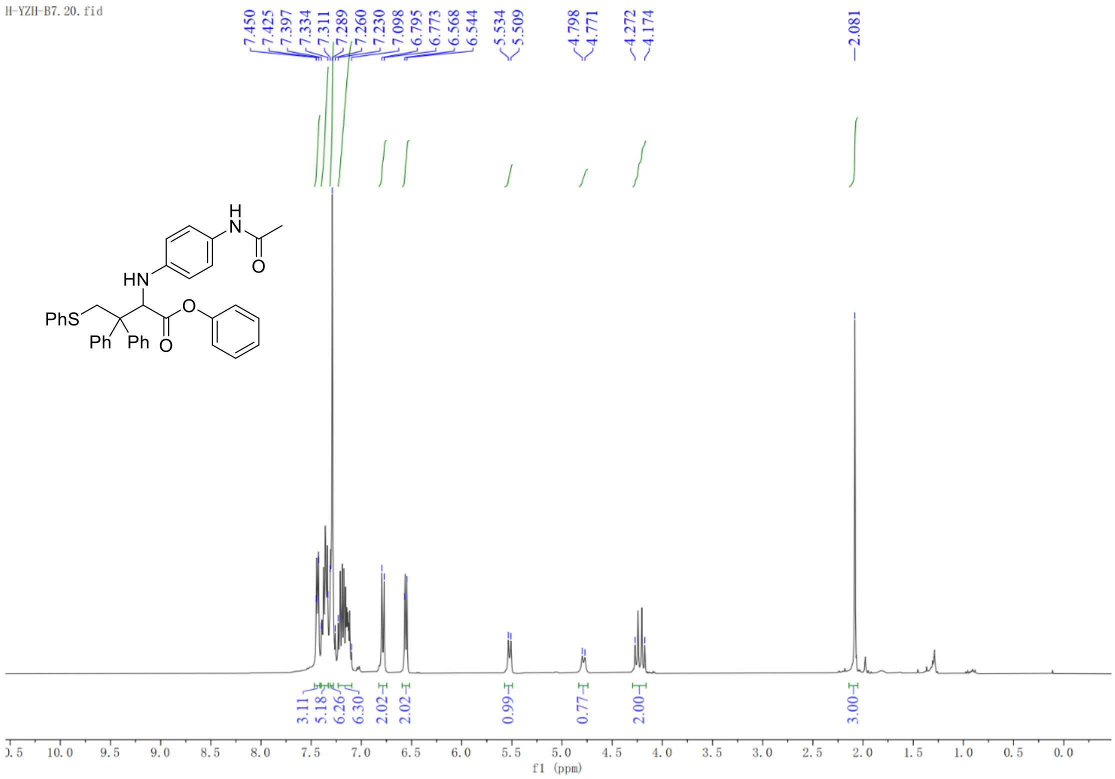

^{13}C NMR (101 MHz, CDCl$_3$) spectrum of **41**

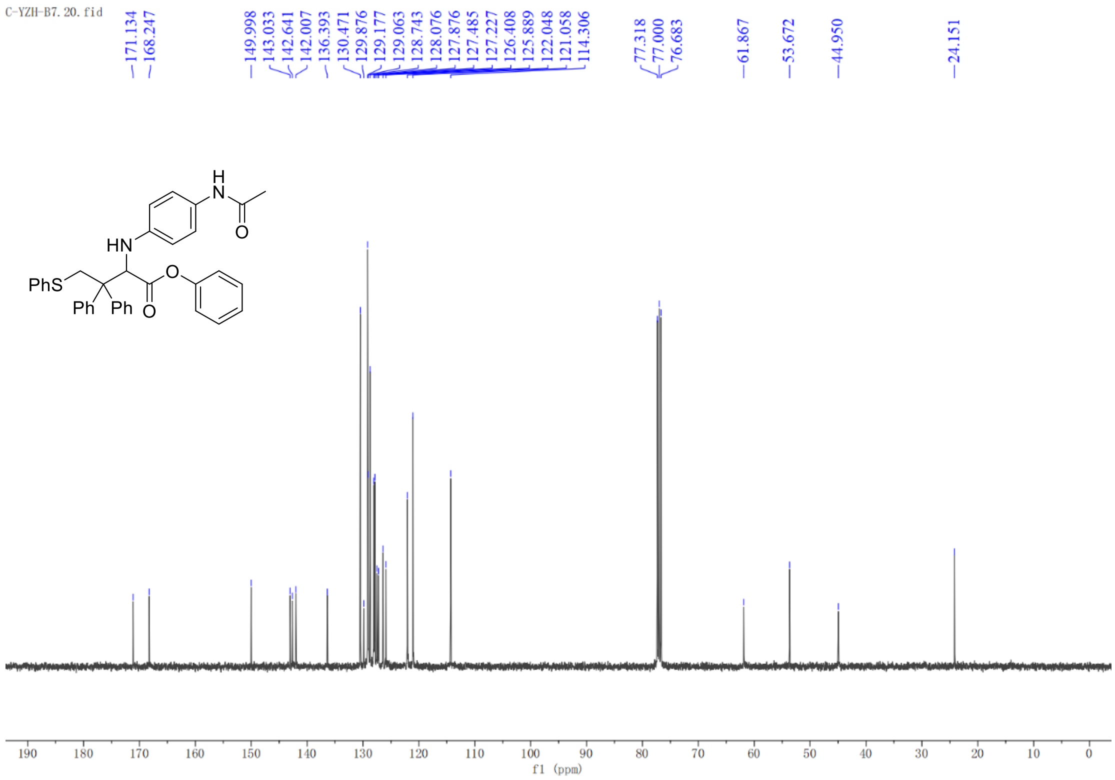

^{1}H NMR (400 MHz, $CDCl_3$) spectrum of **42**

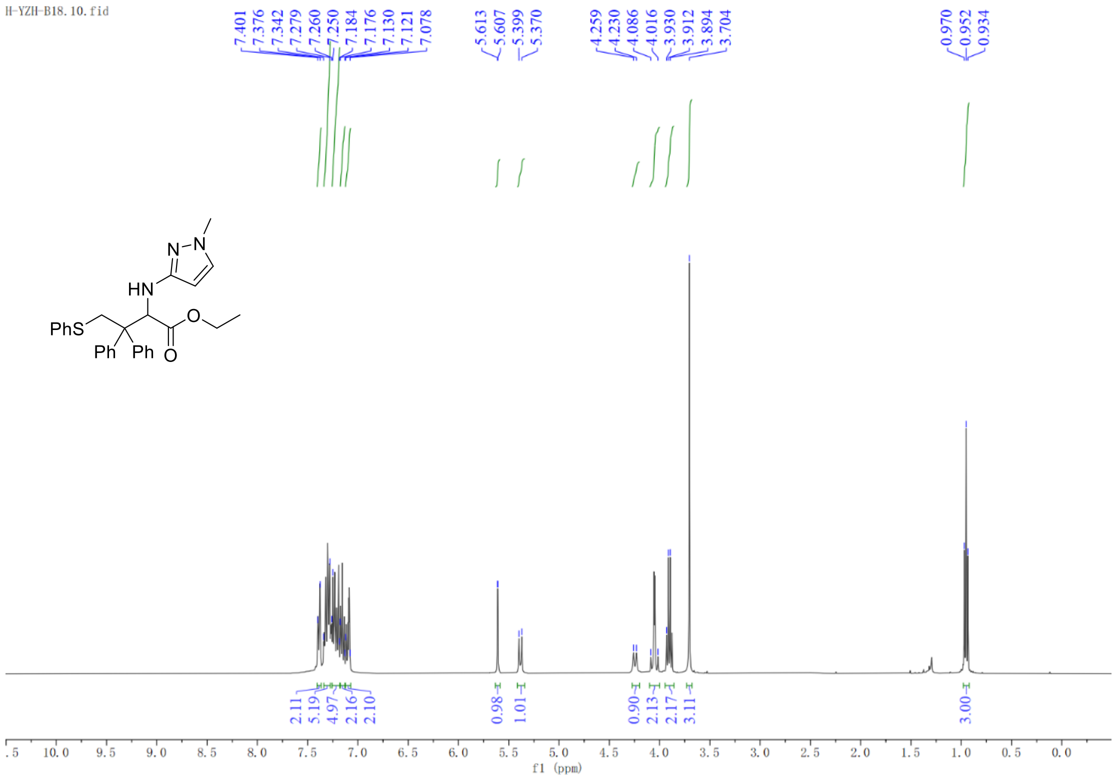

^{13}C NMR (101 MHz, $CDCl_3$) spectrum of **42**

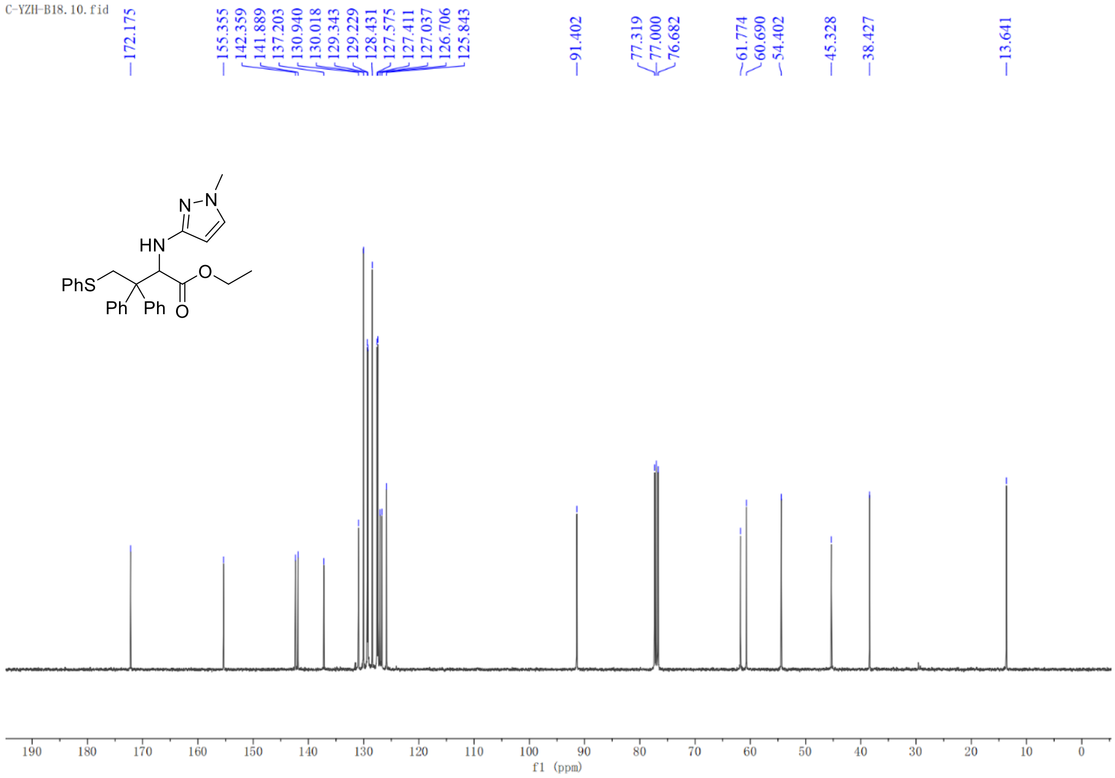

^{1}**H NMR** (400 MHz, $CDCl_3$) spectrum of **43**

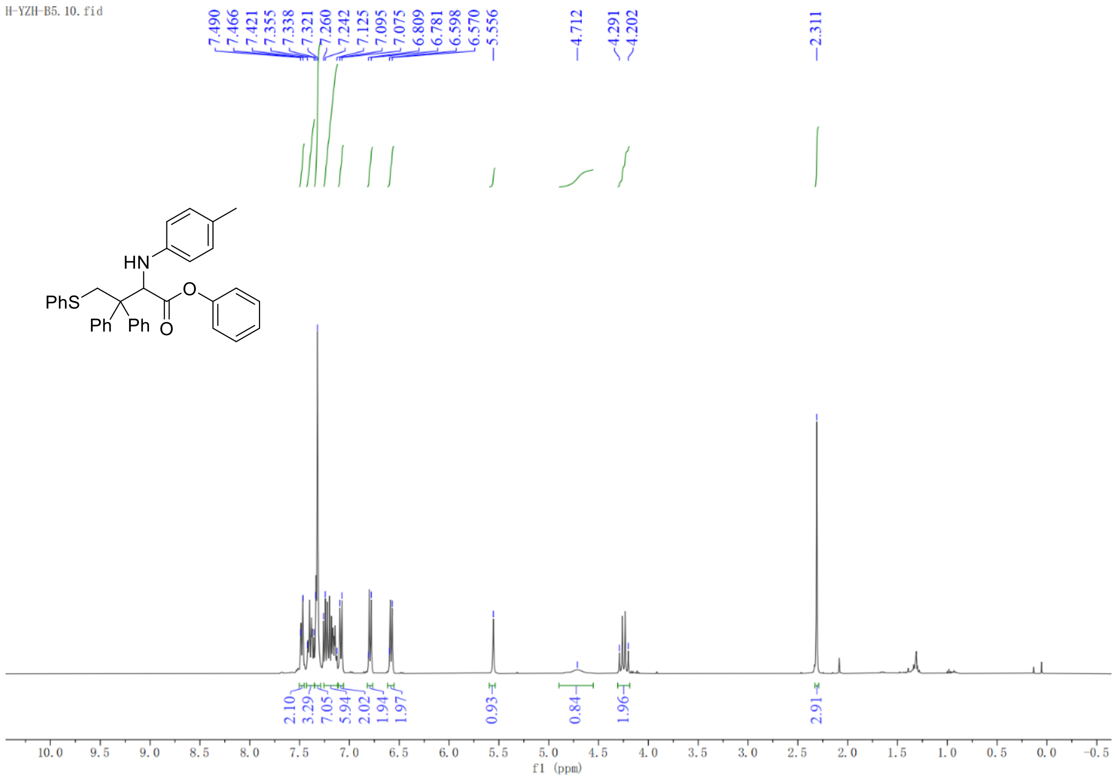

^{13}**C NMR** (101 MHz, $CDCl_3$) spectrum of **43**

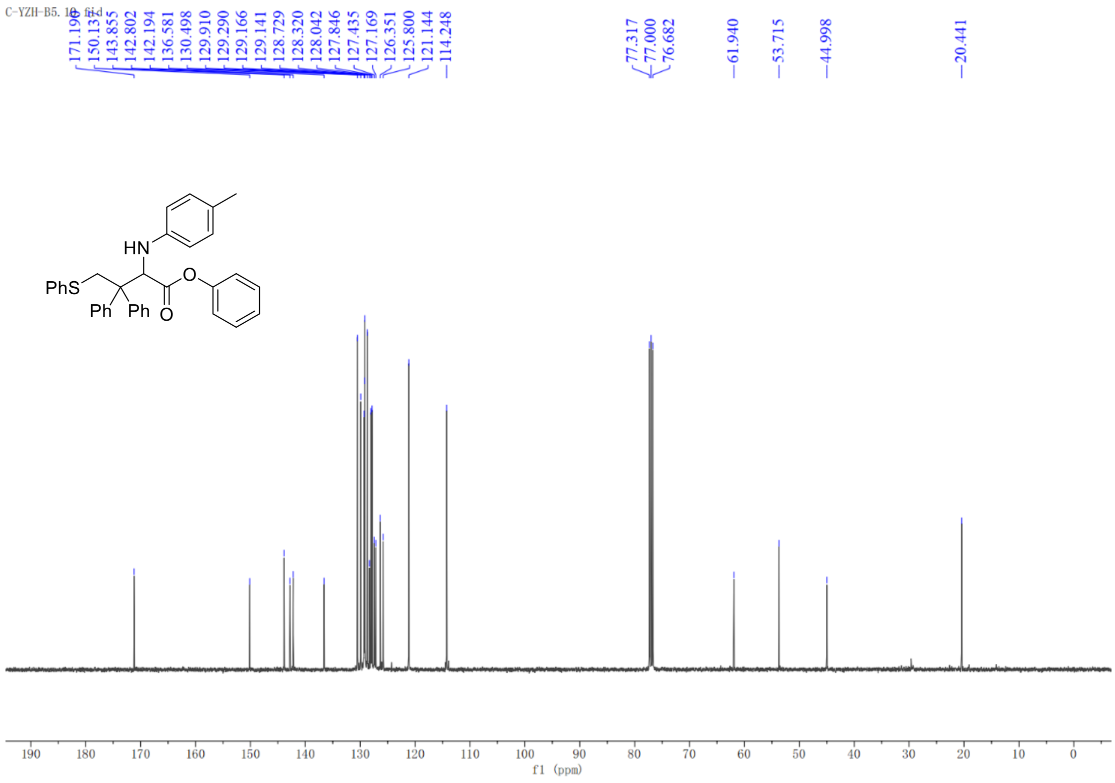

^{1}H NMR (400 MHz, $CDCl_3$) spectrum of **44**

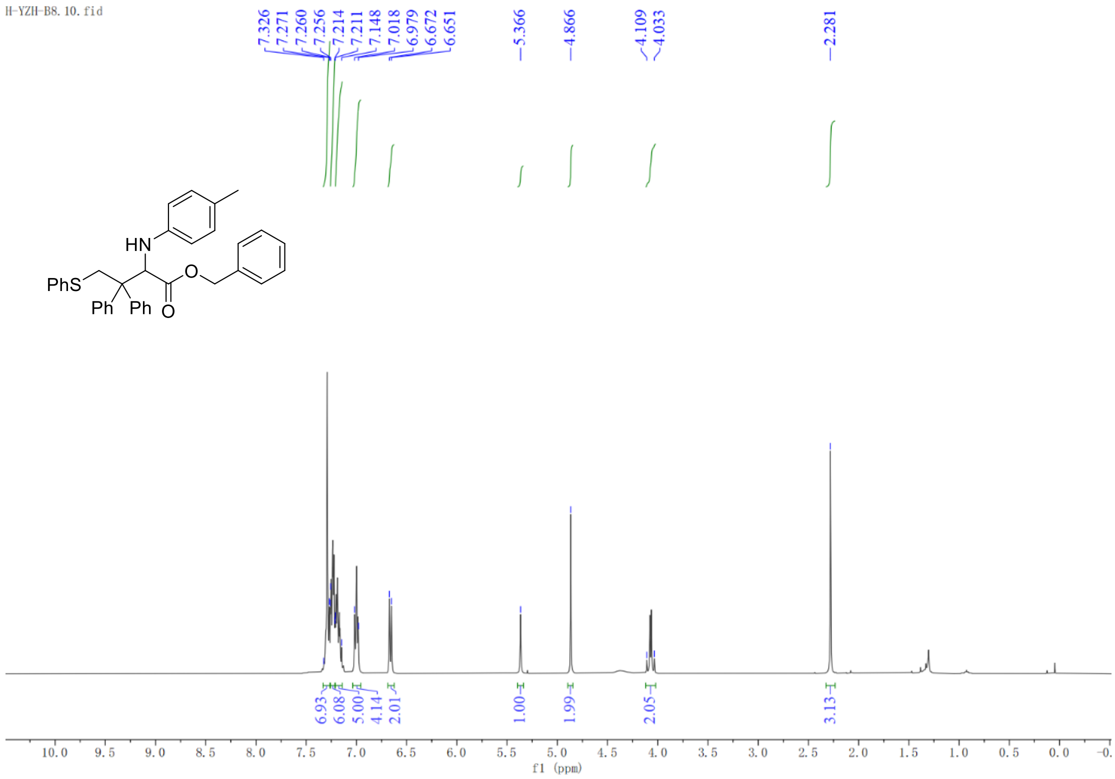

^{13}C NMR (101 MHz, $CDCl_3$) spectrum of **44**

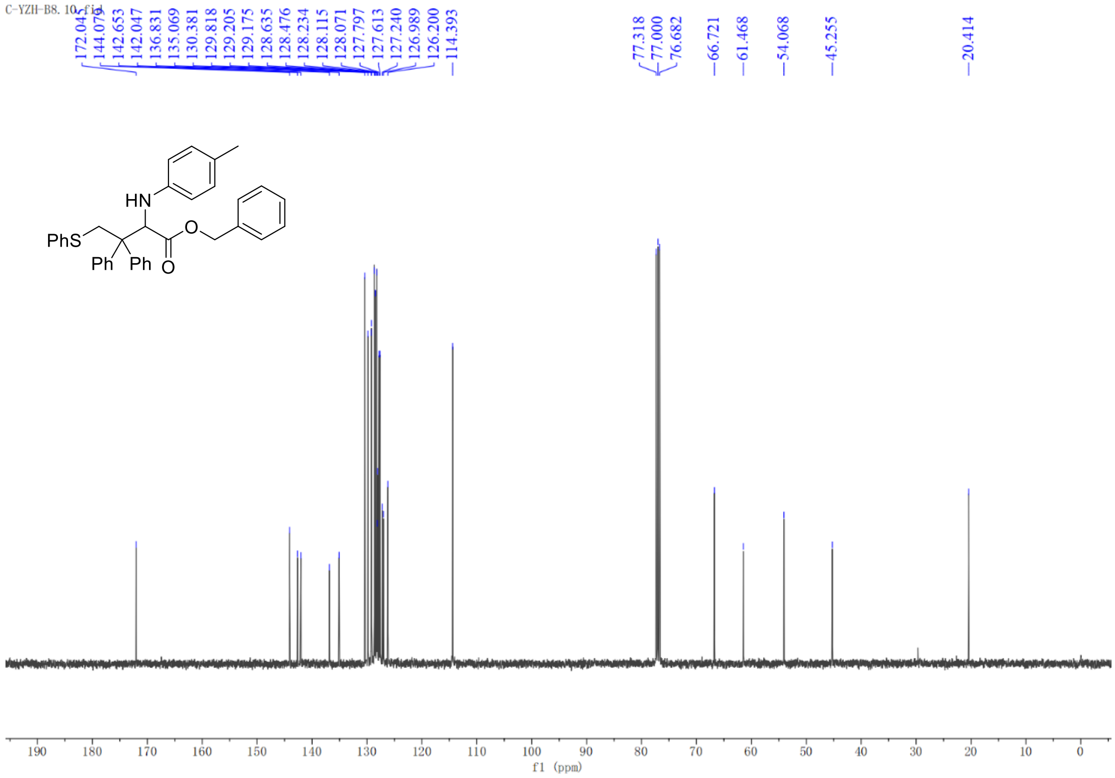

^{1}H NMR (400 MHz, $CDCl_3$) spectrum of **45**

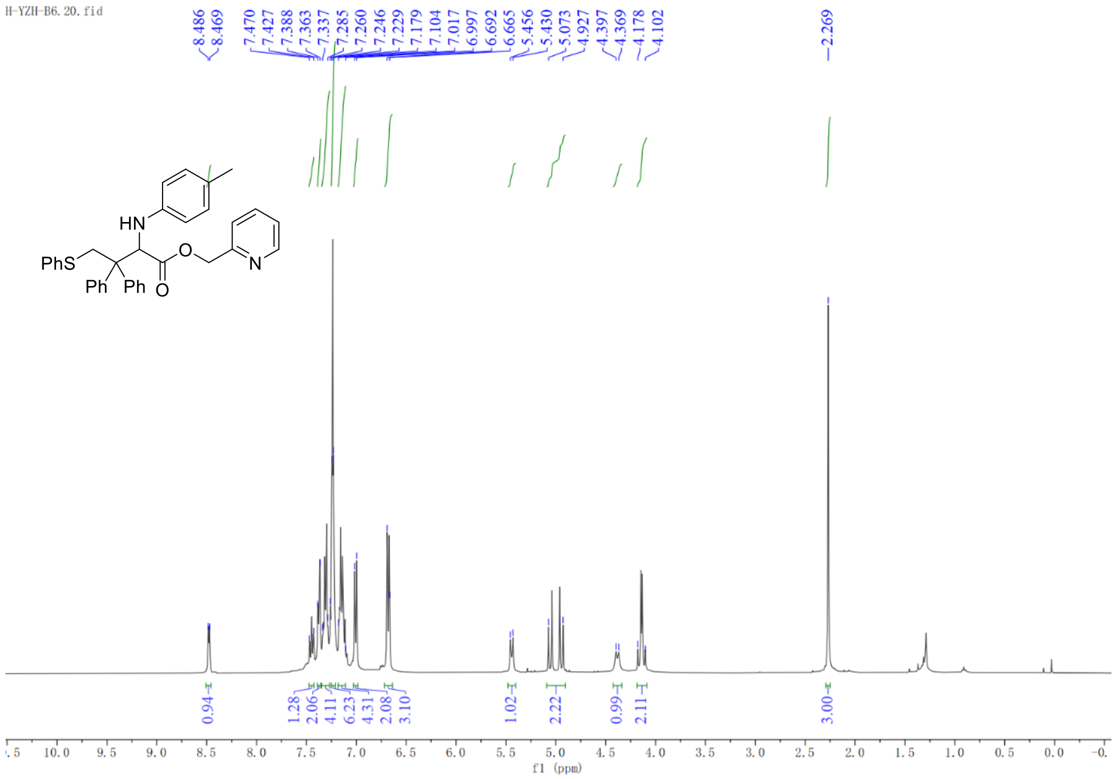

^{13}C NMR (101 MHz, $CDCl_3$) spectrum of **45**

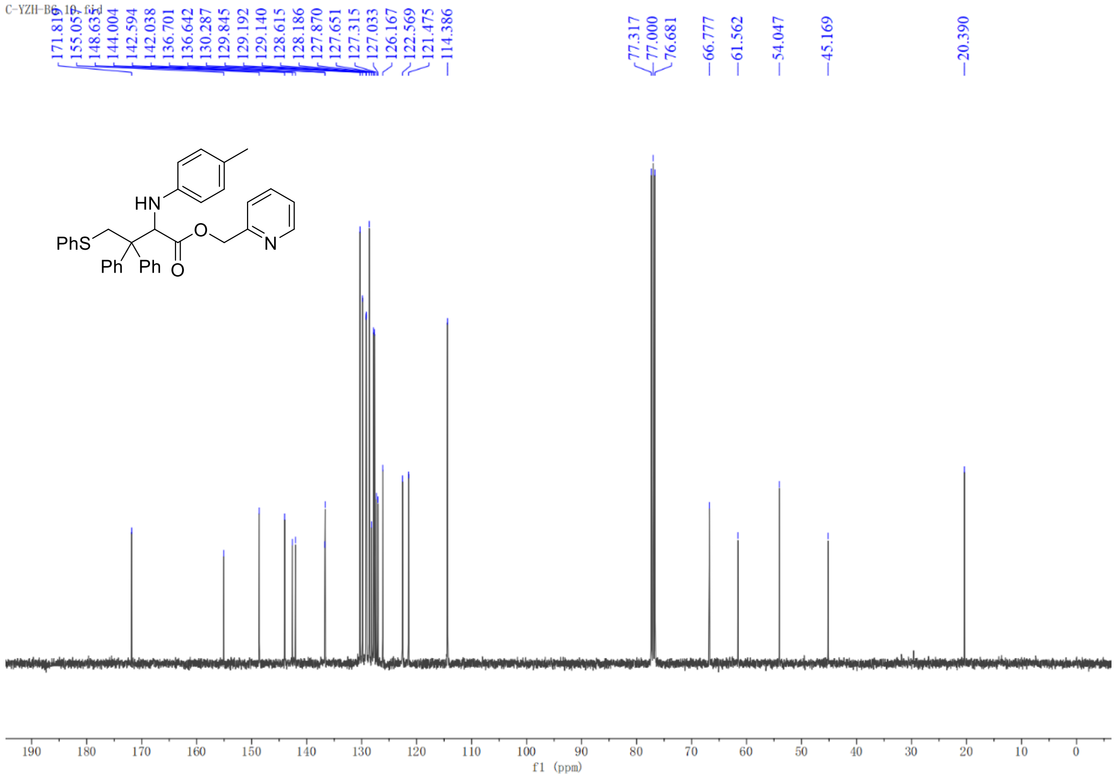

^{1}H NMR (400 MHz, $CDCl_3$) spectrum of **46**

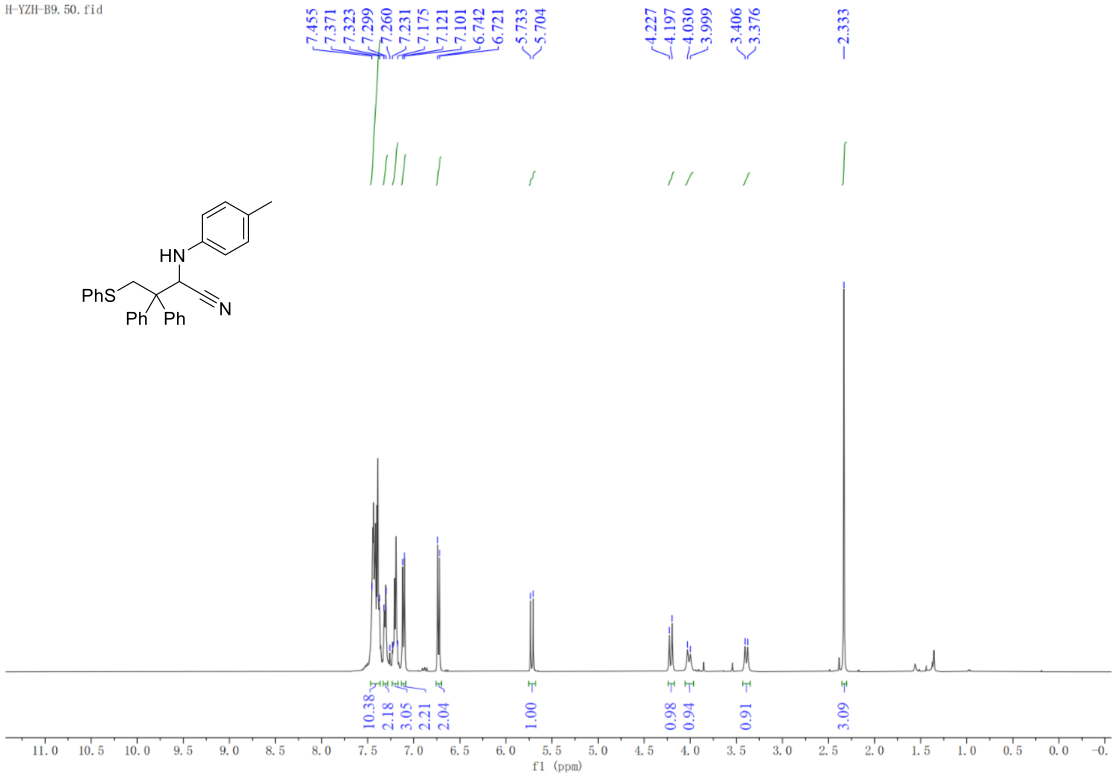

^{13}C NMR (101 MHz, $CDCl_3$) spectrum of **46**

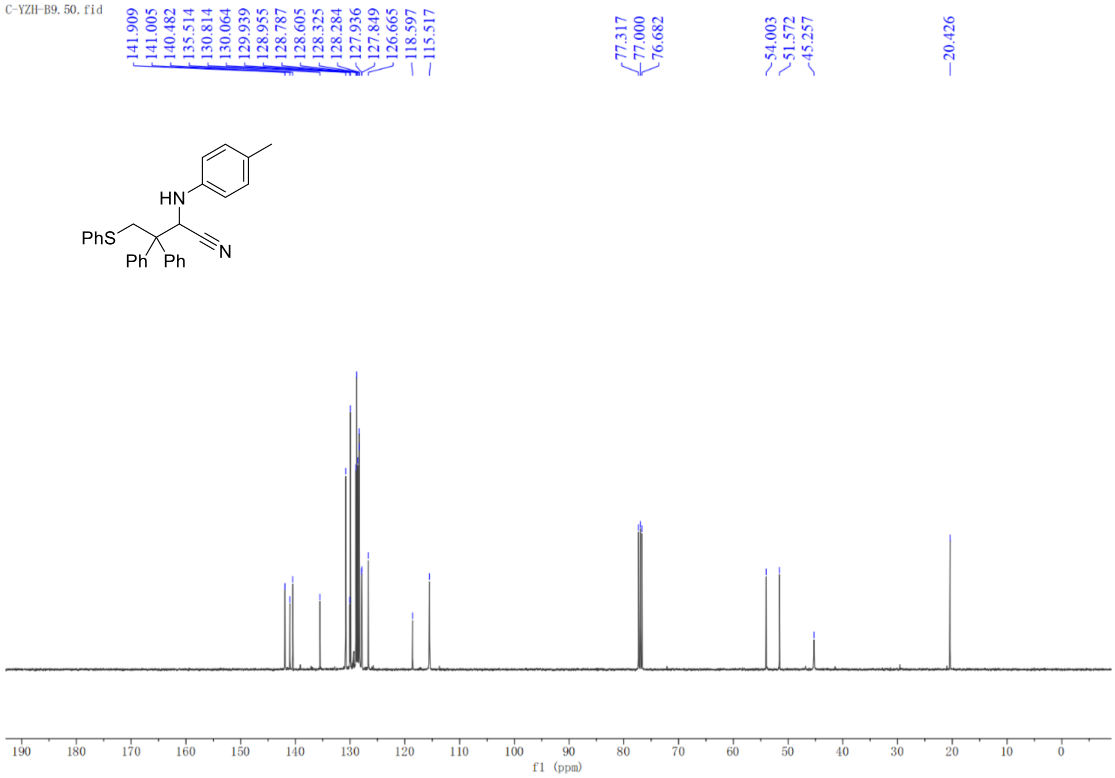

^{1}H NMR (400 MHz, $CDCl_3$) spectrum of **47**

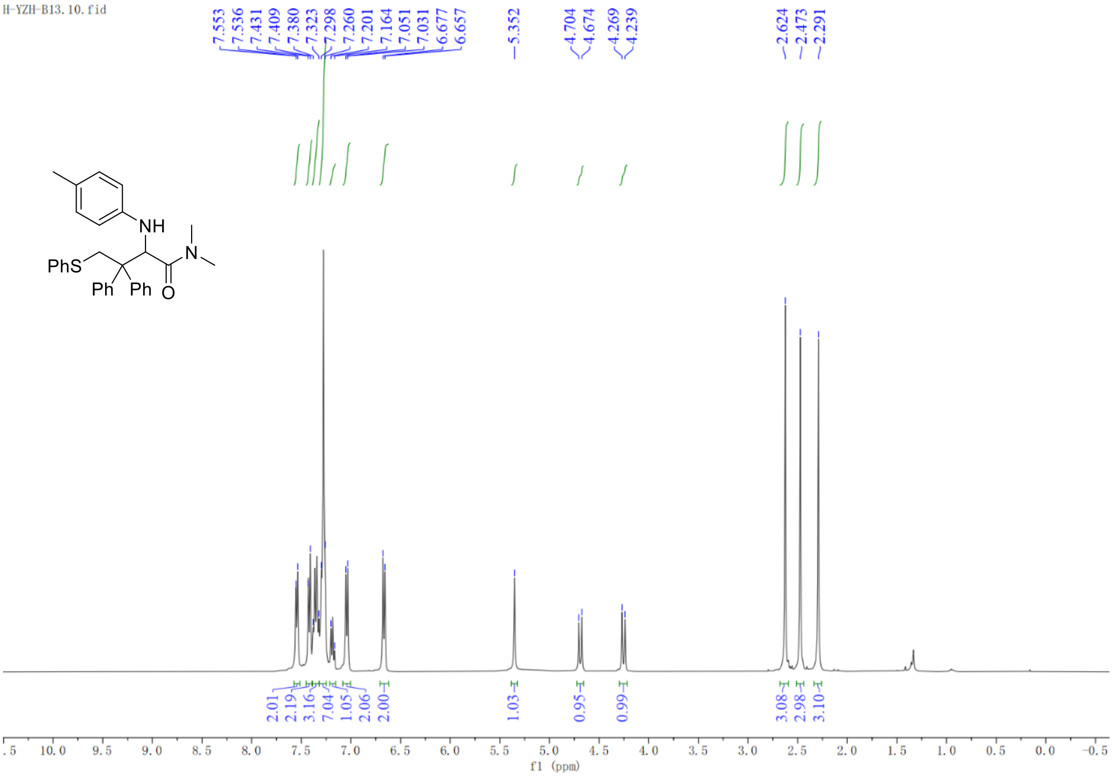

^{13}C NMR (101 MHz, $CDCl_3$) spectrum of **47**

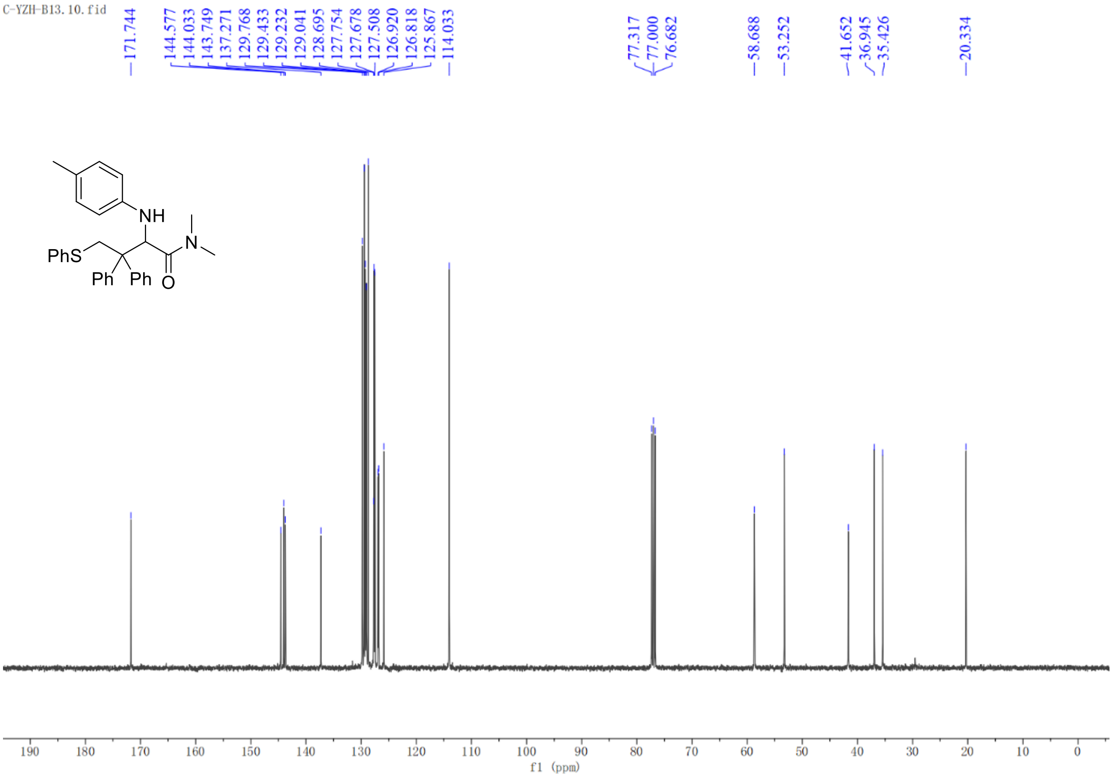

^{1}H NMR (400 MHz, $CDCl_3$) spectrum of **48**

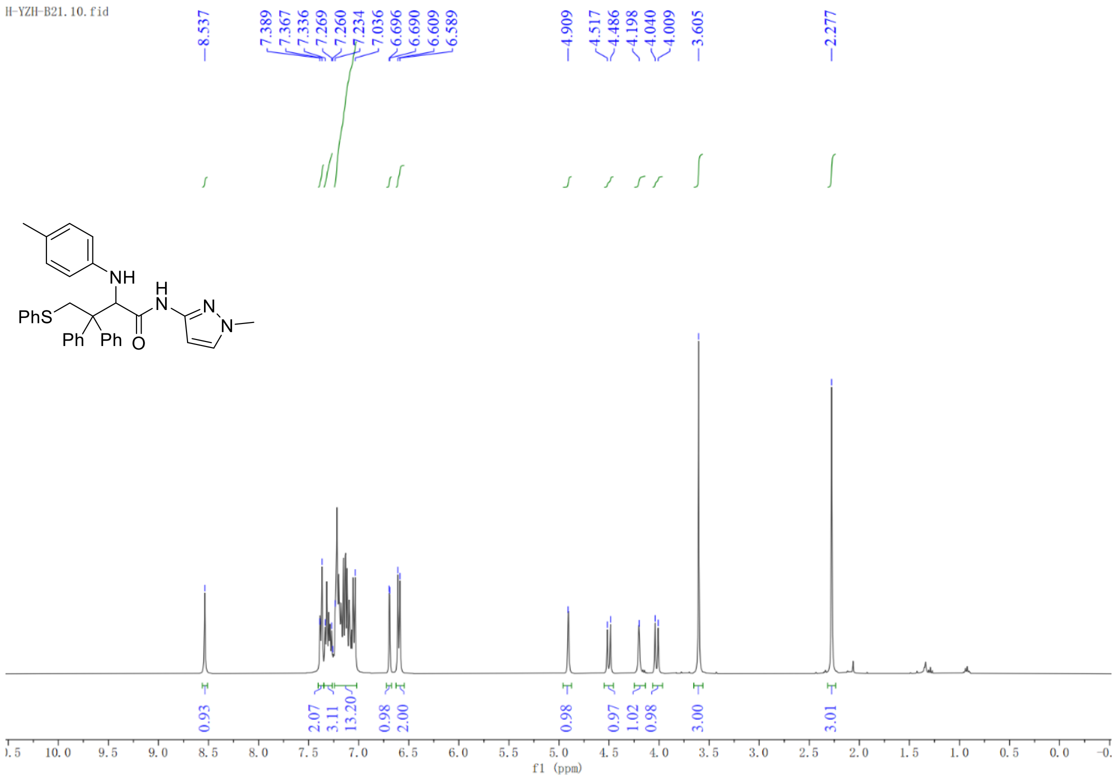

^{13}C NMR (101 MHz, $CDCl_3$) spectrum of **48**

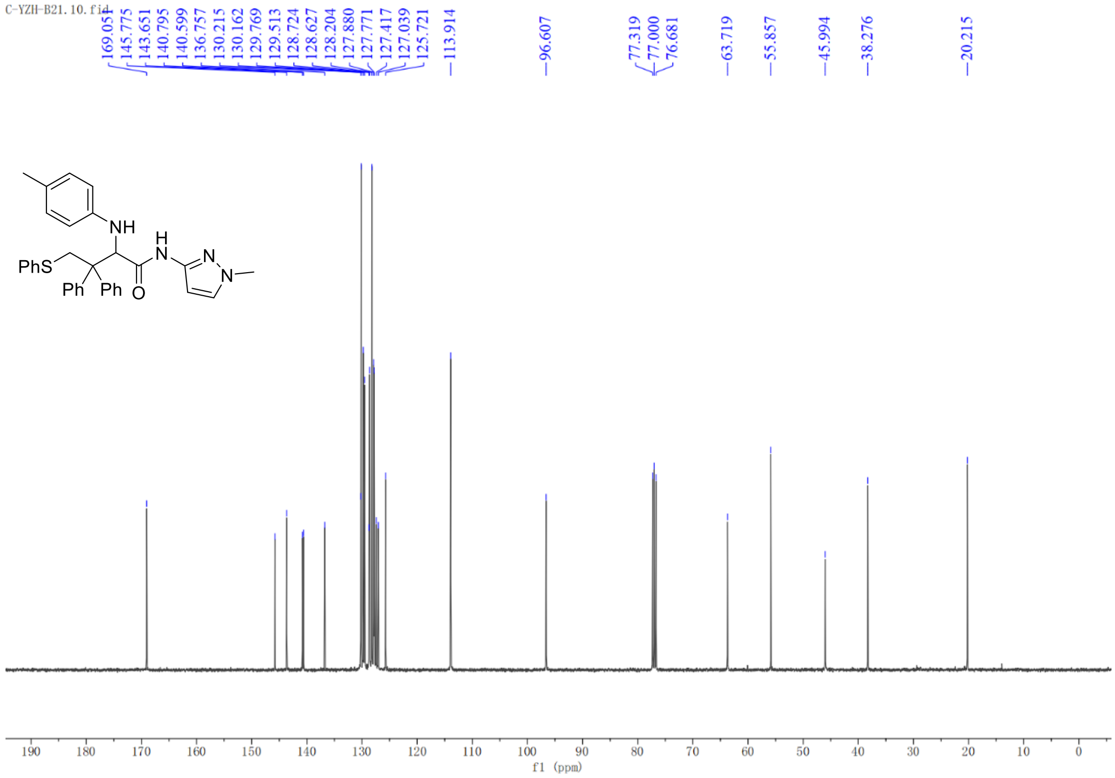

^{1}H NMR (400 MHz, $CDCl_3$) spectrum of **49**

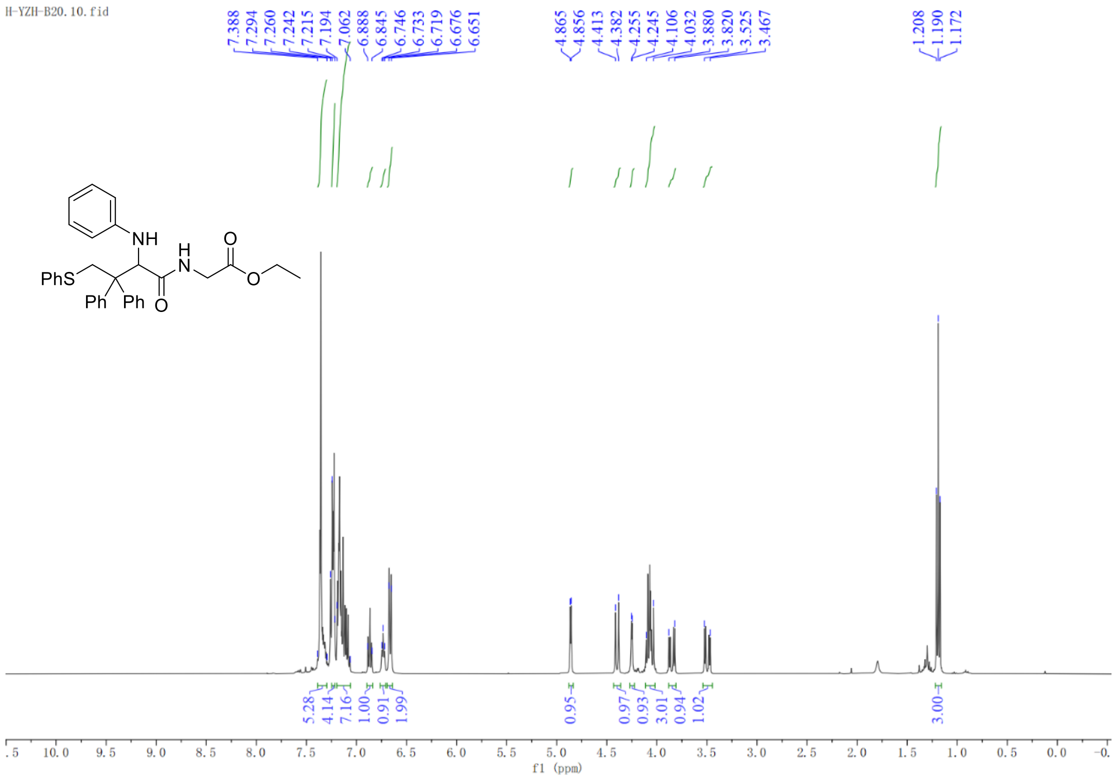

^{13}C NMR (101 MHz, $CDCl_3$) spectrum of **49**

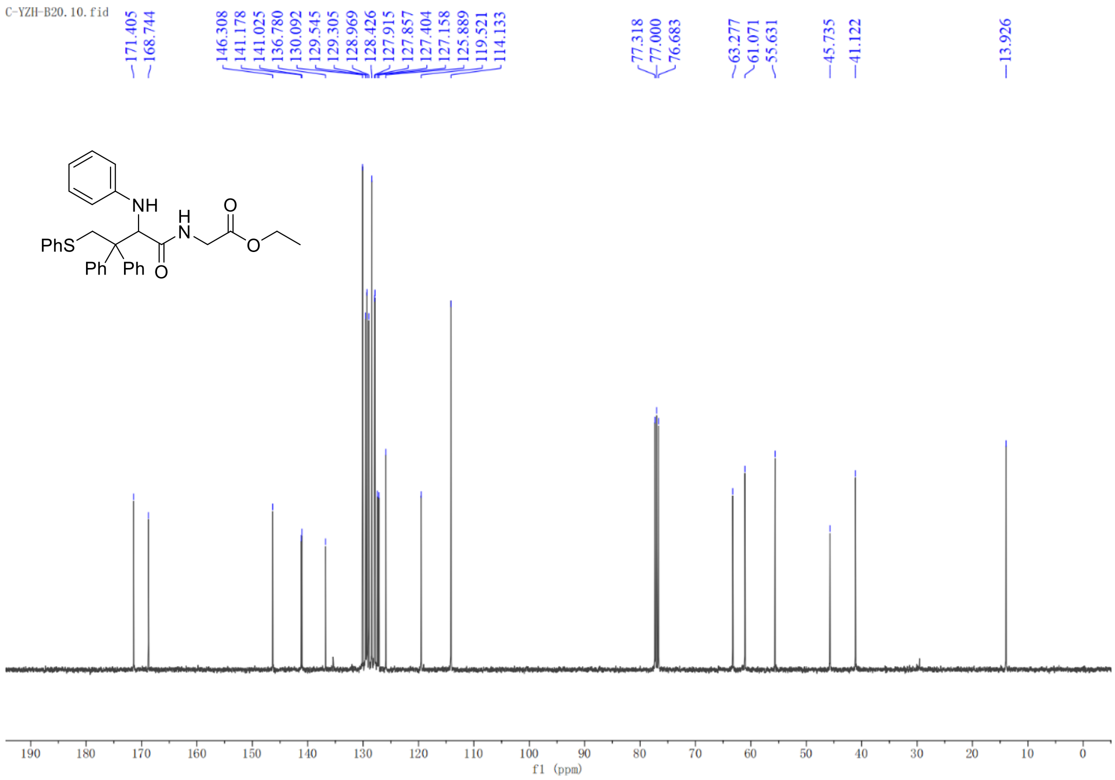

^{1}H NMR (400 MHz, $CDCl_3$) spectrum of **50**

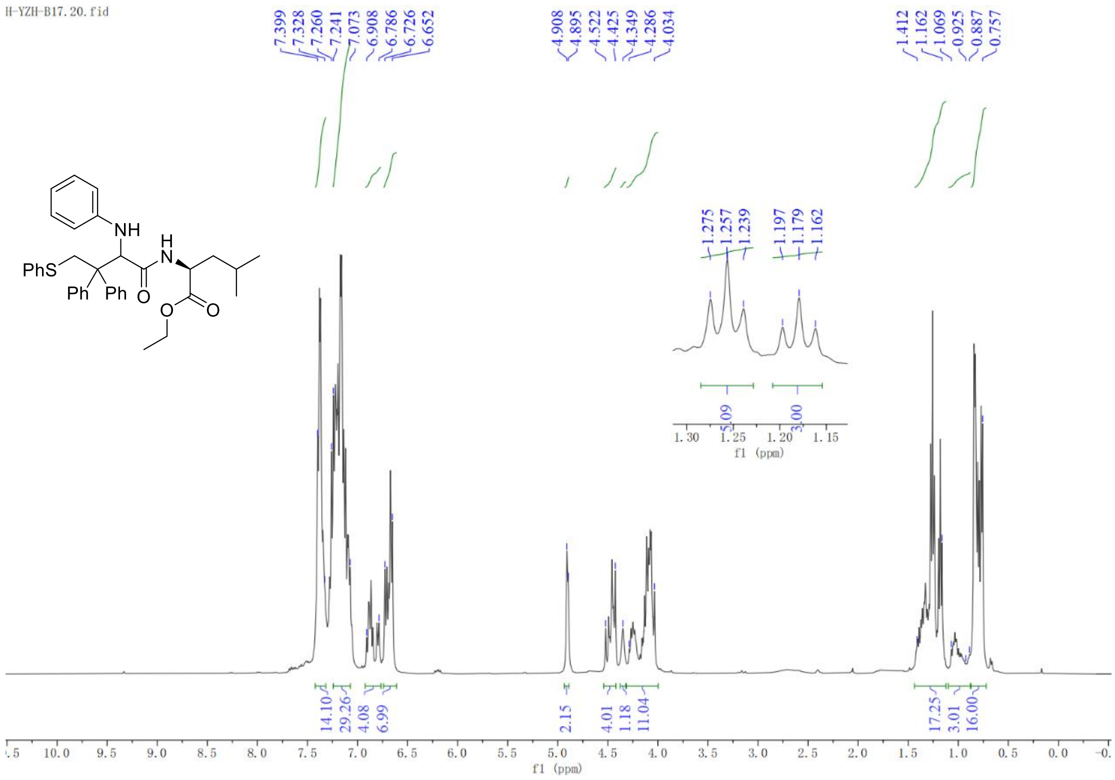

^{13}C NMR (101 MHz, $CDCl_3$) spectrum of **50**

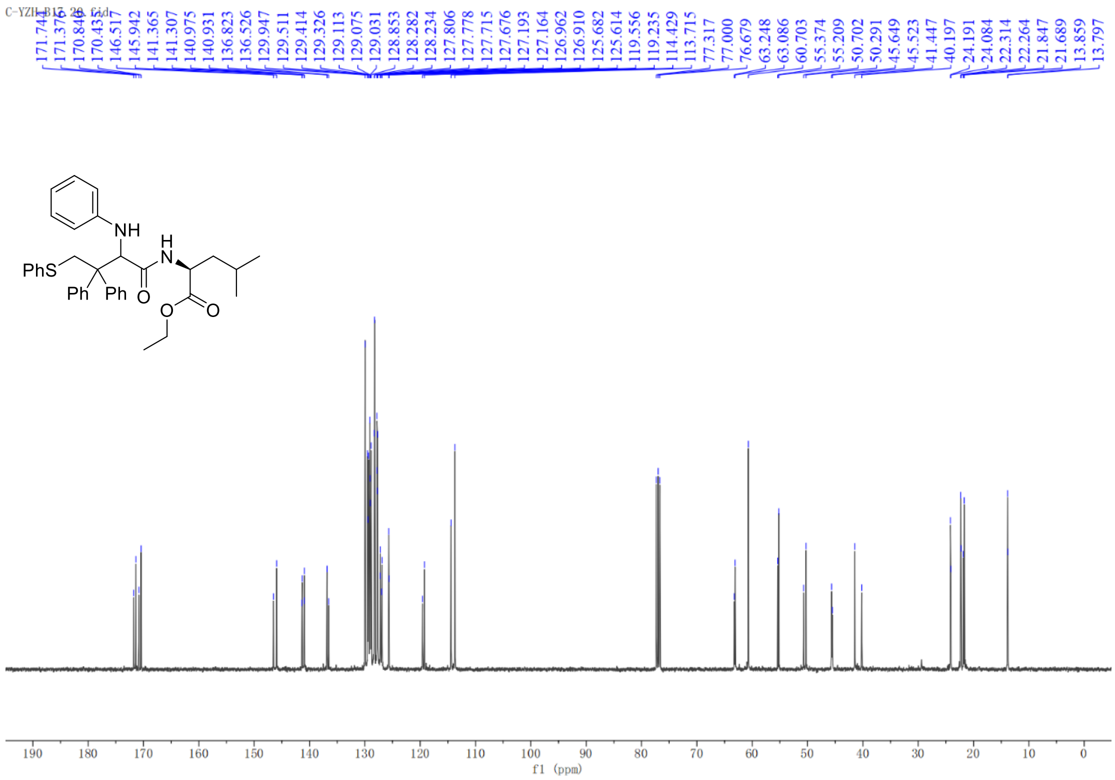

^{1}H NMR (400 MHz, $CDCl_3$) spectrum of **51**

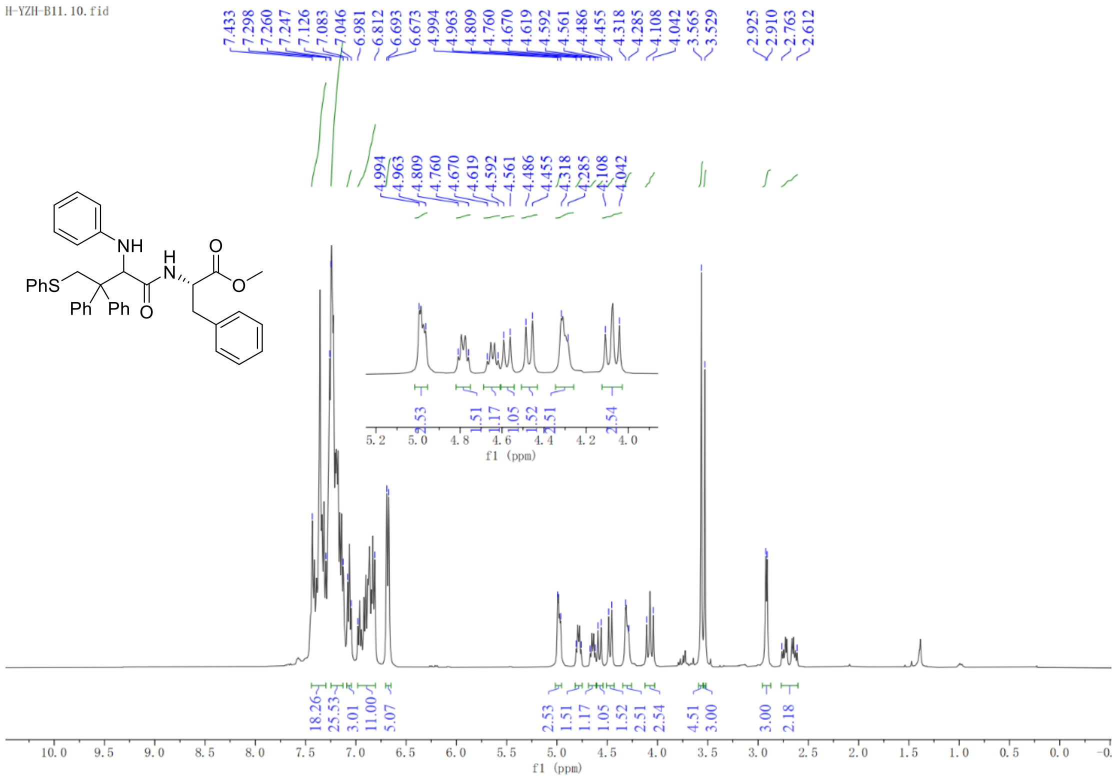

^{13}C NMR (101 MHz, $CDCl_3$) spectrum of **51**

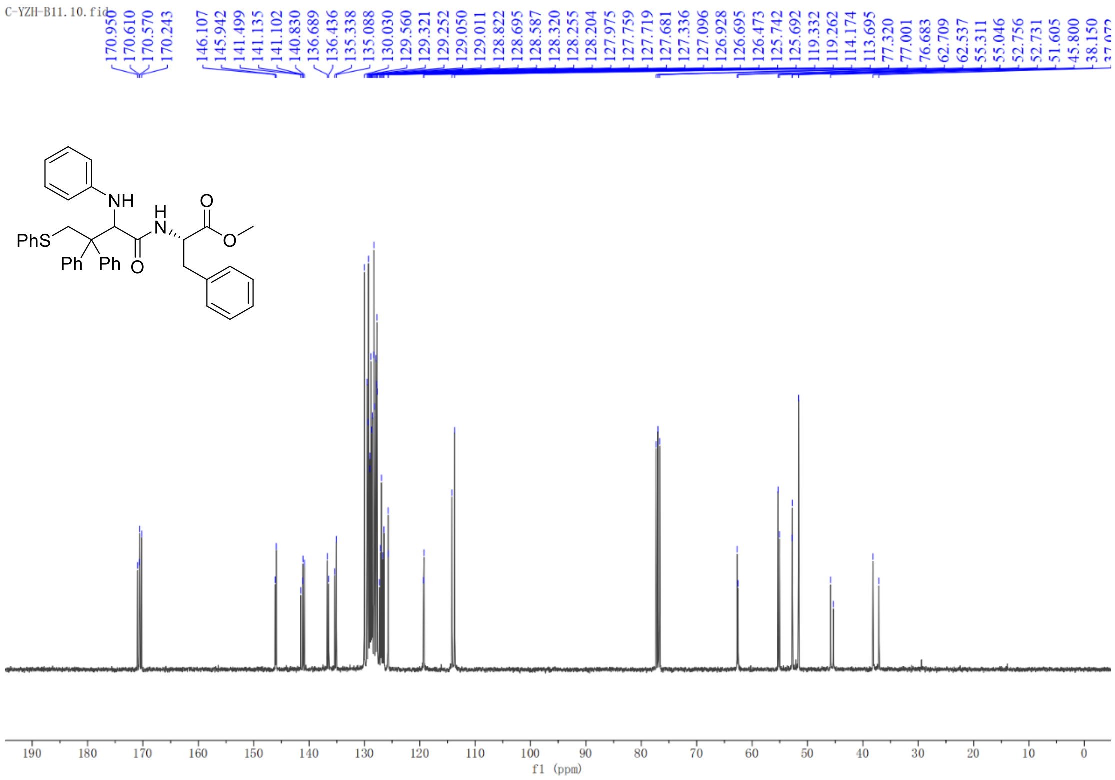

^{1}H NMR (400 MHz, $CDCl_3$) spectrum of **52**

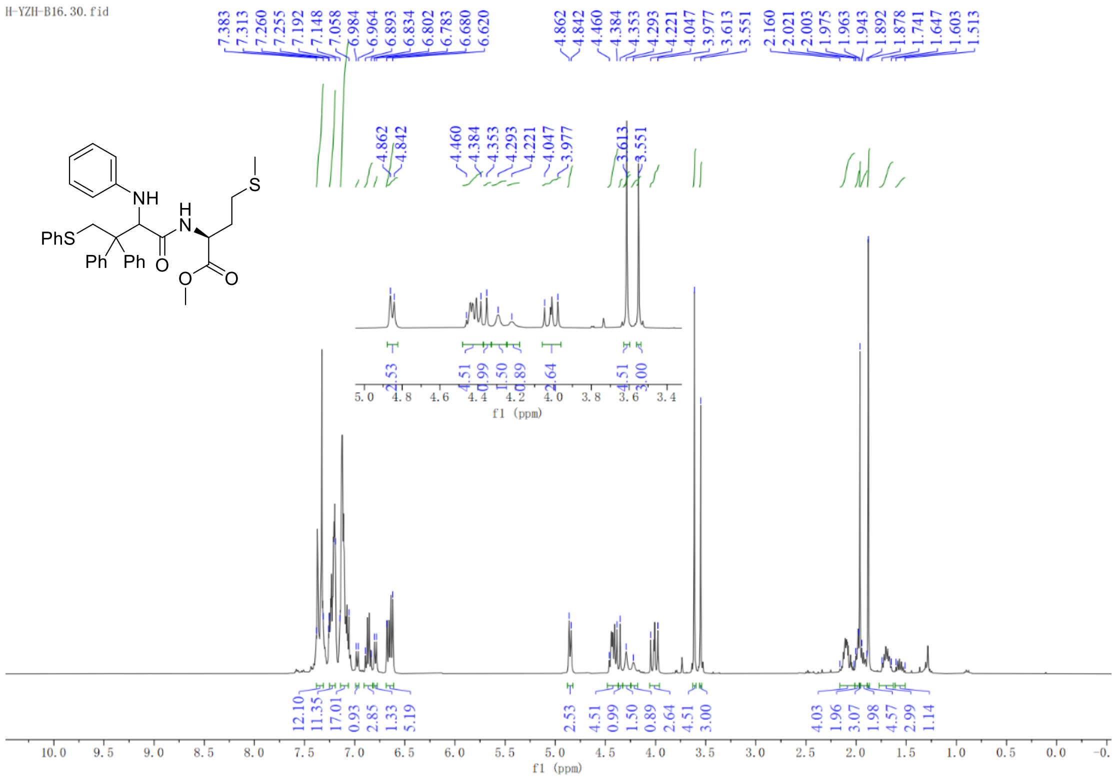

^{13}C NMR (101 MHz, $CDCl_3$) spectrum of **52**

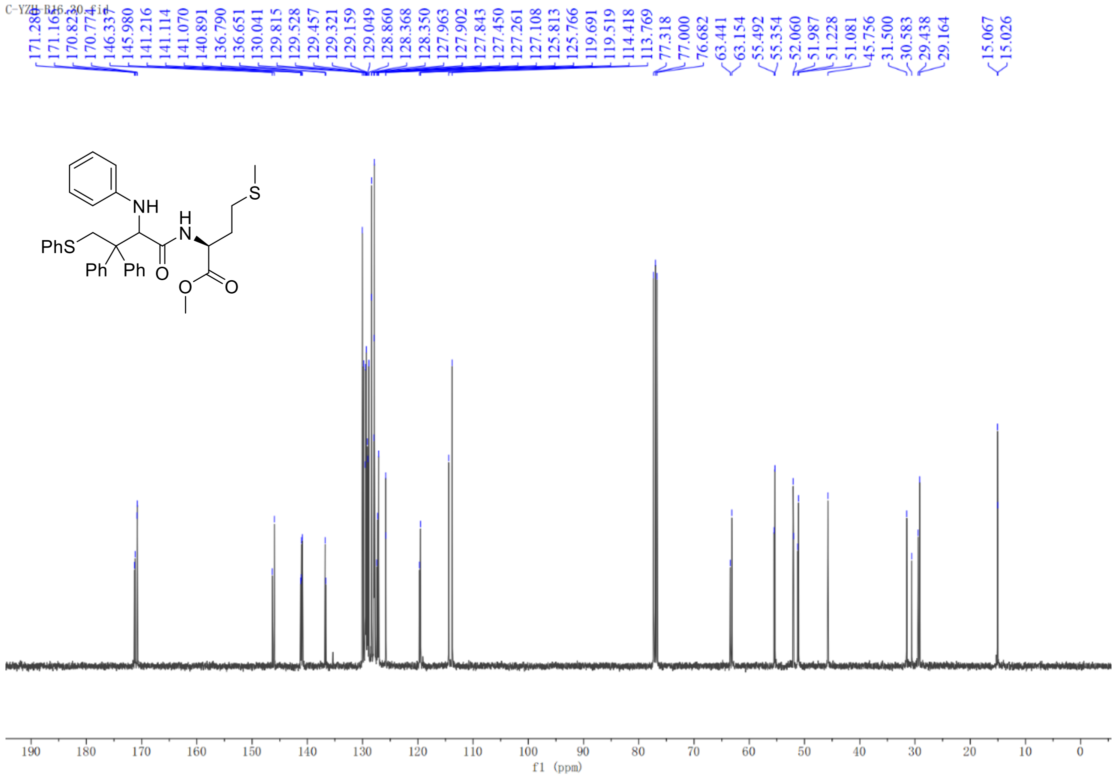

^{1}H NMR (400 MHz, $CDCl_3$) spectrum of **53**

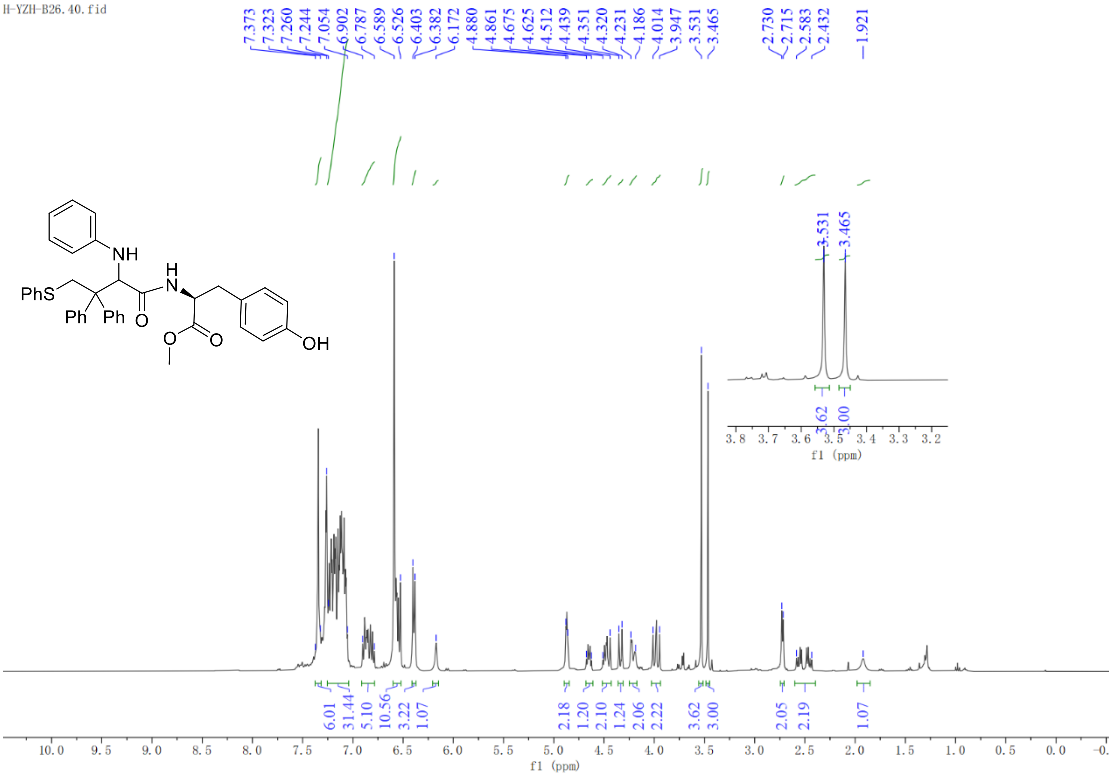

^{13}C NMR (101 MHz, $CDCl_3$) spectrum of **53**

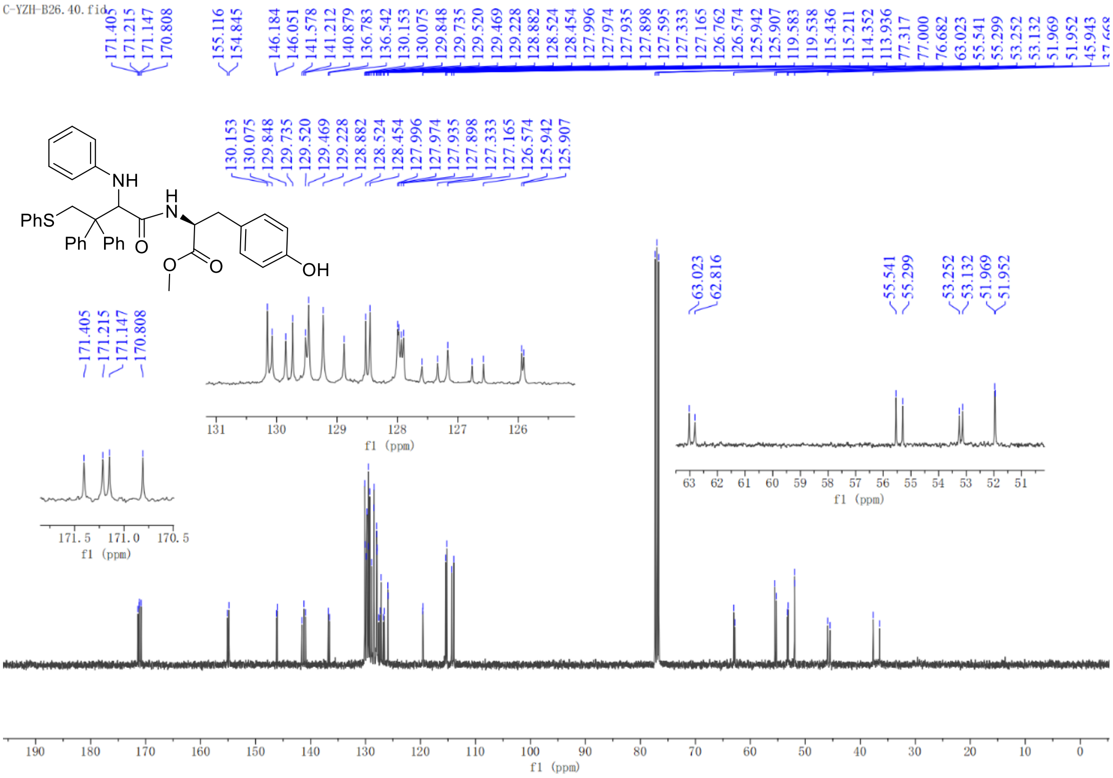

^{1}H NMR (400 MHz, $CDCl_3$) spectrum of **54**

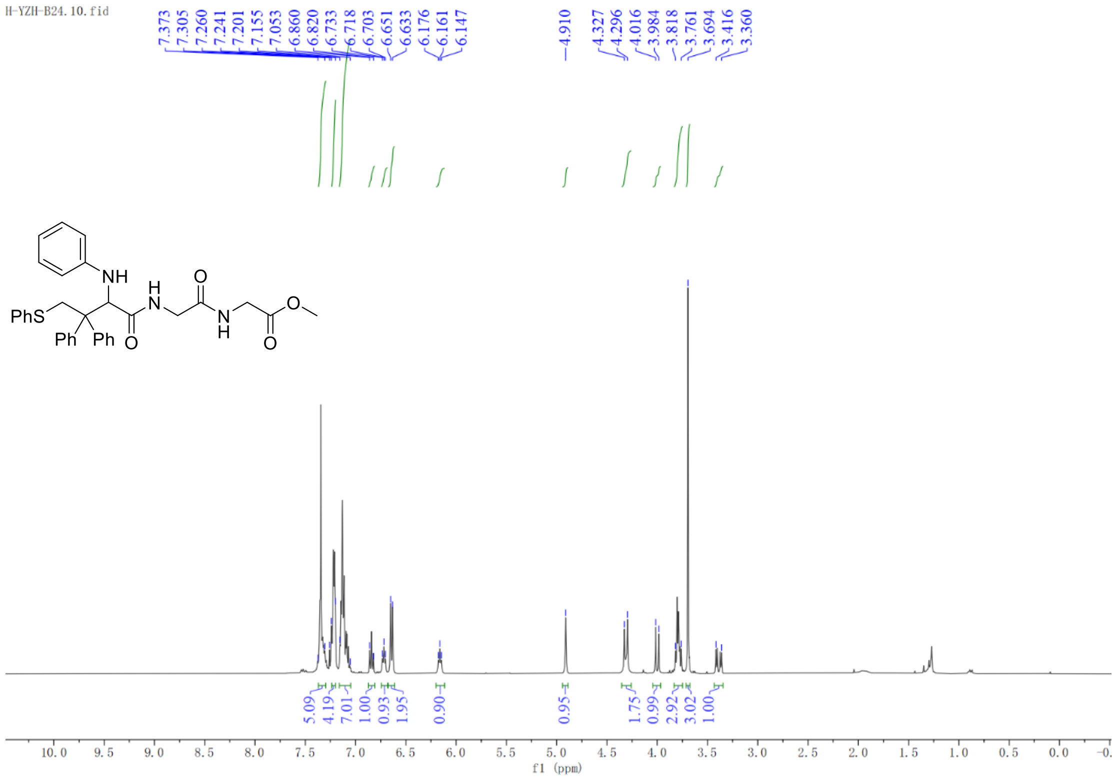

^{13}C NMR (101 MHz, $CDCl_3$) spectrum of **54**

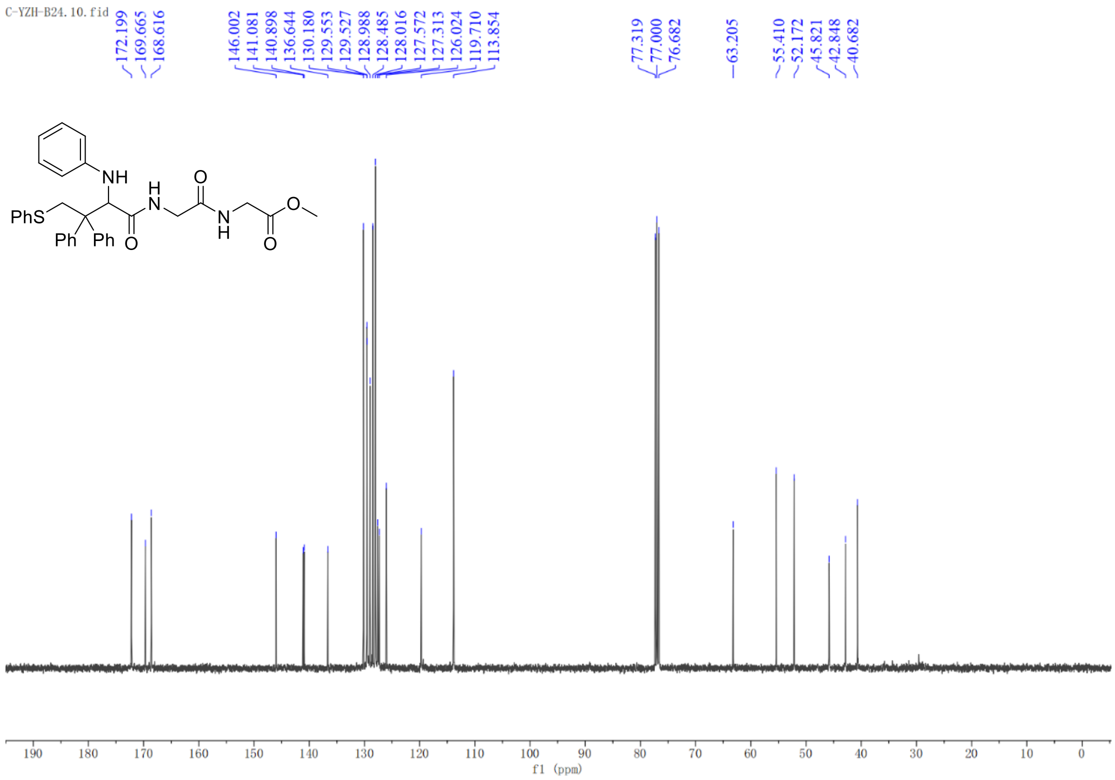

^{1}H NMR (400 MHz, $CDCl_3$) spectrum of **55**

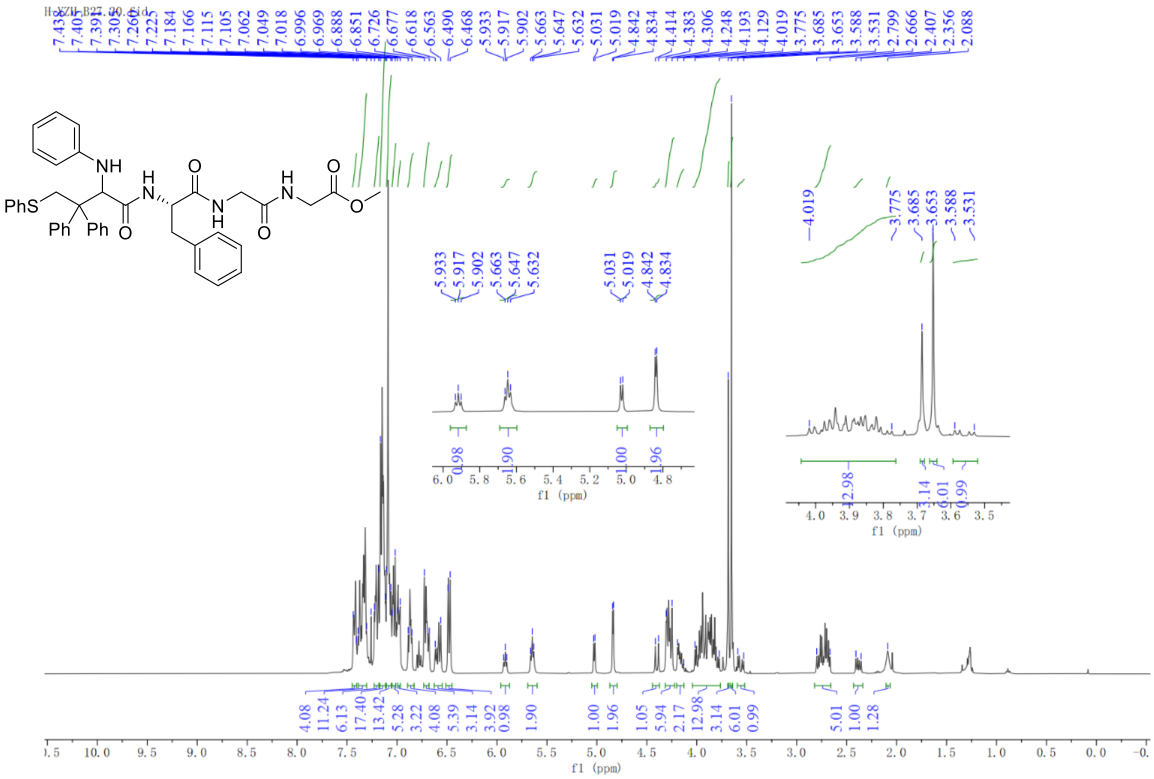

^{13}C NMR (101 MHz, $CDCl_3$) spectrum of **55**

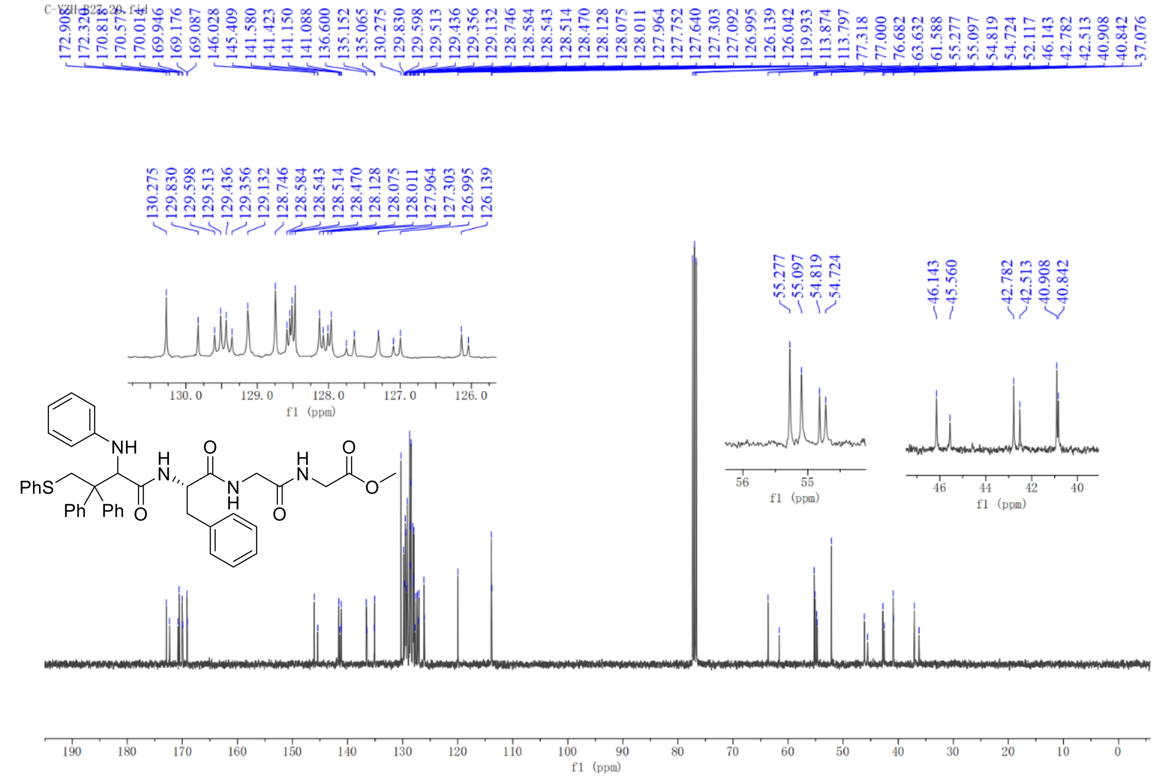

^{1}H NMR (400 MHz, $CDCl_3$) spectrum of **56**

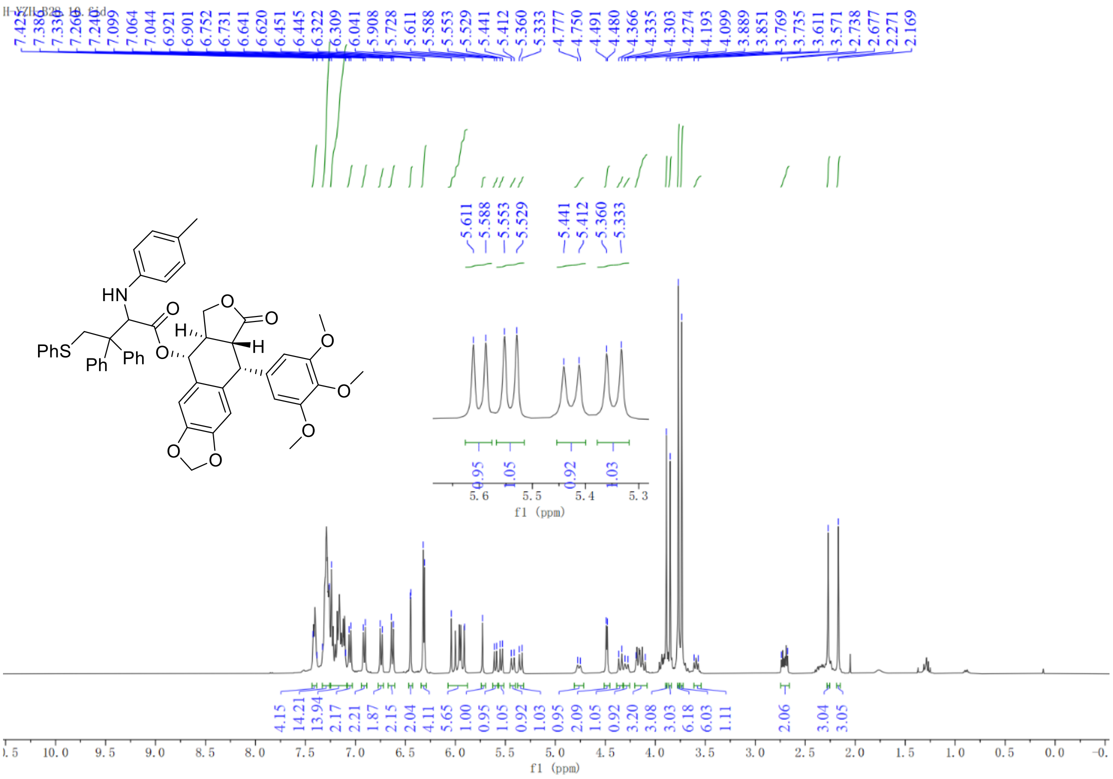

^{13}C NMR (101 MHz, $CDCl_3$) spectrum of **56**

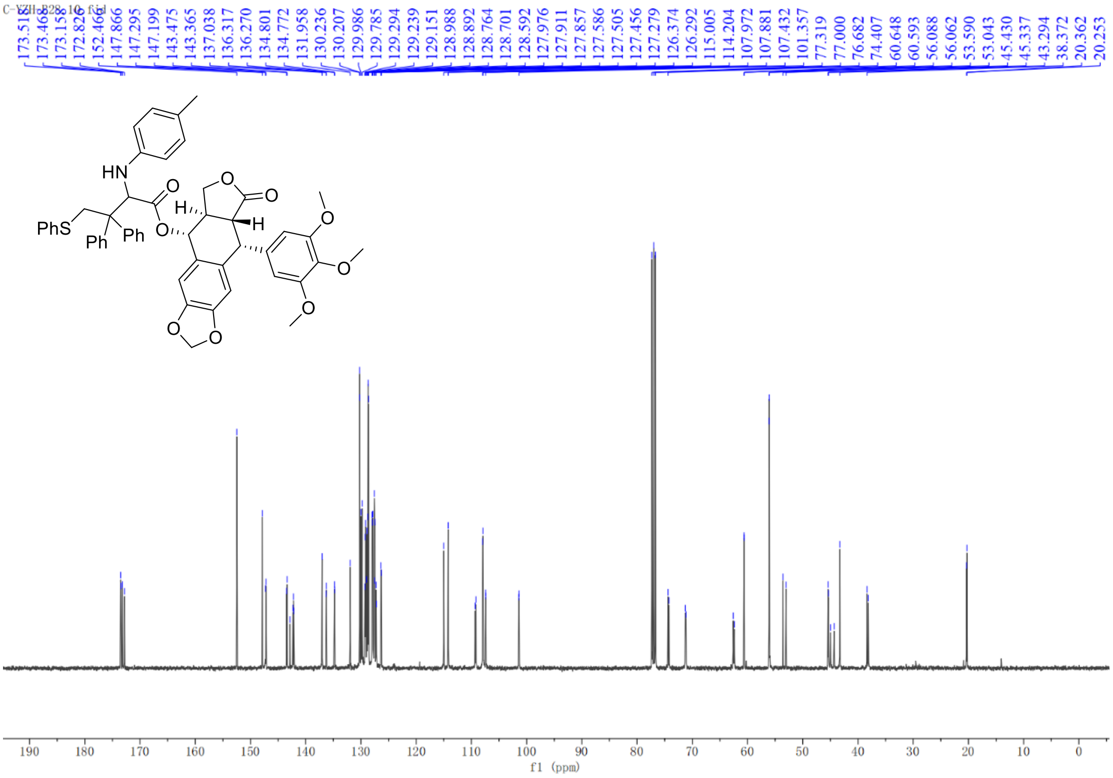

^{1}H NMR (400 MHz, $CDCl_3$) spectrum of **57**

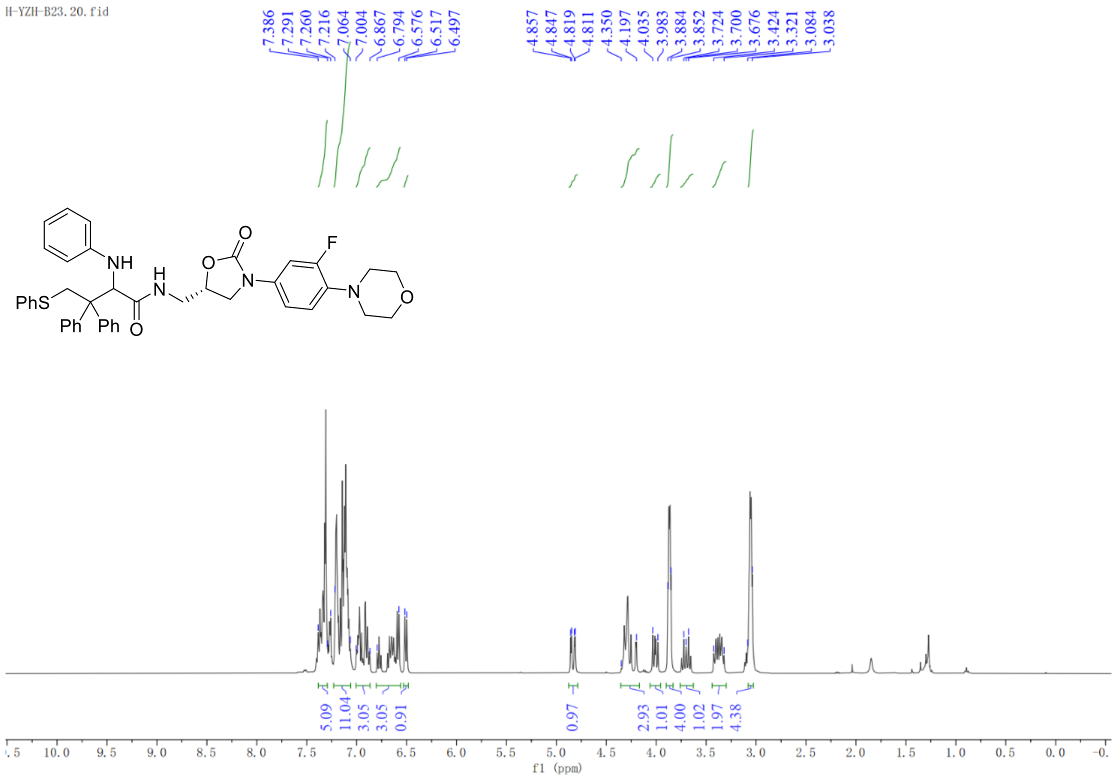

^{13}C NMR (101 MHz, $CDCl_3$) spectrum of **57**

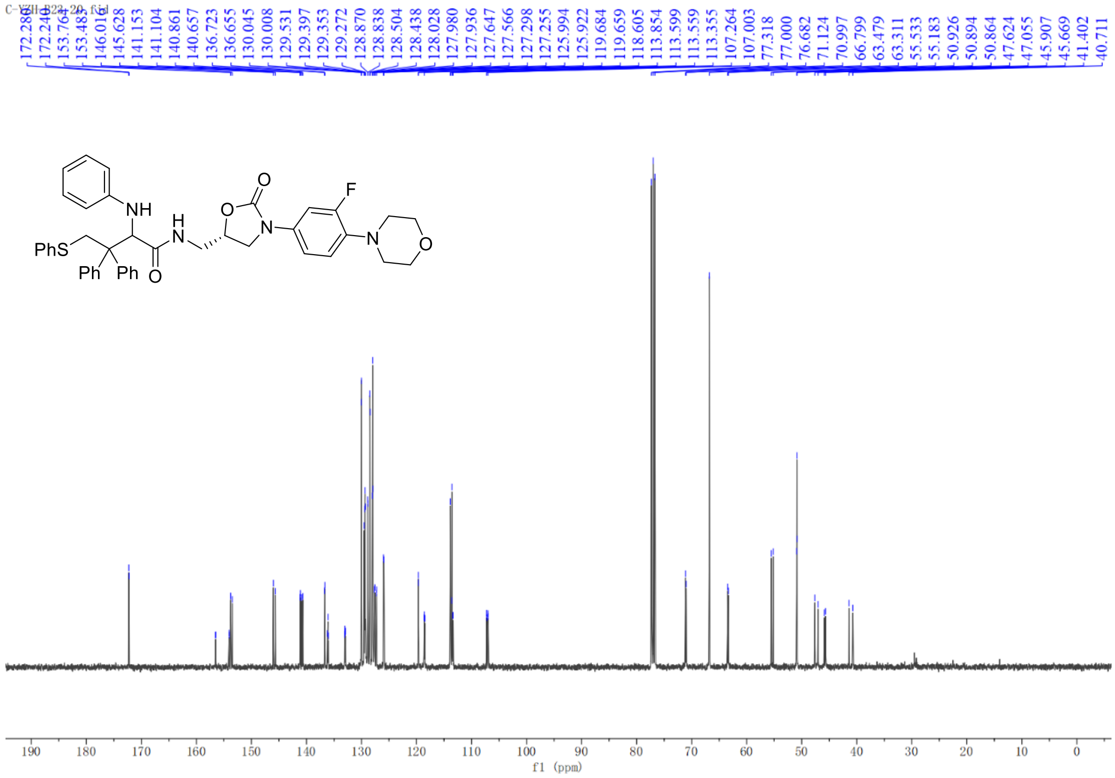

^{19}F NMR (376 MHz, $CDCl_3$) spectrum of **57**

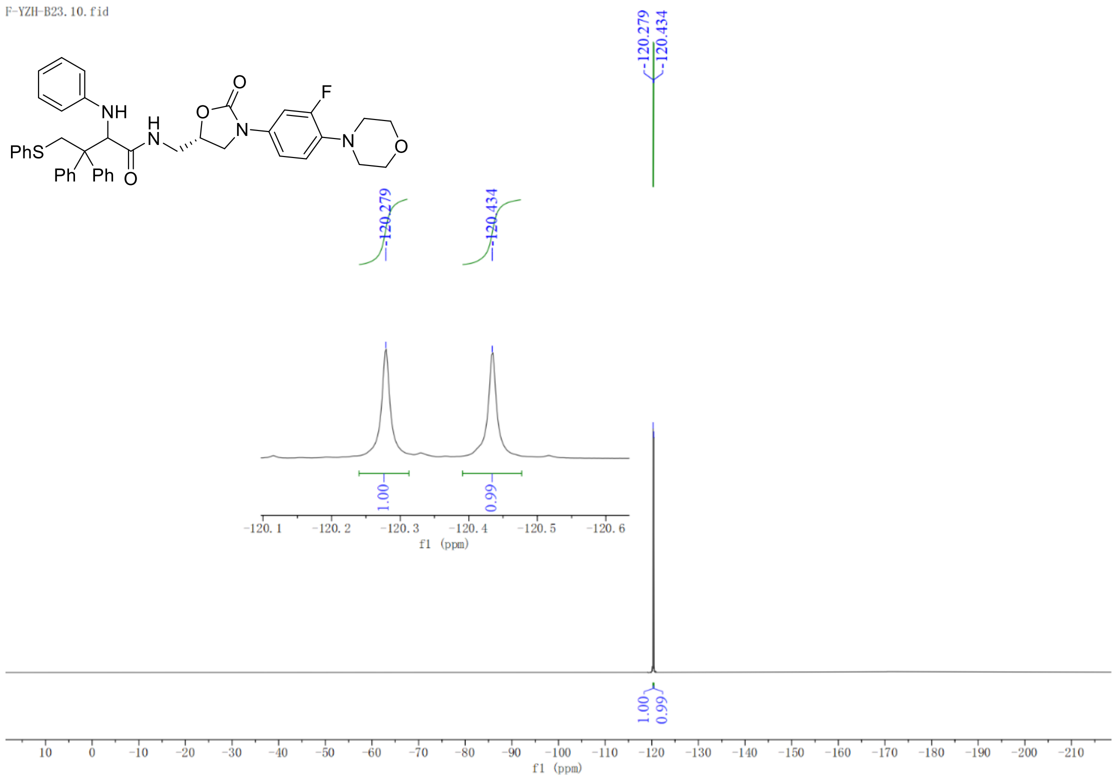

^{1}H NMR (400 MHz, $CDCl_3$) spectrum of **58**

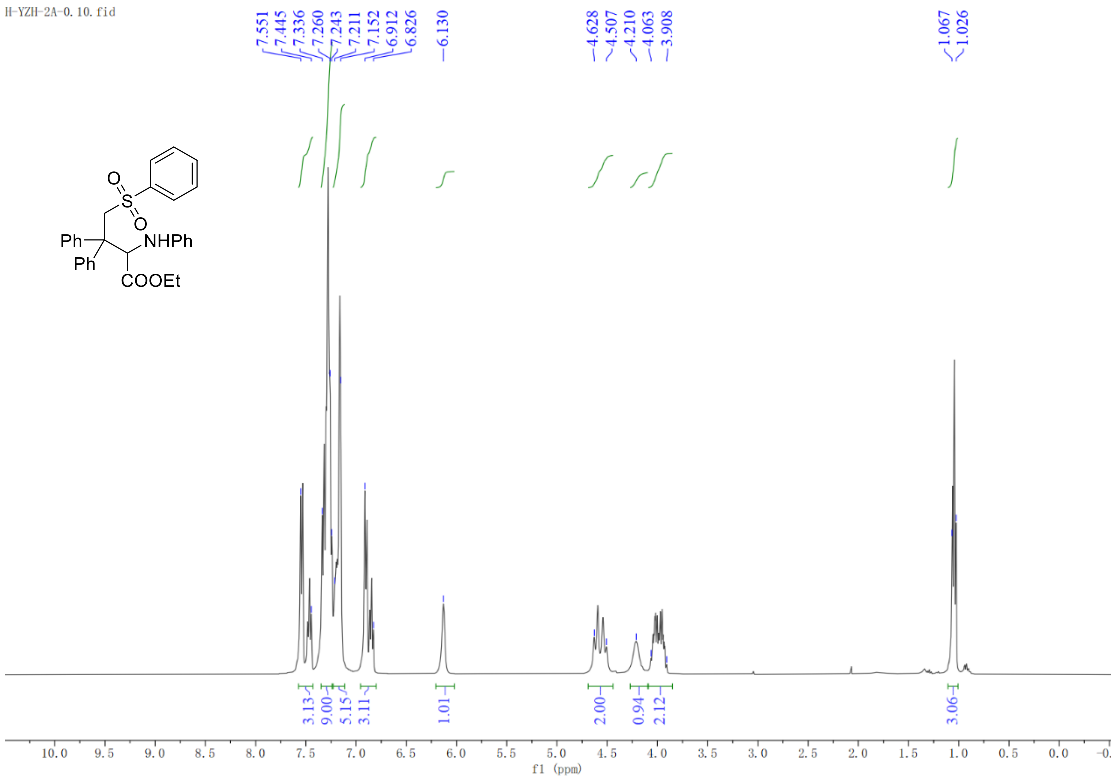

^{13}C NMR (101 MHz, CDCl$_3$) spectrum of **58**

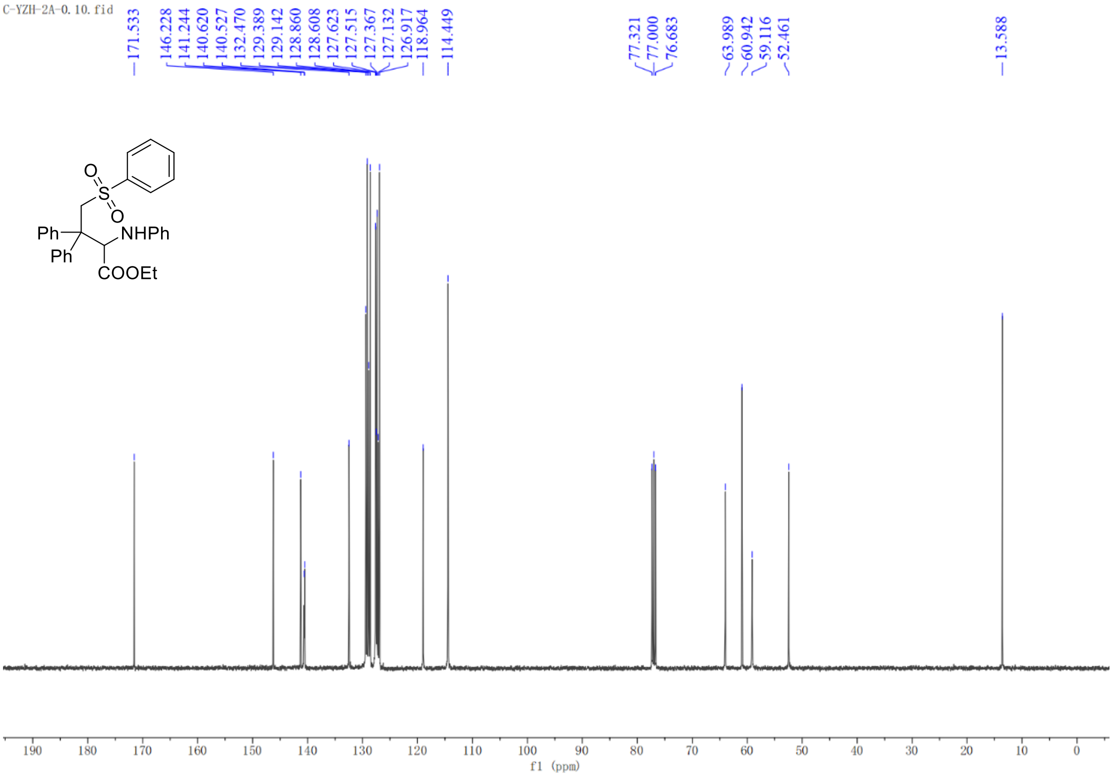

^{1}H NMR (400 MHz, CDCl$_3$) spectrum of **59**

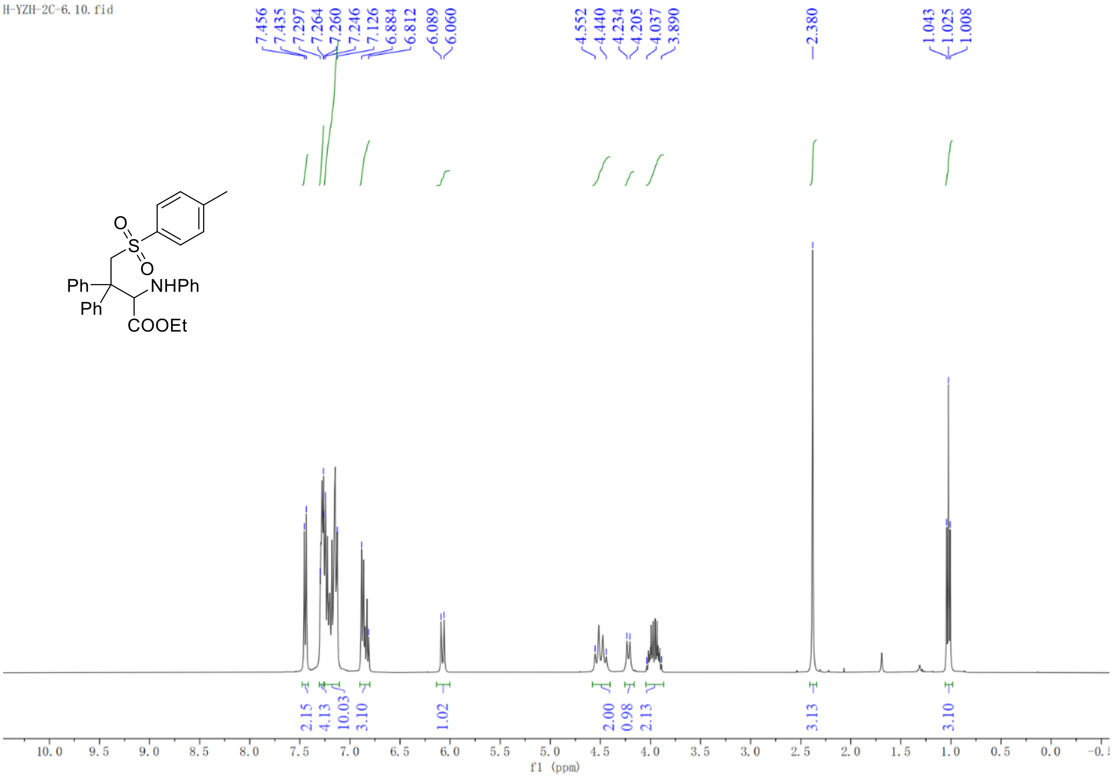

^{13}C NMR (101 MHz, $CDCl_3$) spectrum of **59**

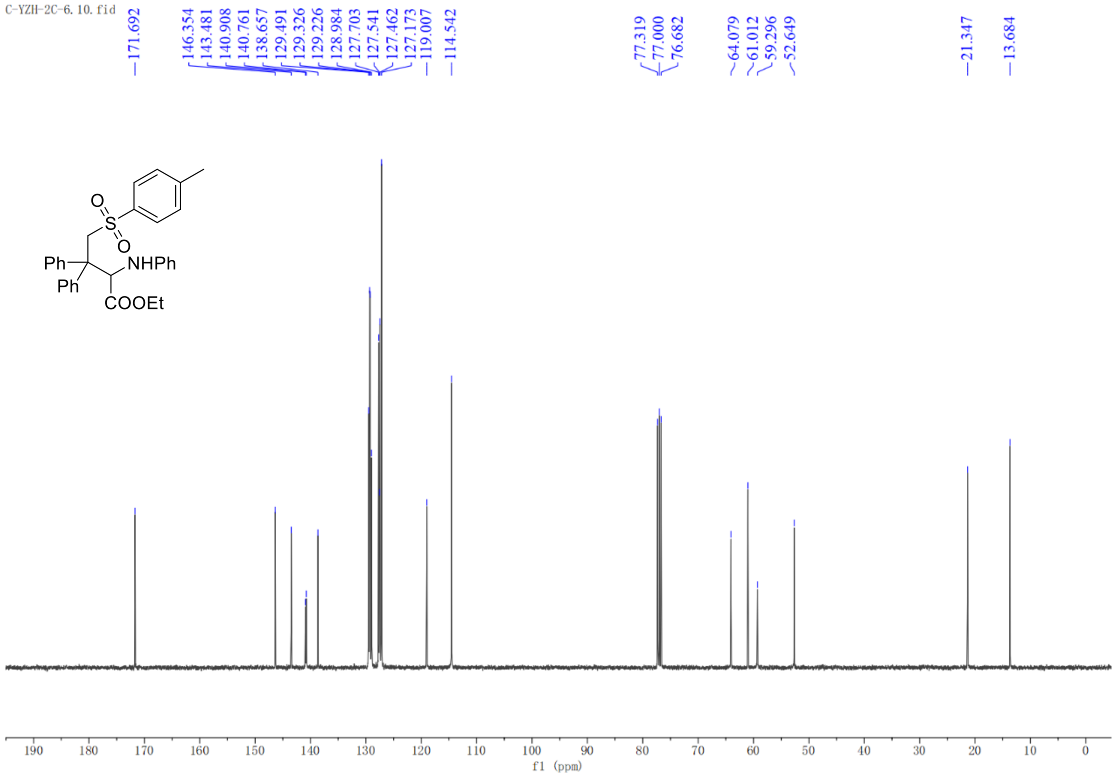

^{1}H NMR (400 MHz, $CDCl_3$) spectrum of **60**

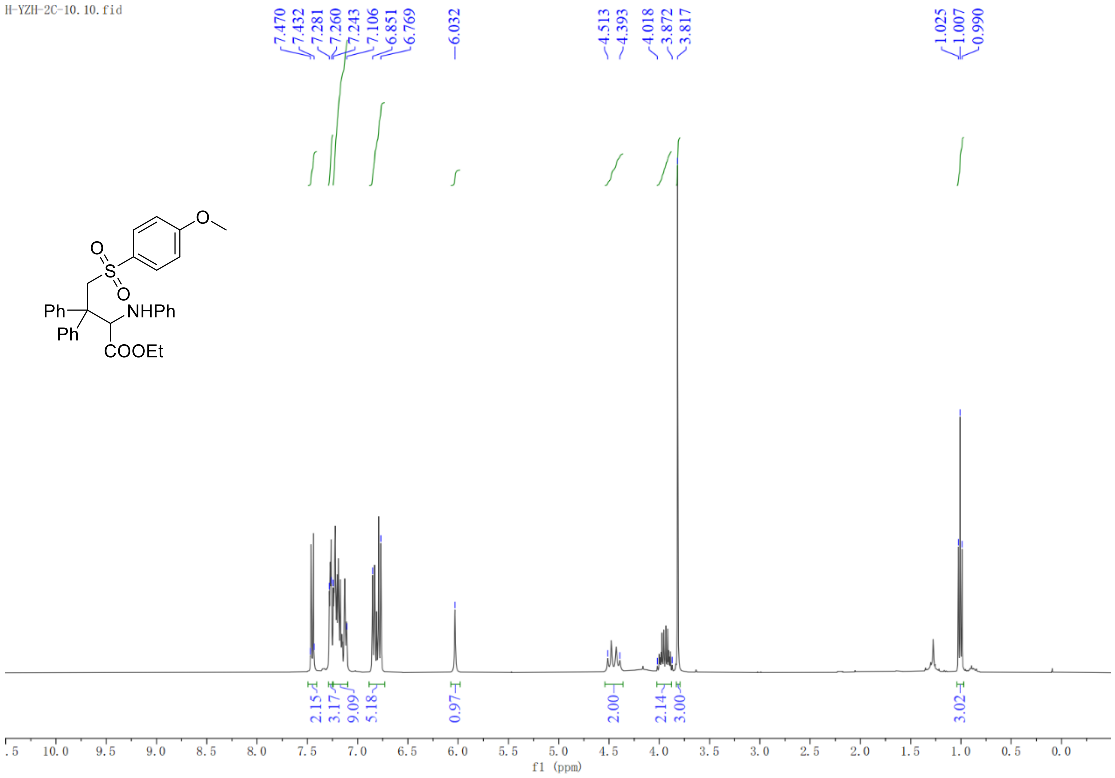

^{13}C NMR (101 MHz, $CDCl_3$) spectrum of **60**

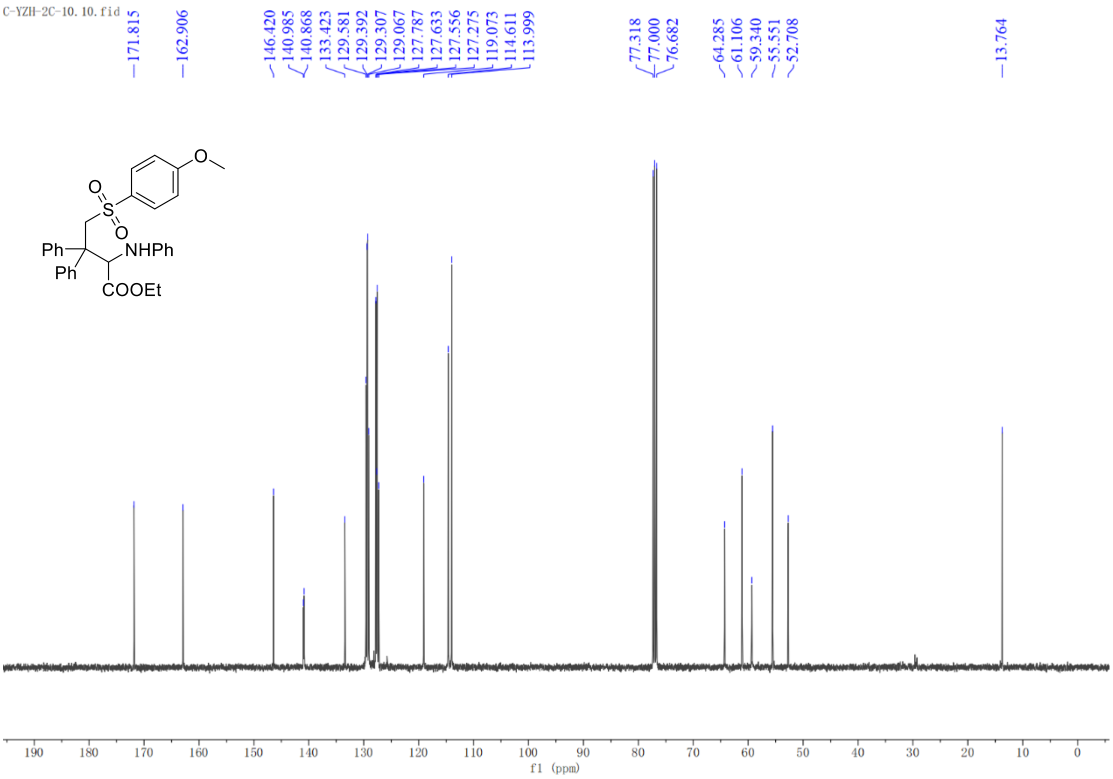

^{1}H NMR (400 MHz, $CDCl_3$) spectrum of **61**

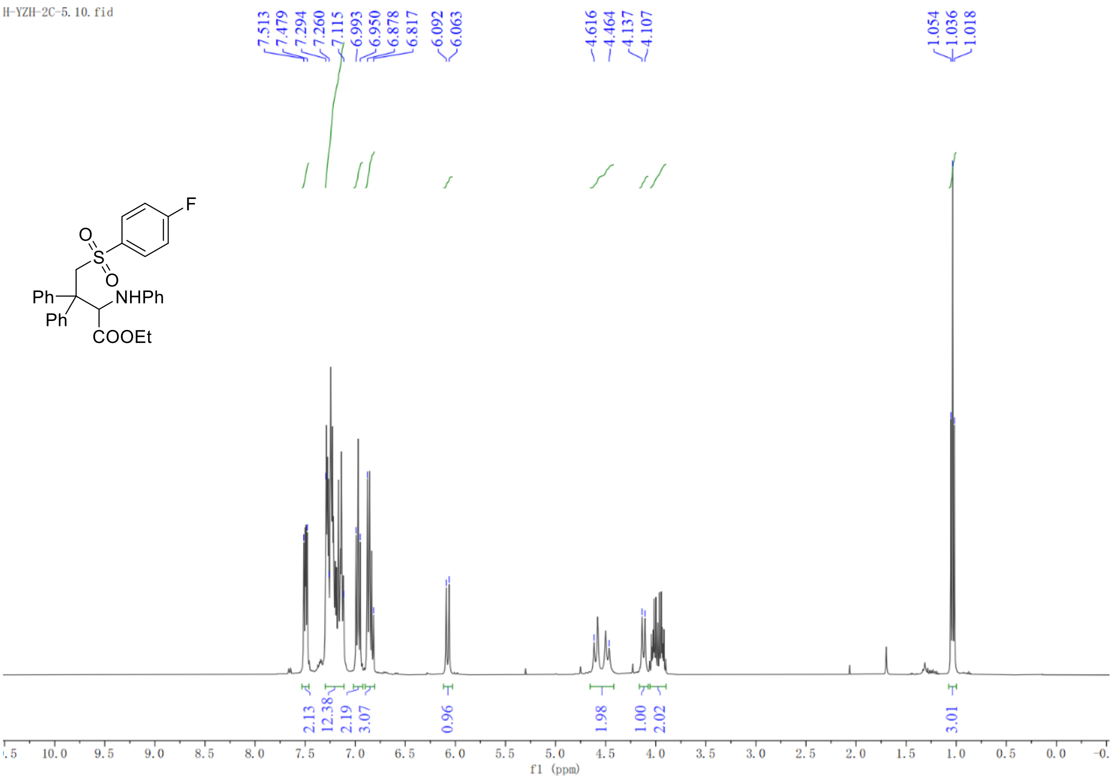

[13] **C NMR** (101 MHz, CDCl$_3$) spectrum of **61**

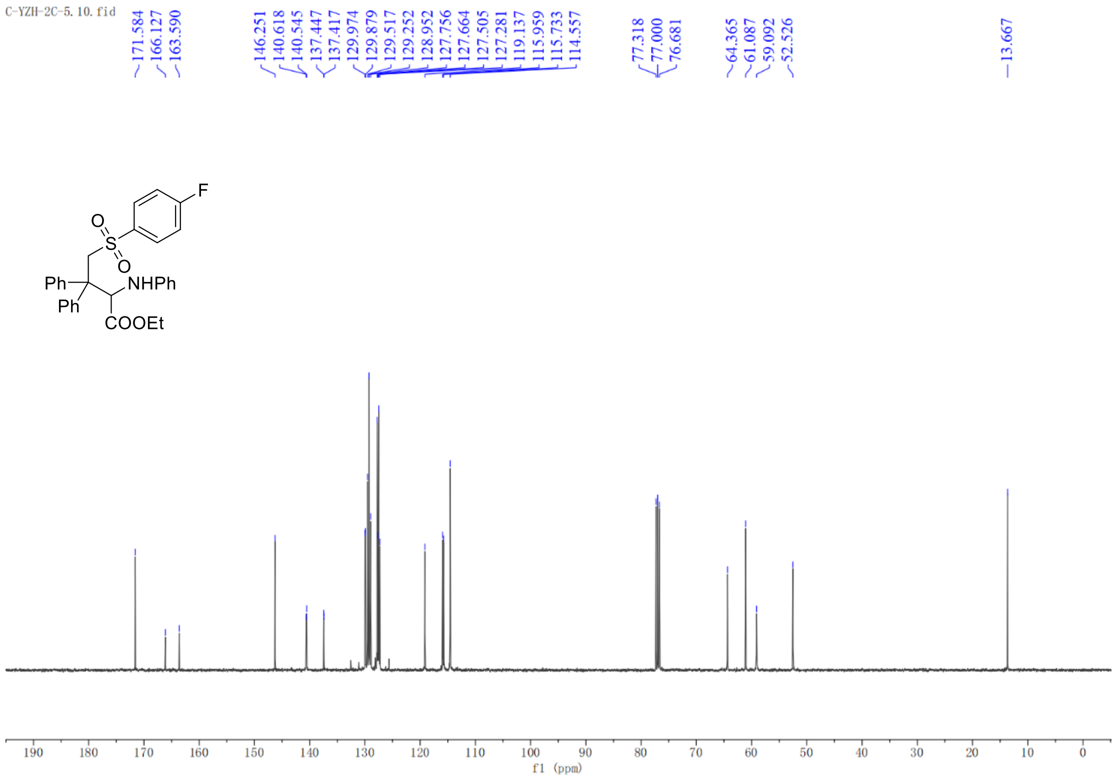

[19] **F NMR** (376 MHz, CDCl$_3$) spectrum of **61**

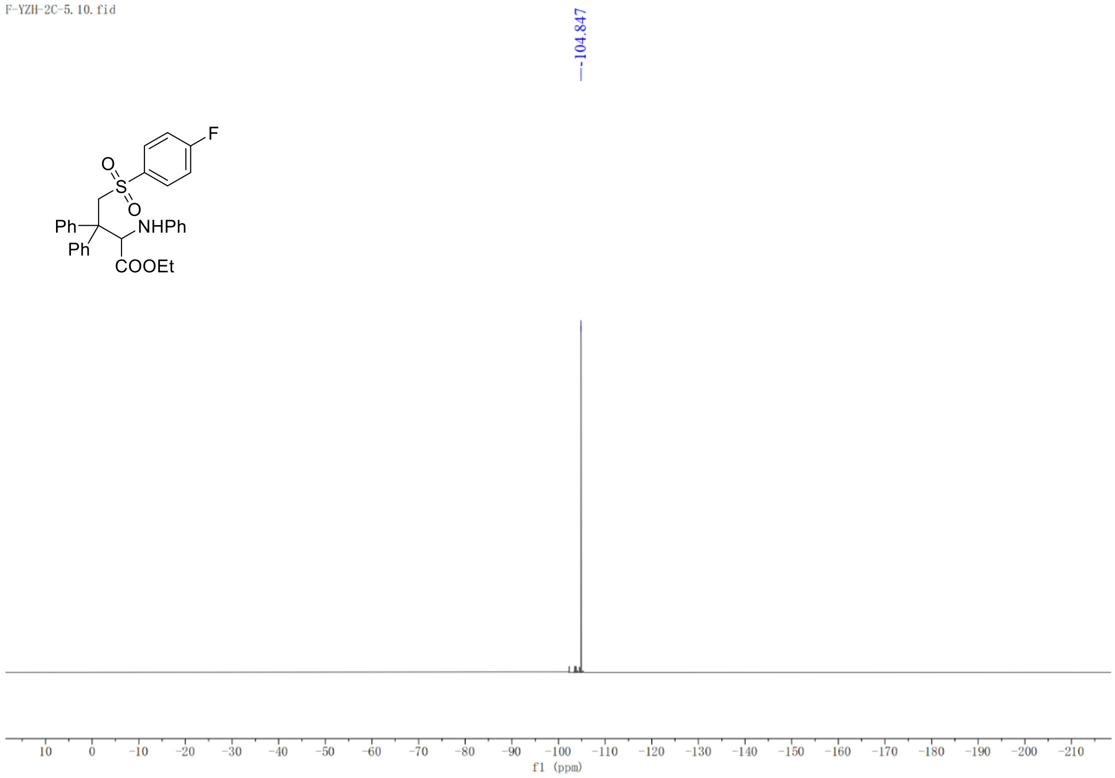

^{1}H NMR (400 MHz, $CDCl_3$) spectrum of **62**

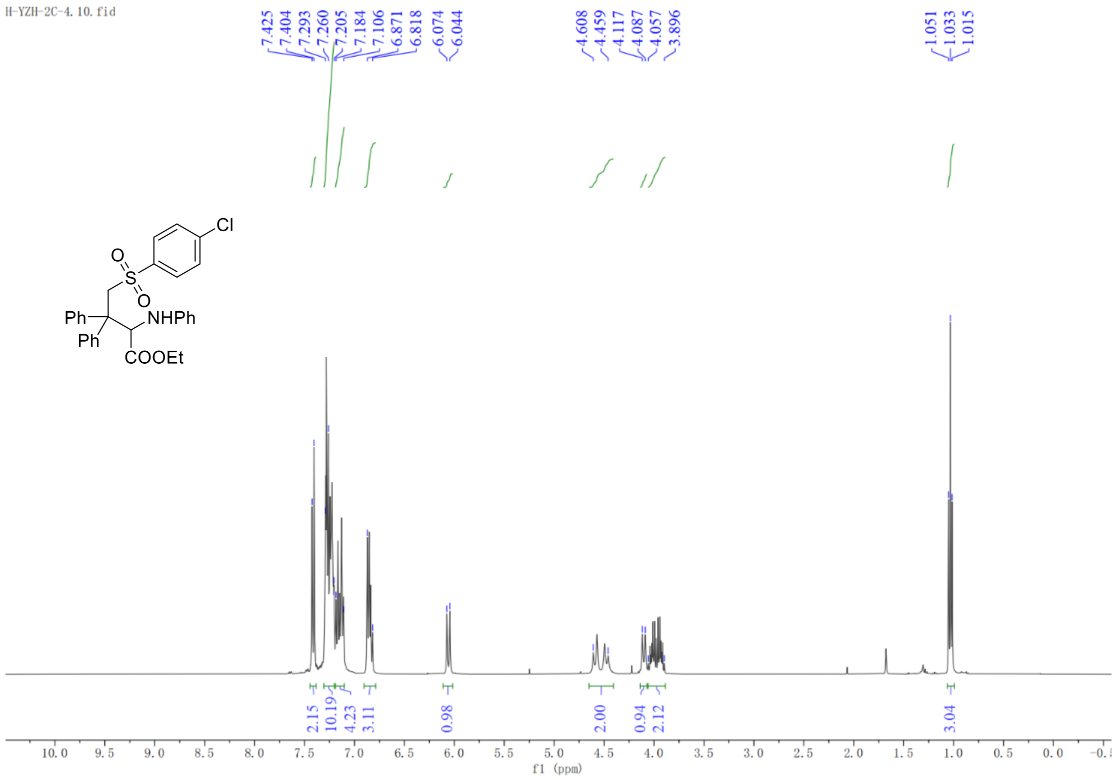

^{13}C NMR (101 MHz, $CDCl_3$) spectrum of **62**

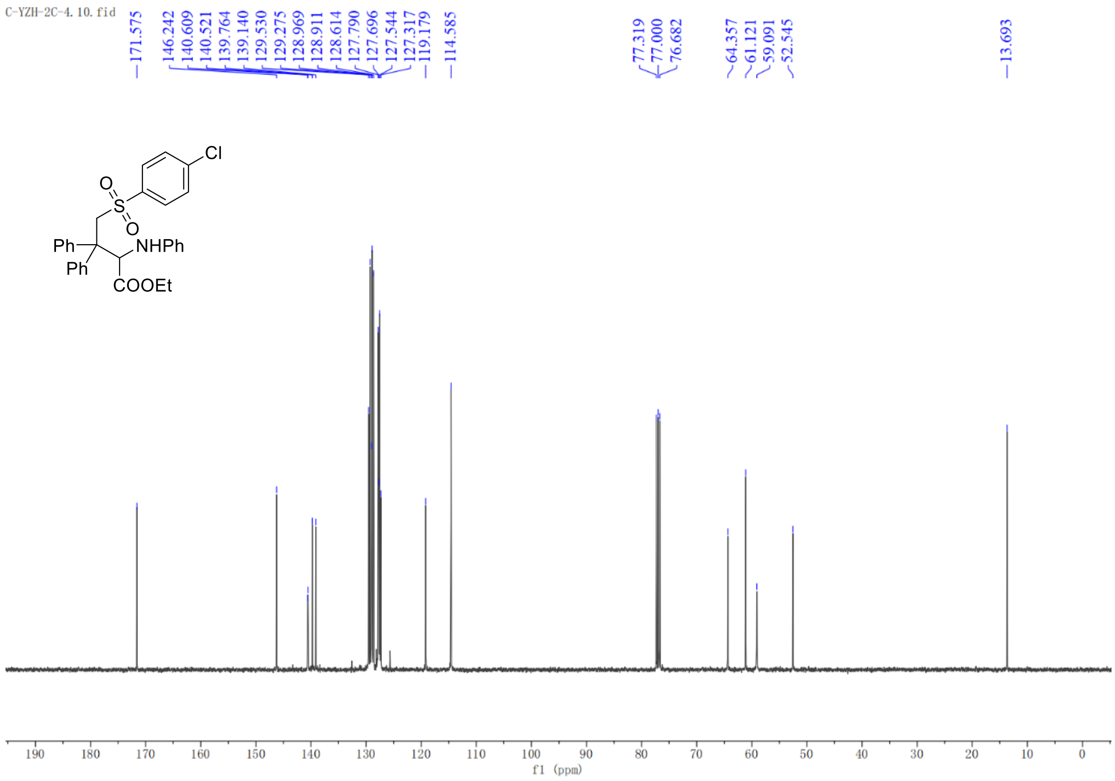

^{1}**H NMR** (400 MHz, $CDCl_3$) spectrum of **63**

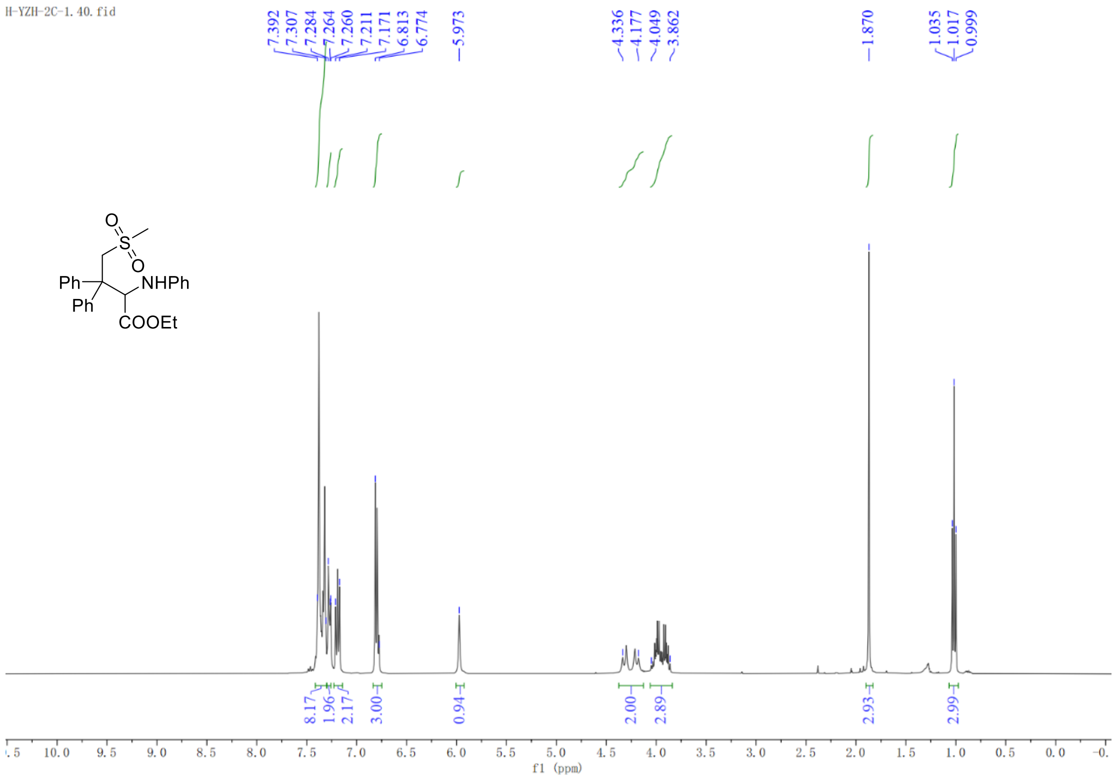

^{13}**C NMR** (101 MHz, $CDCl_3$) spectrum of **63**

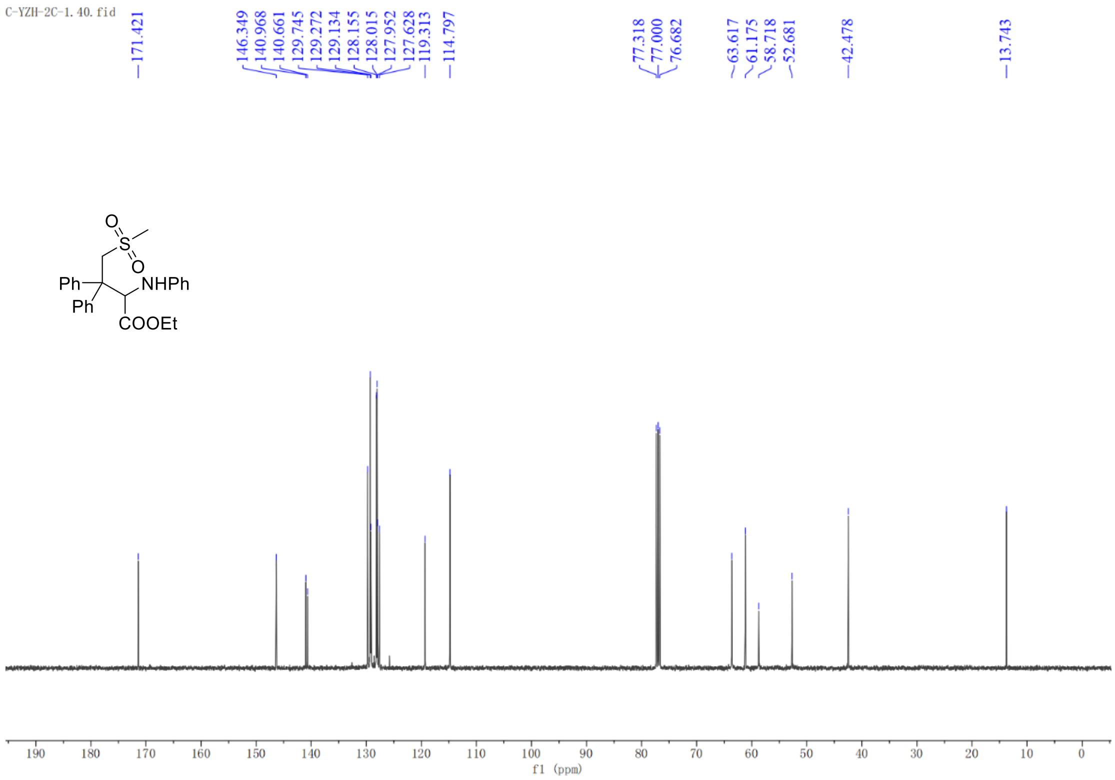

^{1}H NMR (400 MHz, $CDCl_3$) spectrum of **64**

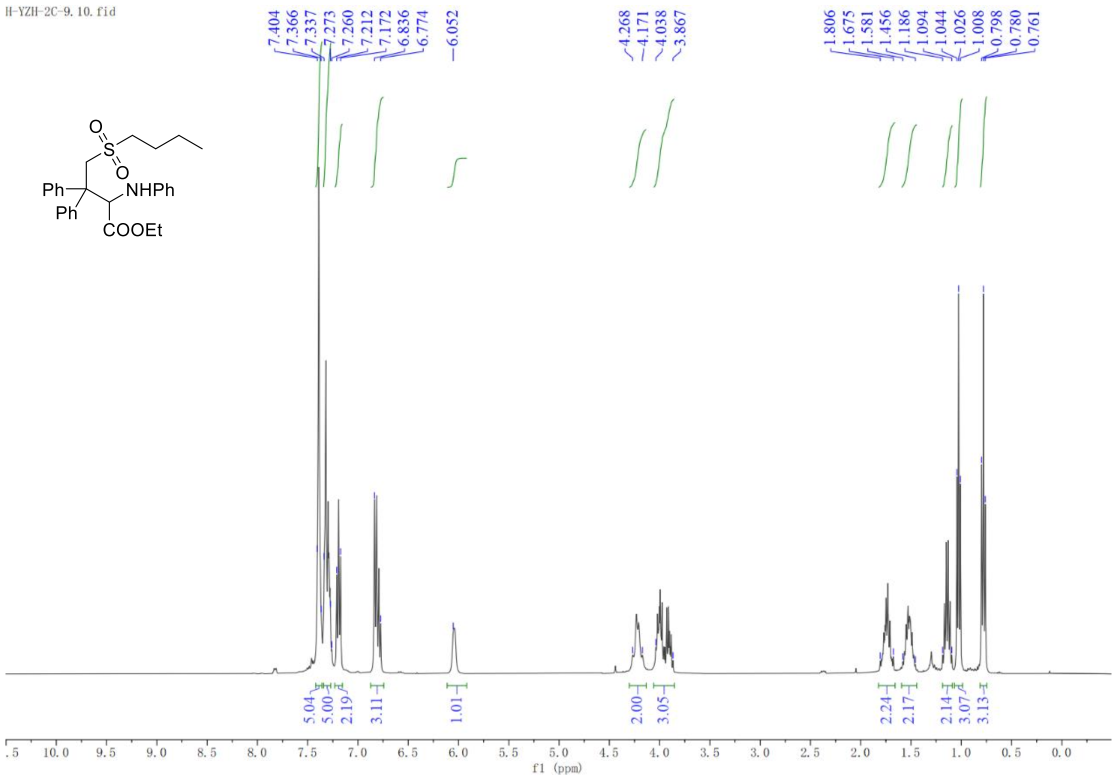

^{13}C NMR (101 MHz, $CDCl_3$) spectrum of **64**

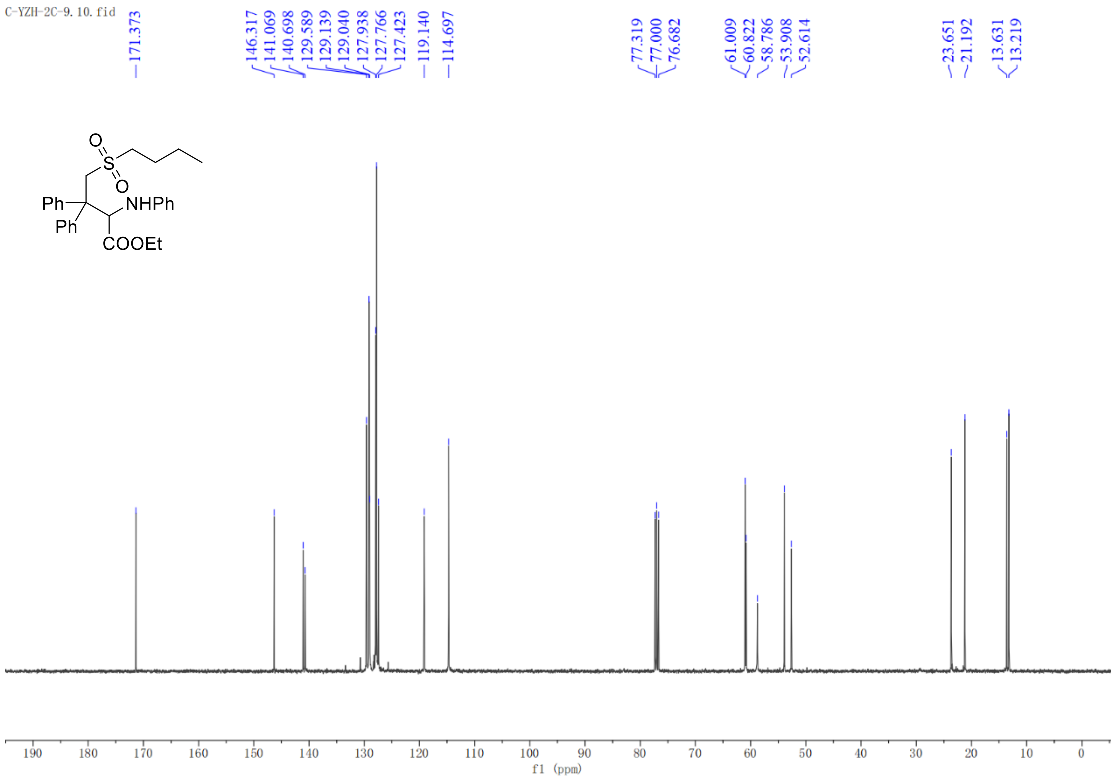

^{1}H NMR (400 MHz, $CDCl_3$) spectrum of **65**

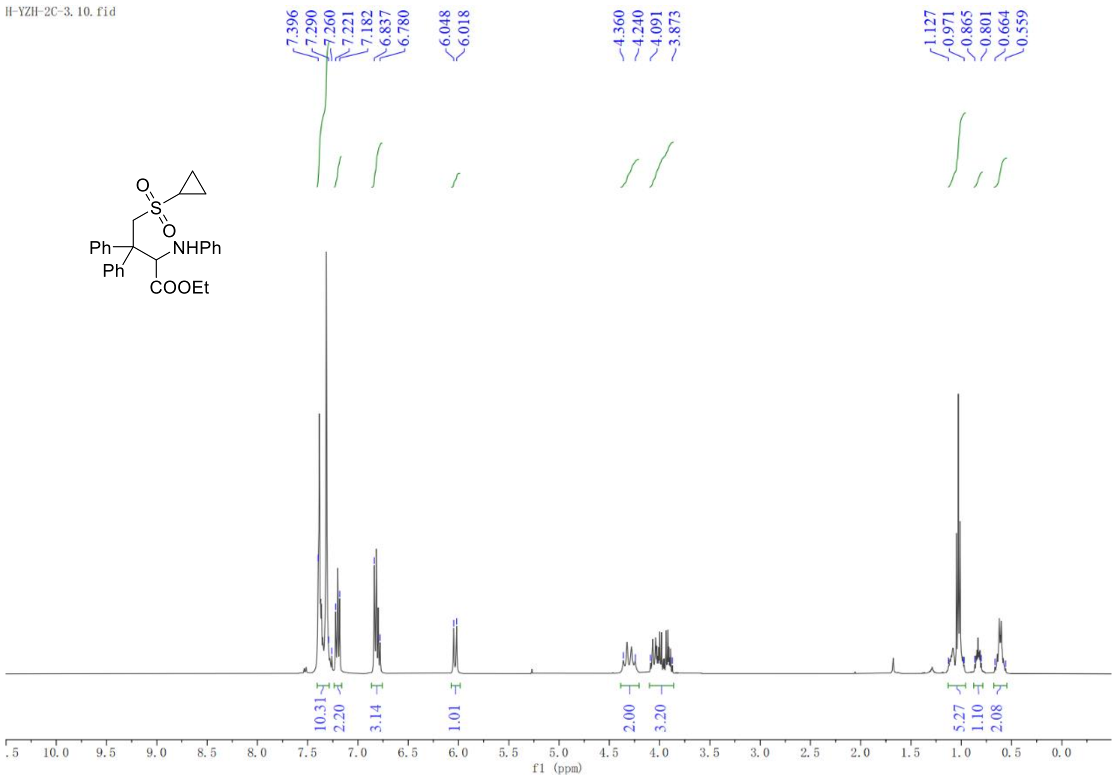

^{13}C NMR (101 MHz, $CDCl_3$) spectrum of **65**

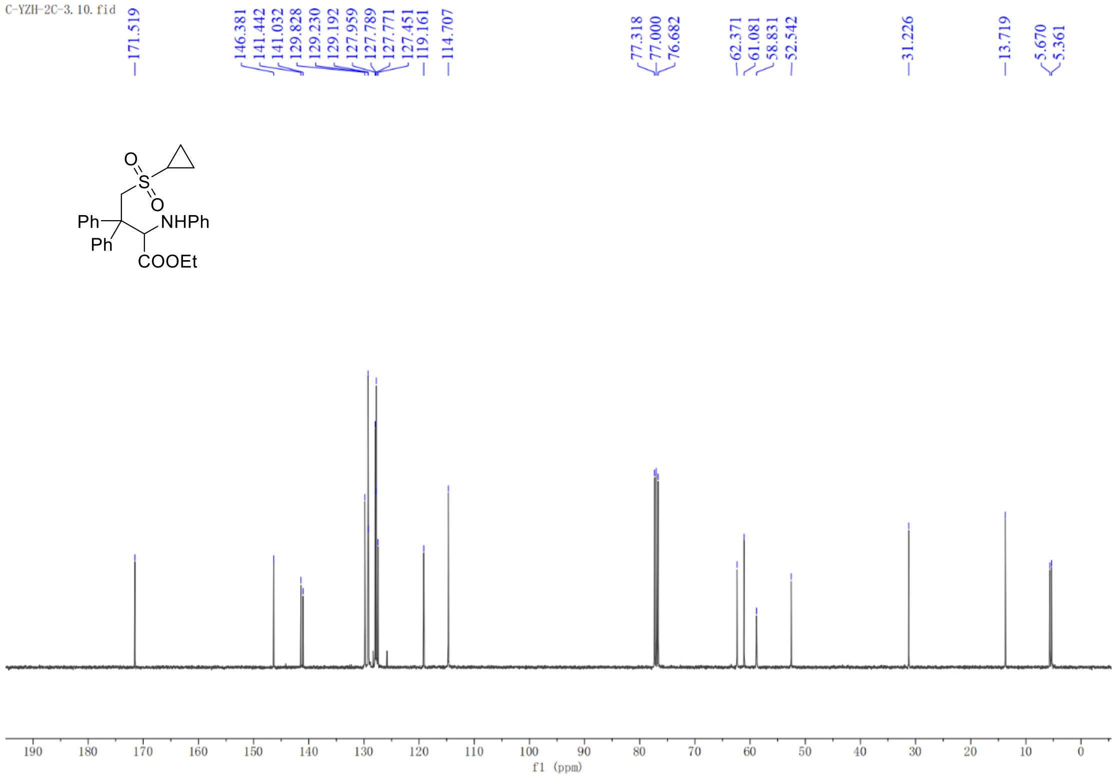

^{1}H NMR (400 MHz, $CDCl_3$) spectrum of **66**

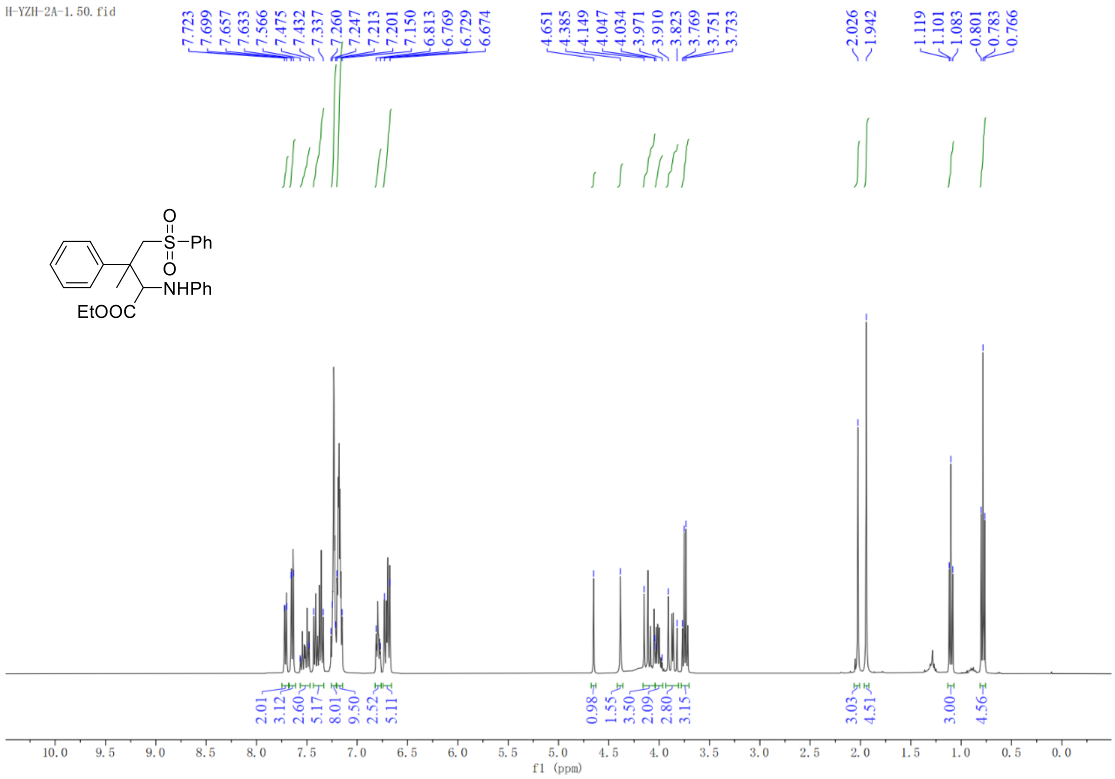

^{13}C NMR (101 MHz, $CDCl_3$) spectrum of **66**

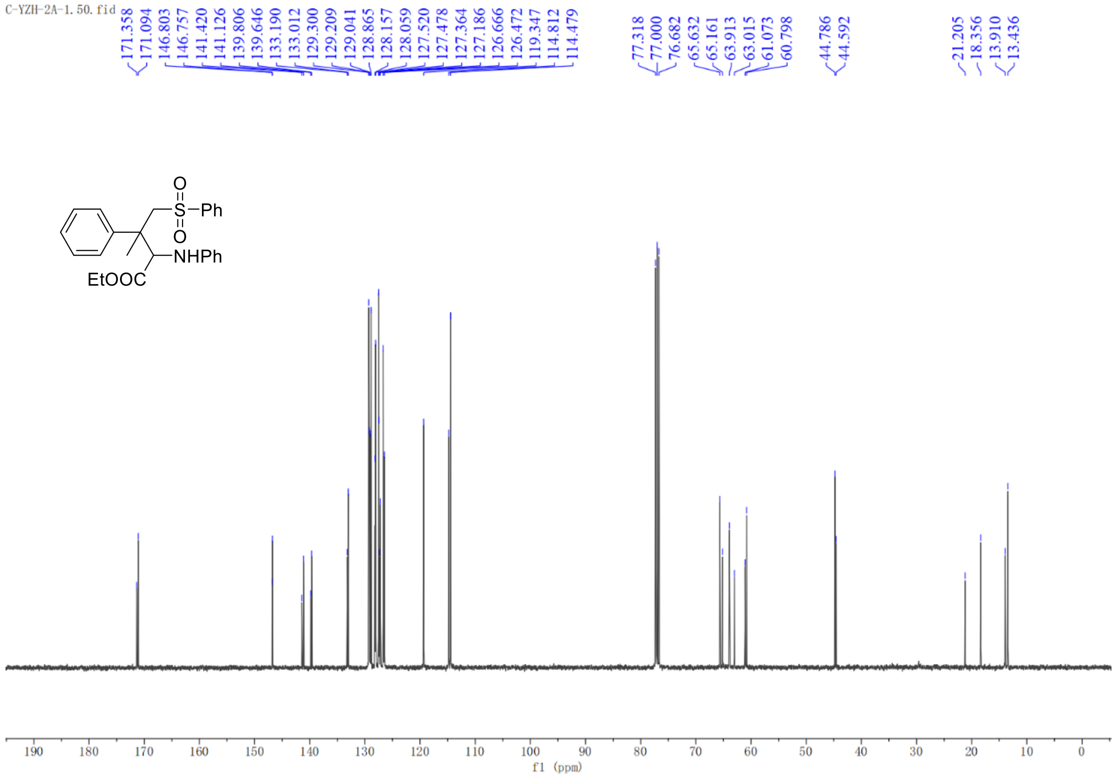

^{1}H NMR (400 MHz, $CDCl_3$) spectrum of **67** major isomer

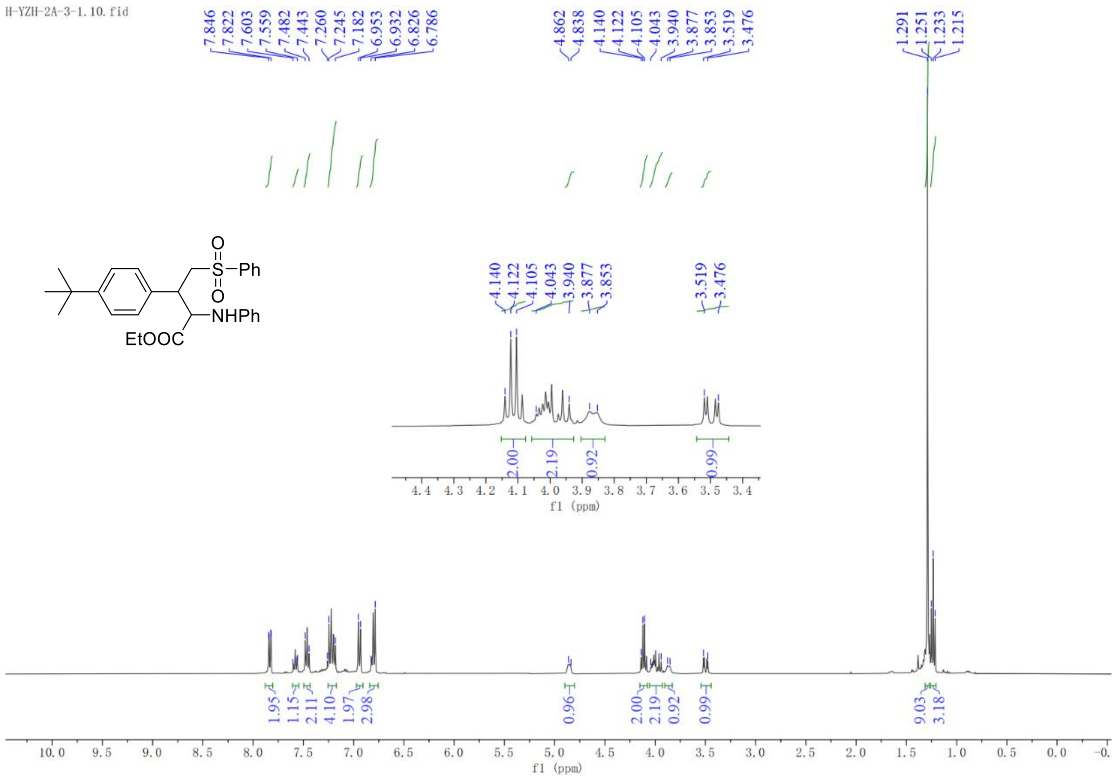

^{13}C NMR (101 MHz, $CDCl_3$) spectrum of **67** major isomer

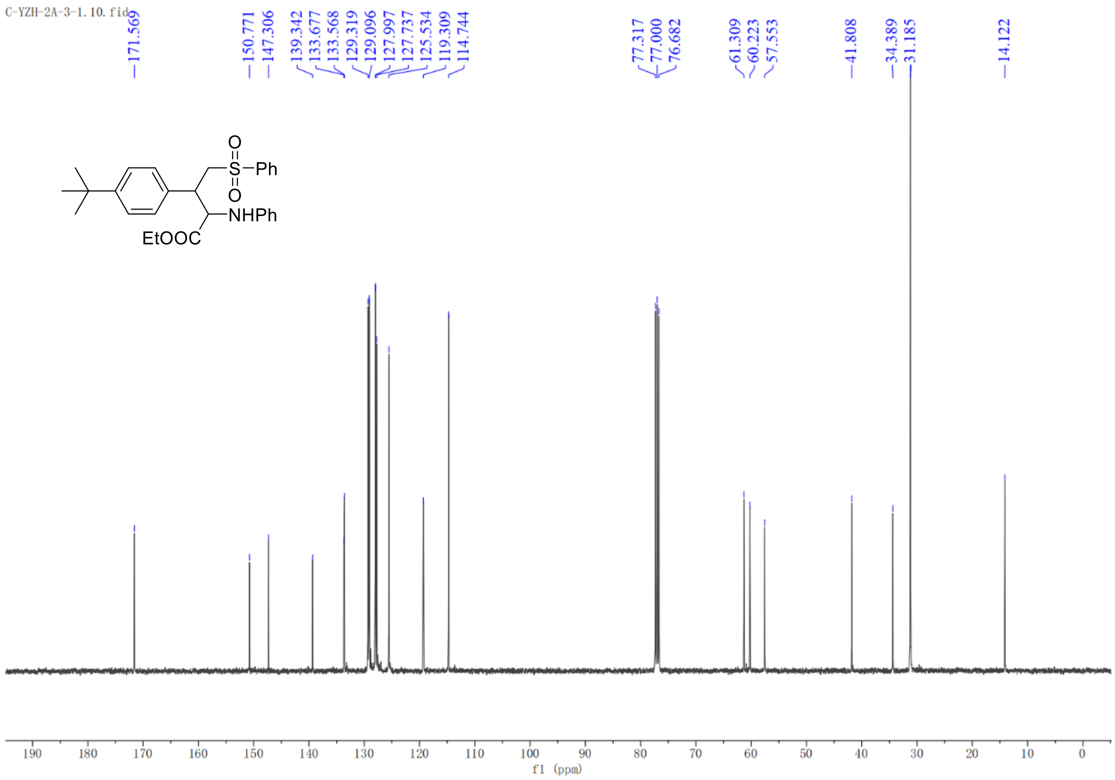

^{1}H NMR (400 MHz, $CDCl_3$) spectrum of **67** minor isomer

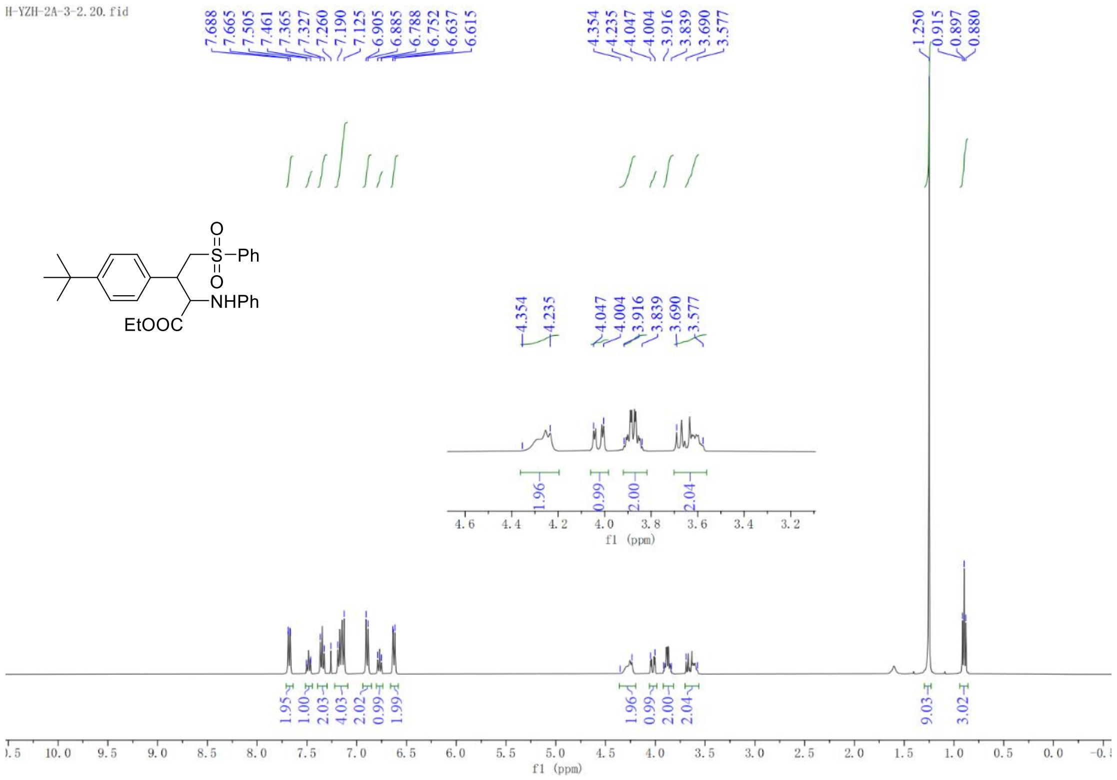

^{13}C NMR (101 MHz, $CDCl_3$) spectrum of **67** minor isomer

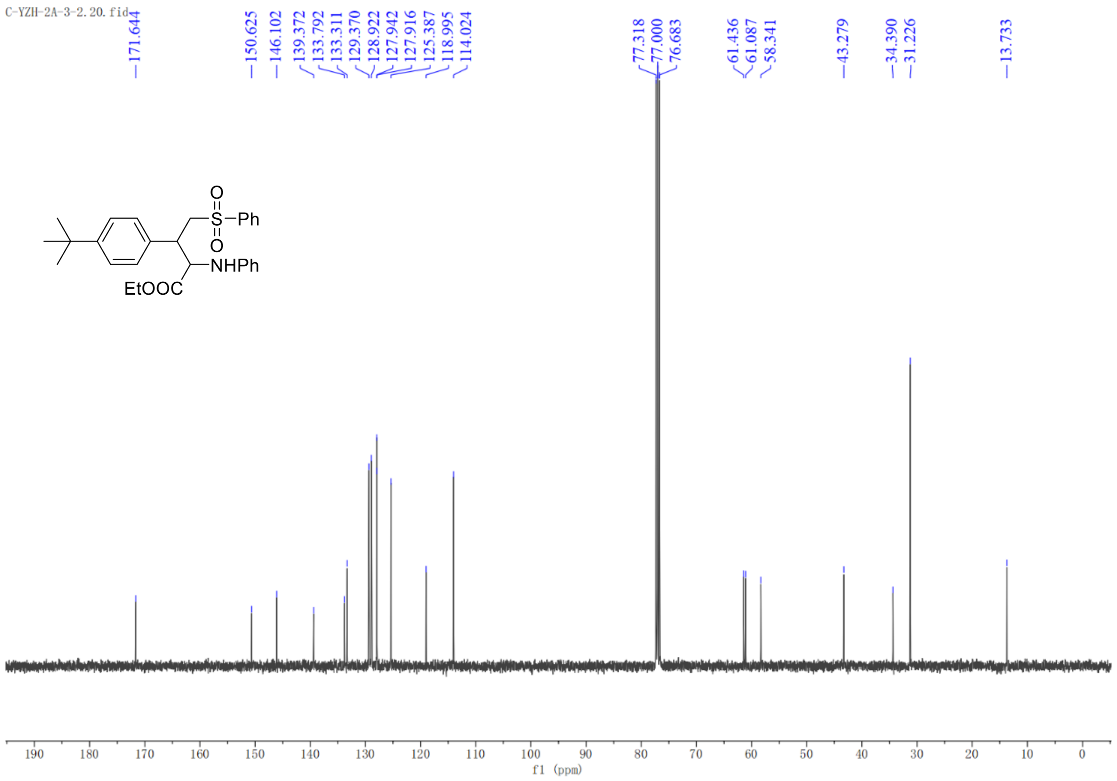

^{1}**H NMR** (400 MHz, $CDCl_3$) spectrum of **68**

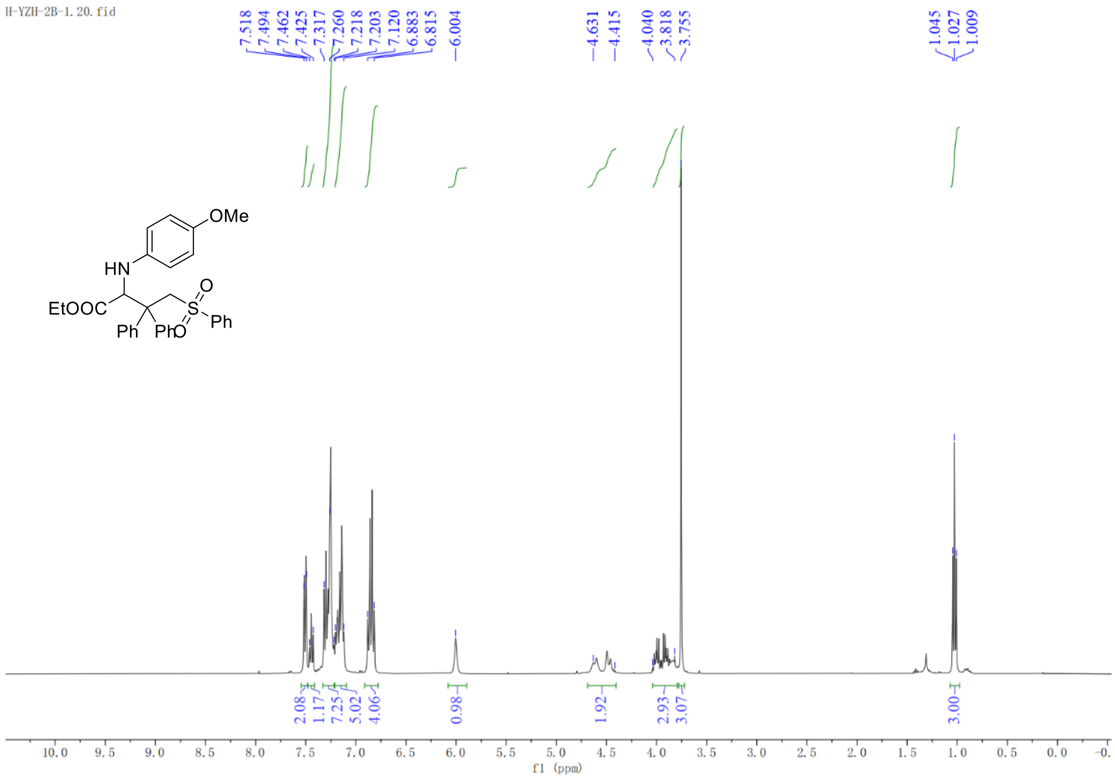

^{13}**C NMR** (101 MHz, $CDCl_3$) spectrum of **68**

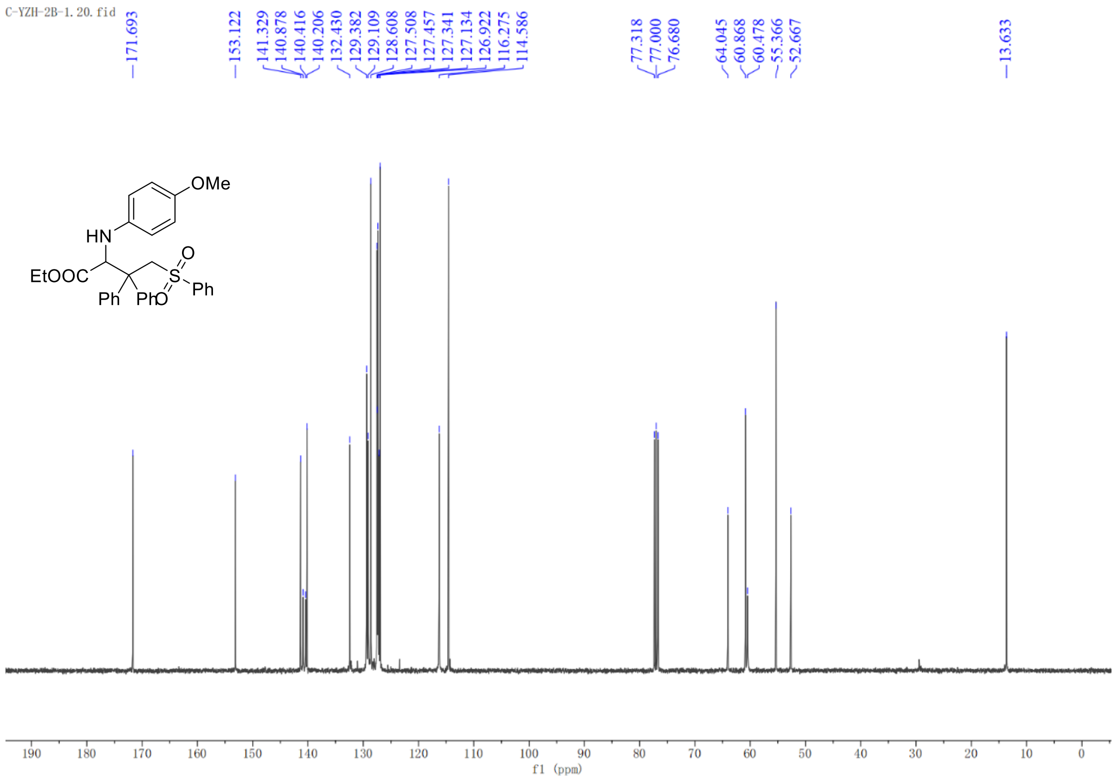

^{1}H NMR (400 MHz, $CDCl_3$) spectrum of **69**

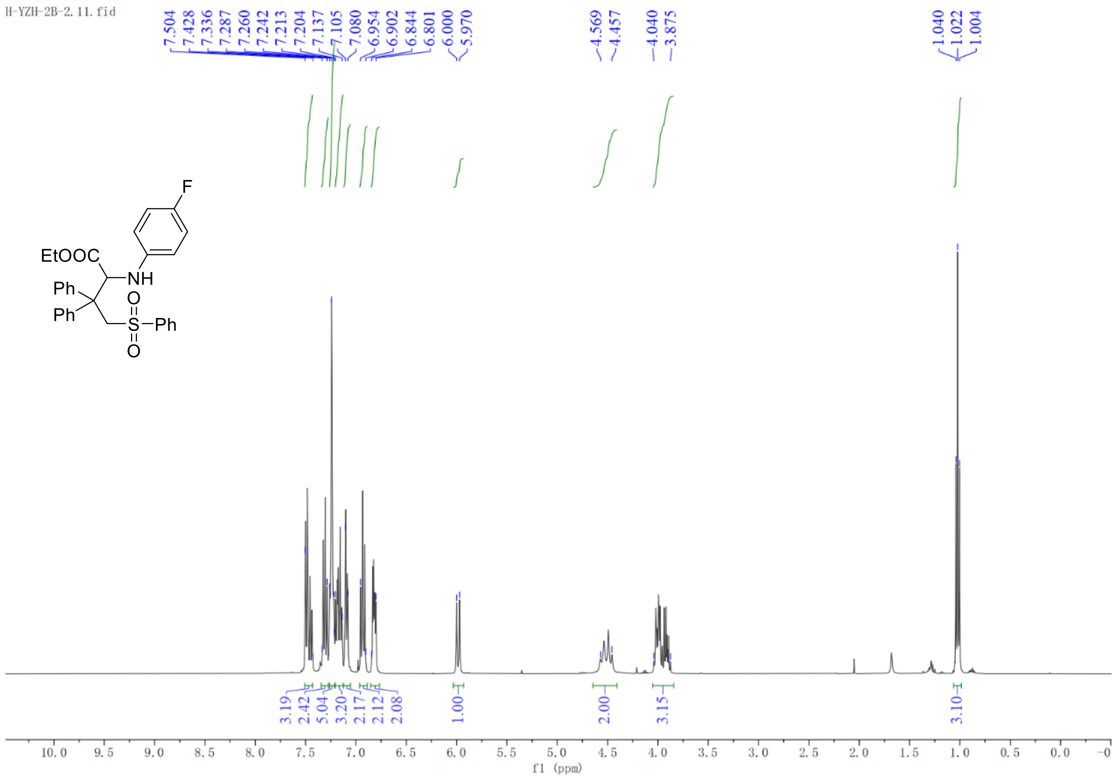

^{13}C NMR (101 MHz, $CDCl_3$) spectrum of **69**

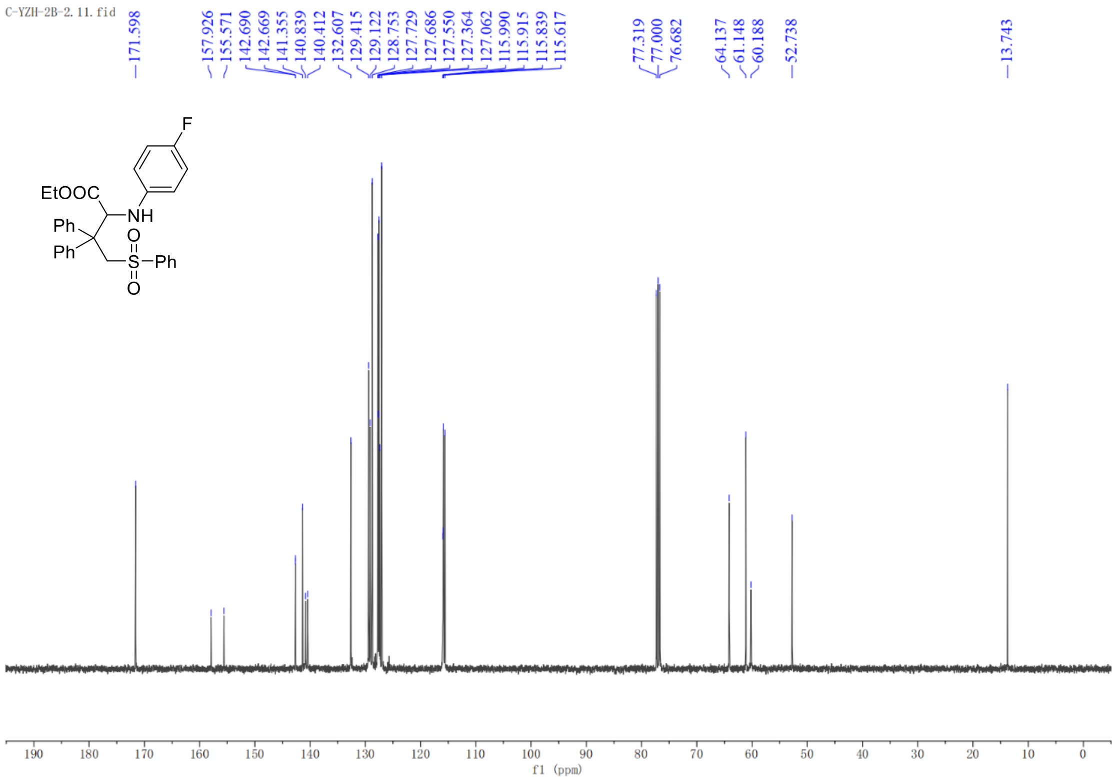

^{19}F NMR (376 MHz, CDCl$_3$) spectrum of **69**

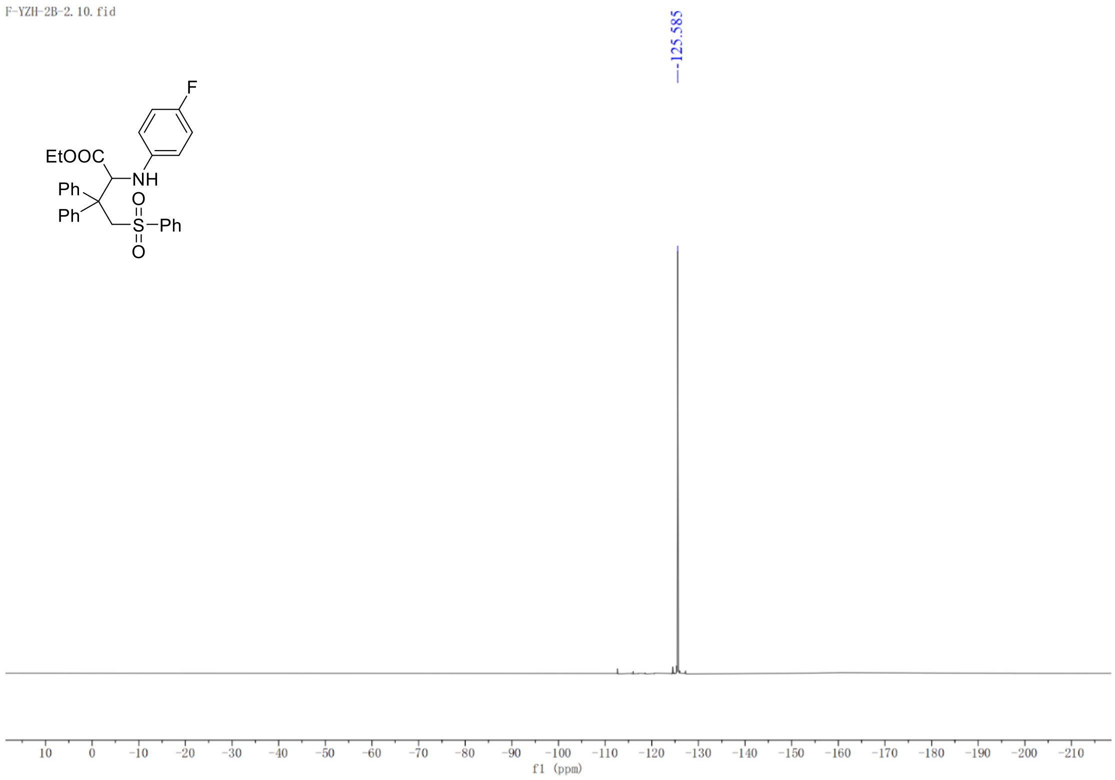

^{1}H NMR (400 MHz, CDCl$_3$) spectrum of **70**

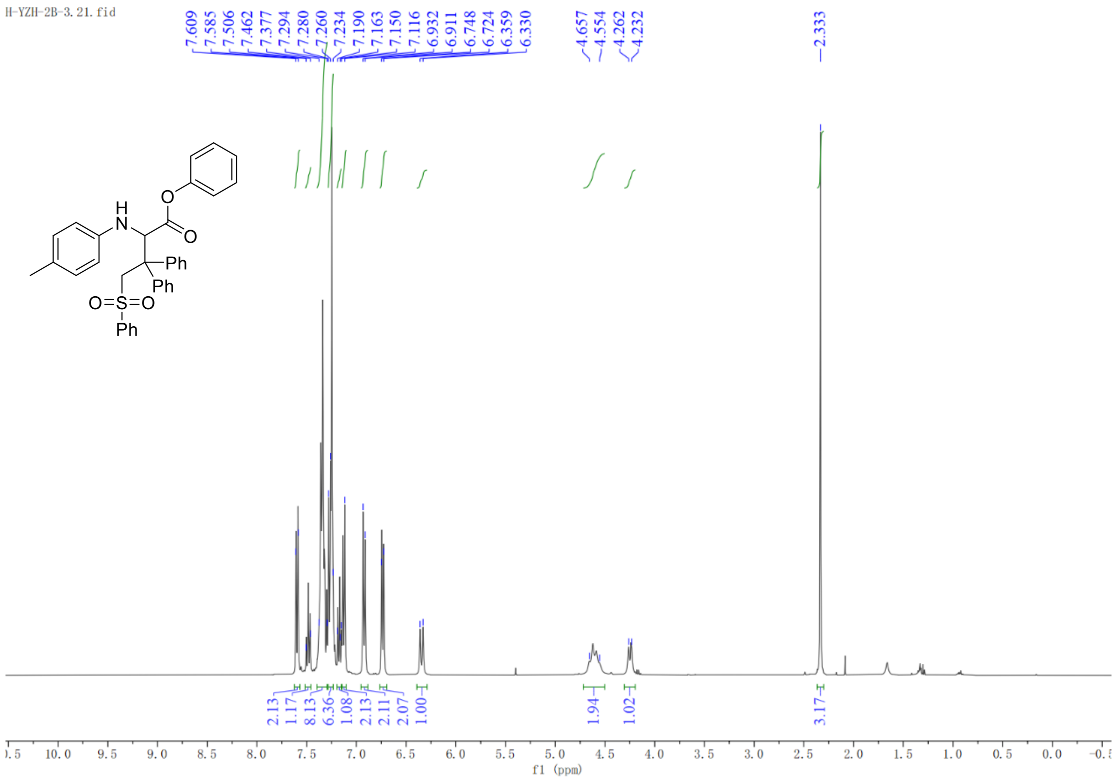

^{13}C NMR (101 MHz, $CDCl_3$) spectrum of **70**

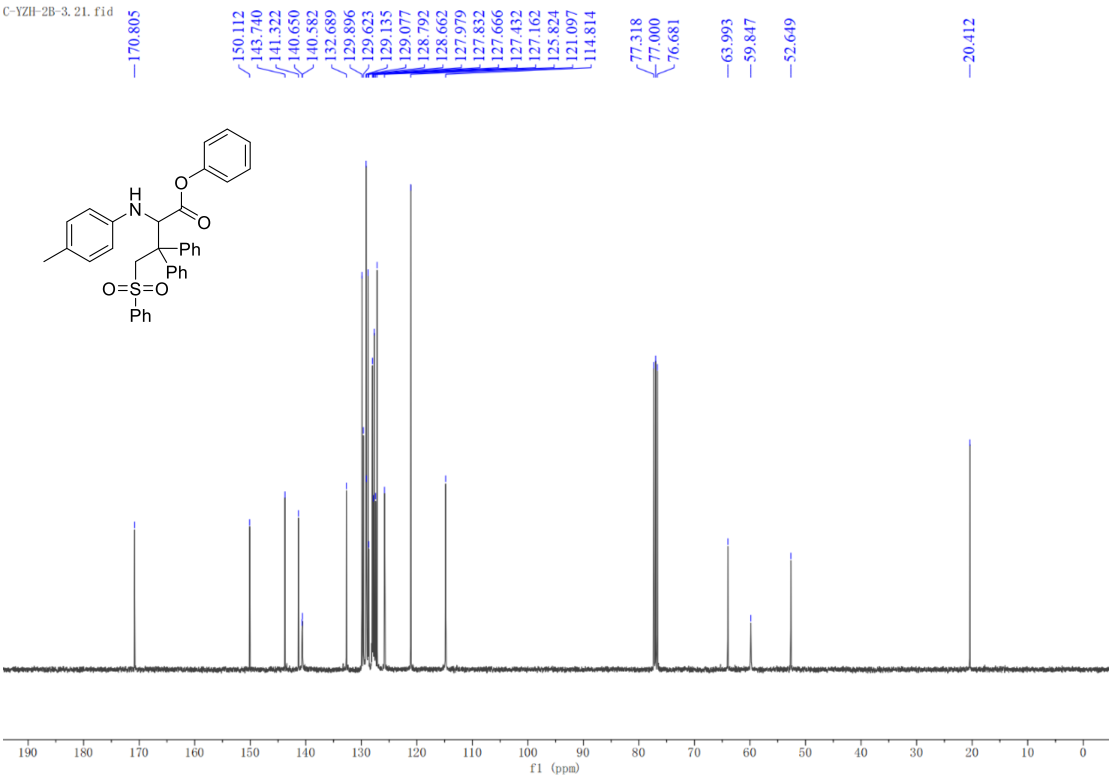

^{1}H NMR (400 MHz, $CDCl_3$) spectrum of **71**

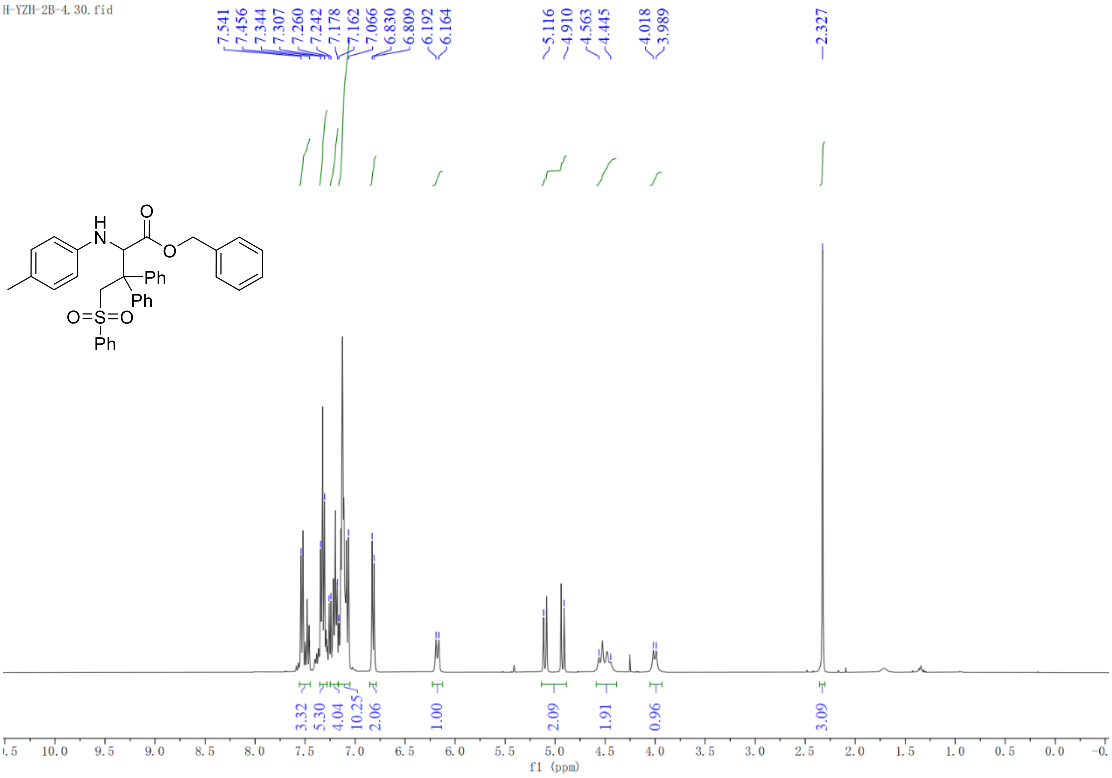

^{13}C NMR (101 MHz, $CDCl_3$) spectrum of **71**

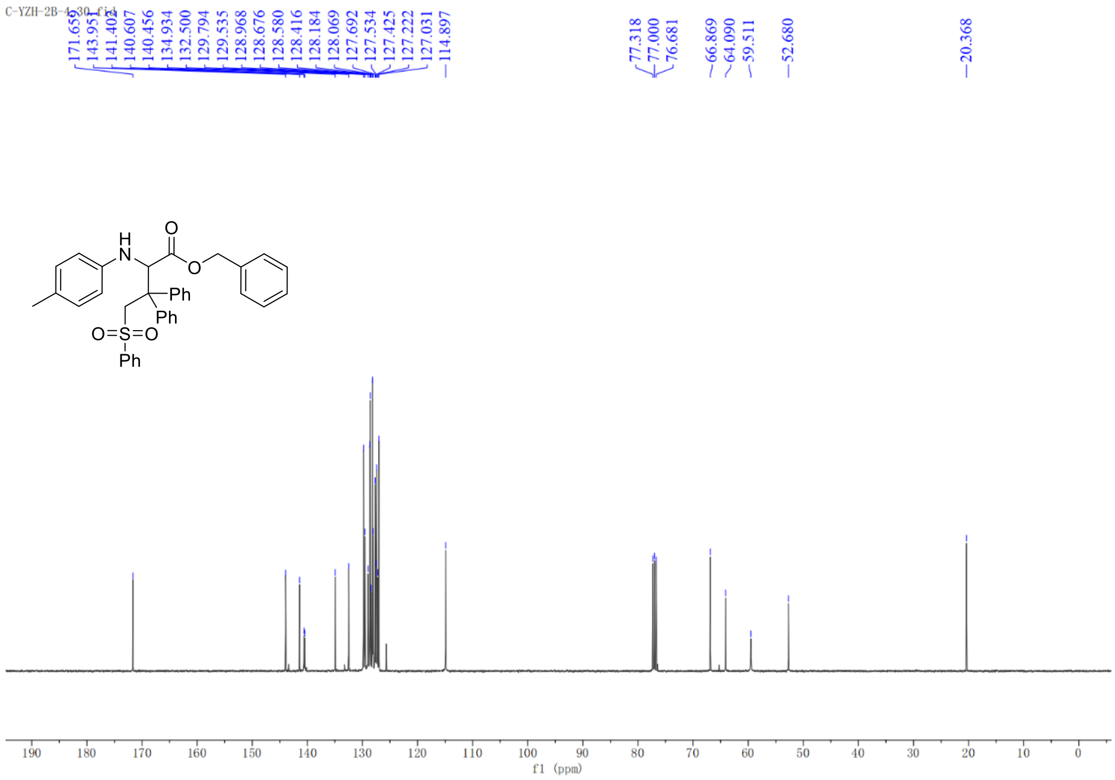

^{1}H NMR (400 MHz, $CDCl_3$) spectrum of **72**

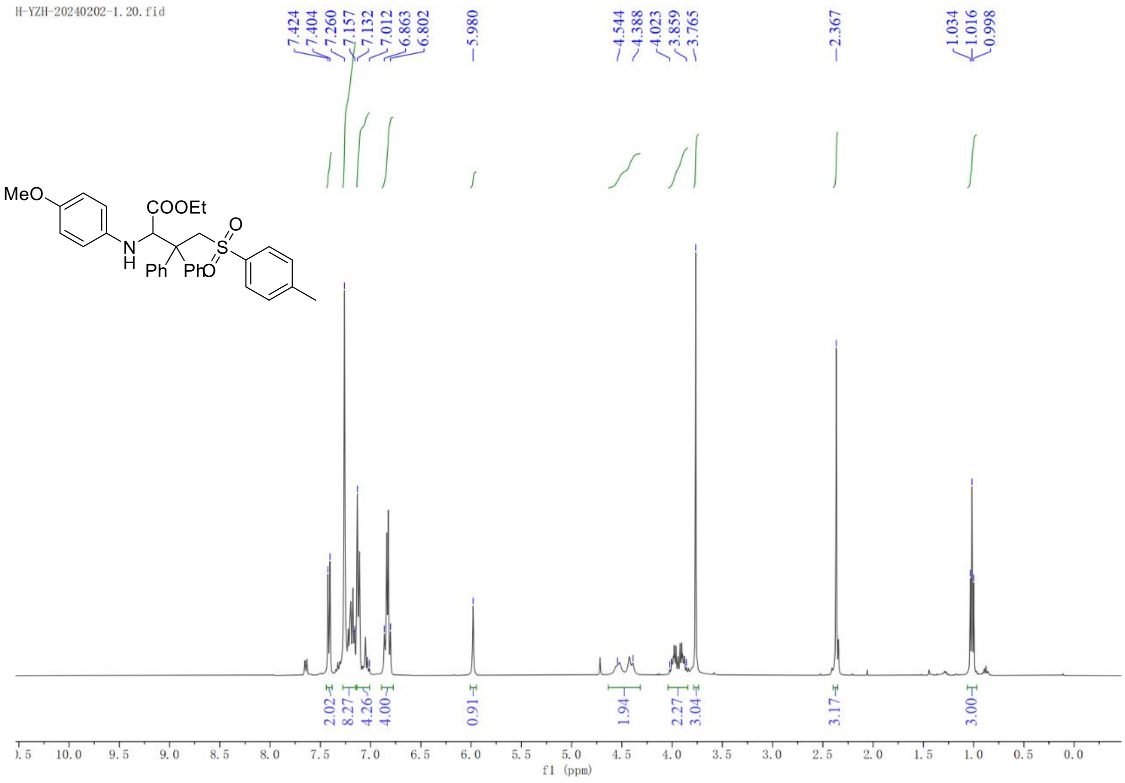

^{13}C NMR (101 MHz, $CDCl_3$) spectrum of **72**

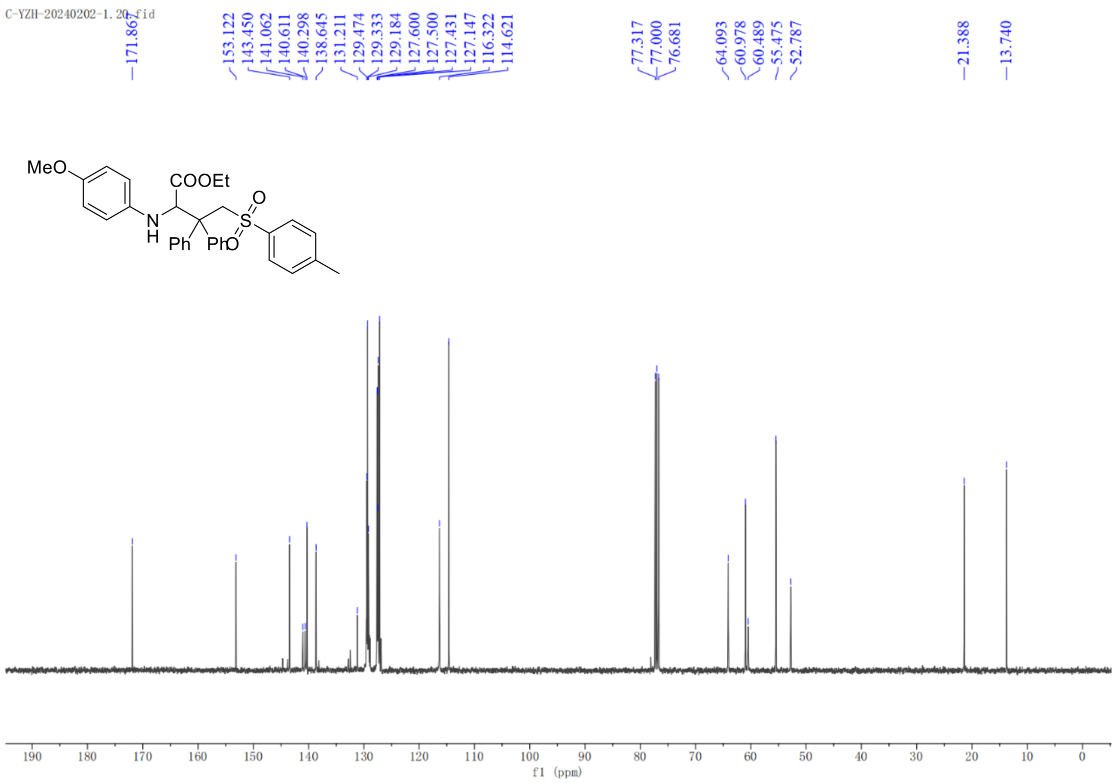

^{1}H NMR (400 MHz, $CDCl_3$) spectrum of **73**

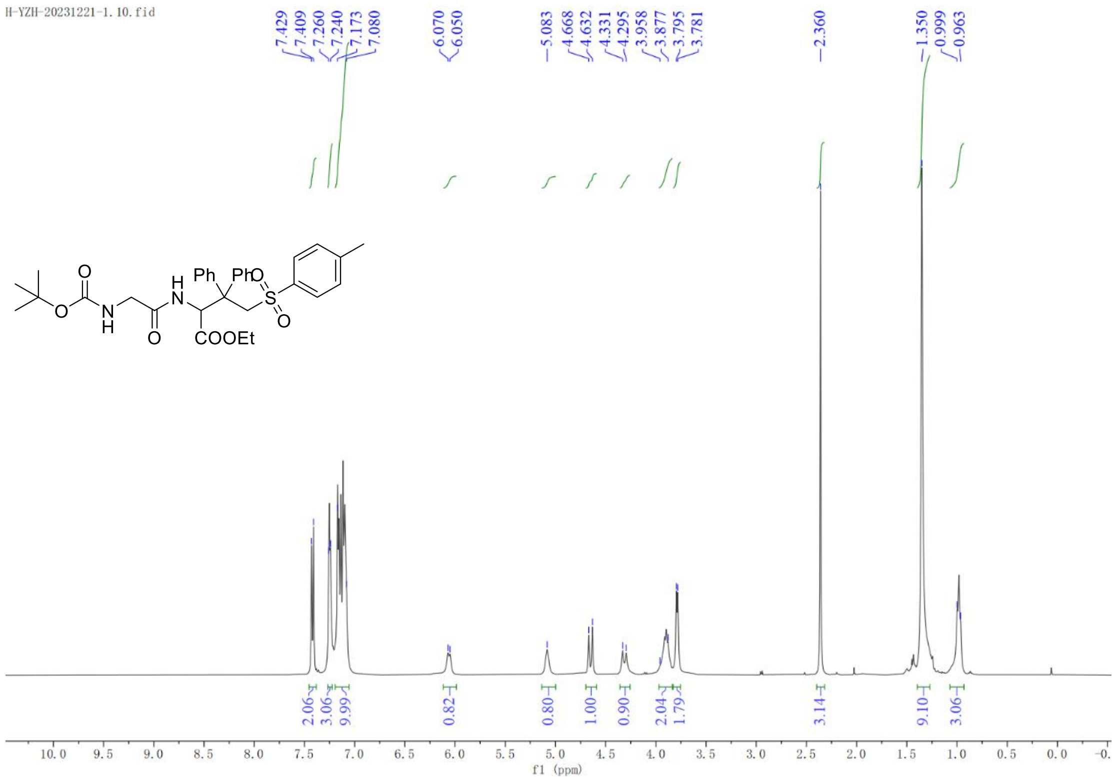

^{13}C NMR (101 MHz, $CDCl_3$) spectrum of **73**

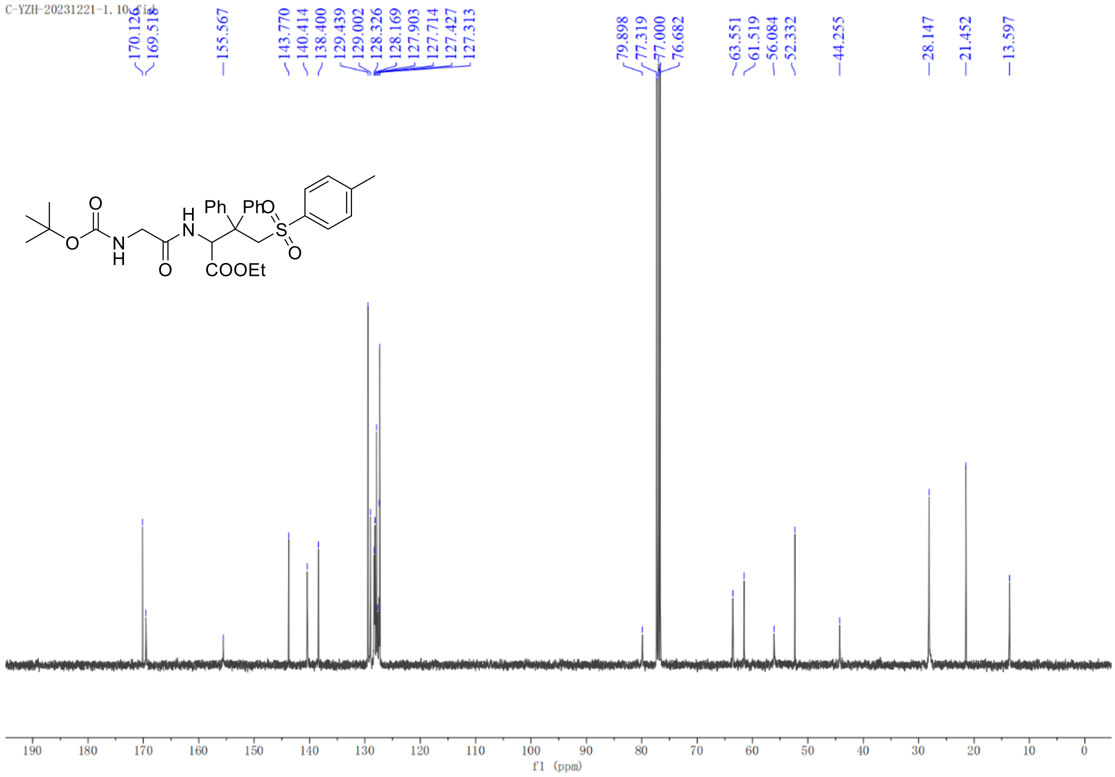

^{1}H NMR (400 MHz, $CDCl_3$) spectrum of **74**

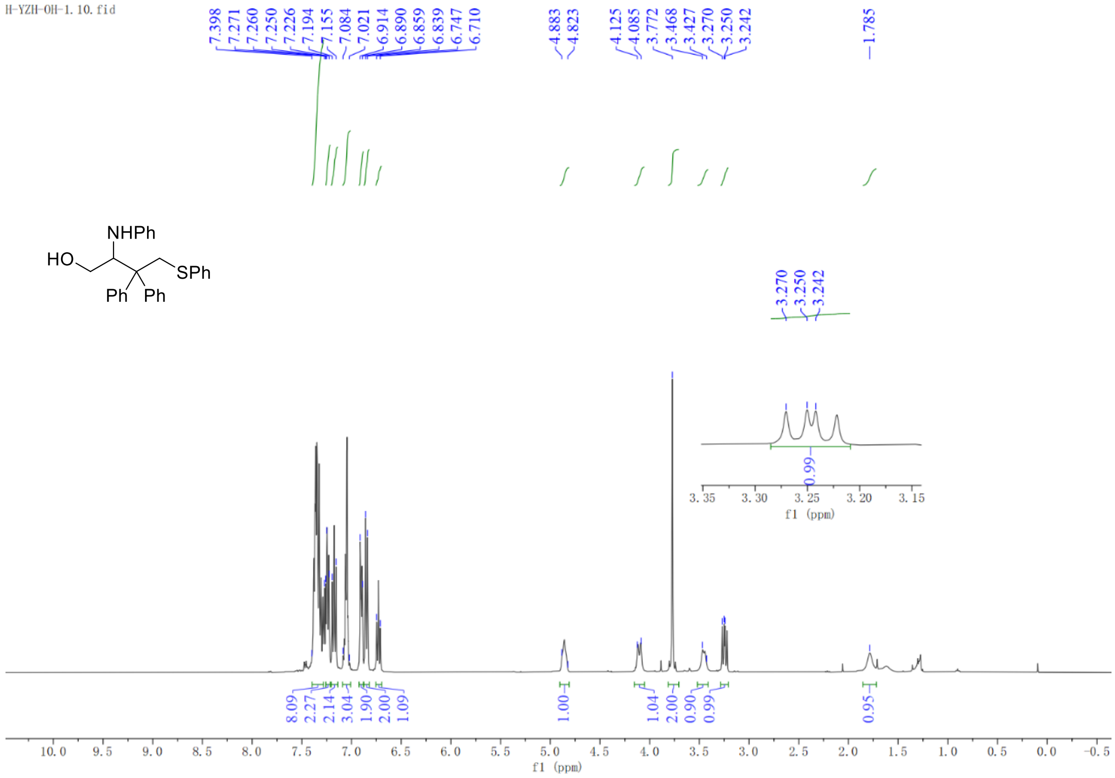

^{13}C NMR (101 MHz, $CDCl_3$) spectrum of **74**

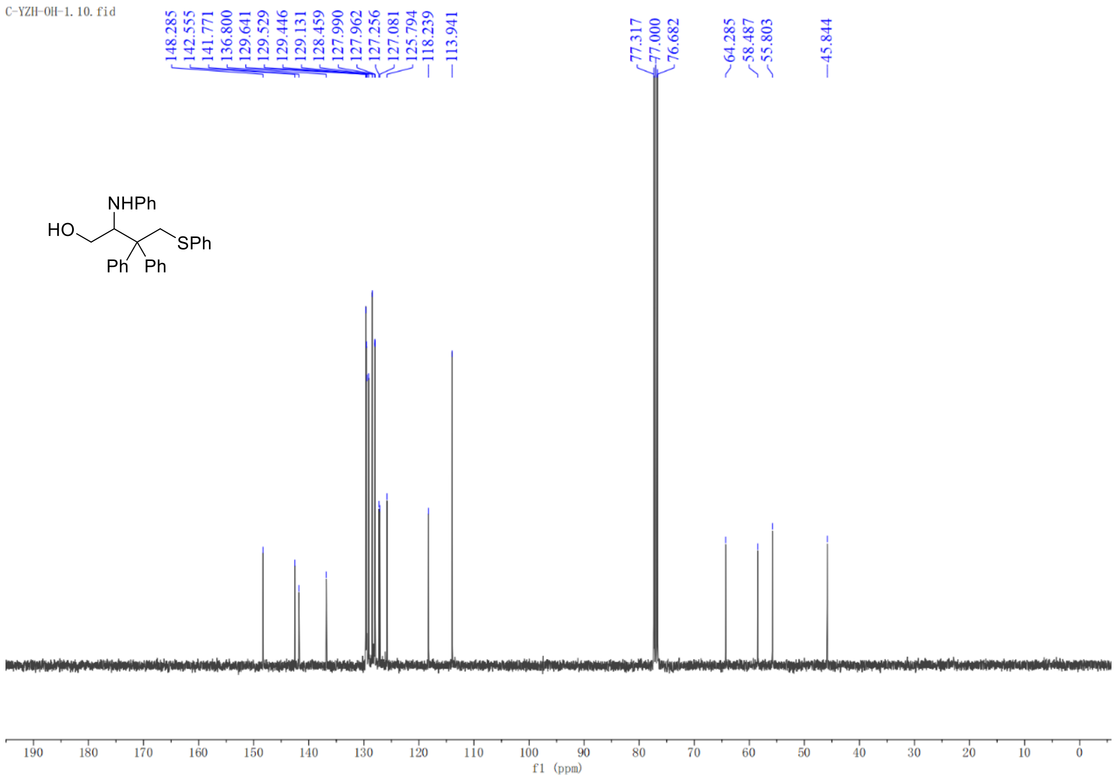

^{1}H NMR (400 MHz, $CDCl_3$) spectrum of **77**

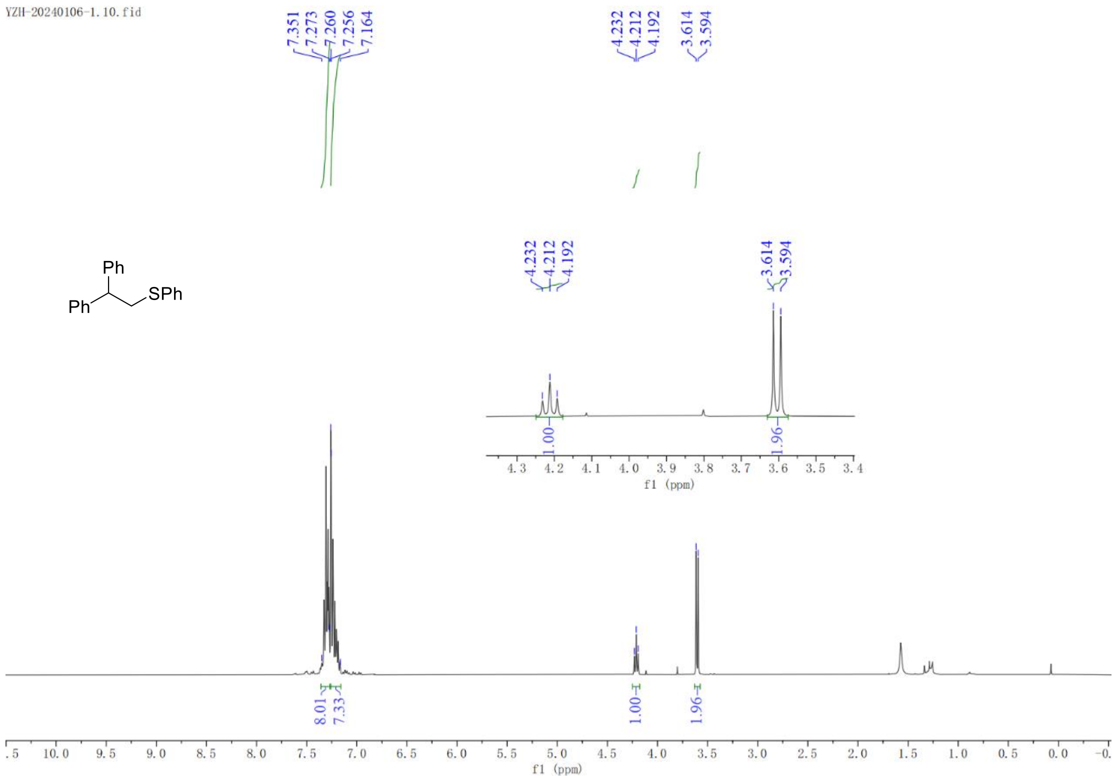

^{13}C NMR (101 MHz, $CDCl_3$) spectrum of **77**

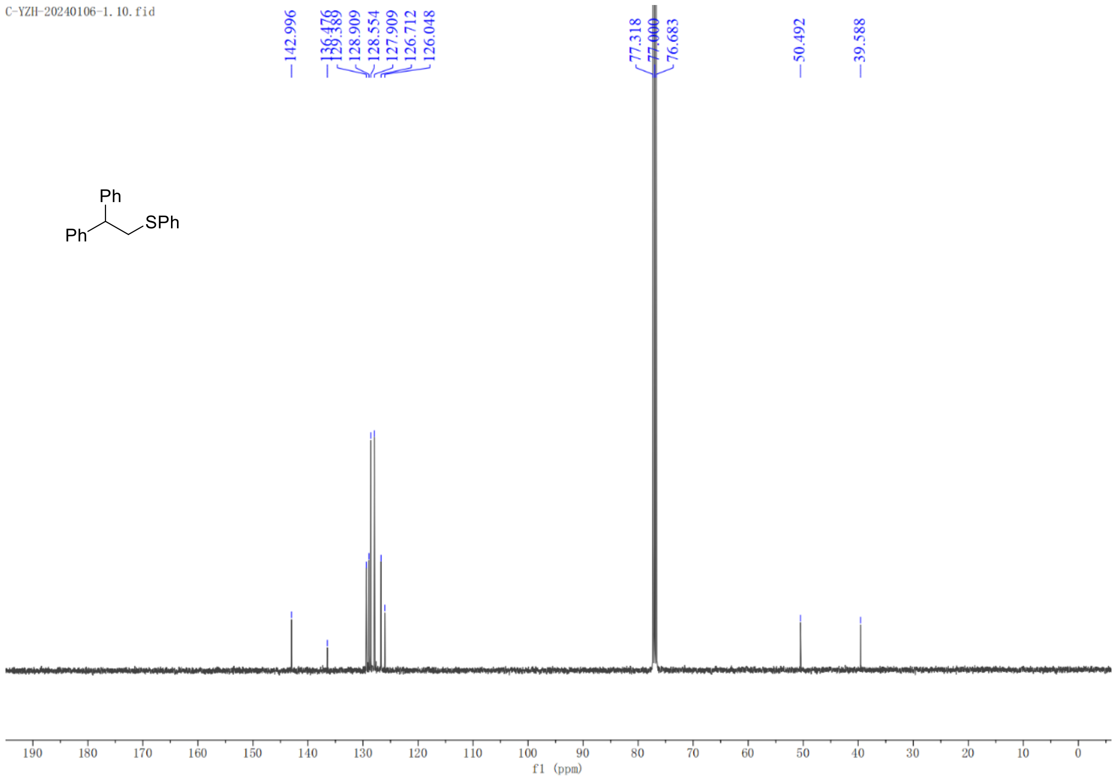

^{1}H NMR (400 MHz, $CDCl_3$) spectrum of **78**

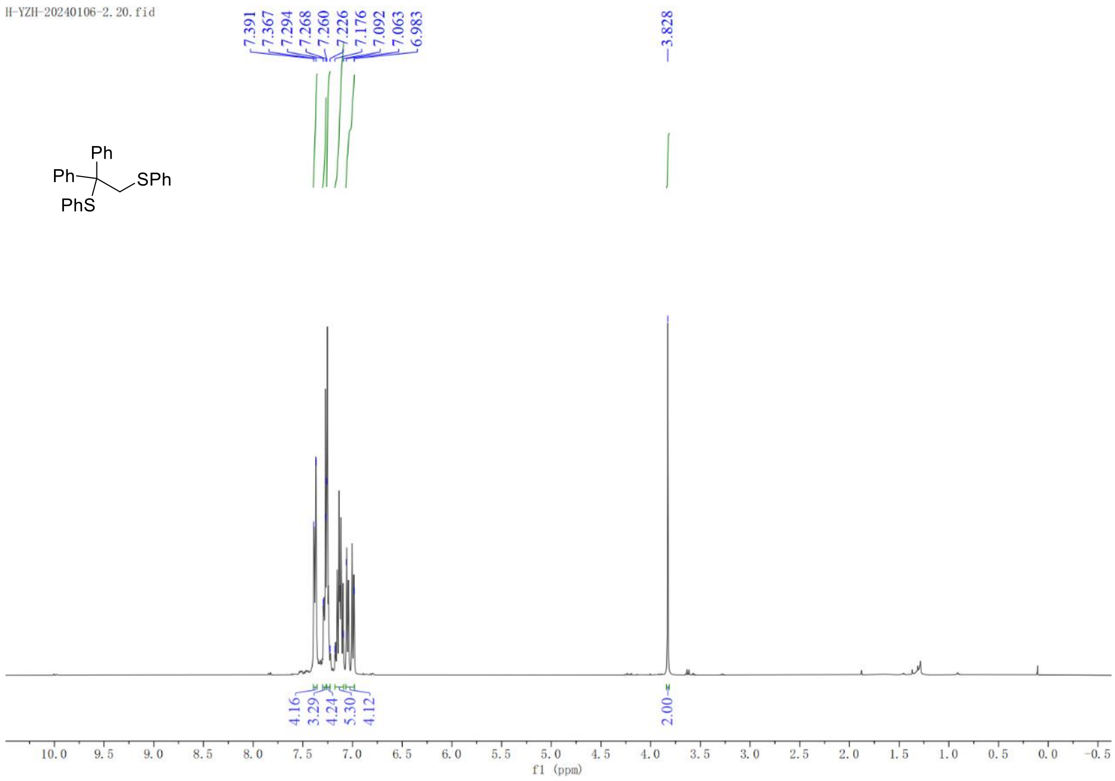

^{13}C NMR (101 MHz, $CDCl_3$) spectrum of **78**

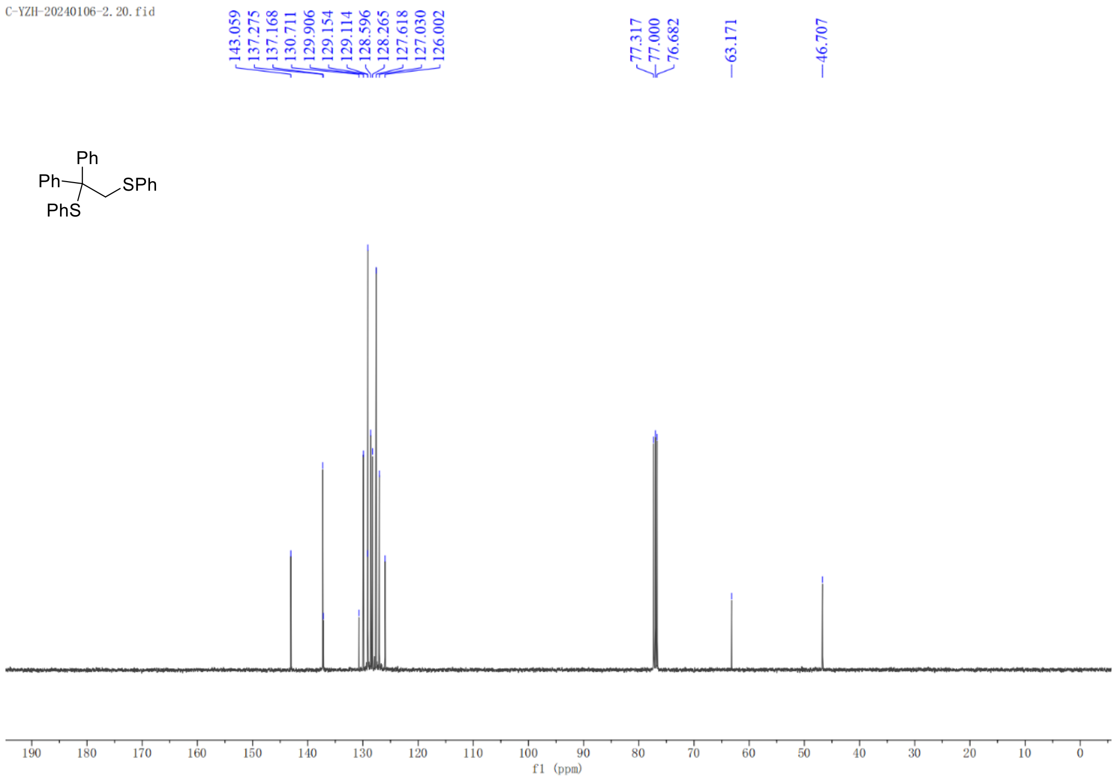

^{1}H NMR (400 MHz, $CDCl_3$) spectrum of **79**

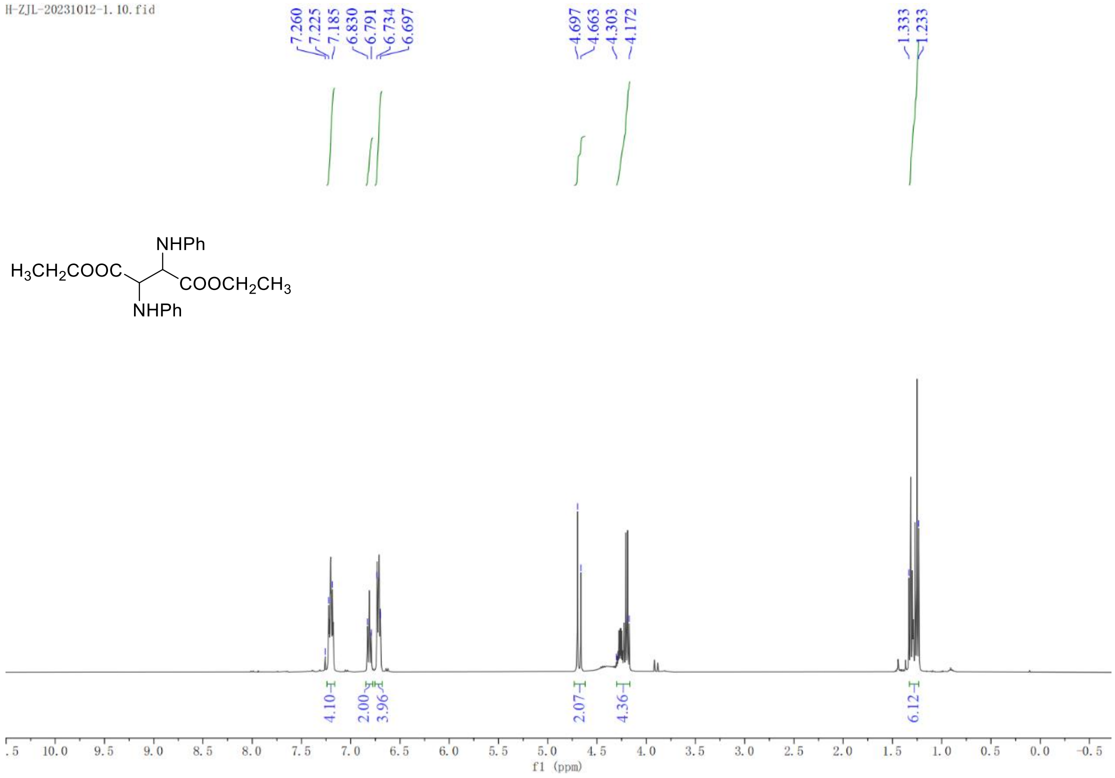

^{13}C NMR (101 MHz, $CDCl_3$) spectrum of **79**

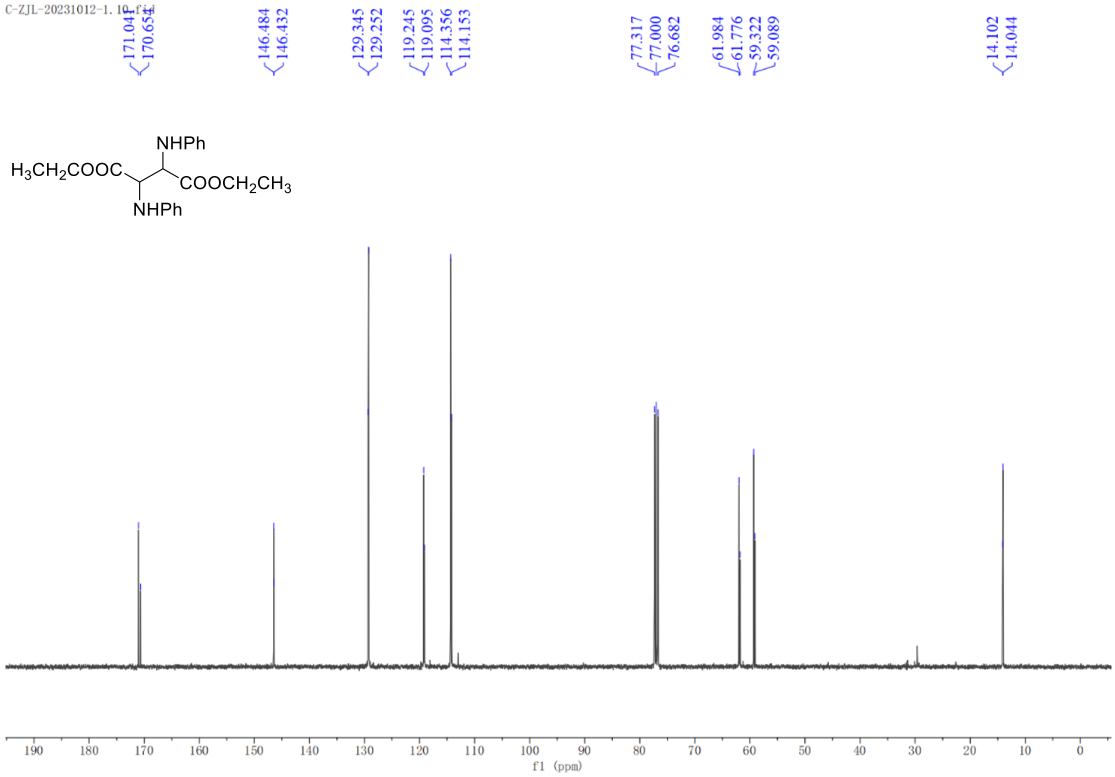

Supplement: Supplementary file 1 — Supporting Information [file ADVS-11-2402428-s002.pdf]
