# Supplementary material for: Stabilized Carbon‐Centered Radical‐Mediated Carbosulfenylation of Styrenes: Modular Synthesis of Sulfur‐Containing Glycine and Peptide Derivatives
Source: Adv Sci (Weinh). 2024 Jun 9;11(29):2402428. doi: 10.1002/advs.202402428 (PMC11304285; doi:10.1002/advs.202402428)

checkCIF/PLATON report

Structure factors have been supplied for datablock(s) mo_yzh1_0m_a

THIS REPORT IS FOR GUIDANCE ONLY. IF USED AS PART OF A REVIEW PROCEDURE FOR PUBLICATION, IT SHOULD NOT REPLACE THE EXPERTISE OF AN EXPERIENCED CRYSTALLOGRAPHIC REFEREE.

No syntax errors found.   CIF dictionary   Interpreting this report

Datablock: mo_yzh1_0m_a

```
Bond precision:   C-C = 0.0037 A          Wavelength=0.71073

Cell:           a=9.712(3)          b=11.760(4)       c=12.338(4)
                alpha=108.416(4)    beta=110.622(4)   gamma=91.533(4)
Temperature:    296 K
```

	Calculated	Reported
Volume	1236.1(7)	1236.2(6)
Space group	P -1	P -1
Hall group	-P 1	-P 1
Moiety formula	C30 H29 N O2 S	C30 H29 N O2 S
Sum formula	C30 H29 N O2 S	C30 H29 N O2 S
Mr	467.60	467.60
Dx,g cm-3	1.256	1.256
Z	2	2
Mu (mm-1)	0.159	0.158
F000	496.0	496.0
F000’	496.44	
h,k,lmax	12,15,16	12,15,16
Nref	5742	5654
Tmin,Tmax	0.994,0.998	0.686,0.746
Tmin’	0.992	

```
Correction method= # Reported T Limits: Tmin=0.686 Tmax=0.746
AbsCorr = MULTI-SCAN

Data completeness= 0.985            Theta(max)= 27.595

R(reflections)= 0.0539( 4594)                        wR2(reflections)=
                                                     0.1715( 5654)
S = 1.040                    Npar= 308
```

The following ALERTS were generated. Each ALERT has the format
test-name_ALERT_alert-type_alert-level.
Click on the hyperlinks for more details of the test.

Alert level C

```
PLAT414_ALERT_2_C Short Intra D-H..H-X       H004     ..H00A     .       1.96 Ang.
                                                    x,y,z  =      1_555 Check
PLAT911_ALERT_3_C Missing FCF Refl Between Thmin & STh/L=    0.600         34 Report
PLAT975_ALERT_2_C Check Calcd Resid. Dens.  0.97Ang From N004    .       0.57 eA-3
```

Alert level G

```
PLAT007_ALERT_5_G Number of Unrefined Donor-H Atoms .............          1 Report
PLAT154_ALERT_1_G The s.u.'s on the Cell Angles are Equal ..(Note)      0.004 Degree
PLAT720_ALERT_4_G Number of Unusual/Non-Standard Labels ..........         62 Note
PLAT883_ALERT_1_G No Info/Value for _atom_sites_solution_primary .     Please Do !
PLAT912_ALERT_4_G Missing # of FCF Reflections Above STh/L=  0.600         55 Note
PLAT933_ALERT_2_G Number of HKL-OMIT Records in Embedded .res File          5 Note
PLAT941_ALERT_3_G Average HKL Measurement Multiplicity ...........        3.1 Low
PLAT978_ALERT_2_G Number C-C Bonds with Positive Residual Density.         10 Info
```

0 **ALERT level A** = Most likely a serious problem - resolve or explain
0 **ALERT level B** = A potentially serious problem, consider carefully
3 **ALERT level C** = Check. Ensure it is not caused by an omission or oversight
8 **ALERT level G** = General information/check it is not something unexpected

2 ALERT type 1 CIF construction/syntax error, inconsistent or missing data
4 ALERT type 2 Indicator that the structure model may be wrong or deficient
2 ALERT type 3 Indicator that the structure quality may be low
2 ALERT type 4 Improvement, methodology, query or suggestion
1 ALERT type 5 Informative message, check

It is advisable to attempt to resolve as many as possible of the alerts in all categories. Often the minor alerts point to easily fixed oversights, errors and omissions in your CIF or refinement strategy, so attention to these fine details can be worthwhile. In order to resolve some of the more serious problems it may be necessary to carry out additional measurements or structure refinements. However, the purpose of your study may justify the reported deviations and the more serious of these should normally be commented upon in the discussion or experimental section of a paper or in the "special_details" fields of the CIF. checkCIF was carefully designed to identify outliers and unusual parameters, but every test has its limitations and alerts that are not important in a particular case may appear. Conversely, the absence of alerts does not guarantee there are no aspects of the results needing attention. It is up to the individual to critically assess their own results and, if necessary, seek expert advice.

Publication of your CIF in IUCr journals

A basic structural check has been run on your CIF. These basic checks will be run on all CIFs submitted for publication in IUCr journals (*Acta Crystallographica*, *Journal of Applied Crystallography*, *Journal of Synchrotron Radiation*); however, if you intend to submit to *Acta Crystallographica Section C* or *E* or *IUCrData*, you should make sure that full publication checks are run on the final version of your CIF prior to submission.

Publication of your CIF in other journals

Please refer to the *Notes for Authors* of the relevant journal for any special instructions relating to CIF submission.

PLATON version of 10/05/2023; check.def file version of 10/05/2023

Datablock mo_yzh1_0m_a - ellipsoid plot

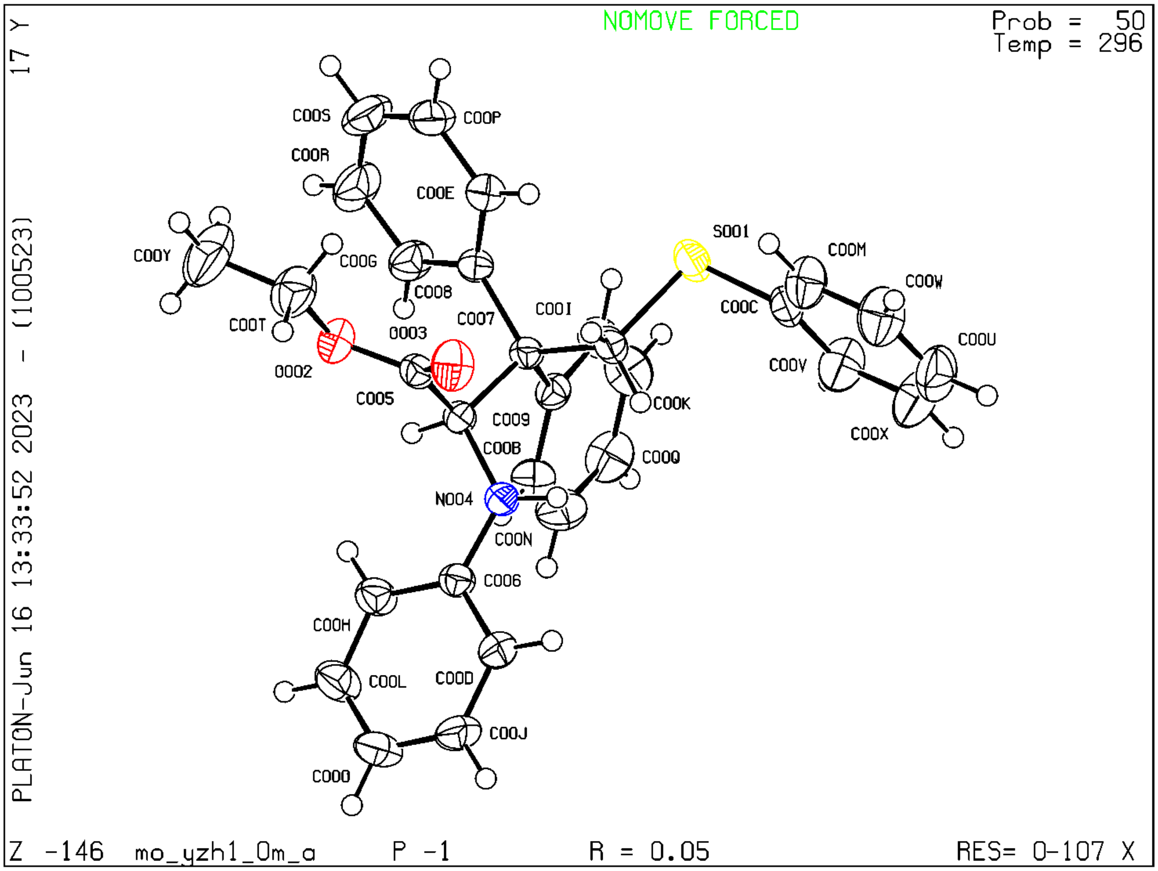

Supplement: Supplementary file 2 — Supporting Information [file ADVS-11-2402428-s001.zip › advs202402428-sup-0002-SuppMat/checkcif.pdf]
